# Guiding a divergent reaction by photochemical control: bichromatic selective access to levulinates and butenolides†
†Electronic supplementary information (ESI) available. See DOI: 10.1039/c7sc05094a


**DOI:** 10.1039/c7sc05094a

**Published:** 2017-12-18

**Authors:** Revannath L. Sutar, Saumik Sen, Or Eivgi, Gal Segalovich, Igor Schapiro, Ofer Reany, N. Gabriel Lemcoff

**Affiliations:** a Department of Chemistry , Ben-Gurion University of the Negev , Beer-Sheva 84105 , Israel . Email: lemcoff@bgu.ac.il; b Department of Natural Sciences , The Open University of Israel , Ra'anana , 43537 , Israel; c Fritz Haber Center for Molecular Dynamics , Institute of Chemistry , The Hebrew University of Jerusalem , Jerusalem , 91904 , Israel; d Ilse Katz Institute for Nanoscale Science and Technology , Ben-Gurion University of the Negev , Beer-Sheva , 84105 , Israel

## Abstract

Allylic and acrylic substrates may be efficiently transformed by a sequential bichromatic photochemical process into derivatives of levulinates or butenolides with high selectivity when phenanthrene is used as a regulator. Thus, UV-A photoinduced cross-metathesis (CM) couples the acrylic and allylic counterparts and subsequent UV-C irradiation initiates *E*–*Z* isomerization of the carbon–carbon double bond, followed by one of two competing processes; namely, cyclization by transesterification or a 1,5-H shift and tautomerization. Quantum chemical calculations demonstrate that intermediates are strongly blue-shifted for the cyclization while red-shifted for the 1,5-H shift reaction. Hence, delaying the double bond migration by employing UV-C absorbing phenanthrene, results in a selective novel divergent all-photochemical pathway for the synthesis of fundamental structural motifs of ubiquitous natural products.

## Abstract 2

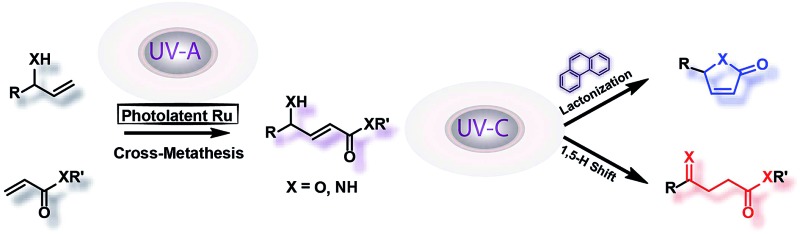
Allylic and acrylic substrates may be efficiently transformed by a sequential bichromatic photochemical process into derivatives of levulinates or butenolides with high selectivity when phenanthrene is used as a regulator.

## Introduction

Chemistry with light provides a range of creative synthetic possibilities.^1^ Many chemical transformations promoted by light, such as photosynthesis, the *cis*–*trans* photoisomerization of the protonated Schiff base of 11-*cis*-retinal and the synthesis of vitamin D are vital for the existence of life on Earth.^2^ Conversely, photocycloadditions caused by UV radiation, may result in DNA damage leading to cancer.^3^ To safeguard us from this, perhaps mimicking organisms that evolved UV protecting molecules,^4^ chemists have developed sunscreens,^5^ which deflect or absorb these harmful radiations. Inspired by the way evolution has selected molecules to block certain UV wavelengths to follow specific biochemical pathways,^6^ selective photochemical syntheses of organic frameworks may be imposed depending on whether molecular light filters are present or not. Indeed, photochemical outcomes of divergent organic reactions may be controlled by subtle changes in the substrate, reaction conditions or the use of sensitizers.^7^ Recently, we employed UV light absorbing organic molecules in a selective deprotection of photolabile protecting groups and photo-induced cross-metathesis.^8^ Moreover, the selective use of light of different frequencies to promote distinct chemical reactions, *i.e.* chromatic orthogonality,^9^ has been applied in synthetic procedures, including the synthesis of a natural peptide product.^10^

**Fig. 1:**
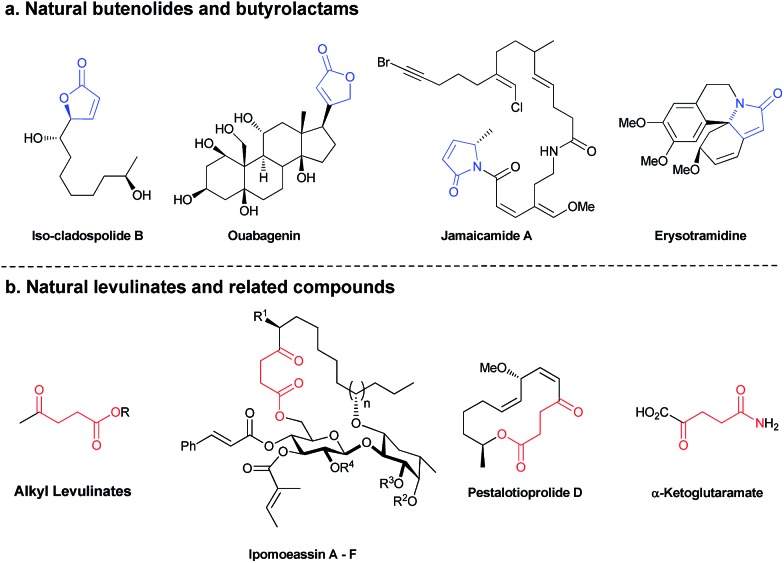
Selected examples of natural products containing butenolides and levulinates.

During our studies on chromatically orthogonal metathesis reactions using photo-switchable catalysts,^11^ the consecutive irradiation at 350 nm and 254 nm UV light of a solution of α-vinylbenzyl alcohol and methyl acrylate resulted in the nonselective synthesis of two products, a butenolide and a levulinate.^12^ Levulinates^13^ and butenolides^14^ are exceptionally ubiquitous, and some prominent natural products and derivatives containing these motifs are highlighted in Fig. 1.^15^ Indeed, the development of efficient synthetic routes for their preparation continues to be an active area of interest to synthetic chemists and many methodologies have been put forth recently.^13^,15a,c–e,^16^ The ability to obtain different products utilizing the same starting materials and intermediates, especially by photochemical processes under mild conditions, may pose an important synthetic and industrial advantage.^17^ Moreover; photochemistry possesses the inherent property of being able to be remotely controlled.^18^

**Scheme 1:**
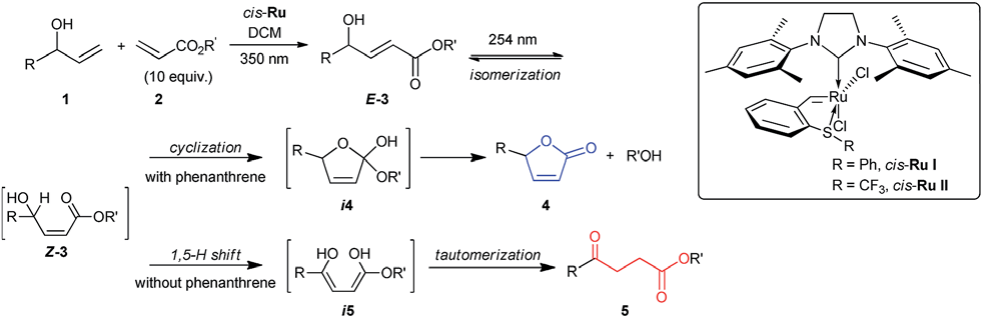
Bichromatic selective divergent reaction.

Herein, we report selective divergent all-photochemical syntheses of levulinates and butanolide derivatives using simple starting materials. Following a UV-A photoinduced cross-metathesis reaction, UV-C irradiation generates the desired products. Mechanistic and computational studies elucidate the role of phenanthrene as a UV-C modulator: the presence of phenanthrene in the reaction suppresses the 1,5-H shift pathway leading to high selectivity in the formation of butenolides, while excluding the phenanthrene allows the opposite selectivity (Scheme 1). Several examples are shown highlighting the scope and broad implications of this method, including the selective synthesis of a natural product molecule in a very efficient and convenient manner.

## Results and discussion

The first step of the desired sequence is a photoinduced CM reaction between an allylic (**1**) and an acrylic (**2**) substrate.^19^ For this, photolatent *S*-chelated ruthenium benzylidenes were used.^20^ Even though the CM reaction may also be efficiently promoted by typical ruthenium metathesis catalysts, the use of photolatent precatalysts allows for an all-photochemical method that can be both spatially and temporally controlled.^10^ Thus, UV-A induced CM between **1a** and **2a** or **2b** was readily promoted by the robust catalyst, *cis*-**Ru I** in good yields (Table 1, entries 1 and 2). Evaporation of the excess acrylate, without the need for further purification, set up the stage for the second photochemical step. In more complex CM reactions, for example when acrylates **2c–2e** or allyl alcohols **1c** and **1d** were used (Table 1, entries 3–9), *cis*-**Ru I** proved to be a less efficient CM catalyst. To our satisfaction, a faster initiating photolatent catalyst (*cis*-**Ru II**)^21^ could perform better in these difficult cases, highlighting the advantages of the versatility of the *S*-chelated family of catalysts.

**Table 1:** Sequential photochemical reactions^*a*^

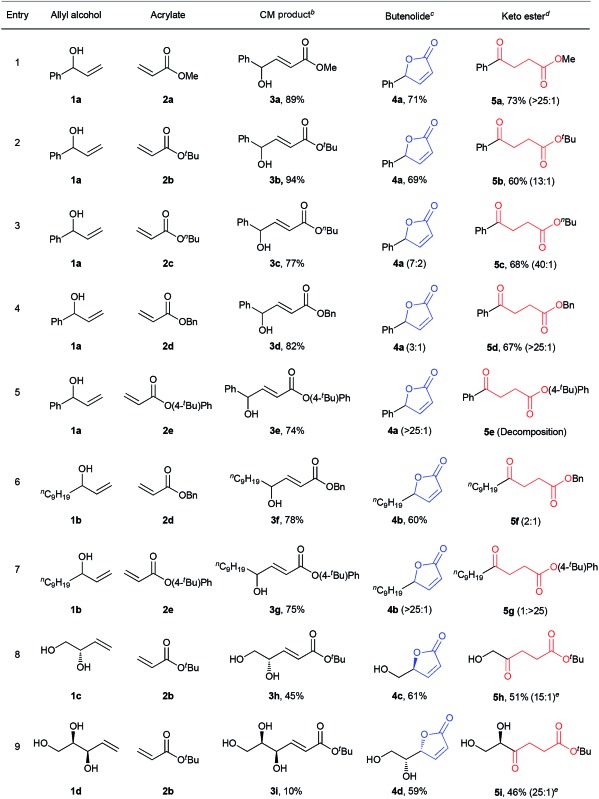

^*a*^CM was carried out at 0.05 M, unless otherwise noted. Isomerization reactions were conducted at 0.01 M concentration in DCM at 25–30 °C. 10 equiv. of acrylate was used in the CM reaction. The reported yields are isolated yields. Product ratios in parentheses are based on ^1^H NMR analyses. See ESI for further details.

^*b*^For entries 1 and 2, *cis*-**Ru I** (3 mol%) was used. For entries 3–7, 3 mol% while for entries 8 and 9, 6 mol% *cis*-**Ru II** was used.

^*c*^For entries 1–5, 2.1 equiv. and for entries 6–9, 0.3 equiv. of phenanthrene was used. The product ratios in the parentheses are **4** : **5**.

^*d*^For entries 2, 8 and 9, DCM and for entries 1 and 3–7, ^*t*^BuOH/DCM (1 : 4) was used as the solvent. The product ratios in the parentheses are **5** : **4**.

^*e*^0.1 mM aq. solution of adenosine was used as an external UV-C filter to minimize side reactions (*vide infra*).

Because ruthenium by-products are known to catalyze non-metathetic processes,^22^ the possible role of the ruthenium species in the second part of the sequence was examined. Two samples of *E*-**3a**, one made by the aforementioned CM route and the other by a metal-free route,^23^ were subjected to irradiation at 254 nm under identical conditions. Similar results were obtained in both cases; demonstrating that the conversion to **4a** and **5a** are sheer photochemical processes not assisted by ruthenium. We then surmised that the transesterification^24^ could be stifled by running the reaction at lower temperature or by the use of a bulky acrylate. Indeed, decreasing the reaction temperature by about 10 °C reduced lactonization and shifted the reaction towards the levulinate compound (see ESI for details†). Better still, higher than 90% selectivity for **5b** was achieved when bulky **2b** was used as the CM partner in step 1 (Table 1, entry 2). Next, we tested whether the addition of a bulky alcohol could help provide the necessary steric barrier to inhibit the lactonization reaction by hydrogen bonding to **3**. This could be quite practical if less sterically demanding acrylates are desired as the CM partners, especially because methyl and butyl acrylates are amongst the most important acrylic esters, with great demand as commodity chemicals. In addition, the possibility to apply different acrylates expands the scope of levulinate products that may be directly obtained by the divergent sequence. To our satisfaction, high selectivities for the 1,5-H shift were achieved when one equivalent of ^*t*^BuOH was added to the reaction solution during irradiation of *E*-**3a** with 254 nm light. Further optimization of reaction conditions showed that a mixture of ^*t*^BuOH and DCM (1 : 4) gave the best selectivity for **5a** (Table 1, entry 1). Notably, reactions with **2a** and **2b** were very convenient given that excess acrylate could be easily removed by evaporation after the CM reactions, allowing sequential reactions without purification of the intermediate.

To promote the opposite selectivity, we envisioned applying the molecular ‘UV-C filter’ protocol^8^ to prevent the photochemically more demanding 1,5-H shift (*vide infra*). Indeed, after screening several UV-C filters and reaction conditions, the irradiation of a DCM solution of *E*-**3a** with phenanthrene afforded 71% isolated yield of the butenolide (Table 1, entry 1). The amount of phenanthrene required for selective lactonization was found to be dependent on the molar absorptivity of the substrate. Thus, for allylic alcohols bearing aromatic substituents, about 2 equiv. of phenanthrene were required to achieve optimal selectivity; while substoichiometric (0.3 equiv.) amounts of phenanthrene were sufficient for the allyl alcohols with aliphatic substituents (Table 1). The type of acrylate used also had a significant effect on the selectivity of the lactonization process; thus, lactonization with *n*-butyl acrylates was somewhat more sluggish and led to lower selectivity (Table 1, entry 3), while the increased leaving group ability of 4-*t*-phenyl acrylate (Table 1, entries 5 and 7) resulted in butanolide formation even without the addition of phenanthrene (albeit in lower yields due to detrimental decomposition). The functional group tolerance and selectivity of the reaction sequence was also probed with more challenging starting materials that could, in principle, form different ring-size lactones. With this in mind, **3h** was irradiated at 254 nm in the presence of phenanthrene (Table 1, entry 8). Hydroxymethyl butyrolactone (**4c**)^24^,^25^ was selectively obtained with full retention of chirality, as expected. In the same manner, optically pure **4d** was obtained from **3i** (Table 1, entry 9). High selectivities, and decent overall isolated yields, were also obtained for the levulinates **5h** and **5i**, the only difference in the procedure being that the UV absorbing phenanthrene was not added to the reaction mixture.

To better understand this remarkable photochemical divergent process, we decided to investigate possible reaction mechanisms. It is reasonable to assume that formation of the lactone (**4a**) occurred through a typical diabatic photochemical *E*–*Z* isomerization of the olefin,^26^ followed by transesterification. Likewise, ketone **5a** may be formed through a 1,5-H shift, generating a dienol intermediate (***i*5**), which may tautomerize to afford the final product (Scheme 1).^27^

**Scheme 2:**
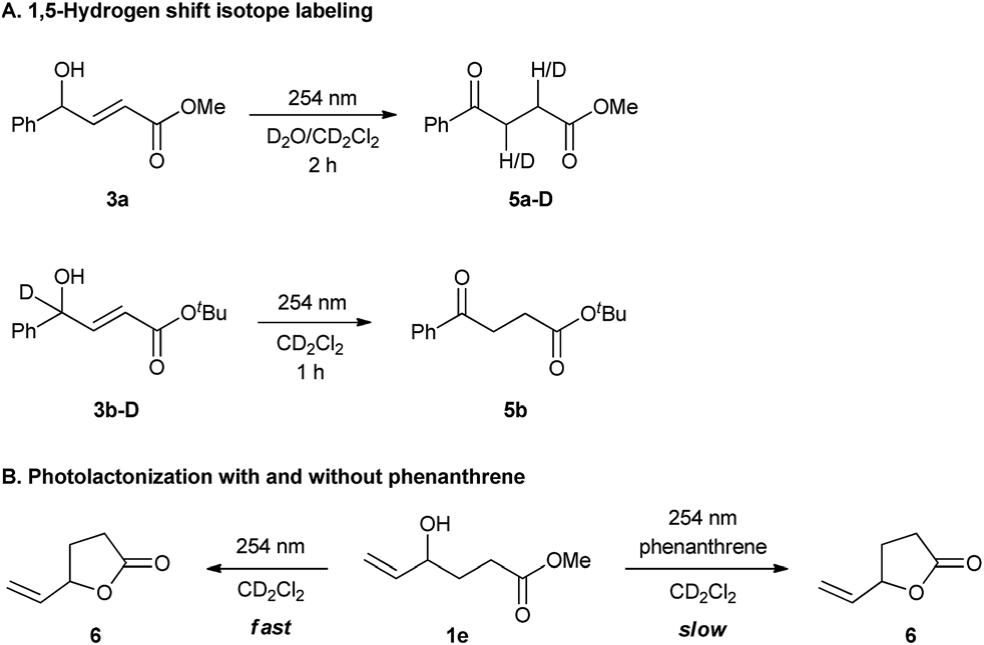
Mechanistic studies.

Supporting this hypothesis, *Z*-**3a** was appreciably observed by ^1^H NMR monitoring during UV-C irradiation of *E*-**3a**. Wavelengths of 350 nm, 380 nm or even thermal stimuli, *e.g.* heating to 140 °C, did not lead to isomerization; however, upon irradiation with 300 nm light some *Z* compound was observed (see ESI for details†). The generation of the dienol intermediate (***i*5**) was validated by deuterium labelling studies (Scheme 2A). The photochemical reaction in the presence of D_2_O/CD_2_Cl_2_ showed the incorporation of deuterium at both the α- and β-positions in product **5a-D**. Also, UV-C irradiation of deuterium labelled intermediate **3b-D** afforded a product without deuterium (Scheme 2A). As a further control experiment, a reaction of the corresponding TMS-protected *E*-**3a** was performed in CD_2_Cl_2_, forming the expected TMS-enolate intermediate (see ESI for details†). These experiments strongly support a 1,5-hydrogen shift mechanism to produce the dienol intermediate (***i*5**) and refute the occurrence of a plausible photochemically induced 1,3-hydrogen shift. The effect of phenanthrene on cyclization was probed by UV-C irradiation of **1e**, a substrate that can only cyclize (Scheme 2B) both with and without phenanthrene. In both cases cyclization to the lactone was observed; however, the reaction was slower when phenanthrene was present. This experiment clearly suggests that the phenanthrene is not sensitizing the cyclization reaction, but is indeed inhibiting the 1,5-H shift.

Quantum chemical calculations were used to further study the reaction mechanisms involving these processes. The brightest excited state was found to be S_2_ for *E*-**3j** (Scheme 1, R = R′ = Me) with 189 nm and S_3_ for *Z*-**3j** with 192 nm. The excitation energies of the *E* and *Z* isomers are very close (Δ*E* = 0.09 eV), and therefore both can be excited by irradiation of UV light of the same wavelength. The S_9_ state of phenanthrene lies at 193 nm (Table S5†), overlapping with the main absorption of both isomers of **3j**. It is therefore expected that addition of phenanthrene should decrease the rate of the *E*–*Z* interconversion. As well noted in the literature,^28^ the computed vertical excitation energies are shifted from the experimentally observed absorption maxima due to lack of solvent effects, vibrational effects and accuracy of the quantum chemical methods. Hence, we need to consider the excitation wavelength of 254 nm to be blue shifted in the theoretical context. To assess the effect of the solvent on the vertical excitation energies, quantum chemical calculations were carried out using an implicit solvent model (see Table S5†). In solution phase the spectra show a red shift in the range of 2 to 16 nm. Hence, inclusion of the solvent model shifts the vertical excitation energies towards the experimental values. In order to rationalize the effect of phenanthrene on the photochemical reactions, intermediates of the two divergent reaction pathways were analyzed theoretically. The intermediate for the levulinate formation pathway is a dienol (Scheme 1, ***i*5j**), and the intermediate for the cyclization pathway is ***i*4e**. Excited state energies reveal the brightest state (S_2_) of dienol intermediate ***i*5j** at 237 nm which is nearly coinciding with the wavelength of a high-lying excited state S_4_ of the phenanthrene (240 nm). Although both states are around 14–17 nm (0.29–0.35 eV) away from the experimental excitation wavelength (energy), considering (i) the blue shift of the computed vertical excitation energies with respect to the experimental absorption maxima, (ii) the broadness of the excitation source (*ca.* 10 nm) and (iii) the deformation of the geometry due to thermal energy, the excitation in this energy range is feasible. This is supported by the experimental finding that also excitation at 300 nm leads to levulinate formation. In case of the cyclization reaction, ***i*4e** (S_4_) is found to absorb at 155 nm which overlaps with the absorption of phenanthrene at 153 nm (S_31_). However, the energy difference to the excitation source is significantly higher considering the blue-shifted calculated energies (at least 1.5 eV compared to **3j**). In addition, the cyclic geometry is more rigid than that of the dienol. The higher rigidity would lead to smaller distortion from the minimum structure and a more narrow absorption band. Hence, the probability of light activation of cyclization reaction is negligible. Therefore, we conclude that the major role of the phenanthrene is to filter the wavelength that triggers 1,5-H shift and to suppress the pathway of levulinate formation while the cyclization is expected to proceed largely unaffected.

The scope of the sequential chromatic divergent process was scrutinized with several other substituted allyl alcohols and the results are displayed in Table 2. Both aromatic and aliphatic substrates underwent the expected photochemical transformations resulting in moderate to good isolated yields of the corresponding products. Notably, aryl bromides (**3l** and **3m**) underwent radical dehalogenation^29^ during the isomerization to ketone (to afford **5a**), while the aryl chloride was stable under these conditions (**3n**). However, in the presence of phenanthrene, the radical dehalogenation is avoided, yielding the corresponding bromoaryl lactones (**4f** and **4g**); and underlining the versatility of the UV-C filter method in yet another photochemical reaction. CM of **2a** with methyl 4-hydroxyhex-5-enoate (**1e**) gave rise to a diester (**3q**) which in principle could form two different cyclic esters in the presence of phenanthrene: the saturated or unsaturated lactone. Interestingly, compound **4k** was the major product when phenanthrene was added, verifying that the *trans*–*cis* isomerization process is faster than simple cyclization. On the other hand, when **3q** was irradiated in the absence of phenanthrene (with added ^*t*^BuOH) the symmetric ketodiester **5o** was exclusively produced. Unfortunately, tertiary allyl alcohol CM products (**3r** and **3s**) did not isomerize to the ketoester (no alkyl shift) by UV-C irradiation, but did follow the lactone pathway when protected by phenanthrene (**4l**, **4m**).

**Table 2:** Reaction scope with allylic alcohols^*a*^

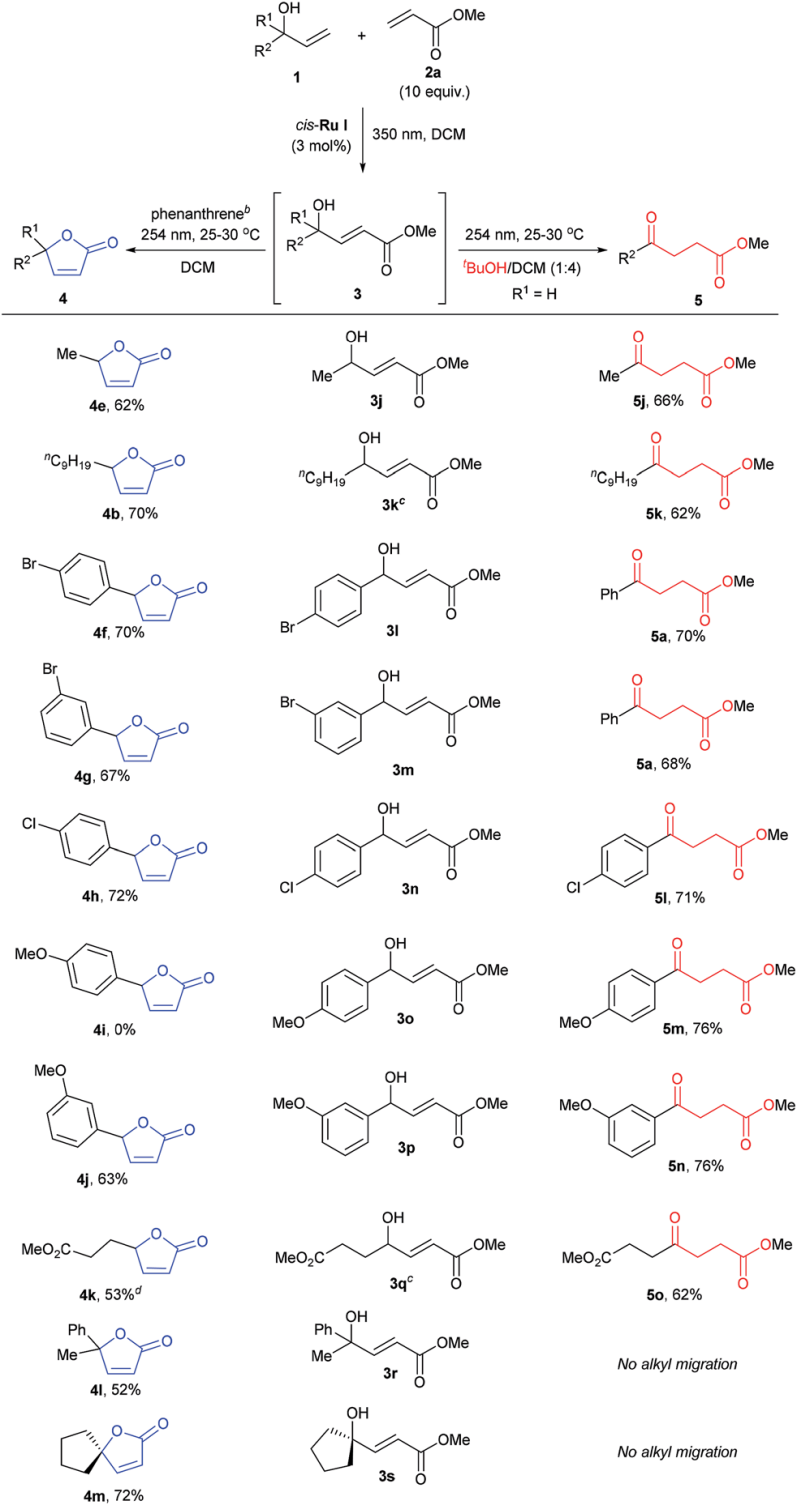

^*a*^Reported yields are isolated yields after purification and for the reaction times, see ESI.

^*b*^For **3j**, **3k**, **3q** and **3s**, 0.3 equiv., for **3l–3p**, 2.1 equiv., and for **3r**, 2.3 equiv. of phenanthrene was used as internal UV-C filter during lactonization.

^*c*^ *cis*-**Ru II** (3 mol%) was used for metathesis reaction.

^*d*^Around 21% of the saturated lactone was obtained.

**Scheme 3:**
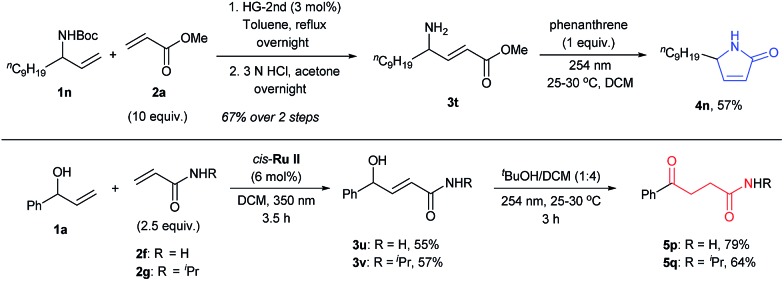
Photochemical synthesis of N-containing compounds.

Further prospects for this selective photochemical transformation were investigated by probing the reaction sequence on amines and amides. Due to the known susceptibility of metathesis catalysts for amine containing substrates,^30^*N*-Boc protected allyl amine (**1n**) was used as the CM partner with methyl acrylate (**2a**), which upon *N*-Boc-deprotection and irradiation with 254 nm light in the presence of phenanthrene, afforded the desired lactam **4n**.^31^ On the other hand, γ-ketoamides (**5p** and **5q**) were obtained in decent yields by the irradiation of CM products of α-vinyl benzyl alcohol (**1a**) and acrylamides in the presence of ^*t*^BuOH, as expected for the irradiation without UV protection (Scheme 3). Moreover, the UV-C irradiation of **3t** without phenanthrene resulted in a complex mixture; highlighting the special role that phenanthrene has in these reactions.

**Scheme 4:**
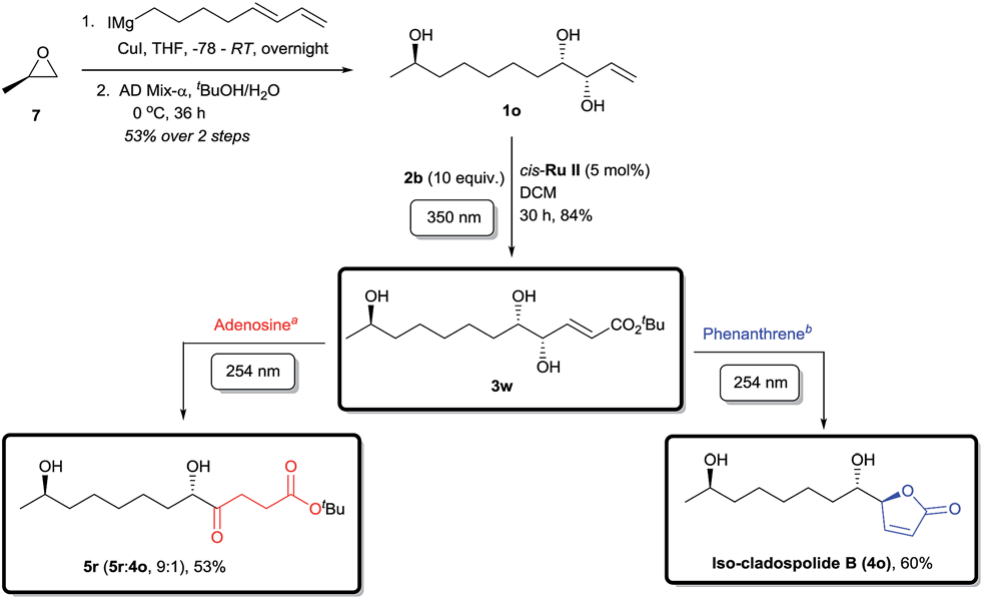
Application of a divergent photochemical sequence in total synthesis. ^*a*^A 0.1 mM aqueous solution of adenosine was used as external UV-C filter. ^*b*^0.3 equiv. of phenanthrene was used as internal UV-C filter.

Finally, the applicability of this divergent tandem photochemical process was rigorously tested in the total synthesis of a member of the anti-allergenic cladospolide family, iso-cladospolide B,15*a* and the enantiomer precursor (**5r**) of another natural product isolated from the endophytic fungal strain *Cladosporium tenuissimum* of *Maytenus hookeri*^32^ (Scheme 4). Thus, *R*-(+)-propylene oxide (**7**) was opened with a Grignard reagent prepared from 8-iodoocta-1,3-diene^33^ in the presence of CuI. The diene intermediate obtained was selectively dihydroxylated using AD-mix-α to afford the novel allyl alcohol **1o** with high enantioselectivity (see ESI for details†). Irradiation of **1o** at 350 nm wavelength with **2b** in the presence of *cis*-**Ru II**, furnished **3w** in good yields, notwithstanding the presence of three hydroxyl groups in the molecule.19a,b Due to the relative instability of the intermediates, further irradiation with 254 nm light in the absence of UV-C filter gave a more complex mixture than usual. The use of a dilute adenosine aqueous solution following the external UV-C filter protocol effectively inhibited the side reactions and CM product **3w** underwent a smooth and selective conversion to ketone **5r**, a valuable intermediate towards a biologically relevant twelve-membered macrocycle and complex levulinate containing compounds.15*e* The alternative pathway, a photochemical *tour de force*, was achieved by irradiation with 254 nm light in the presence of phenanthrene as internal UV-C filter, following the isomerization–cyclization path to give iso-cladospolide B (**4o**),15*a* a natural product isolated from marine fungi; in four protecting group free steps, the final two of which are photochemical transformations and with a total overall yield of 26%.^34^

## Conclusions

In summary, we have shown that by addition of phenanthrene as a UV regulator, commonly available starting materials may be selectively transformed into complex organic molecules by two consecutive light induced reactions. Quantum chemical calculations and experimental studies revealed important mechanistic aspects of this photochemical process, highlighting the fundamental role of phenanthrene in guiding the divergent reaction towards a selective product by hindering a 1,5-H shift. The scope of the presented sequential reactions and its limitations were exposed by the use of several allylic and acrylic substrates as CM partners to produce a wide variety of final products; including a natural product in a very efficient manner. This method presents an original divergent synthetic pathway and may inspire other types of controlled photochemistry by adaptation of this protocol.

## Conflicts of interest

There are no conflicts to declare.

## Supplementary Material

Supplementary information
