# Supplementary material for: Guiding a divergent reaction by photochemical control: bichromatic selective access to levulinates and butenolides
Source: Chem Sci. 2017 Dec 18;9(5):1368–74. doi: 10.1039/c7sc05094a (PMC5885942; doi:10.1039/c7sc05094a)
Supplement: Supplementary file 1 [file SC-009-C7SC05094A-s001.pdf]

Electronic Supplementary Material (ESI) for Chemical Science.
This journal is © The Royal Society of Chemistry 2017

Supplementary Information

Guiding a divergent reaction by photochemical control: Bichromatic selective access to levulinates and butenolides.

Revannath L. Sutar,[ab] Saumik Sen,[‡c] Or Eivgi,[‡a] Gal Segalovich,[a] Igor Schapiro,[c] Ofer Reany[b] and N. Gabriel Lemcoff*[ad]

[a] Department of Chemistry, Ben-Gurion University of the Negev, Beer-Sheva 84105, Israel.

[b] Department of Natural Sciences, The Open University of Israel, Raanana, 43537, Israel.

[c] Fritz Haber Center for Molecular Dynamics, Institute of Chemistry, The Hebrew University of Jerusalem, Jerusalem, 91904, Israel.

[d] Ilse Katz Institute for Nanoscale Science and Technology, Ben-Gurion University of the Negev, Beer-Sheva, 84105, Israel.

1. General remarks **2**

2. Synthesis of substrates **2**

3. Optimization and mechanistic studies of divergent photoisomerization **6**

4. General procedure for tandem divergent CM-photoisomerization **23**

5. Characterization data of products from tandem divergent CM-photoisomerization **24**

6. Characterization data of products from sequential divergent CM-photoisomerization **33**

7. Synthesis of iso-cladospolide B (4o) and *tert*-butyl (5*S*,11*R*)-5,11-dihydroxy-4-oxododecanoate (5r) **42**

8. Synthesis of (±)-1o **45**

9. NMR spectra of isolated products **47**

10. Chiral GC spectra **94**

11. Computational methods **100**

12. References **104**

1. General remarks

All commercially available solvents and reagents were of reagent grade and used without further purification unless otherwise stated. When stated, solvents were either dried by passage through MBraun solvent purification system or dried and purified according to the literature procedures. NMR spectra were recorded on Bruker DPX 400 or DPX 500 instruments. Chemical shifts, given in ppm, are relative to the residual solvent peak. Signal patterns are indicated as s- singlet; d- doublet; t- triplet; q- quartet, m- multiplet; br- broaden peak. Gas chromatography data was obtained using an Agilent 6850 GC equipped with an Agilent 5973 MSD working under standard conditions and an Agilent HP5-MS column, while chiral compounds were analyzed for enantiomeric excess using Rt-βDEXsm column. Optical rotations were measured using ADP410 polarimeter at λ = 589 nm and sample concentration "*c*" expressed in g/100 ml. A Thermoscientific LTQU XL Orbitrap equipped with APCI was used for high resolution mass spectrometry. Purification by column chromatography was performed on Fluka silica gel (35-75 μm). TLC analyses were performed using Merck pre-coated silica gel (0.2 mm) aluminum sheets. UV-VIS measurements were performed on a JASCO V-550 UV-VIS spectrophotometer. Irradiation experiments were carried out in quartz 5 mm NMR tube using RM 500 merry go-round unit or in fused quartz tube (15 mm diameter) under magnetic stirring inside a Rayonet RPR-200 instrument.

2. Synthesis of substrates

α-Substituted allyl alcohol **1d**[1] was prepared from D-(-)-tartaric acid while **1b**,[2a] **1g-k**,[2b] **1l**[2c] and **1m**[2d] were prepared by the reaction of vinylmagnesium bromide with corresponding carbonyl compound (aldehyde/ketone) and products were distilled using Kugelrohr apparatus under vacuum. The acrylates **2d** and **2e** were prepared by using the reported procedure[2e] and were also distilled under vacuum with Kugelrohr apparatus. The CM product, ***E*-3a** was alternatively prepared by a metal free route from benzaldehyde using a literature report.[3]

2.1. 1-Phenylprop-2-en-1-*d*-1-ol (1a-D)

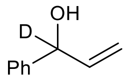

1a-D

A 25 mL oven-dried Schlenk flask was charged with a solution of benzaldehyde-α-d_1[4] (215 mg, 2 mmol) in anhydrous THF (4 mL) under nitrogen atmosphere. It was cooled to 0 °C and vinyl magnesium bromide (0.7 M in THF, 3.4 mL, 2.4 mmol) was added dropwise to it. The reaction mass was slowly brought to room temperature and the progress was monitored by TLC. After completion of the reaction (2 h), it was again cooled to 0 °C, quenched with saturated aqueous NH_4Cl solution and extracted with diethyl ether (2 × 20 mL). Combined organic extract was washed with brine (20 mL), dried over anhydrous $MgSO_4$, filtered and concentrated to get crude product. It was purified by silica gel column chromatography using ethyl acetate/hexane (1:7) as the eluent to get **1a-D**[5] (190 mg, 70%) as colorless oil and was further distilled using Kugelrohr to remove traces of solvent and moisture. R_f = 0.2 (EtOAc/hexane 1:7); (^{1}H NMR (400 MHz, $CDCl_3$) δ: 7.47 – 7.27 (m, 5H), 6.06 (dd, *J* = 16.8, 10.7 Hz, 1H), 5.36 (dd, *J* = 17.1, 0.8 Hz, 1H), 5.21 (dd, *J* = 10.3, 0.8 Hz, 1H), 1.88 (s, 1H); GC-MS (EI) for C_9H_9DO, M+ Calculated: 135.1, Found: 135.1.

2.2. Methyl 4-hydroxyhex-5-enolate (1e)

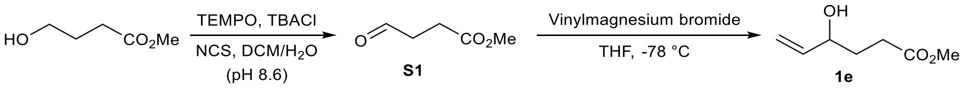

To the solution of methyl 4-hydroxybutanoate[6] (43 mmol, 3.7 g) in dichloromethane (430 mL), a solution of $NaHCO_3$ (18 g) and K_2CO_3 (6 g) in water (860 mL) was added. The biphasic solution was vigorously stirred and TEMPO (312 mg, 2 mmol), tetrabutylammonium bromide (1.4 g, 4.3 mmol) and *N*-chlorosuccinimide (7.5 g, 56 mmol) were added to it. After completion of the reaction as indicated by TLC (2 h), organic layer was separated, aqueous layer was extracted with DCM (100 mL) and combined organic extract was washed with brine (2 × 100 mL). It was dried over anhydrous magnesium sulfate, filtered and concentrated to get crude product, which was purified by silica gel column chromatography using ethyl acetate/hexane (1:9) as the eluent to get **S1** (3.5 g, 70%) as a light yellow liquid.[7] R_f = 0.2 (EtOAc/hexane 1:9); ^{1}H NMR (400 MHz, $CDCl_3$): δ 9.82 (t, *J* = 0.7 Hz, 1H), 3.70 (s, 3H), 2.83 – 2.78 (m, 2H), 2.64 (t, *J* = 6.5 Hz, 2H).

The solution of **S1** (1.16 g, 10 mmol) in anhydrous THF (20 mL) was cooled to -78 °C and 0.7 M solution of vinylmagnesium bromide (17 mL, 12 mmol) in THF was added dropwise to it. After stirring for 1 h at the same temperature, the reaction mass was gradually warmed and

when the solution reached to -40 °C, it was quenched with saturated aqueous NH_4Cl solution. It was extracted with diethyl ether (2 × 50 mL) and the combined organic extract was washed with brine (50 mL). After drying over anhydrous $MgSO_4$ and evaporation of the solvent on rotavapor, the crude product obtained was purified by silica gel column chromatography using ethyl acetate/hexane (1:5) as the eluent to get **1e** (400 mg, 28%) as a colorless liquid.[8] R_f = 0.3 (EtOAc/hexane 1:4). ^{1}H NMR (400 MHz, $CDCl_3$): δ 5.86 (ddd, *J* = 17.2, 10.4, 5.9 Hz, 1H), 5.26 (dt, *J* = 17.2, 1.4 Hz, 1H), 5.14 (dt, *J* = 10.4, 1.3 Hz, 1H), 4.21 – 4.14 (m, 1H), 3.68 (s, 3H), 2.45 (t, *J* = 7.3 Hz, 2H), 1.96 – 1.80 (m, 3H).

2.3. *tert*-Butyldodec-1-en-3-ylcarbamate (1n)

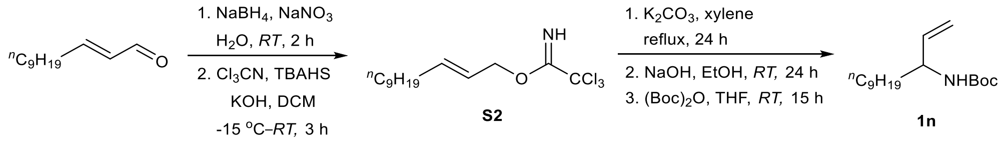

A 500 mL round bottom flask was charged with $NaBH_4$ (946 mg, 25 mmol), $NaNO_3$ (5.1 g, 60 mmol) and water (200 mL). *Trans*-2-dodecenal (4.3 mL, 20 mmol) was added dropwise to it at room temperature under vigorous stirring and the reaction was monitored by TLC. After completion of the reaction (2 h), diethyl ether (100 mL) was added and on further stirring for 10 min, organic layer was separated and washed with brine (40 mL). It was dried over anhydrous $MgSO_4$, filtered and concentrated to get crude product which was purified by silica gel column chromatography using ethyl acetate/hexane (1:9) as the eluent to yield (*E*)-dodec-2-en-1-ol (3.24 g, 88%) as colorless oil.[9] The solution of this (*E*)-dodec-2-en-1-ol (2.2 g, 12 mmol) in dichloromethane (12 mL) was cooled to -15 °C with ice salt mixture and 50 % aqueous KOH (12 mL) followed by tetrabutyl ammonium bisulfate (24 mg, 0.07 mmol) were added to it. After stirring for 5 min, trichloroacetonitrile (1.44 mL, 14.4 mmol) was added dropwise. The resulting suspension was stirred vigorously at the same temperature for 30 min, warmed to room temperature and further stirred for 3 h. Organic layer was separated and the aqueous layer was extracted with DCM (3 × 20 mL). The combined organic extract was washed with brine, dried over anhydrous $MgSO_4$, filtered and concentrated to 1/3 volume under reduced pressure. It was filtered through a 2 cm thick celite pad and washed with dichloromethane (20 mL). The filtrate was concentrated to afford product (**S2**). A small portion of it was purified by silica gel column chromatography for analysis, using ethyl acetate/hexane (1:49) as the eluent to afford a viscous oil, while rest of the product was used for the next step without further purification. R_f = 0.2

(EtOAc/hexane 1:49); ^{1}H NMR (400 MHz, $CDCl_3$) δ: 5.92 (dtt, J = 14.7, 6.8, 1.1 Hz, 1H), 5.66 – 5.58 (m, 1H), 4.78 (ddd, J = 6.7, 1.8, 0.8 Hz, 2H), 2.08 (q, J = 7.1 Hz, 2H), 1.43 – 1.36 (m, 2H), 1.34–1.23 (m, 12H), 0.88 (t, J = 6.9 Hz, 3H); ^{13}C NMR (100 MHz, $CDCl_3$) δ: 161.8, 139.6, 121.5, 70.0, 32.2, 31.9, 29.5, 29.4, 29.3, 29.1, 28.6, 22.7, 14.1, HRMS (ESI) for $C_{14}H_{24}Cl_3NO$ m/z: $[M+H]^+$ Calculated: 328.0996; Found: 328.0997; $[M+Na]^+$ Calculated: 350.0816; Found: 350.0818.

To the solution of above crude intermediate **S2** in xylene (72 mL), K_2CO_3 (0.17 mmol, 24 mg) was added and the suspension was refluxed under vigorous stirring for 24 h. Solvent was evaporated under reduced pressure and the crude product was passed through a short silica gel column using toluene/hexane (1:1) as the eluent to obtain 2,2,2-trichloro-*N*-(dodec-1-en-3-yl)acetamide (2.63 g, 67%, after two steps) as a viscous oil. R_f = 0.3 (toluene/hexane 1:7); ^{1}H NMR (400 MHz, $CDCl_3$): δ 6.51 (d, J = 8.0 Hz, 1H), 5.80 (ddd, J = 17.2, 10.4, 5.6 Hz, 1H), 5.36 – 5.06 (m, 2H), 4.49 – 4.32 (m, 1H), 1.70 – 1.60 (m, 2H), 1.40 – 1.31 (m, 4H), 1.31 – 1.18 (m, 10H), 0.87 (t, J = 6.9 Hz, 3H); ^{13}C NMR (100 MHz, $CDCl_3$): δ 161.1, 136.7, 116.0, 53.5, 34.4, 31.8, 29.7, 29.44, 29.42, 29.24, 29.21, 25.5, 22.7, 14.1; HRMS (ESI) for $C_{14}H_{24}Cl_3NO$ m/z: $[M+H]^+$ Calculated: 328.0996; Found: 328.0997; $[M+Na]^+$ Calculated: 350.0816; Found: 350.0818.

The solution of above 2,2,2-trichloro-*N*-(dodec-1-en-3-yl)acetamide (986 mg, 3 mmol) in ethanol (15 mL) was treated with 5 M aqueous NaOH (18 mL) and the mixture was stirred at room temperature for 24 h. After completion of the reaction, ethanol was evaporated on rotavapor and the aqueous residue was extracted with DCM (3 × 30 mL). It was concentrated to 1/3 volume and Boc anhydride (687 mg, 3.15 mmol) followed by triethylamine (0.44 mL, 3.15 mmol) was added to it. After stirring overnight, it was washed with brine (2 × 10 mL), organic layer was dried over $MgSO_4$, filtered and concentrated to get yellow residue. Further purification by silica gel column chromatography using EtOAc/hexane (1:16) as the eluent provided the required product (**1n**) as pale yellow oil (760 mg, 90%). R_f = 0.25 (EtOAc/hexane 1:9); ^{1}H NMR (500 MHz, $CDCl_3$) δ: 5.78 – 5.69 (m, 1H), 5.14 (dt, J = 17.2, 1.2 Hz, 1H), 5.07 (dt, J = 10.4, 1.3 Hz, 1H), 4.42 (br s, 1H), 4.07 (br s, 1H), 1.53 – 1.49 (m, 2H), 1.44 (s, 9H), 1.35 – 1.20 (m, 14H), 0.88 (t, J = 7.0 Hz, 3H); ^{13}C NMR (125 MHz, $CDCl_3$) δ: 155.3, 139.2, 114.2, 79.2, 52.8, 35.3, 31.9, 29.5, 29.4, 29.3, 28.4, 27.4, 25.7, 22.7, 14.1; HRMS (ESI) for $C_{17}H_{33}NO_2$ m/z: $[M+Na]^+$ Calculated: 306.2404; Found: 306.2398.

3. Optimization and mechanistic studies of divergent photoisomerization

Under argon atmosphere, a quartz NMR tube was charged with *E*-**3a**[3] and appropriate solvent (0.5 mL). The solution was thoroughly purged with argon and the tube was closed. It was irradiated with 254 nm light, inside the Rayonet RPR-200 using RM 500 merry go-round unit and progress of the reaction was monitored by ^{1}H NMR analysis.

Table S1. Optimization of divergent photoisomerization reaction.

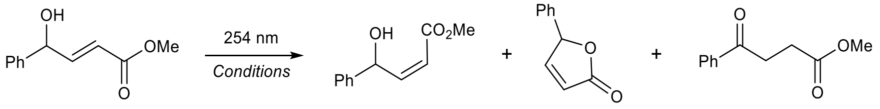

E-3a *Z*-3a 4a 5a

No	Conditions	Conversion[a] (%)	Product distribution (%)[a]		
			***Z*-3a**	**4a**	**5a**
1	0.01 M in CD_2Cl_2, ~35 ^{o}C, 2 h	quant.	Trace	40	60
2[b]	0.01 M in CD_2Cl_2, ~20 ^{o}C, 2 h	quant.	Trace	4	81
3	0.1 M in CD_2Cl_2, 6 h	76	8	33	59
4	0.01 M in $C_2D_2Cl_4$, 1 h	43	9	65	25
5	0.01 M in CD_2Cl_2, 300 nm, 17 h	91	25	4	71
6	0.01 M in CD_2Cl_2, 350 nm, 1 h	No reaction	--	--	--
7	0.01 M in CD_2Cl_2, 380 nm, 1 h	No reaction	--	--	--
8[c]	0.01 M in HFIP, 1 h	50	40	7	53
9[c]	0.01 M in ^{t}BuOH, 2 h	quant.	--	--	82
10[c]	0.01 M in ^{t}BuOH/DCM (1:4), 4.5 h	quant.	--	--	84
11	Phenanthrene (1 equiv.)[d] 0.01 M in CD_2Cl_2, 12 h	86	--	90	10
12	Pyrene (1 equiv.) 0.01 M in CD_2Cl_2, 1 h	40	24	50	26

[a] Determined by the integration of ^{1}H NMR signals. [b] During irradiation, the NMR tube was immersed in cold water in a 15 mL (15 mm wide) quartz tube. [c] Samples were analyzed after evaporation of the solvent and dissolving the residue in $CDCl_3$. [d] The equivalent of phenanthrene required for the optimal selectivity in this reaction depends on the size of reaction vessel.

Figure S1. Molar absorptivity *vs* wavelength of ***E*-3** (1×10^{-5} M) in DCM.

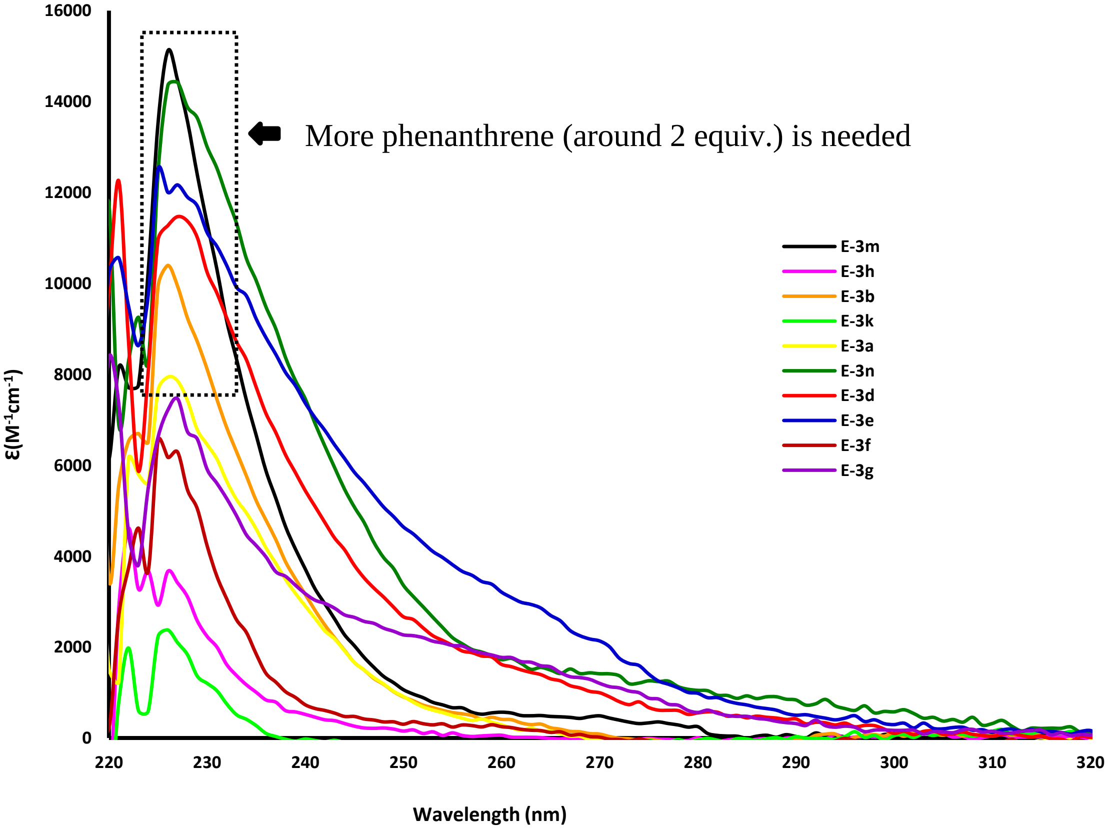

Figure S2. UV spectra of phenanthrene and pyrene (1×10^{-5} M) in DCM.

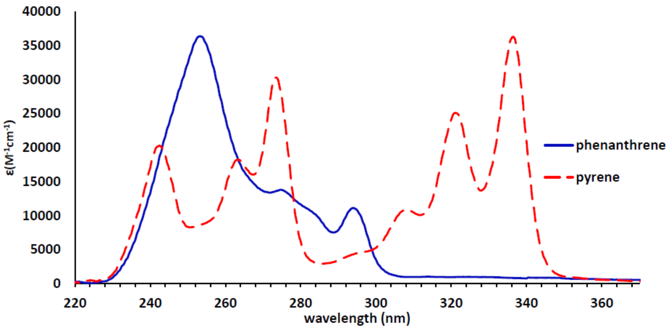

It should be noted that for substrates bearing aromatic substituents at allylic positions, the optimal amount of phenanthrene for best selectivity and time of reaction depended on the width of the reaction vessel. For quartz NMR tubes of 5 mm diameter, 1 equiv. phenanthrene was sufficient, while for the quartz tubes of 15 mm diameter, around 2 equiv. phenanthrene were needed, along with slow stirring of the reaction with magnetic needle. In addition, the reaction time is more than double for the reactions in 15 mm wide tubes. For lactonization reactions of

substrates bearing aliphatic allylic substituents, 0.3 equivalents of phenanthrene were sufficient in both cases.

Figure S3. UV absorption spectra of phenanthrene and the reaction mass of ***E*-3a** and ***E*-3k** (1×10^{-5} M) in DCM.

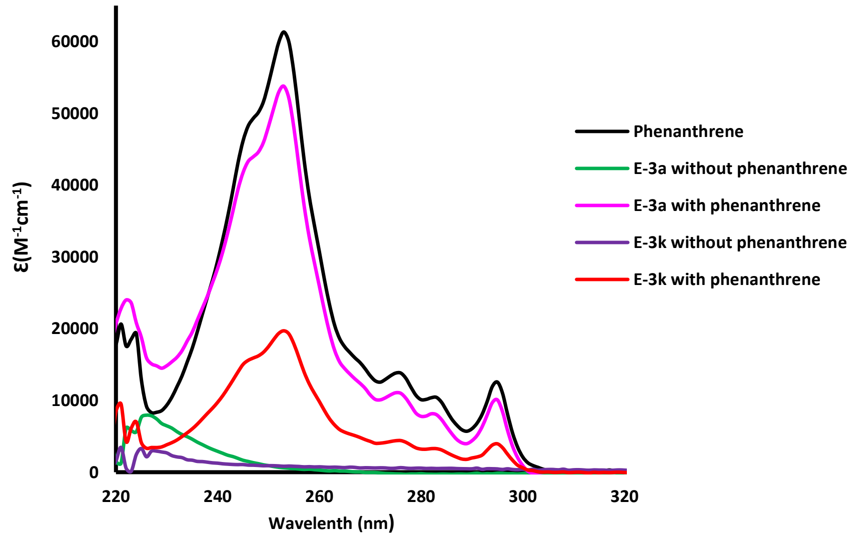

Figure S4. Fluorescence spectra (excited at 254 nm) of phenanthrene and the reaction mass of ***E*-3a** and ***E*-3k** (1×10^{-5} M) in DCM.

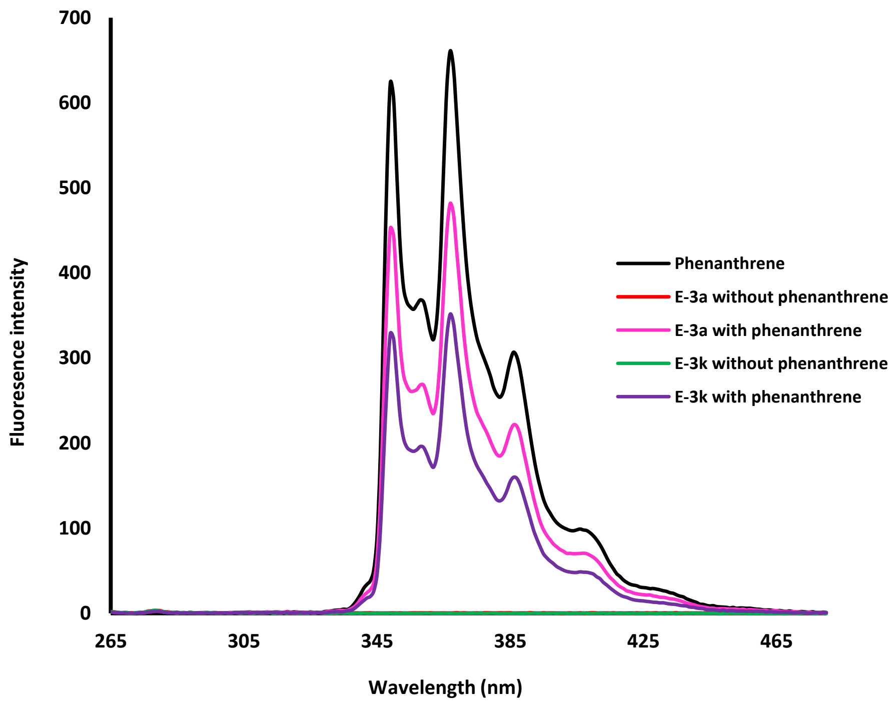

Figure S5. Fluorescence (excited at 255 nm) quenching spectra of phenanthrene (1×10^{-4} M) on addition of different concentrations of ***E*****-3a** in DCM.

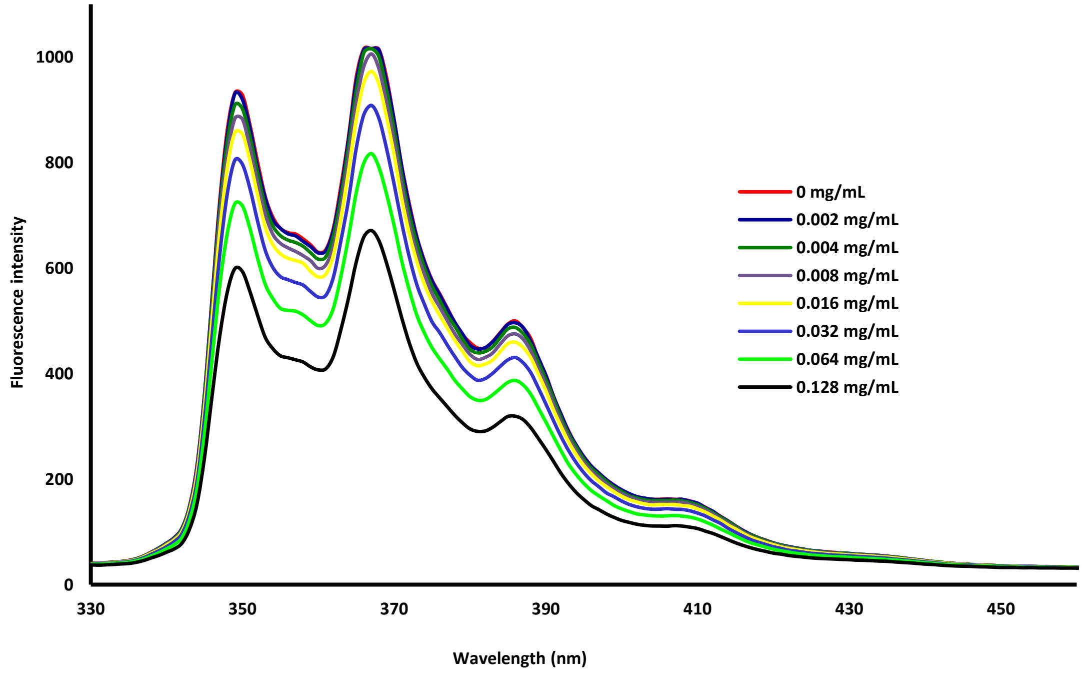

Figure S6. Stern-Volmer plot phenanthrene (F_0/F) versus concentrations of ***E*****-3a** at 386 nm.

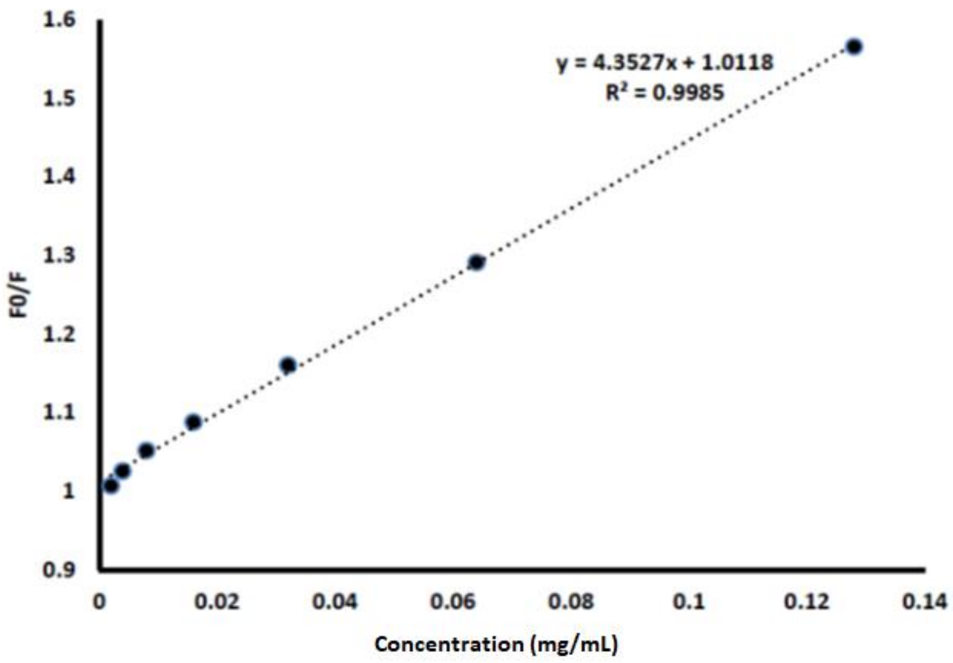

Figure S7. Fluorescence (excited at 254 nm) spectra of phenanthrene (1×10^{-4} M) on addition of different concentrations of ***E*-3k**.

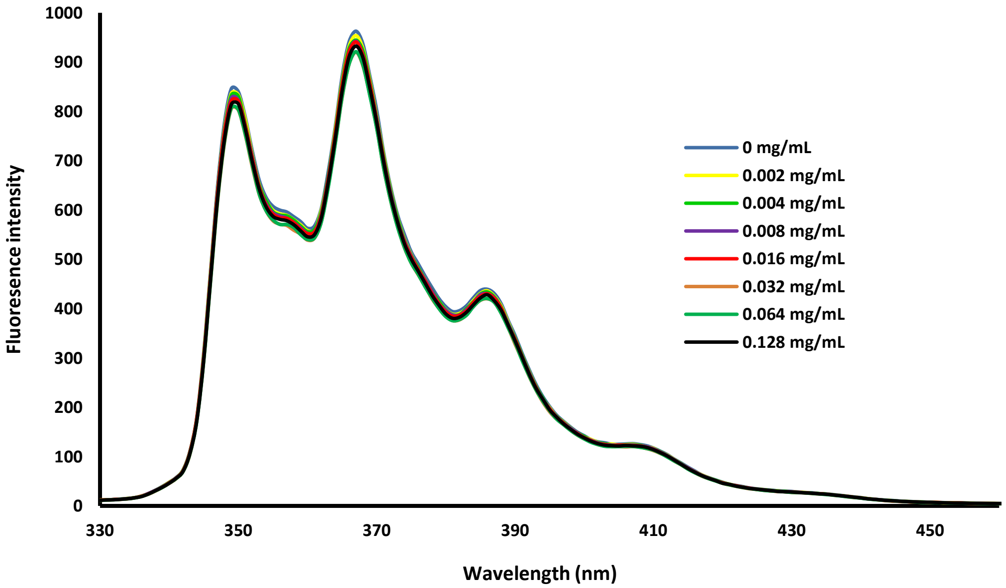

Figure S8. Isomerization of ***E*-3a** (0.01 M CD_2Cl_2) with 254 nm light at ~35 °C (^{1}H NMR, 400 MHz).

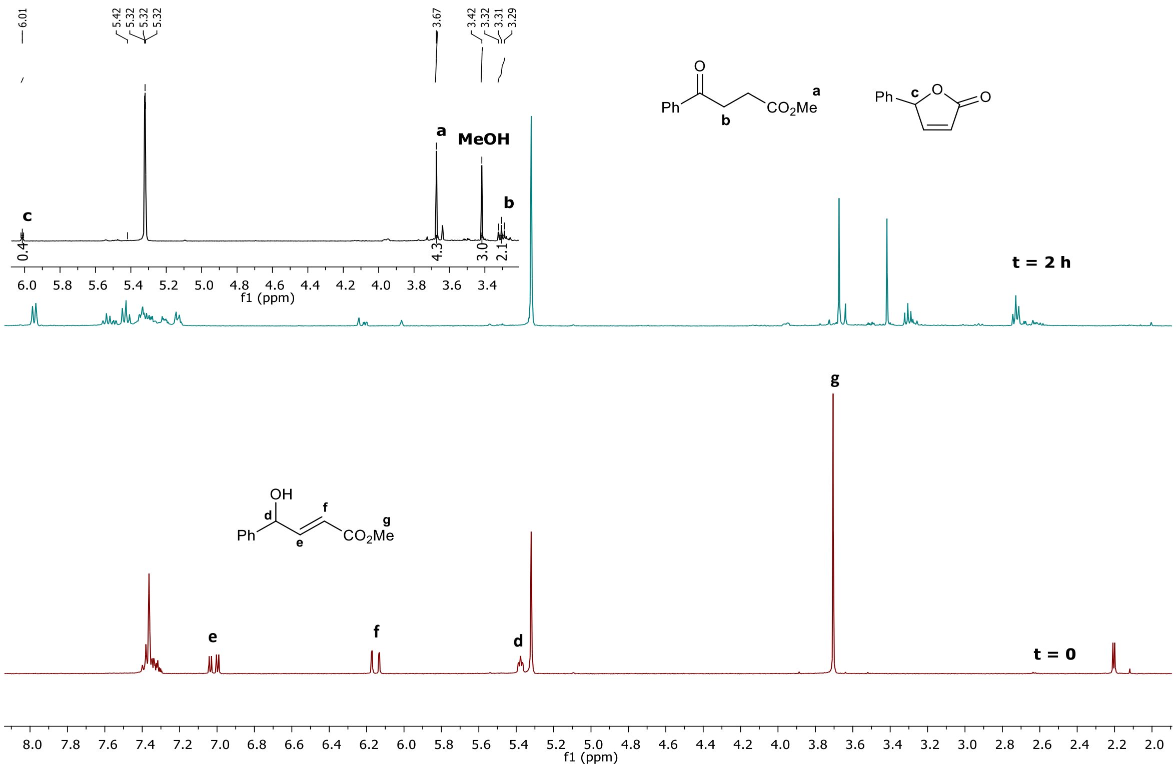

Figure S9. Isomerization of ***E*-3a** (0.01 M in CD_2Cl_2) with 254 nm light at ~20 °C (^{1}H NMR, 400 MHz).

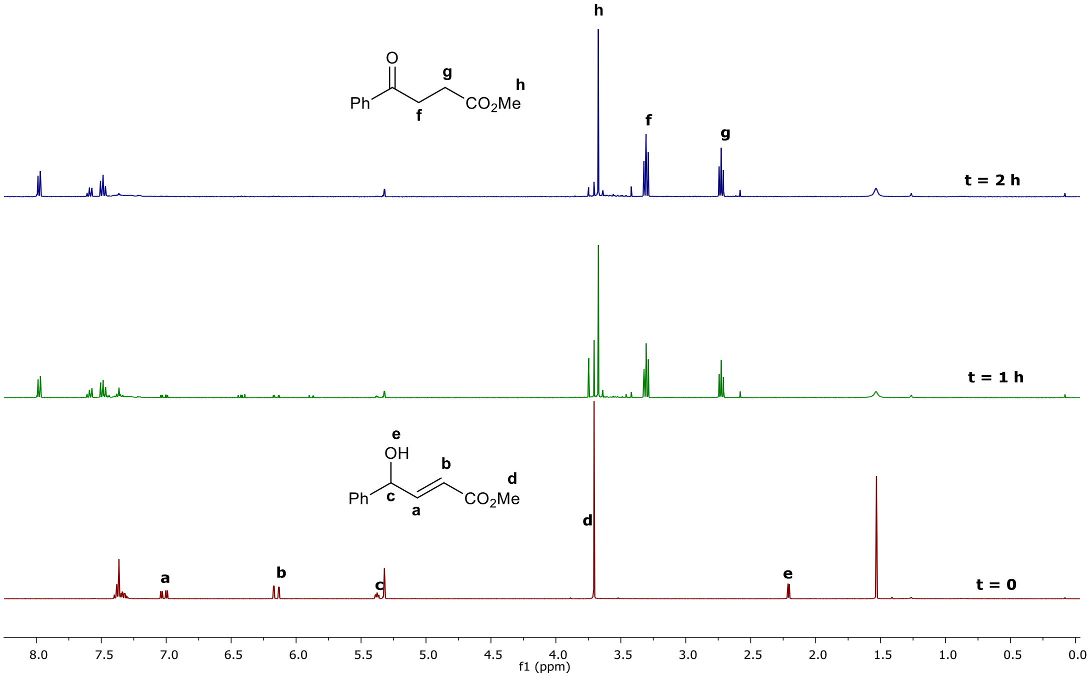

Figure S10. Isomerization of ***E*-3b** (0.01 M in CD_2Cl_2) with 254 nm light at 25-30 °C (^{1}H NMR, 400 MHz).

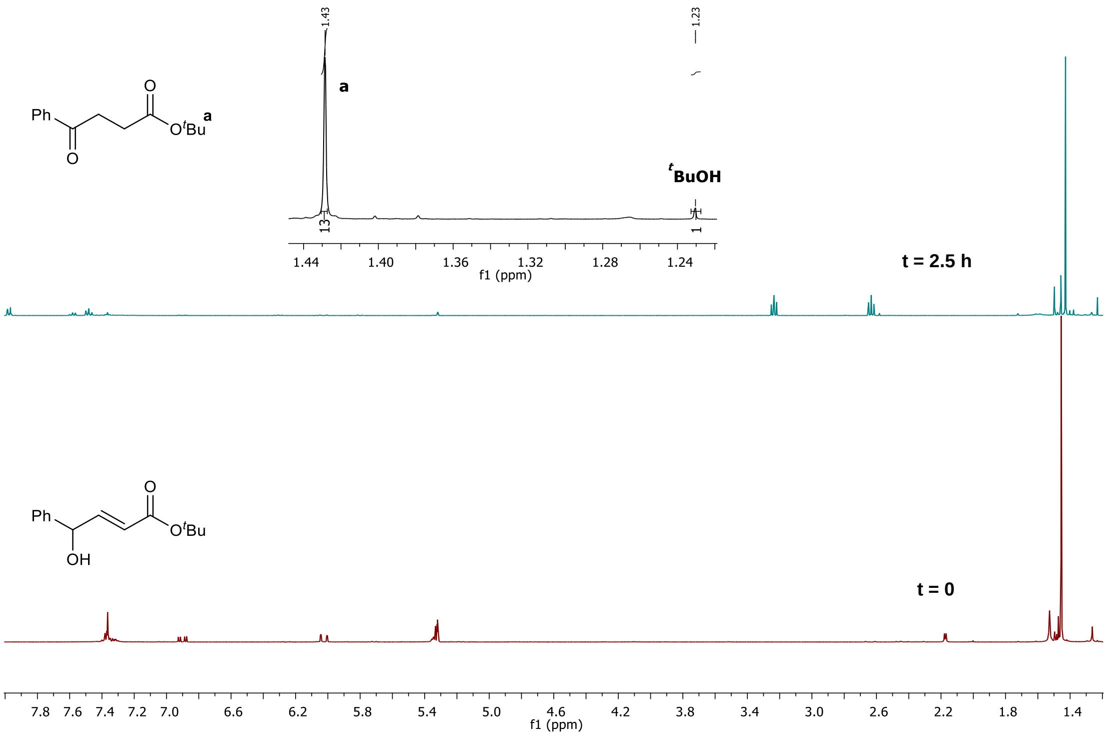

Figure S11. Selective *E*-*Z* isomerization-lactonization of ***E*-3a** (0.01 M, CD_2Cl_2) with phenanthrene (1 equiv.) on 254 nm irradiation at ~35 °C (^1H NMR, 400 MHz).

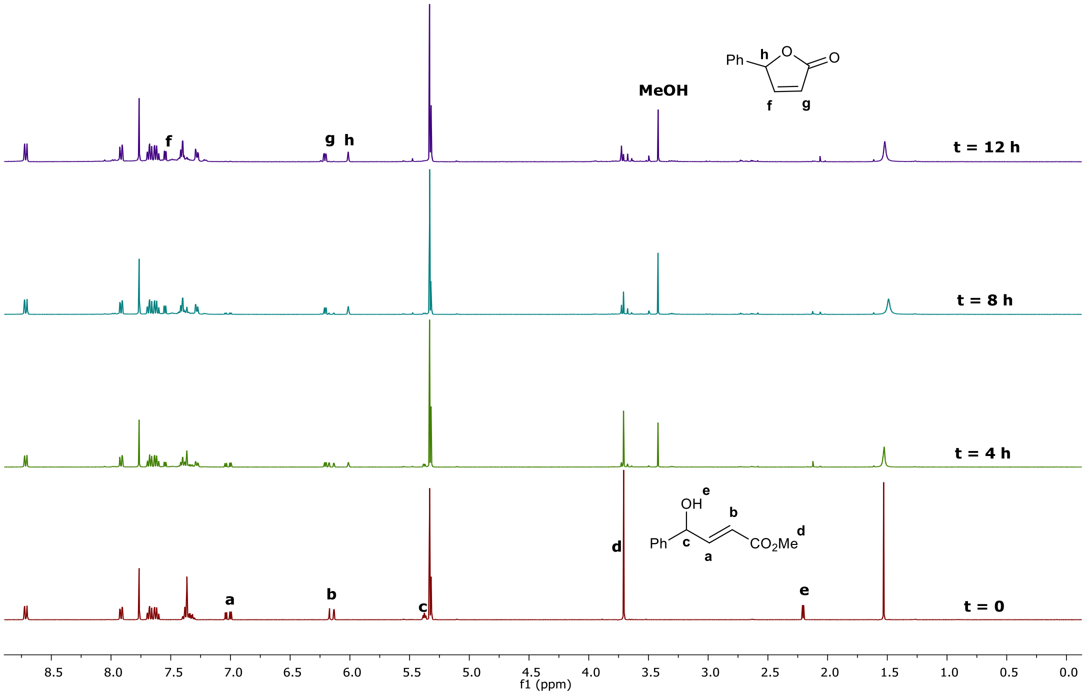

Figure S12. Selective *E*-*Z* isomerization-lactonization of ***E*-3b** (0.01 M in CD_2Cl_2) with phenanthrene (1 equiv.) on 254 nm irradiation at ~35 °C (^1H NMR, 400 MHz).

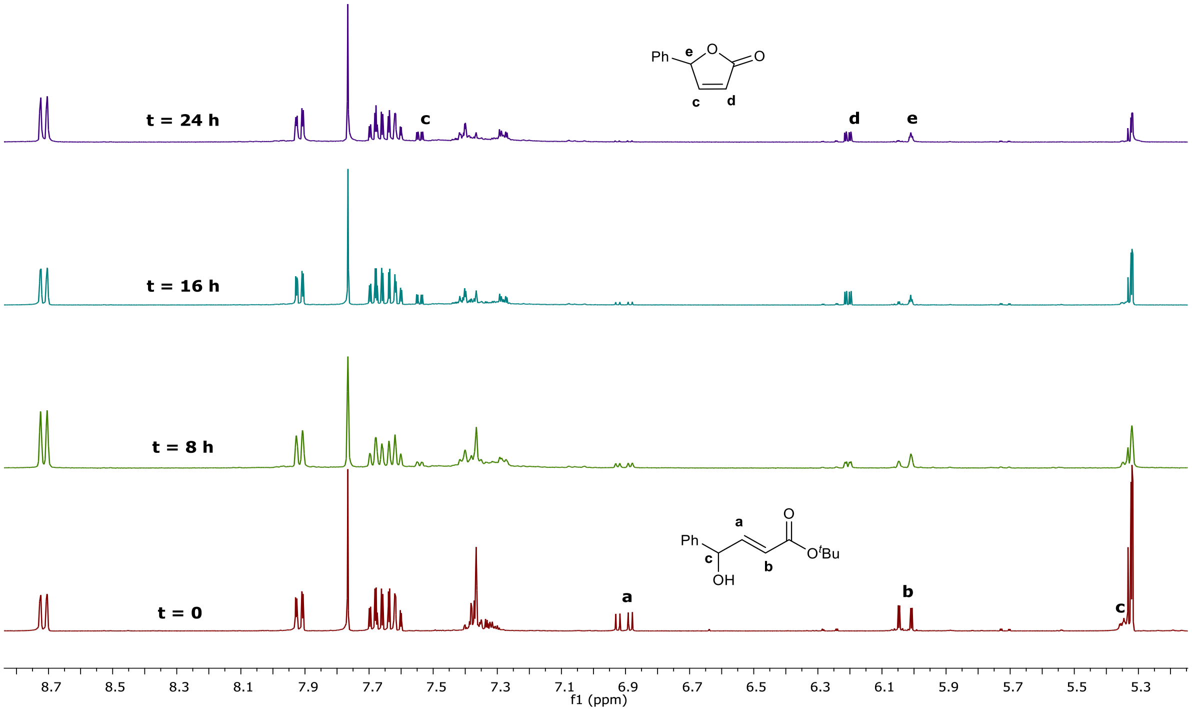

Figure S13. *E*-*Z* isomerization-lactonization of ***E*-3c** (0.01 M in CD_2Cl_2) with 254 nm light at 25-30 °C (^{1}H NMR, 400 MHz).

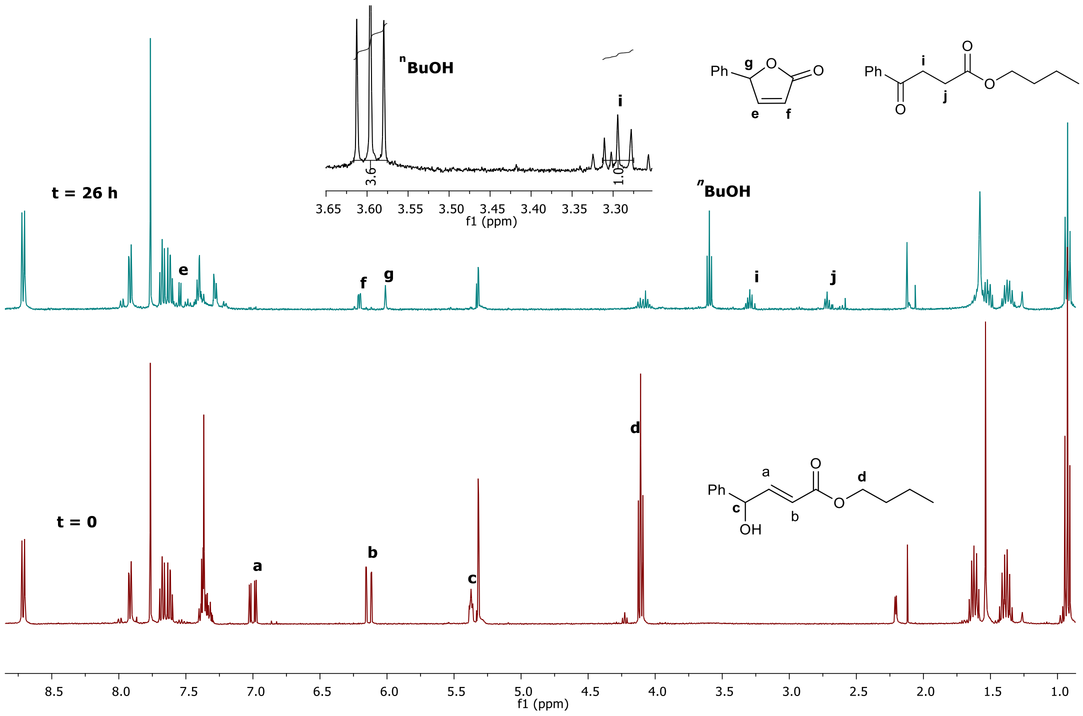

Figure S14. Isomerization of ***E*-3c** (0.01 M in CD_2Cl_2) with 254 nm light at 25-30 °C (^{1}H NMR, 400 MHz).

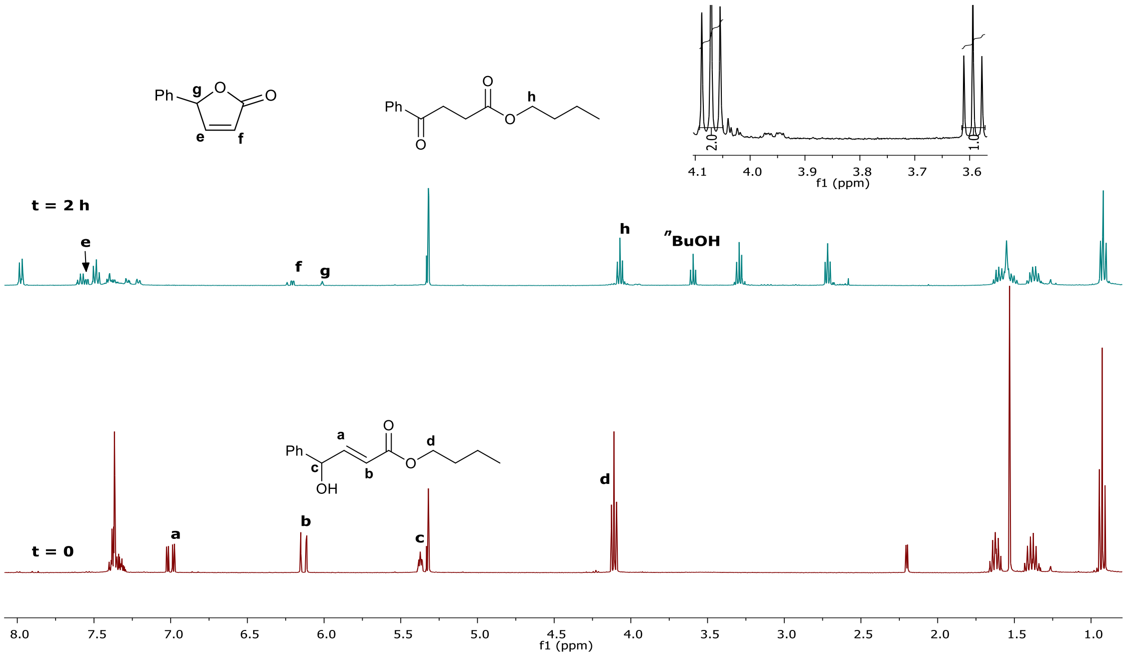

Figure S15. Isomerization of ***E*-3c** (0.01 M in ^{t}BuOH/DCM (1:4)) with 254 nm light at 25-30 °C for 2 h (^{1}H NMR, 400 MHz).

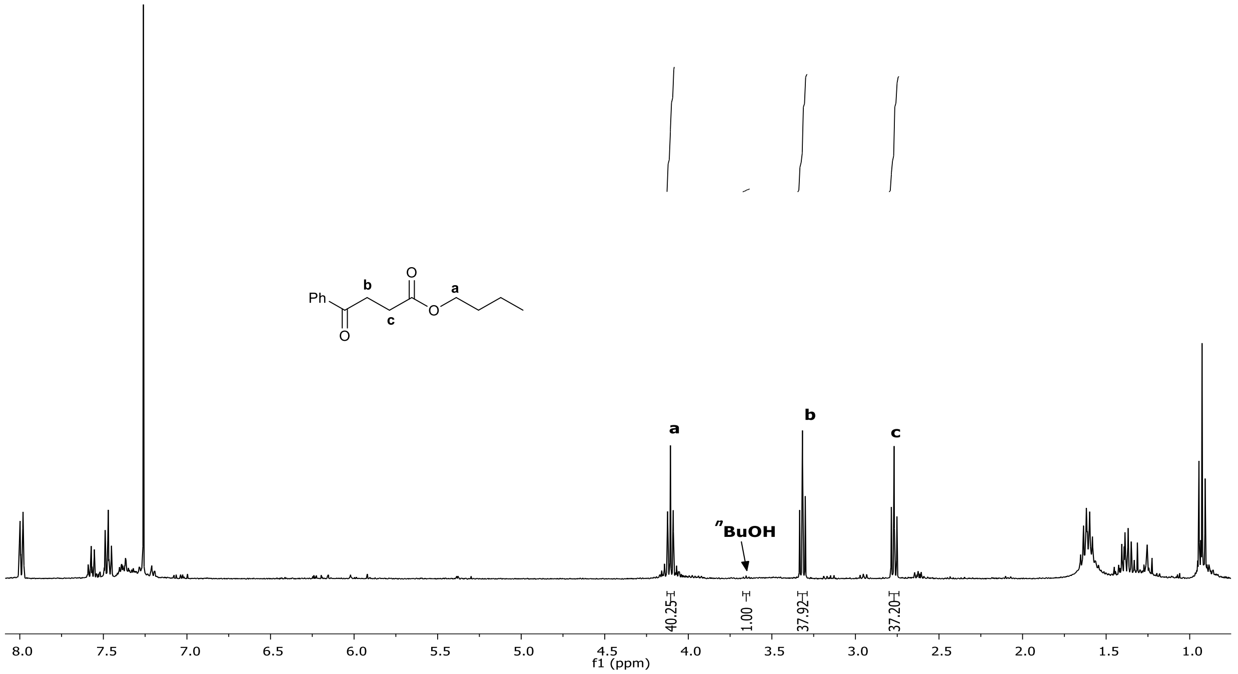

Figure S16. *E*-*Z* isomerization-lactonization of ***E*-3d** (0.01 M in CD_2Cl_2) with 254 nm light at 25-30 °C (^{1}H NMR, 400 MHz).

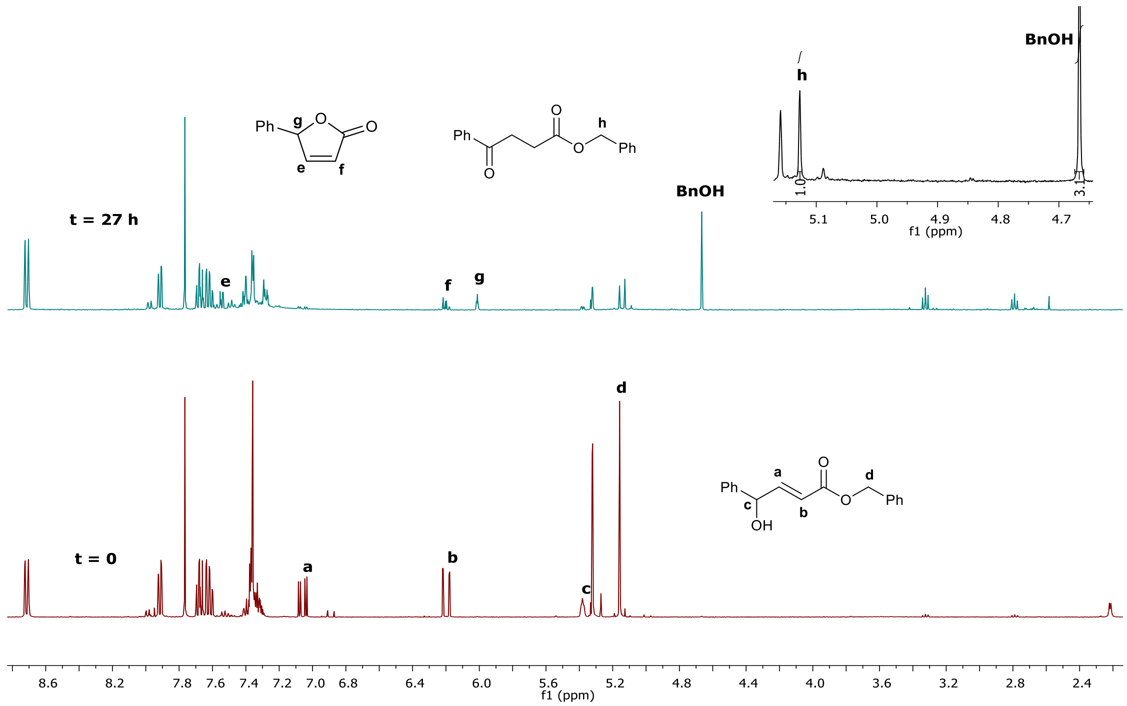

Figure S17. Isomerization of ***E*-3d** (0.01 M in ^{t}BuOH/DCM (1:4)) with 254 nm light at 25-30 °C for 2 h (^{1}H NMR, 400 MHz).

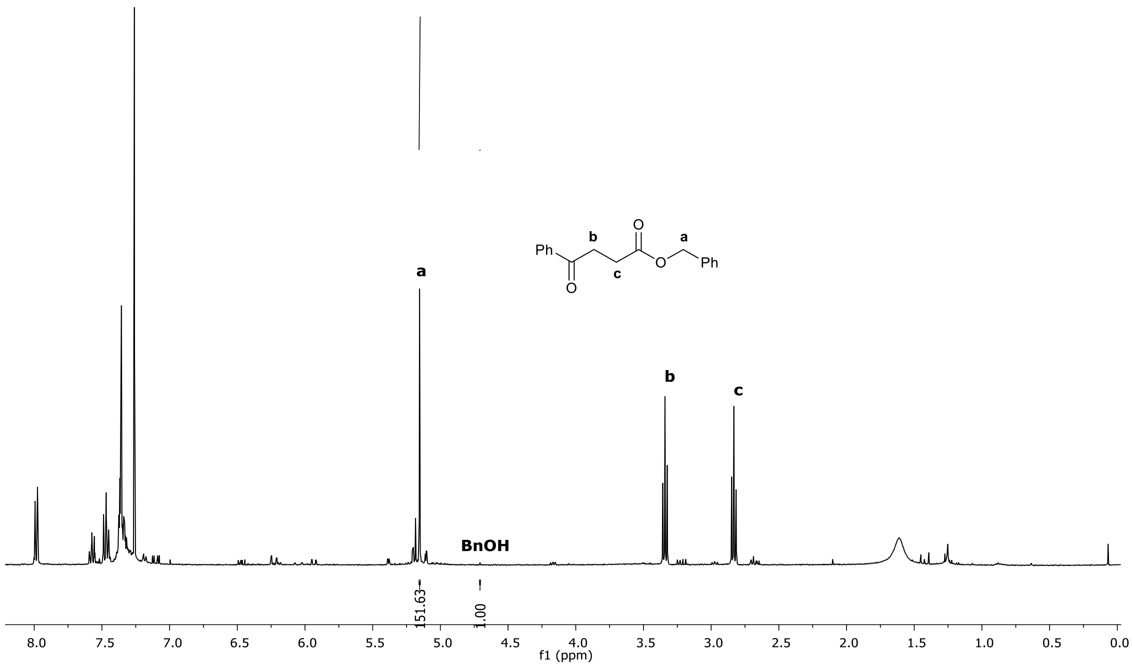

Figure S18. *E*-*Z* isomerization-lactonization of ***E*-3e** (0.01 M in CD_2Cl_2) with 254 nm light at 25-30 °C (^{1}H NMR, 400 MHz).

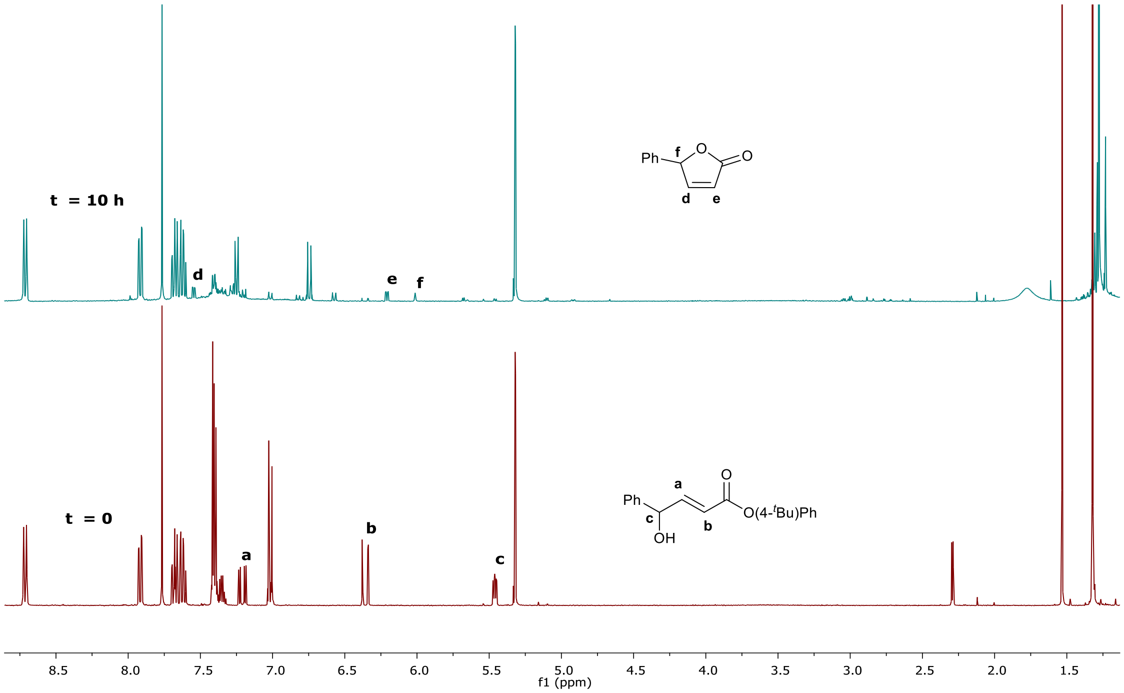

Figure S19. Isomerization of ***E*-3e** (0.01 M in ^{t}BuOH/DCM (1:4)) with 254 nm light at 25-30 °C for 2 h (^{1}H NMR, 400 MHz).

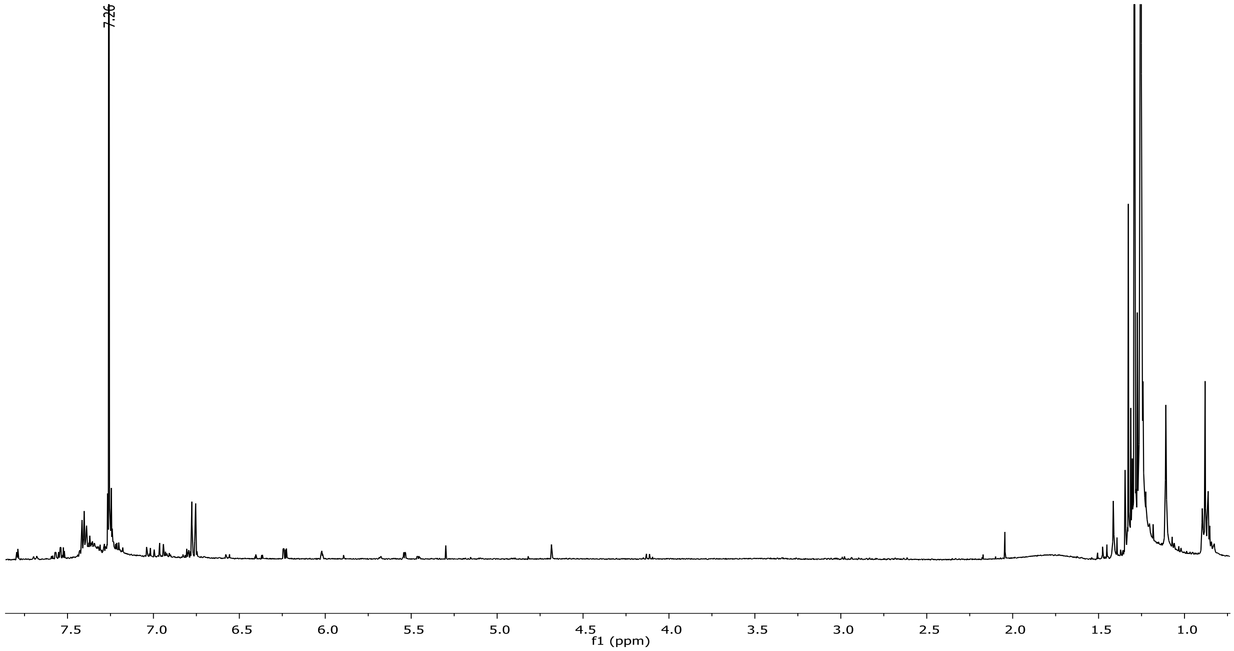

Figure S20. *E*-*Z* isomerization-lactonization of ***E*-3f** (0.01 M in CD_2Cl_2) with 254 nm light at 25-30 °C (^{1}H NMR, 400 MHz).

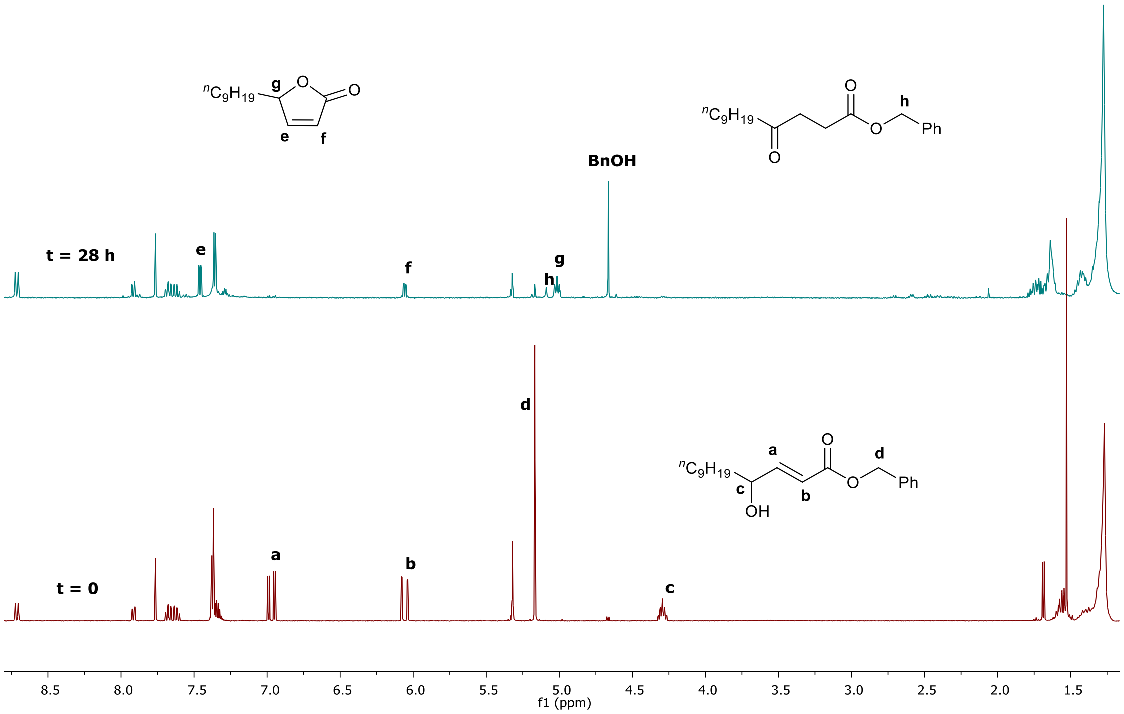

Figure S21. Isomerization of ***E*-3f** (0.01 M in ^{t}BuOH/DCM (1:4)) with 254 nm light at 25-30 °C for 2 h (^{1}H NMR, 400 MHz).

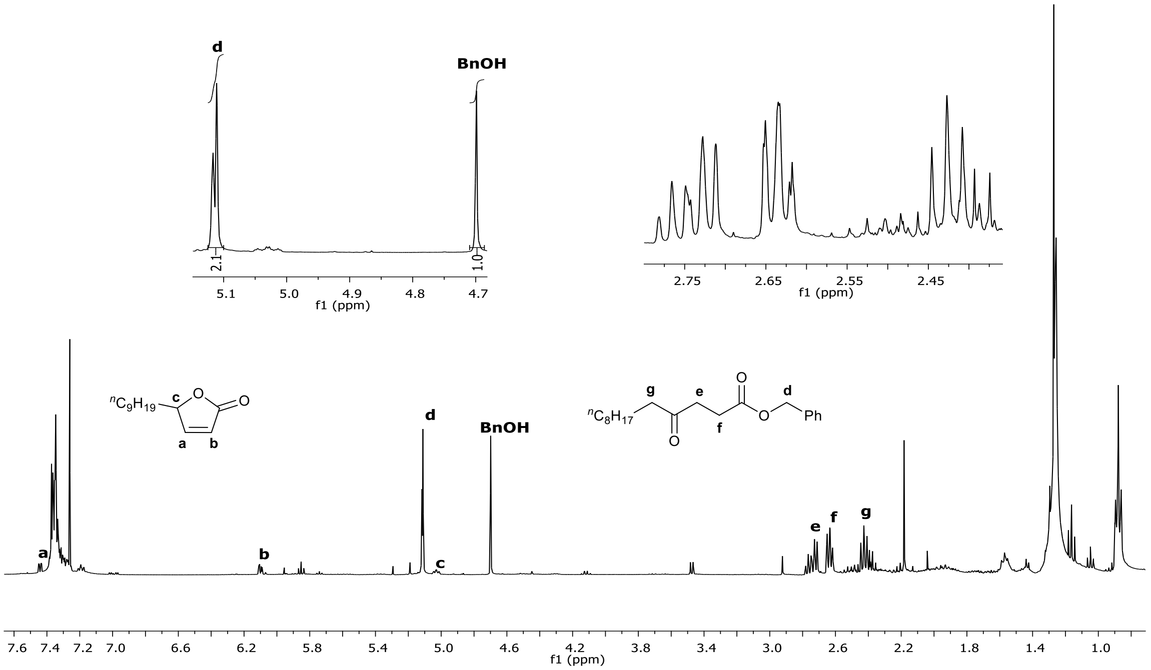

Figure S22. *E*-*Z* isomerization-lactonization of ***E*-3g** (0.01 M in CD_2Cl_2) with 254 nm light at 25-30 °C (^{1}H NMR, 400 MHz).

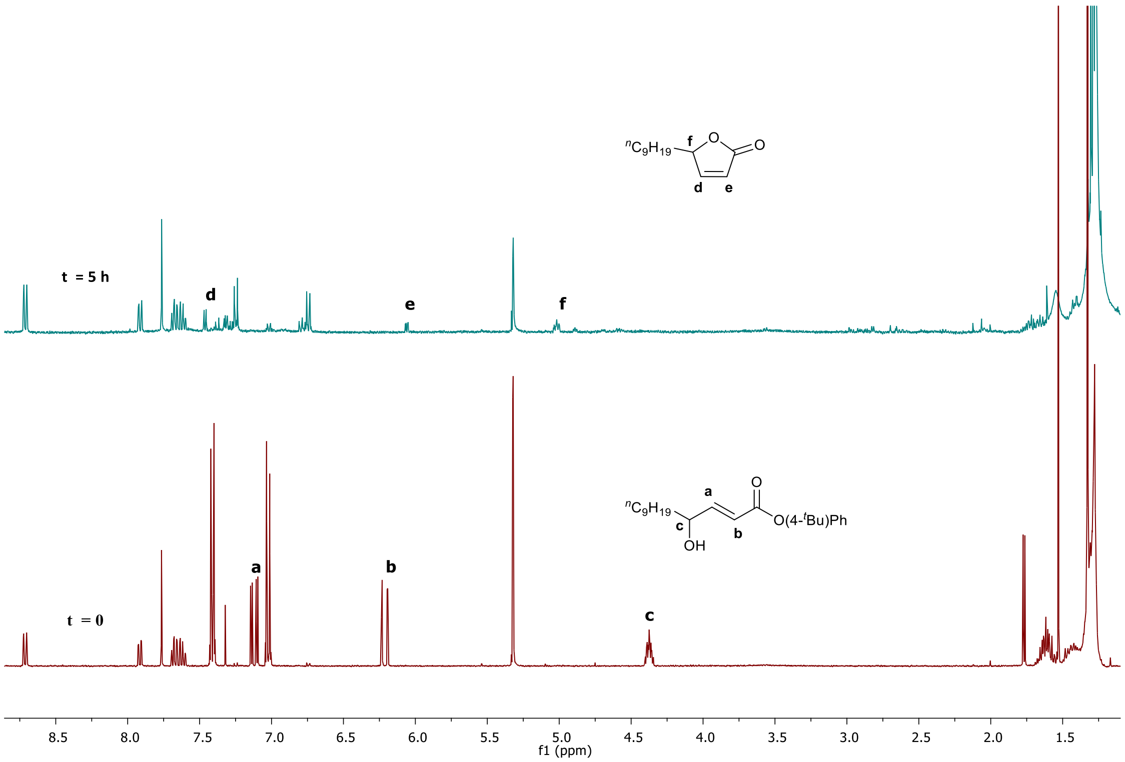

Figure S23. Isomerization of ***E*-3g** (0.01 M in ^{t}BuOH/DCM (1:4)) with 254 nm light at 25-30 °C for 2 h (^{1}H NMR, 400 MHz).

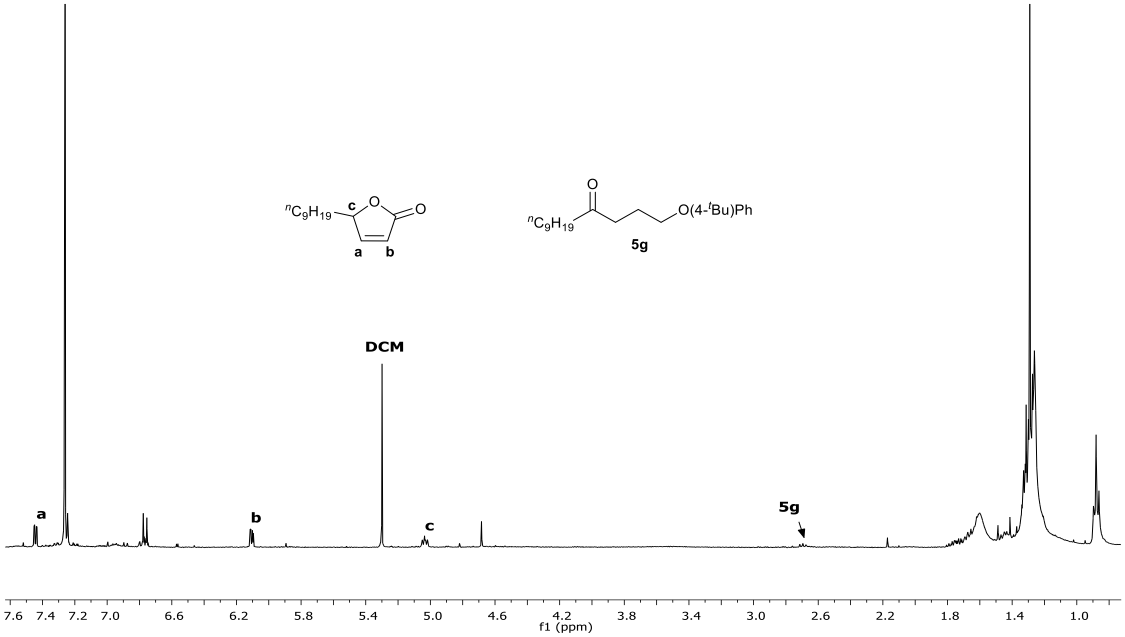

Figure S24. Isomerization of (*S*)-***E*-3h** (0.01 M in CD_2Cl_2) with 254 nm light at 25-30 °C (^{1}H NMR, 400 MHz).

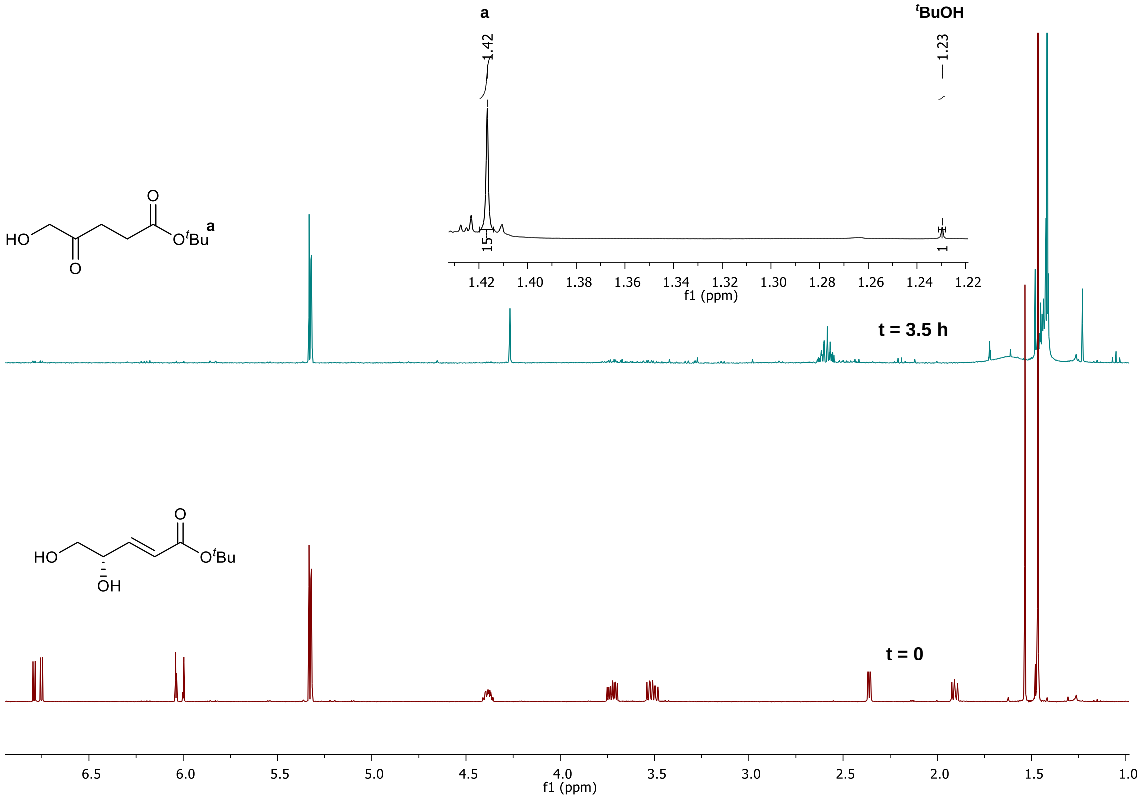

Figure S25. Isomerization of **(**4*R*,5*R*,*E***)-3i** (0.01 M in CD_2Cl_2) with 254 nm at 25-30 °C (^1H NMR, 400 MHz).

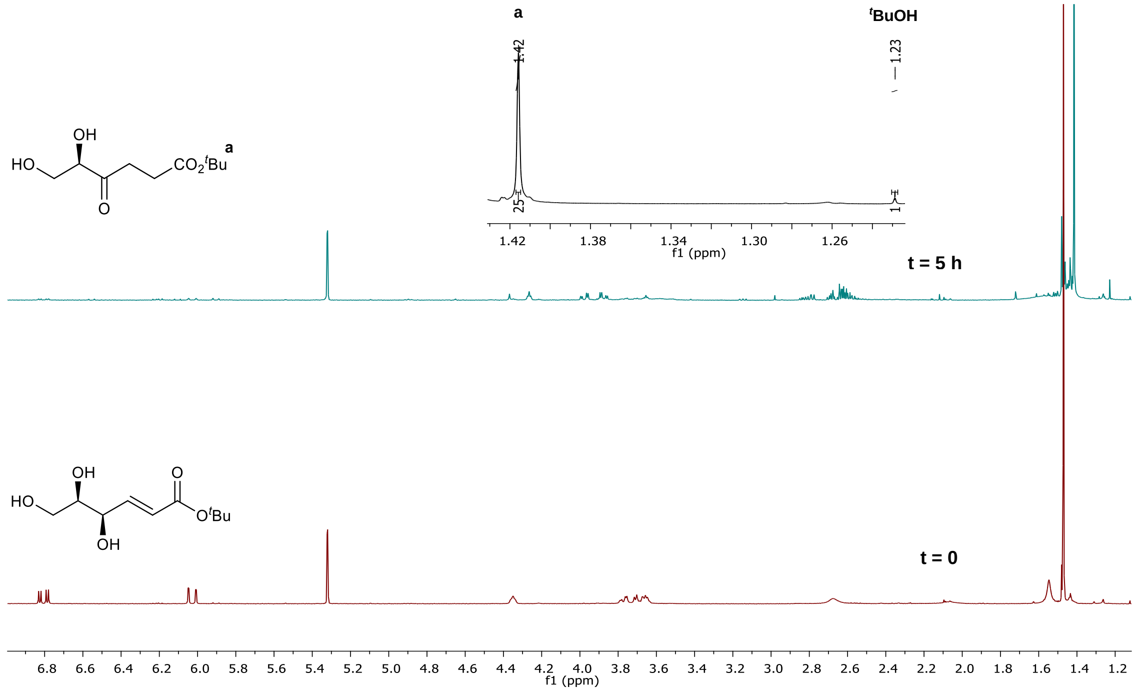

Figure S26. Isomerization of ***E*-3b-D** (0.01 M in CD_2Cl_2) by irradiation with 254 nm light at ~20 °C (^1H NMR, 400 MHz) showing no deuterium incorporation (no 1,3-D shift).

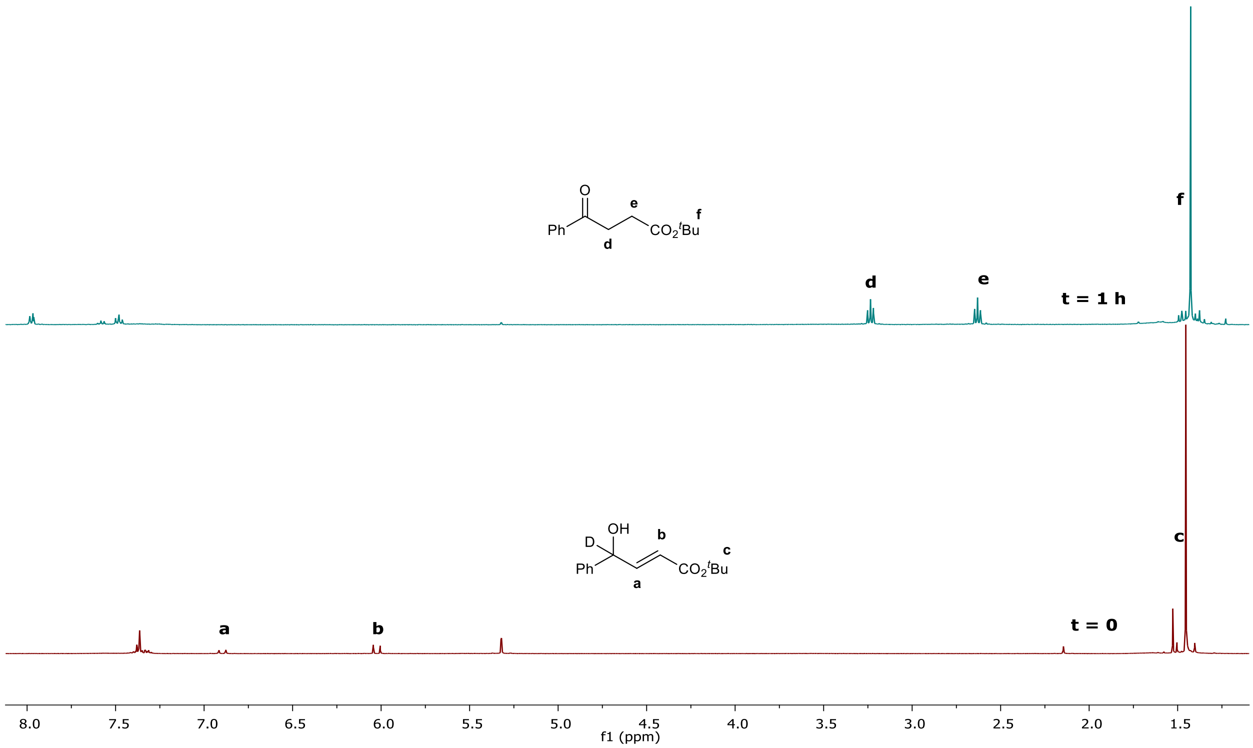

Figure S27. Isomerization of ***E*-3a** (0.01 M in CD_2Cl_2 having one drop of D_2O) by irradiation with 254 nm light at ~20 °C (^{1}H NMR, 400 MHz), showing incorporation of deuterium after tautomerization.

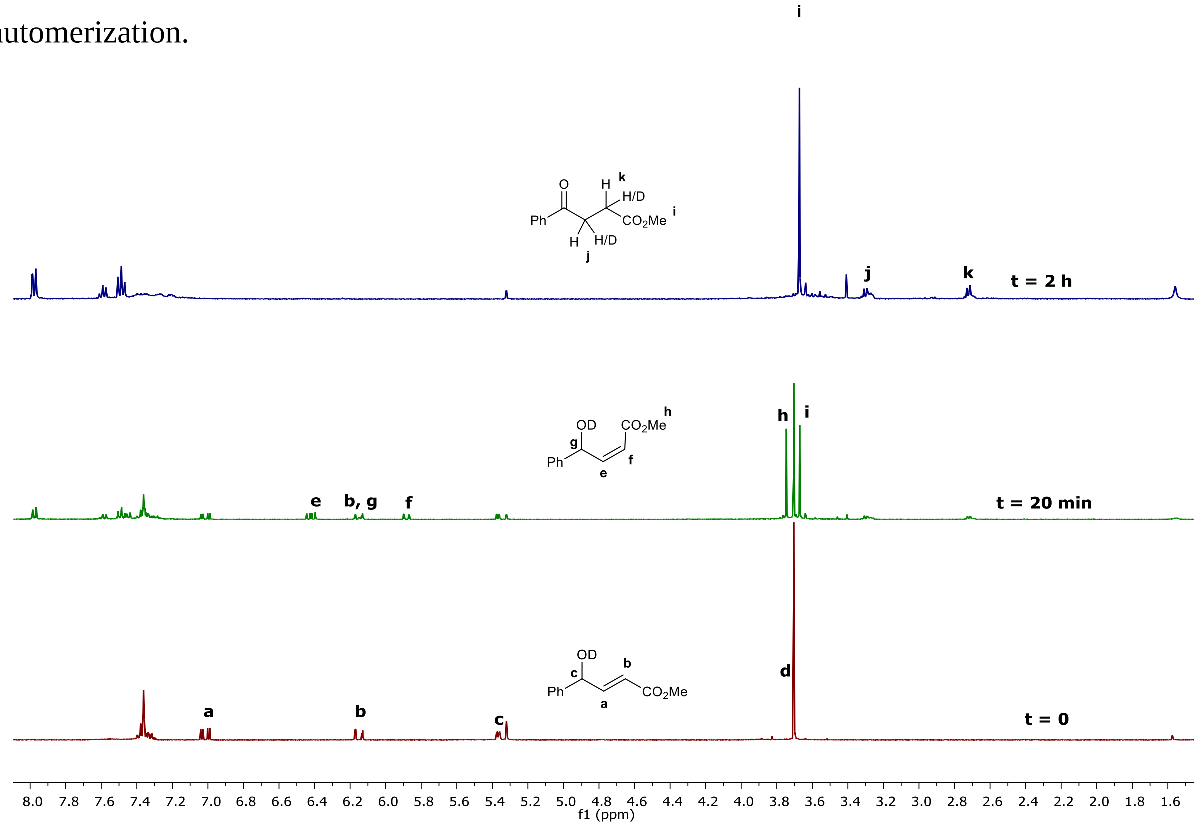

Figure S28. GC-MS analysis of reaction products of irradiation of ***E*-3a** in the presence of different amounts of D_2O.

1. Sample with traces of D_2O

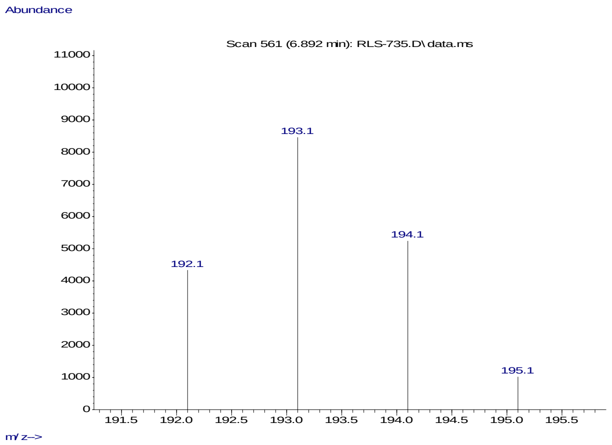

2. Sample with excess D_2O

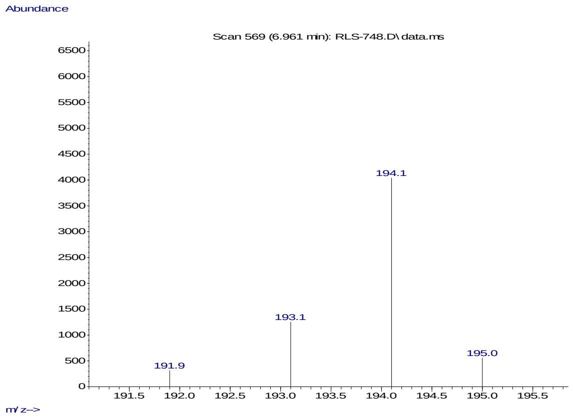

Figure S29. Selective *E*-*Z* isomerization-lactonization of ***E*-3b-D** (0.01 M in CD_2Cl_2) with 1 equiv. of phenanthrene on 254 nm irradiation at ~35 °C (^{1}H NMR, 400 MHz). The deuterium atom remains unscathed.

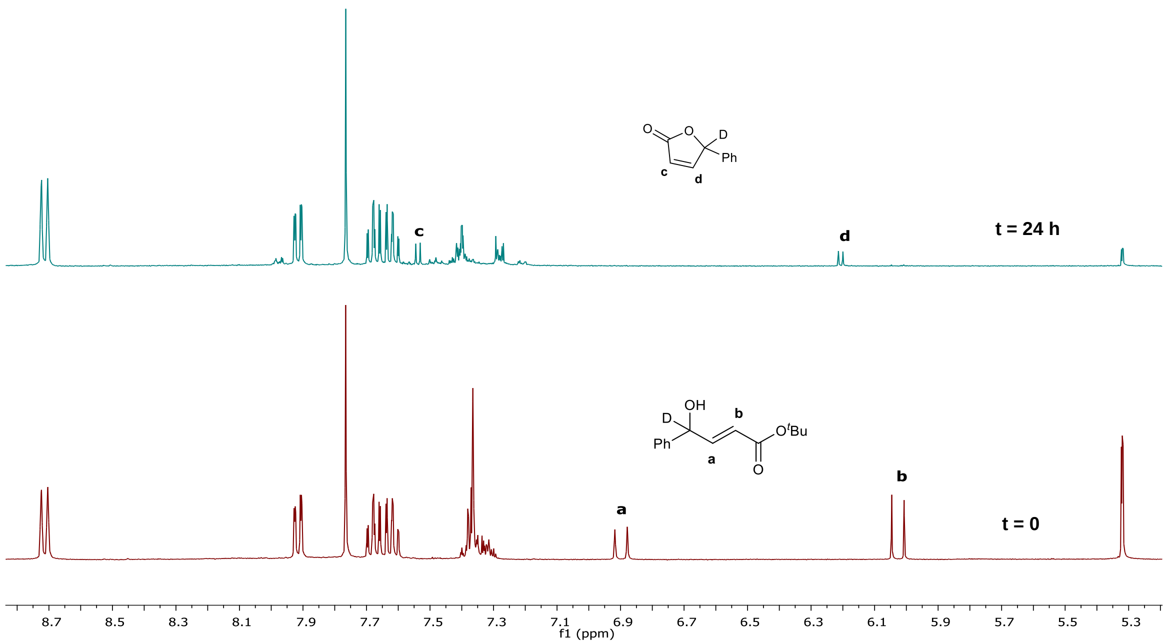

Figure S30. Irradiation of ***O*-TMS-*E*-3a** (0.01 M in CD_2Cl_2) with 254 nm irradiation (^{1}H NMR, 400 MHz).

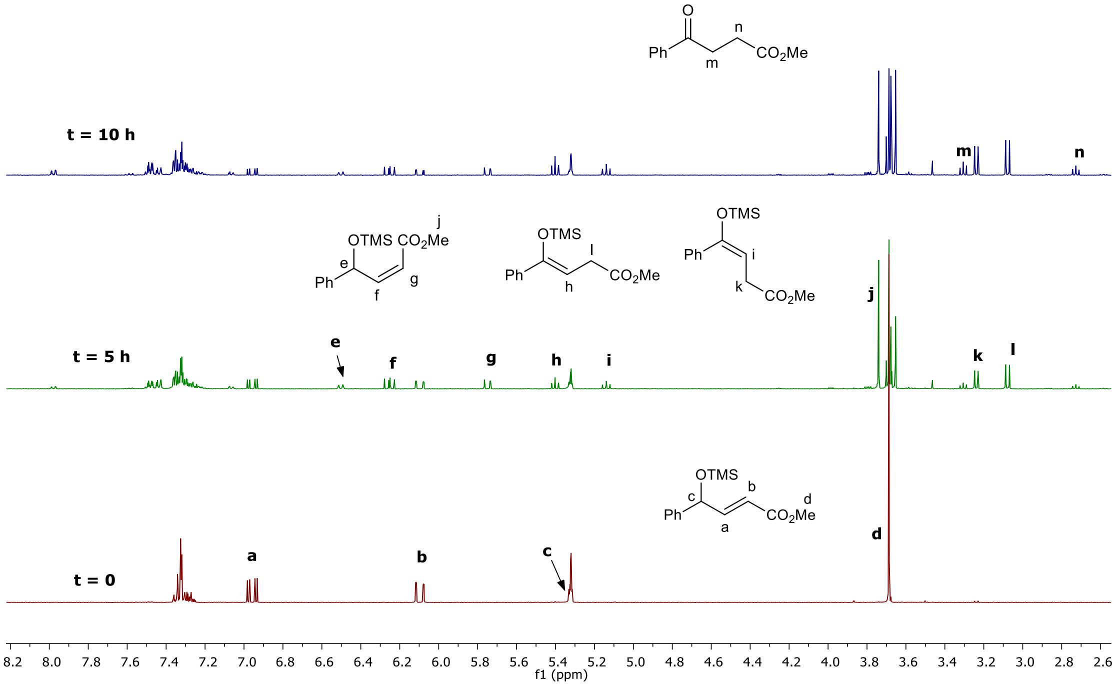

Figure S31. Effect of phenanthrene on photolactonization of **1e** (0.01 M in CD_2Cl_2 with 1,4-dioxane as internal standard) at 254 nm (^{1}H NMR, 400 MHz) (lactonization[8] (%) *vs* time)

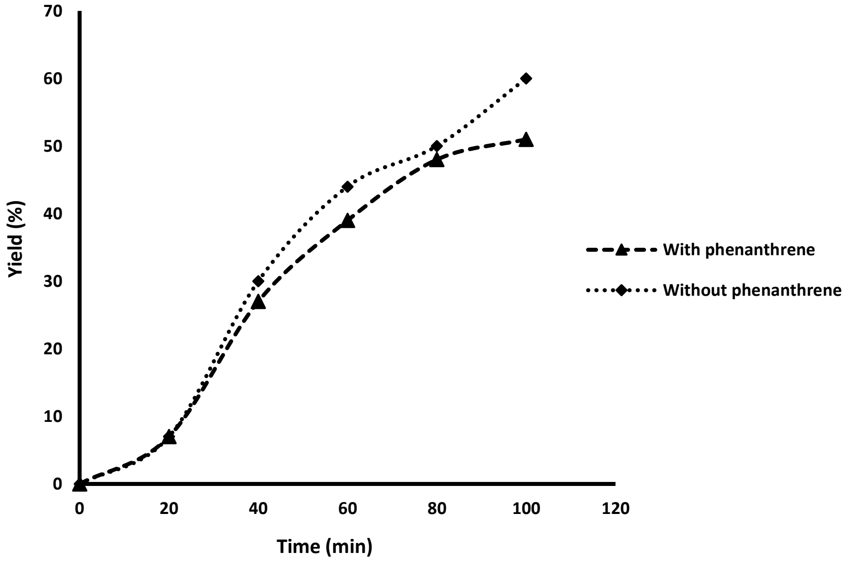

Figure S32. Effect of phenanthrene on photolactonization of ***E*-3a** (0.01 M in CD_2Cl_2 with 1,4-dioxane as internal standard) at 300 nm (^{1}H NMR, 400 MHz) (*conversions are indicated in the bracket*)

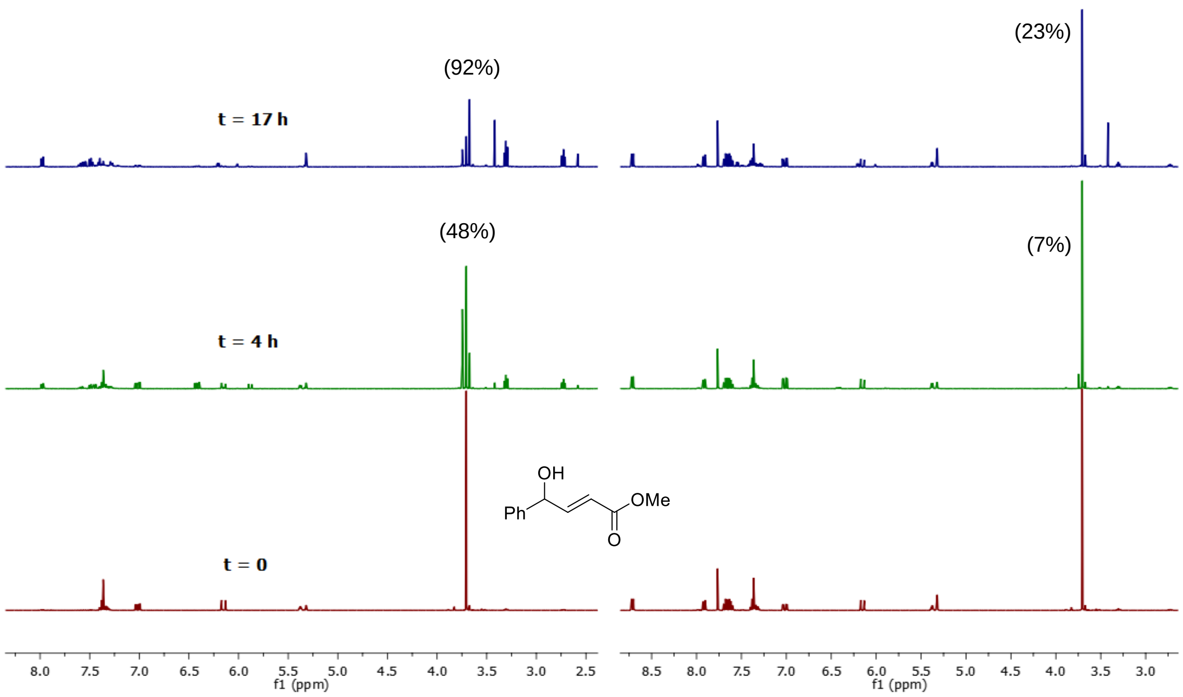

Figure S33. Setup for the UV-C filter controlled photochemical reactions.

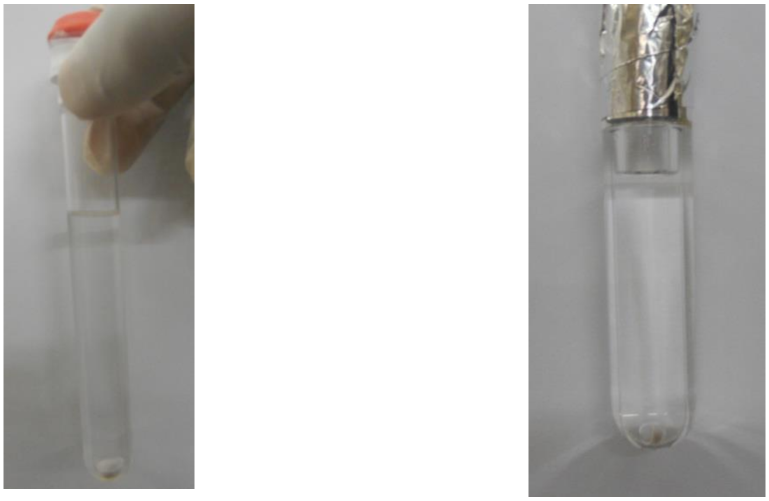

a. Internal UV-C filter **b. External UV-C filter**

For the reactions with internal UV-C filter, a quartz test tube (inner diameter- 15 mm, wall thickness 1 mm) equipped with magnetic stir bar was charged with the solution of *E*-**3** and phenanthrene in DCM. It was thoroughly purged with argon, capped with a rubber septum and irradiated directly to 254 nm light under slow stirring. While for the reactions with external UV-C filter; the similar quartz test tube was charged with the solution of *E*-**3** in DCM, purged with argon and capped with a rubber septum. It was immersed in another quartz tube (inner diameter- 25 mm) filled with the required UV-C filter solution. The upper part of the tube was covered with aluminum foil so as to just expose the reaction mass to UV-light and it was irradiated in the Rayonet under slow magnetic stirring.

4. General procedure of tandem divergent CM-photoisomerization

In a 4 mL glass vial, the solution of α-substituted allyl alcohol (**1**, 0.1 mmol), methyl acrylate (**2**, 1 mmol) and the catalyst, *cis*-**Ru I** or *cis*-**Ru II** (0.003 mmol) in dichloromethane was charged under argon and it was irradiated with 350 nm light in the Rayonet. After completion of the reaction, solvent and excess of methyl acrylate was removed under reduced pressure. The crude products were analyzed by ^{1}H NMR and GC-MS.

For the lactonization reaction, the required amount of phenanthrene was added to the residue and it was redissolved in dichloromethane (10 mL). A 15 mL quartz tube equipped with magnetic stir bar was charged with this solution and the solution was thoroughly purged with argon. The tube was closed with a rubber septum and irradiated at 254 nm in the Rayonet with

slow stirring and the reaction was monitored with TLC. After completion of the reaction, product was purified by silica gel column chromatography. While for photoisomerization to ketone, the residue obtained after cross-metathesis reaction was dissolved in the mixture of ^{t}BuOH (2 mL) and dichloromethane (8 mL). It was transferred to a 15 mL quartz tube equipped with magnetic stir bar, thoroughly purged with argon and the tube was closed with rubber septum. It was irradiated at 254 nm light inside the Rayonet with stirring and the reaction was monitored by TLC. After completion of the reaction, the solvent was evaporated and crude product was purified by silica gel column chromatography.

5. Characterization data of products from tandem divergent CM-photoisomerization

Table S2. Characterization data for the cross-metathesis products (**3**)[a]

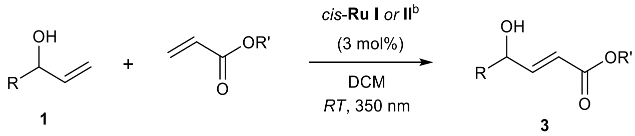

1 **3**

The crude cross-metathesis products **3a**,[10a] **3b**,[10b] **3j**,[10c] **3l**,[10a] **3o**[10a] and **3r**[10d] were characterized by ^{1}H NMR and the data has been found to be in good agreement with the reported values.

No	CM-product	Time	Characterization data
1	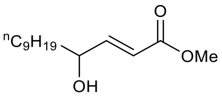<br>**3k**	12 h	White solid; 37–40 °C; R_f = 0.2 (EtOAc/hexane 1:4). ^{1}H NMR (400 MHz, $CDCl_3$): δ 6.95 (dd, *J* = 15.7, 4.9 Hz, 1H), 6.04 (dd, *J* = 15.7, 1.7 Hz, 1H), 4.33 – 4.30 (m, 1H), 3.75 (s, 3H), 1.66 – 1.50 (m, 3H), 1.47 – 1.32 (m, 2H), 1.32 – 1.23 (m, 12H), 0.88 (t, *J* = 6.9 Hz, 3H).<br><br>^{13}C NMR (100 MHz, $CDCl_3$): δ 167.0, 150.4, 119.7, 71.2, 51.6, 36.7, 31.9, 29.5, 29.4, 29.3, 25.2, 22.7, 14.1.<br><br>HRMS (ESI) for $C_{14}H_{26}O_3$ m/z: $[M+Na]^+$ Calculated: 265.1774; Found: 265.1772.
2	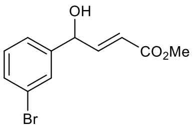<br>**3m**	17 h	Colorless oil; R_f = 0.2 (EtOAc/hexane 1:3).<br><br>^{1}H NMR (400 MHz, $CDCl_3$): δ 7.51 (t, *J* = 1.8 Hz, 1H), 7.45 (dt, *J* = 7.5, 1.8 Hz, 1H), 7.30 – 7.27 (m, 1H), 7.23

			(d, *J* = 7.7 Hz, 1H), 7.00 (dd, *J* = 15.6, 4.9 Hz, 1H), 6.17 (dd, *J* = 15.6, 1.7 Hz, 1H), 5.37 – 5.33 (m, 1H), 3.75 (s, 3H), 2.16 (d, *J* = 3.6 Hz, 1H).<br><br>^{13}C NMR (100 MHz, $CDCl_3$): δ 166.6, 147.9, 143.0, 131.5, 130.4, 129.6, 125.1, 122.9, 120.4, 72.8, 51.8. HRMS (ESI) for $C_{11}H_{11}BrO_3$ m/z: $[M+Na]^+$ Calculated: 292.9784 and 294.9763 (1:1); Found: 292.9818 and 294.9796.
3	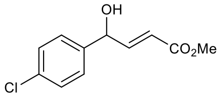<br>**3n**	30 h	Colorless oil; R_f = 0.2 (EtOAc/hexane 1:9).<br><br>^{1}H NMR (400 MHz, $CDCl_3$): δ 7.37 – 7.32 (m, 2H), 7.31 – 7.27 (m, 2H), 7.00 (dd, *J* = 15.6, 4.9 Hz, 1H), 6.16 (dd, *J* = 15.6, 1.7 Hz, 1H), 5.36 (d, *J* = 4.6 Hz, 1H), 3.74 (s, 3H), 2.15 (br s, 1H).<br><br>^{13}C NMR (100 MHz, $CDCl_3$): δ 166.7, 148.2, 139.3, 134.2, 129.0, 127.9, 120.2, 72.8, 51.7.<br><br>HRMS (ESI) for $C_{11}H_{11}ClO_3$ m/z: $[M+H]^+$ Calculated: 227.0469; Found: 227.0432.
4	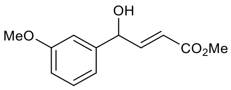<br>**3p**	17 h	Colorless oil; R_f = 0.2 (EtOAc/hexane 1:4).<br>^{1}H NMR (500 MHz, $CDCl_3$): δ 7.28 (t, *J* = 7.9 Hz, 1H), 7.04 (dd, *J* = 15.6, 4.8 Hz, 1H), 6.94 – 6.89 (m, 2H), 6.85 (ddd, *J* = 8.2, 2.6, 0.9 Hz, 1H), 6.17 (dd, *J* = 15.6, 1.7 Hz, 1H), 5.36 – 5.32 (m, 1H), 3.81 (s, 3H), 3.74 (s, 3H); 2.14 (d, *J* = 3.1 Hz, 1H).<br>^{13}C NMR (125 MHz, $CDCl_3$): δ 166.8, 160.0, 148.6, 142.5, 129.9, 119.9, 118.8, 113.9, 112.0, 73.4, 55.3, 51.7.<br>HRMS (ESI) for $C_{12}H_{14}O_4$ m/z: $[M+Na]^+$ Calculated: 245.0784, Found: 245.0813.

5	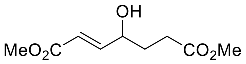 **3q**	10 h	Colorless oil; R$_f$ = 0.2 (EtOAc/hexane 1:3).<br><br>^{1}H NMR (400 MHz, CD_2Cl_2): δ 6.90 (dd, *J* = 15.6, 4.6 Hz, 1H), 6.05 (dd, *J* = 15.7, 1.8 Hz, 1H), 4.38 (dqd, *J* = 9.4, 4.7, 1.8 Hz, 1H), 3.71 (s, 3H), 3.66 (s, 3H), 2.45 (td, *J* = 7.1, 2.3 Hz, 2H), 2.35 (d, *J* = 4.8 Hz, 1H), 1.97 (dtd, *J* = 11.8, 7.3, 4.5 Hz, 1H), 1.82 (dt, *J* = 14.4, 7.2 Hz, 1H).<br><br>^{13}C NMR (100 MHz, CD_2Cl_2): δ 174.6, 167.1, 150.0, 120.6, 70.4, 52.1, 51.9, 31.5, 30.1.<br><br>HRMS (ESI) for $C_9H_{14}O_5$ m/z: $[M+Na]^+$ Calculated: 225.0733; Found: 225.0725.
6	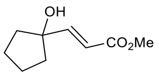 **3s**	15 h	Colorless oil; R$_f$ = 0.2 (EtOAc/hexane 1:9).<br><br>^{1}H NMR (400 MHz, $CDCl_3$): δ 7.05 (d, *J* = 15.6 Hz, 1H), 6.11 (d, *J* = 15.6 Hz, 1H), 3.74 (s, 3H), 1.96 – 1.87 (m, 2H), 1.82 – 1.67 (m, 6H), 1.40 (br s, 1H).<br><br>^{13}C NMR (100 MHz, $CDCl_3$): δ 167.3, 154.1, 117.8, 81.8, 51.6, 40.7, 24.0.<br><br>HRMS (ESI) for $C_9H_{14}O_3$ m/z: $[M+Na]^+$ Calculated: 193.0835; Found: 193.0852.

[a] All metathesis reactions were carried out at 0.05 M concentration of **1**, except for the synthesis of **3j** (0.025 M).

[b] Except for the entries 1 and 5 (*cis*-**Ru II**), for all reactions *cis*-**Ru I** was used as catalyst.

Table S3. Characterization data of γ-butenolides synthesized by tandem CM-photoisomerization.[a]

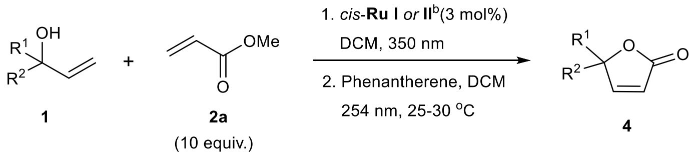

No	Butenolide	Time[c]	Yield and characterization data
1	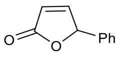 **4a**	24 h/ 36 h	Viscous oil,[11a] 11.5 mg (71%); R_f = 0.2 (DCM/hexane 1:2 with 0.5% TFA).<br><br>^{1}H NMR (400 MHz, $CDCl_3$) δ: 7.53 (dd, *J* = 5.7, 1.6 Hz, 1H), 7.44 – 7.35 (m, 3H), 7.28 (d, *J* = 2.5 Hz, 2H), 6.24 (dd, *J* = 5.6, 2.1 Hz, 1H), 6.02 (t, *J* = 1.8 Hz, 1H).<br><br>GC-MS (EI) for $C_{10}H_8O_2$, M^+ Calculated: 160.0, Found: 160.0.
2	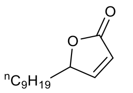 **4b**	12 h/ 45 h	Viscous oil,[11b] 14.7 mg (70%); R_f = 0.2 (EtOAc/hexane 1:9).<br><br>^{1}H NMR (400 MHz, $CDCl_3$) δ: 7.44 (dd, *J* = 5.7, 1.5 Hz, 1H), 6.10 (dd, *J* = 5.7, 2.0 Hz, 1H), 5.05 – 5.01 (m, 1H), 1.82 – 1.61 (m, 2H), 1.48 – 1.40 (m, 2H), 1.26 (m, 12H), 0.88 (t, *J* = 6.9 Hz, 3H).<br><br>GC-MS (EI) for $C_{13}H_{22}O_2$, M^+ Calculated: 210.2, Found: 210.1
3	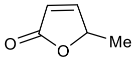 **4e**	17 h/ 60 h	Colorless oil,[11c] 6 mg (61%); R_f = 0.2 (DCM/hexane 1:2 with 0.5% TFA).<br><br>^{1}H NMR (400 MHz, $CDCl_3$) δ: 7.45 (dd, *J* = 5.7, 1.5 Hz, 1H), 6.10 (dd, *J* = 5.7, 2.0 Hz, 1H), 5.30 – 5.07 (qt, 6.9, 1.7 Hz, 1H), 1.46 (d, *J* = 6.9 Hz, 3H).
4	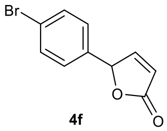 **4f**	29 h/ 36 h	Yellow oil,[11a] 16.6 mg (70%); R_f = 0.2 (DCM/hexane 2:3 with 0.5% TFA): 0.2.<br><br>^{1}H NMR (400 MHz, $CDCl_3$) δ: 7.55 – 7.52 (m, 2H), 7.50 (dd, *J*

			= 5.6, 1.7 Hz, 1H), 7.15 (d, *J* = 8.5 Hz, 2H), 6.24 (dd, *J* = 5.6, 2.1 Hz, 1H), 5.97 (t, *J* = 1.8 Hz, 1H).<br><br>GC-MS (EI) for $C_{10}H_7BrO_2$, M+ Calculated: 238.0 and 240.0 (1:1) Found: 237.9 and 239.9.
5	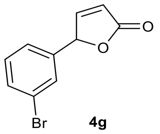<br>4g	17 h/<br>36 h	Colorless oil, 16 mg (67%); R_f = 0.2 (DCM/hexane 2:3 with 0.5% TFA): 0.2.<br><br>^{1}H NMR (400 MHz, $CDCl_3$): δ 7.54 – 7.47 (m, 2H), 7.42 (dd, *J* = 2.6, 1.0 Hz, 1H), 7.28 (t, *J* = 7.8 Hz, 1H), 7.23 – 7.20 (m, 1H), 6.26 – 6.24 (m, 1H), 5.97 (t, *J* = 1.8 Hz, 1H).<br><br>^{13}C NMR (100 MHz, $CDCl_3$): δ 155.2, 132.4, 130.6, 129.5, 125.0, 123.1, 121.3, 83.3.<br>HRMS (ESI) for $C_{10}H_7BrO_2$ m/z: $[M+H]^+$ Calculated: 238.9702 and 240.9706 (1:1) Found: 238.9726 and 240.9682; $[M+Na]^+$ Calculated: 260.9522 and 262.9502 (1:1) Found: 260.9548 and 262.9526.
6	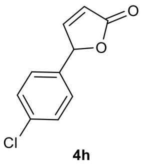<br>4h	30 h/<br>36 h	Colorless oil, 14 mg (72%); R_f = 0.2 (DCM/hexane 2:3 with 0.5% TFA): 0.2.<br><br>^{1}H NMR (400 MHz, $CDCl_3$): δ 7.51 (dd, *J* = 5.6, 1.6 Hz, 1H), 7.41 – 7.35 (m, 2H), 7.23 – 7.18 (m, 2H), 6.24 (dd, *J* = 5.6, 2.1 Hz, 1H), 5.99 (t, *J* = 1.7 Hz, 1H).<br><br>^{13}C NMR (100 MHz, $CDCl_3$): δ 155.3, 135.3, 132.8, 129.3, 128.9, 127.8, 121.3, 83.5.<br><br>HRMS (ESI) for $C_{10}H_7ClO_2$ m/z: $[M+Na]^+$ Calculated: 217.0027, Found: 217.0049.
7	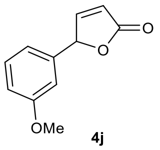<br>4j	17 h/<br>36 h	Colorless oil,[11d] 12 mg (63%); R_f = 0.2 (DCM/hexane 1:1 with 0.5% TFA).

			^{1}H NMR (400 MHz, $CDCl_3$): δ 7.52 (dd, *J* = 5.6, 1.7 Hz, 1H), 7.31 (t, *J* = 7.9 Hz, 1H), 6.90 (ddd, *J* = 8.3, 2.5, 0.9 Hz, 1H), 6.85 (d, *J* = 7.6 Hz, 1H), 6.80 – 6.77 (m, 1H), 6.21 (dd, *J* = 5.6, 2.1 Hz, 1H), 5.98 (t, *J* = 1.8 Hz, 1H), 3.80 (s, 3H).<br><br>GC-MS (EI) for $C_{11}H_{10}O_3$, M^+ Calculated: 190.0, Found: 190.0.
8	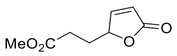<br>4k	10 h/<br>26 h	Colorless oil; 9 mg (53%); R_f = 0.2 (EtOAc/hexane 1:3).<br><br>^{1}H NMR (400 MHz, $CDCl_3$): δ 7.45 (dd, *J* = 5.7, 1.5 Hz, 1H), 6.13 (dd, *J* = 5.7, 2.0 Hz, 1H), 5.20 – 5.05 (m, 1H), 3.69 (s, 3H), 2.59 – 2.46 (m, 2H), 2.23 (dtd, *J* = 11.8, 7.5, 4.2 Hz, 1H), 1.94 – 1.84 (m, 1H).<br><br>^{13}C NMR (100 MHz, $CDCl_3$): 172.9, 172.7, 155.6, 122.0, 81.9, 51.9, 29.0, 28.13.<br><br>HRMS (ESI) for $C_8H_{10}O_4$ m/z: $[M+H]^+$ Calculated: 171.065, Found: 171.0648.
	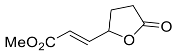<br>8		Colorless oil; R_f = 0.22 (EtOAc/hexane 1:3).<br><br>^{1}H NMR (400 MHz, $CDCl_3$): δ 6.92 (dd, *J* = 15.7, 4.7 Hz, 1H), 6.12 (dd, *J* = 15.7, 1.7 Hz, 1H), 5.22 – 4.95 (m, 1H), 3.77 (s, 3H), 2.60 – 2.54 (m, 2H), 2.54 – 2.46 (m, 1H), 2.11 – 2.01 (m, 1H).<br><br>^{13}C NMR (101 MHz, $CDCl_3$): δ 176.0, 166.1, 143.8, 121.7, 77.7, 51.9, 27.8, 27.6.<br><br>HRMS (ESI) for $C_8H_{10}O_4$ m/z: $[M+Na]^+$ Calculated: 193.0471, Found: 193.0463.

9	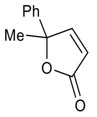 **4l**	20 h/ 36 h	Colorless oil,[11e] 9 mg (52%); R_f = 0.25 (EtOAc/hexane 1:4).<br><br>^{1}H NMR (400 MHz, $CDCl_3$): δ 7.63 (d, *J* = 5.6 Hz, 1H), 7.38 (d, *J* = 4.5 Hz, 4H), 7.33 (dd, *J* = 4.8, 3.7 Hz, 1H), 6.06 (d, *J* = 5.6 Hz, 1H), 1.83 (s, 3H).<br><br>GC-MS (EI) for $C_{11}H_{10}O_2$, M^+ Calculated: 174.2, Found: 174.0.
10	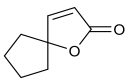 **4m**	15 h/ 45 h	Colorless oil,[11e] 10 mg (72%); R_f = 0.3 (EtOAc/hexane 1:4).<br><br>^{1}H NMR (400 MHz, $CDCl_3$): δ 7.36 (d, *J* = 5.6 Hz, 1H), 6.00 (d, *J* = 5.6 Hz, 1H), 2.13 – 1.77 (m, 8H).<br><br>GC-MS (EI) for $C_8H_{10}O_2$, M^+ Calculated: 138.1, Found: 138.0.

[a] For entries 2, 3, 8 and 10, 0.3 equiv., for entries 1 and 4–7, 2.1 equiv. and for entry 9, 2.3 equiv. of phenanthrene was used as internal UV-C filter. [b] Except for the entries 2 and 8 (*cis*-**Ru II**), for all reactions *cis*-**Ru I** was used as catalyst. [c] The first figure indicates time of CM reaction.

Table S4. Characterization data of γ-ketoesters synthesized by tandem CM-photoisomerization.

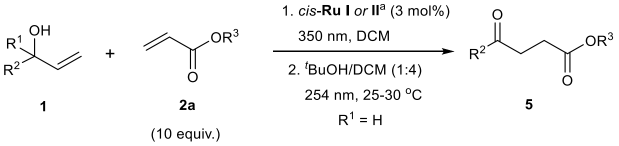

No	γ-ketoester	Time[b]	Yield and characterization data
1	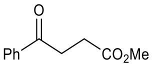 **5a**	4.5 h[c]	Colorless oil,[10a] 14 mg (73%); R_f = 0.2 (EtOAc/hexane 1:6).<br>^{1}H NMR (400 MHz, $CDCl_3$) δ: 8.04 – 7.94 (m, 2H), 7.61 – 7.54 (tt, *J* = 7.4, 1.3 Hz, 1H), 7.50 – 7.45 (tt, *J* = 7.8, 1.3 Hz, 2H), 3.71 (s, 3H), 3.33 (t, *J* = 6.6 Hz, 2H), 2.78 (t, *J* = 6.6 Hz, 2H).<br>GC-MS (EI) for $C_{11}H_{12}O_3$, M^+ Calculated: 192.1, Found: 192.0.

2[d]	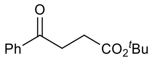<br>**5b**	4.5 h	Colorless oil,[11f] 14 mg (60%); R_f = 0.3 (EtOAc/hexane 1:9).<br><br>^{1}H NMR (400 MHz, $CDCl_3$): δ 7.99 (ddd, *J* = 7.2, 3.0, 1.6 Hz, 2H), 7.56 (tt, *J* = 7.4, 2.1 Hz, 2H), 7.50 – 7.44 (m, 1H), 3.26 (t, *J* = 6.7 Hz, 2H), 2.68 (t, *J* = 6.7 Hz, 2H), 1.45 (s, 9H).<br><br>GC-MS (EI) for $C_{14}H_{18}O_3$, M^+ Calculated: 234.3, Found: 234.9.
3	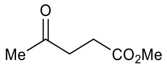<br>**5j**	7 h	Colorless liquid,[11g] 8.6 mg (66%); R_f = 0.2 (EtOAc/ hexane 1:9).<br><br>^{1}H NMR (400 MHz, $CDCl_3$) δ: 3.68 (s, 3H), 2.77 (t, J = 6.5 Hz, 2H), 2.59 (t, *J* = 6.6 Hz, 2H), 2.20 (s, 3H).
4	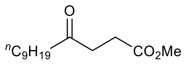<br>**5k**	5 h	Colorless oil, 15 mg (62%); R_f = 0.2 (EtOAc/hexane 1:9).<br><br>^{1}H NMR (400 MHz, $CDCl_3$) δ: 3.68 (s, 3H), 2.72 (t, *J* = 6.5 Hz, 2H), 2.58 (t, *J* = 6.5 Hz, 2H), 2.44 (t, *J* = 7.5 Hz, 2H), 1.67 – 1.56 (m, 2H), 1.37 – 1.20 (s, 12H), 0.88 (t, *J* = 6.8 Hz, 3H).<br><br>^{13}C NMR (100 MHz, $CDCl_3$): δ 209.2, 173.3, 51.8, 42.8, 37.0, 31.8, 29.4, 29.2, 27.7, 25.1, 23.8, 22.6, 14.1.<br><br>HRMS (ESI) for $C_{14}H_{26}O_3$ m/z: $[M+Na]^+$ Calculated: 265.1774, Found: 265.1772.
5	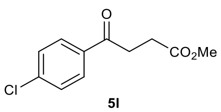<br>**5l**	4.5 h	Colorless oil,[11h] 16 mg (71%); R_f = 0.35 (EtOAc/hexane 1:4).<br><br>^{1}H NMR (400 MHz, $CDCl_3$): δ 7.92 (d, *J* = 8.7 Hz, 2H), 7.44 (d, *J* = 8.7 Hz, 2H), 3.70 (s, 3H), 3.28 (t, *J* = 6.6 Hz, 2H), 2.76 (t, *J* = 6.6 Hz, 2H).

			GC-MS (EI) for $C_{11}H_{11}ClO_3$, M^+ Calculated: 226.0 Found: 226.0.
6	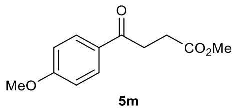<br>**5m**	4 h	Colorless oil,[10a] 17 mg (76%); R_f = 0.3 (EtOAc/hexane 1:4).<br><br>^{1}H NMR (400 MHz, $CDCl_3$) δ 7.97 (d, *J* = 8.3 Hz, 2H), 6.94 (d, *J* = 8.3 Hz, 2H), 3.87 (s, 3H), 3.70 (s, 3H), 3.27 (t, *J* = 6.8 Hz, 2H), 2.75 (t, *J* = 6.7 Hz, 2H).<br><br>GC-MS (EI) for $C_{12}H_{14}O_4$, M^+ Calculated: 222.1, Found: 222.0.
7	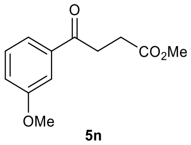<br>**5n**	4.5 h	Colorless oil,[11i] 15 mg (67%); R_f = 0.35 (EtOAc/hexane 1:4).<br><br>^{1}H NMR (400 MHz, $CDCl_3$): δ 7.59 – 7.55 (m, 1H), 7.50 (dd, *J* = 2.6, 1.6 Hz, 1H), 7.37 (t, *J* = 7.9 Hz, 1H), 7.12 (ddd, *J* = 8.2, 2.7, 0.9 Hz, 1H), 3.85 (s, 3H), 3.71 (s, 3H), 3.31 (t, *J* = 6.6 Hz, 2H), 2.76 (t, *J* = 6.6 Hz, 2H).<br><br>GC-MS (EI) for $C_{12}H_{14}O_4$, M^+ Calculated: 222.1, Found: 222.0.
8	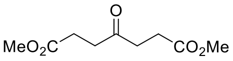<br>**5o**	2 h	Colorless solid.[11j] 12.5 mg (62%); R_f = 0.2 (EtOAc/hexane 1:4).<br><br>^{1}H NMR (400 MHz, $CDCl_3$): δ 3.67 (s, 6H), 2.78 (t, *J* = 6.5 Hz, 4H), 2.61 (t, *J* = 6.5 Hz, 4H).<br><br>GC-MS (EI) for $C_9H_{14}O_5$, M^+ Calculated: 202.1, Found: 202.1.

[a] Except for the entries 4 and 8 (*cis*-**Ru II**), for all reactions *cis*-**Ru I** was used as catalyst. [b] For the time of metathesis reaction see Table S3. [c] Both **3l** and **3m** isomerized to **3a** on 5 h irradiation at 254 nm light. [d] Only DCM was used as the solvent.

6. Characterization data of products from sequential divergent CM-photoisomerization

Following the above general procedure for tandem divergent CM-photoisomerization, the crude CM-products obtained were purified by silica gel column chromatography and used for the next photoisomerization reactions.

6.1 *tert*-Butyl (*E*)-4-hydroxy-4-phenylbut-2-enoate-4-*d* (3b-D)

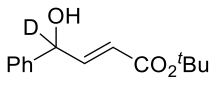

3b-D

A 5 mL glass vial was charged with 1-Phenylprop-2-en-1-*d*-1-ol **(1a-D)** (27 mg, 0.2 mmol), *tert*-butyl acrylate (**2b**, 0.3 mL, 2 mmol) and *cis*-**Ru I** (4.1 mg, 0.006 mmol) in DCM (4 mL) under the argon atmosphere. The vial was flushed with argon, caped and irradiated in the Rayonet with 350 nm light. After completion of the reaction (17 h), solvent was evaporated and the residue was purified by silica gel column chromatography using EtOAc/hexane (1:4) as the eluent to get **3b-D** (40 mg, 85%) as a white solid. Mp 46–47 °C; R_f = 0.25 (EtOAc/hexane 1:4); ^{1}H NMR (400 MHz, $CDCl_3$): δ 7.43 – 7.30 (m, 5H), 6.94 (d, *J* = 15.6 Hz, 1H), 6.07 (d, *J* = 15.6 Hz, 1H), 2.06 (s, 1H), 1.47 (s, 9H); ^{13}C NMR (100 MHz, $CDCl_3$) δ: 165.7, 147.1, 141.1, 128.8, 128.3, 126.5, 122.4, 80.6, 73.2 (t, *J* = 23 Hz), 28.1; HRMS (ESI) for $C_{14}H_{17}DO_3$ m/z: $[M+Na]^+$ Calculated: 258.1211; Found: 258.1210.

6.2 *n*-Butyl (*E*)-4-hydroxy-4-phenylbut-2-enoate (3c)

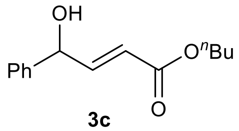

3c

A 20 mL glass vial was charged with 1-Phenylprop-2-en-1-ol (**1a**) (67 mg, 0.5 mmol), *n*-butyl acrylate (**2c**, 0.72 mL, 5 mmol) and *cis*-**Ru II** (10 mg, 0.015 mmol) in DCM (10 mL) under the argon atmosphere. The vial was flushed with argon, caped and irradiated in the Rayonet with 350 nm light. After completion of the reaction (12 h), solvent was evaporated and the residue was purified by silica gel column chromatography using EtOAc/hexane (1:6) as the eluent to get **3c** (90 mg, 77%) as a viscous oil. R_f = 0.5 (EtOAc/hexane 1:4); ^{1}H NMR (400 MHz, $CDCl_3$): δ 7.41 – 7.29 (m, 5H), 7.04 (dd, *J* = 15.6, 4.9 Hz, 1H), 6.16 (dd, *J* = 15.6, 1.7 Hz, 1H), 5.36 (dd, *J* = 4.8, 1.7 Hz, 1H), 4.13 (t, *J* = 6.7 Hz, 2H), 2.12 (br s, 1H), 1.70 – 1.57 (m, 2H), 1.39 (tdd, *J* = 14.6, 8.5, 6.5 Hz, 2H), 0.93 (t, *J* = 7.4 Hz, 3H); ^{13}C NMR (100 MHz, $CDCl_3$): δ 166.5, 148.4, 140.9,

128.8, 128.4, 126.6, 120.3, 73.6, 64.4, 30.6, 19.1, 13.7; HRMS (ESI) for $C_{14}H_{18}O_3$ m/z: $[M+H]^+$ Calculated: 235.1329; Found: 235.1339; $[M+Na]^+$ Calculated: 251.1148; Found: 251.1157.

6.3 *n*-Butyl 4-oxo-4-phenylbutanoate (5c) [11k]

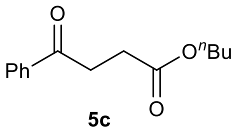

5c

From the solution of **3c** (24 mg, 0.1 mmol) in 10 mL of ^{t}BuOH/DCM (1:4), on irradiation with 254 nm light for 2 h and purification by silica gel column chromatography using ethyl acetate/hexane (1:12) as the eluent, **5c** (16 mg, 68%) was obtained as colorless oil. R_f = 0.4 (EtOAc/hexane 1:9); ^{1}H NMR (400 MHz, $CDCl_3$): δ 8.02 – 7.97 (m, 2H), 7.57 (ddd, *J* = 6.8, 4.0, 1.3 Hz, 1H), 7.51 – 7.44 (m, 2H), 4.10 (t, *J* = 6.7 Hz, 2H), 3.32 (t, *J* = 6.7 Hz, 2H), 2.77 (t, *J* = 6.7 Hz, 2H), 1.61 (dtd, *J* = 8.7, 7.0, 6.0 Hz, 2H), 1.44 – 1.32 (m, 2H), 0.92 (t, *J* = 7.4 Hz, 3H); GC-MS (EI) for $C_{14}H_{18}O_3$, M+ Calculated: 234.13; Found: 234.1.

6.4 Benzyl (*E*)-4-hydroxy-4-phenylbut-2-enoate (3d)

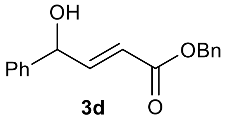

3d

The CM reaction of **1a** (67 mg, 0.5 mmol) and benzyl acrylate (**2d**, 810 mg, 5 mmol) was carried out in the same manner as described above for **3c** using *cis*-**Ru II** (10 mg, 0.015 mmol) as the catalyst in DCM (10 mL). After 3 h irradiation at 350 nm followed by purification with silica gel column chromatography using EtOAc/hexane (1:4) as the eluent gave **3d** (110 mg, 82%) as a colorless oil; R_f = 0.23 (EtOAc/hexane 1:4); ^{1}H NMR (400 MHz, $CDCl_3$): δ 7.42 – 7.29 (m, 10H), 7.10 (dd, *J* = 15.6, 4.8 Hz, 1H), 6.23 (dd, *J* = 15.6, 1.8 Hz, 1H), 5.38 (d, *J* = 4.2 Hz, 1H), 5.18 (s, 2H), 2.05 (s, 1H); ^{13}C NMR (100 MHz, $CDCl_3$): δ 166.2, 149.0, 140.8, 135.8, 128.9, 128.5, 128.4, 128.3, 127.0, 126.6, 120.0, 73.6, 66.4; HRMS (ESI) for $C_{17}H_{16}O_3$ m/z: $[M+Na]^+$ Calculated: 291.0992; Found: 291.1000.

6.5 Benzyl 4-oxo-4-phenylbutanoate (5d)[11l]

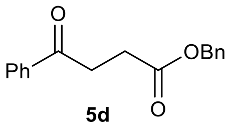

5d

From the solution of **3d** (27 mg, 0.1 mmol) in 10 mL of ^{t}BuOH/DCM (1:4), on irradiation of 254 nm light for 2 h and purification by silica gel column chromatography using ethyl acetate/hexane (1:12) as the eluent offered **5d** (18 mg, 67%) as colorless oil. R_f = 0.35 (EtOAc/hexane 1:9); ^{1}H NMR (400 MHz, $CDCl_3$): δ 7.99 (dt, *J* = 8.5, 1.7 Hz, 2H), 7.61 – 7.55 (m, 1H), 7.50 – 7.44 (m, 2H), 7.41 – 7.27 (m, 5H), 5.16 (s, 2H), 3.34 (t, *J* = 6.6 Hz, 2H), 2.83 (t, *J* = 6.6 Hz, 2H); GC-MS (EI) for $C_{17}H_{16}O_3$, M+ Calculated: 268.11; Found: 267.9.

6.6 4-(*tert*-Butyl)phenyl (*E*)-4-hydroxy-4-phenylbut-2-enoate (3e)

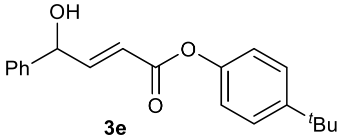

3e

As described above for **3c**, the CM reaction of **1a** (67 mg, 0.5 mmol) and 4-(*tert*-Butyl)phenyl acrylate (**2e**, 1.02 g, 5 mmol) was carried out using *cis*-**Ru II** (10 mg, 0.015 mmol). After 7 h of irradiation at 350 nm followed by purification with silica gel column chromatography using EtOAc/hexane (1:4) as the eluent gave **3e** (115 mg, 74%) as an oil; R_f = 0.23 (EtOAc/hexane 1:4); ^{1}H NMR (400 MHz, $CDCl_3$): δ 7.51 – 7.30 (m, 7H), 7.24 (dd, *J* = 15.6, 4.7 Hz, 1H), 7.06 – 7.01 (m, 2H), 6.38 (dd, *J* = 15.6, 1.8 Hz, 1H), 5.45 (dd, *J* = 4.7, 1.7 Hz, 1H), 1.86 (br s, 1H), 1.32 (s, 9H); ^{13}C NMR (100 MHz, $CDCl_3$): δ 164.9, 150.2, 148.6, 148.3, 140.7, 129.0, 128.6, 126.6, 126.3, 120.8, 119.7, 73.7, 34.5, 31.4; HRMS (ESI) for $C_{20}H_{22}O_3$ m/z: $[M+H]^+$ Calculated: 311.1642; Found: 311.1655; $[M+Na]^+$ Calculated: 333.1461; Found: 333.1470.

6.7 Benzyl (*E*)-4-hydroxy-tridec-2-enoate (3f)

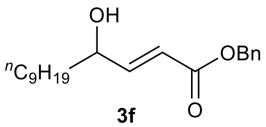

3f

The CM reaction of **1b** (92.1 mg, 0.5 mmol) and benzyl acrylate (**2d**, 810 mg, 5 mmol) was carried out in the same manner as described above for **3c** using *cis*-**Ru II** (10 mg, 0.015 mmol) in DCM (10 mL). After 4 h total irradiation at 350 nm followed by purification with silica gel column chromatography using EtOAc/hexane (1:12) as the eluent provided **3f** (125 mg, 78%) as a white solid; Mp 38–40 °C; R_f = 0.23 (EtOAc/hexane 1:9); ^{1}H NMR (400 MHz, $CDCl_3$): δ 7.46 – 7.28 (m, 5H), 6.99 (dd, *J* = 15.7, 4.9 Hz, 1H), 6.09 (dd, *J* = 15.7, 1.7 Hz, 1H), 5.19 (s, 2H), 4.31 (ddd, *J* = 10.6, 7.0, 1.7 Hz, 1H), 1.66 – 1.49 (m, 3H), 1.47 – 1.33 (m, 2H), 1.28 (d, *J* = 14.9

Hz, 12H), 0.88 (t, *J* = 6.9 Hz, 3H); ^{13}C NMR (100 MHz, $CDCl_3$): δ 166.3, 150.8, 135.9, 128.5, 128.2, 119.8, 71.2, 66.3, 36.6, 31.8, 29.5, 29.4, 29.3, 25.2, 22.6, 14.1; HRMS (ESI) for $C_{20}H_{30}O_3$ m/z: $[M+H]^+$ Calculated: 319.2268; Found: 319.2281; $[M+Na]^+$ Calculated: 341.2087; Found: 341.2096.

6.8 4-(*tert*-Butyl)phenyl (*E*)-4-hydroxytridec-2-enoate (3g)

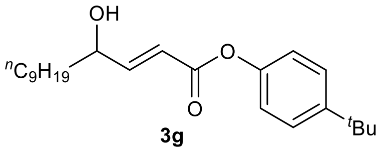

The CM reaction of **1b** (92.1 mg, 0.5 mmol) and 4-(*tert*-butyl) phenylacrylate (**2e**, 1.02 g, 5 mmol) in the presence of *cis*-**Ru II** (10 mg, 0.015 mmol) in DCM (10 mL) on irradiation at 350 nm for 18 h followed by purification with silica gel column chromatography using ethyl acetate/hexane (1:12) as the eluent furnished **3g** (135 mg, 75%). R_f = 0.22 (EtOAc/hexane 1:9); ^{1}H NMR (400 MHz, $CDCl_3$): δ 7.48 – 7.33 (m, 2H), 7.14 (dd, *J* = 15.6, 4.8 Hz, 1H), 7.07 – 6.99 (m, 2H), 6.25 (dd, *J* = 15.6, 1.7 Hz, 1H), 4.48 – 4.33 (m, 1H), 1.70 – 1.59 (m, 2H), 1.51 – 1.40 (m, 2H), 1.35 – 1.26 (m, 21H), 0.88 (t, *J* = 6.9 Hz, 3H); ^{13}C NMR (100 MHz, $CDCl_3$): δ 165.0, 152.1, 148.6, 148.3, 126.3, 120.8, 119.5, 71.2, 36.7, 34.5, 31.9, 31.4, 29.5, 29.5, 29.3, 25.2, 22.7, 14.1; HRMS (ESI) for $C_{23}H_{36}O_3$ m/z: $[M+H]^+$ Calculated: 361.2737; Found: 361.2751; $[M+Na]^+$ Calculated: 383.2557; Found: 383.2563.

6.9 *tert*-Butyl (*R*,*E*)-4,5-dihydroxypent-2-enoate (3h)

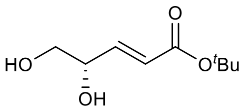

3h

The reaction of (*S*)-3,4-dihydroxy-1-butene (**1c**) (70 mg, 0.8 mmol) and *tert*-butyl acrylate (**2b**, 1.17 mL, 8 mmol) was carried out in the presence of *cis*-**Ru II** (32 mg, 0.048 mmol) with half portion of catalyst addition after 7 h. On complete irradiation for 20 h at 350 nm and purification by silica gel column chromatography using EtOAc/hexane (1:1) gave **3h** (70 mg, 46%) as viscous oil. R_f = 0.2 (EtOAc/hexane 1:1); $[\alpha]_D^{23}$ = -6.16 (*c* 4.87, $CHCl_3$); ^{1}H NMR (400 MHz, $CDCl_3$): δ 6.79 (dd, *J* = 15.7, 4.7 Hz, 1H), 6.05 (dd, *J* = 15.7, 1.8 Hz, 1H), 4.42 – 4.38 (m, 1H), 3.74 (d, *J* = 11.2 Hz, 1H), 3.54 (dd, *J* = 11.0, 7.1 Hz, 1H), 2.98 (br s, 1H), 2.60 (br s, 1H), 1.47

(s, 9H); ^{13}C NMR (100 MHz, $CDCl_3$): δ 165.7, 144.6, 124.0, 80.9, 71.7, 65.6, 28.1; HRMS (ESI) for $C_9H_{16}O_4$ m/z: $[M+Na]^+$ Calculated: 211.0941; Found: 211.0938.

6.10 (*S*)-5-(Hydroxymethyl)furan-2(5H)-one (4c)[12a]

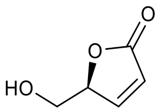

4c

From **3h** (19 mg, 0.1 mmol) using phenanthrene (5.4 mg, 0.03 mmol) as internal UV-C filter, on 45 h irradiation at 254 nm and purification by silica gel column chromatography using DCM/Et_2O (7:3) as the eluent furnished **4c** (7 mg, 61%) as viscous oil; R_f = 0.2 (DCM/Et_2O 7:3); $[\alpha]^{23}_{D}$ = -130 (*c* 1.0, H_2O); [Reported in Sigma-Aldrich, $[\alpha]^{23}_{D}$ = -144 (*c* 1.0, H_2O)] ^{1}H NMR (400 MHz, $CDCl_3$): δ 7.48 (dd, *J* = 5.8, 1.5 Hz, 1H), 6.21 (dd, *J* = 5.8, 2.0 Hz, 1H), 5.16 (ddd, *J* = 7.2, 3.7, 1.9 Hz, 1H), 4.09 – 3.93 (m, 1H), 3.78 (dt, *J* = 17.4, 5.5 Hz, 1H), 2.18 (t, *J* = 6.5 Hz, 1H); GC-MS (EI) for $C_5H_6O_3$, M^+ Calculated: 114.03, Found: 113.90.

6.11 *tert*-Butyl 5-hydroxy-4-oxopentanoate (5h)[12b]

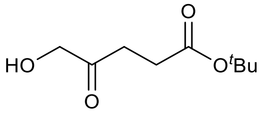

5h

From the solution of **3h** (19 mg, 0.1 mmol) in DCM (10 mL), using 0.1 mM aqueous solution of adenosine as an external UV-C filter, on irradiation at 254 nm for 5 h and purification by silica gel column chromatography using EtOAc/hexane (1:1) yielded **5h** (9.7 mg, 51%) a colorless oil; R_f = 0.3 (EtOAc/hexane 1:1); ^{1}H NMR (400 MHz, $CDCl_3$): δ 4.31 (s, 2H), 3.02 (br s, 1H), 2.65 – 2.59 (m, 4H), 1.43 (s, 9H).

6.12 *tert*-Butyl (4*R*,5*R*,*E*)-4,5,6-trihydroxyhex-2-enoate (3i)

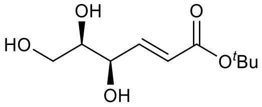

3i

The CM reaction of 3,4,5-trihydroxy-1-butene (236 mg, 2 mmol) and *tert*-butyl acrylate (**2b**, 2.9 mL, 20 mmol) was performed using *cis*-**Ru II** (80 mg, 6 mol%) with half portion of the catalyst

addition after 11 h. On complete irradiation for 30 h at 350 nm light and purification by silica gel column chromatography using ethyl acetate as the eluent, **3i** (45 mg, 10%) was obtained as a viscous oil; R_f = 0.3 (EtOAc); $[\alpha]_D^{23}$ = 10.84 (*c* 1.94, $CHCl_3$); ^1H NMR (400 MHz, $CDCl_3$): δ 6.83 (dd, *J* = 15.6, 5.0 Hz, 1H), 6.07 (dd, *J* = 15.6, 1.2 Hz, 1H), 4.39 – 4.35 (m, 1H), 3.83 – 3.66 (br s, 2H), 1.83 – 1.64 (br s, 1H), 1.48 (s, 9H); δ ^{13}C NMR (100 MHz, $CDCl_3$): δ 165.7, 144.9, 124.4, 80.9, 73.5, 71.9, 63.9, 28.1; HRMS (ESI) for $C_{10}H_{18}O_5$, m/z: $[M+Na]^+$ Calculated: 241.1048; Found: 241.1051.

6.13 (*S*)-5-((*S*)-1,2-Dihydroxyethyl)furan-2(5H)-one (4d)[12c]

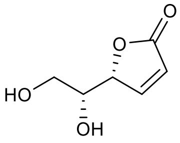

4d

The solution of **3i** (22 mg, 0.1 mmol) in DCM (10 mL) using phenanthrene (5.4 mg, 0.03 mmol) as internal UV-C filter on irradiation to 254 nm light for 45 h and purification by silica gel column chromatography using ethyl acetate as the eluent provided **4d** (8.5 mg, 59%) as a white solid. R_f = 0.2 (EtOAc); $[\alpha]_D^{23}$ = 103.2 (*c* 0.3, Acetone); ^1H NMR (400 MHz, Acetone-d6): δ 7.72 (dd, *J* = 5.8, 1.6 Hz, 1H), 6.13 (dd, *J* = 5.8, 2.1 Hz, 1H), 5.23 (ddd, *J* = 3.7, 2.1, 1.6 Hz, 1H), 3.87 (td, *J* = 6.1, 3.6 Hz, 1H), 3.66 (d, *J* = 6.2 Hz, 2H), 2.86 (br s, 2H).

6.14 *tert*-Butyl (*R*)-5,6-dihydroxy-4-oxohexanoate (5i)

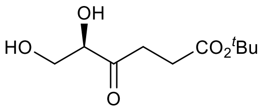

5i

From the solution of **3i** (22 mg, 0.1 mmol) in DCM (10 mL), using 0.01 mM aqueous solution of adenosine as external UV-C filter, on irradiation to 254 nm light for 5 h and purification by silica gel column chromatography using ethyl acetate/hexane (4:1) as the eluent offered **5i** (10 mg, 46%) as colorless oil. R_f = 0.5 (EtOAc); $[\alpha]_D^{23}$ = -26.67 (*c* 3.0, $CHCl_3$); ^1H NMR (400 MHz, $CDCl_3$): δ 4.29 (d, *J* = 2.6 Hz, 1H), 4.01 (d, *J* = 12.2 Hz, 1H), 3.92 (d, *J* = 12.2 Hz, 1H), 3.69 (d,

J = 6.1 Hz, 1H), 2.88 – 2.76 (m, 1H), 2.75 – 2.68 (m, 1H), 2.68 – 2.65 (m, 2H), 2.60 – 2.54 (m, 1H), 1.43 (s, 9H); ^{13}C NMR (100 MHz, $CDCl_3$): δ 209.3, 172.3, 81.4, 77.8, 63.91 32.91 29.11 28.0; HRMS (ESI) for $C_{10}H_{18}O_5$, m/z: $[M+Na]^+$ Calculated: 241.1048; Found: 241.1062; $[M+K]^+$ Calculated: 257.0786; Found: 257.1373.

6.15 Methyl (*E*)-4-((*tert*-butoxycarbonyl)amino)tridec-2-enoate (S3)

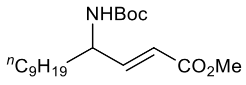

S3

The CM reaction of **1n** (566 mg, 2 mmol) and methyl acrylate (**1a**, 1.8 mL, 20 mmol) was performed in the presence of Hoveyda-Grubbs 2nd generation catalyst (38 mg, 0.6 mmol) in toluene (40 mL) under reflux for overnight. After evaporation of the volatiles on the rotatory evaporator, the residue obtained was purified by silica gel column chromatography using EtOAc/hexane (1:9) as the eluent to obtain **S3** (490 mg, 72%) as a white solid. Mp 42–43 $^{\circ}$C; R_f = 0.3 (EtOAc/hexane 1:9); ^{1}H NMR (400 MHz, $CDCl_3$): δ 6.84 (dd, *J* = 15.6, 6.3 Hz, 1H), 5.91 (dd, *J* = 15.7, 1.6 Hz, 1H), 4.51 (d, *J* = 8.0 Hz, 1H), 4.26 (br s, 1H), 3.73 (s, 3H), 1.69 – 1.49 (m, 2H), 1.43 (s, 9H), 1.36 – 1.28 (m, 4H), 1.27– 1.23 (m, 10H), 0.87 (t, *J* = 6.9 Hz, 3H); ^{13}C NMR (100 MHz, $CDCl_3$): δ 166.8, 155.1, 149.0, 120.1, 79.7, 51.6, 51.5, 34.6, 31.8, 29.5, 29.4, 29.3, 28.3, 25.6, 22.6, 14.1; HRMS (ESI) for $C_{19}H_{35}NO_4$, m/z: $[M+Na]^+$ Calculated: 364.2458; Found: 364.2457.

6.16 Methyl (*E*)-4-aminotridec-2-enoate (3t)

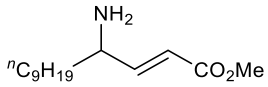

3t

To the solution of **S3** (340 mg, 1 mmol) in acetone (5 mL), 3 N aqueous HCl (5 mL) was added and the solution was stirred at room temperature for overnight. Acetone was evaporated and the residue was washed with diethyl ether. It was neutralized with saturated aqueous $NaHCO_3$ solution and extracted with diethyl ether (2 × 15 mL). Solvent was evaporated to get **3t** (225 mg, 93%) as a yellow oil; R_f = 0.2 (MeOH/DCM 1:19); ^{1}H NMR (500 MHz, $CDCl_3$): δ 6.90 (dd, *J* = 15.7, 6.6 Hz, 1H), 5.91 (dd, *J* = 15.7, 1.4 Hz, 1H), 3.74 (s, 3H), 3.45 (qd, *J* = 6.6, 1.3 Hz, 1H), 1.59 – 1.40 (m, 2H), 1.40 – 1.32 (m, 2H), 1.31 – 1.23 (m, 14H), 0.88 (t, *J* = 7.0 Hz, 3H); ^{13}C

NMR (125 MHz, $CDCl_3$) δ: 167.1, 153.0, 119.2, 52.9, 52.8, 51.6, 51.5, 37.1, 31.9, 29.5, 29.3, 25.9, 22.7, 14.1; HRMS (ESI) for $C_{14}H_{27}NO_2$, m/z: $[M+H]^+$ Calculated: 242.2115; Found: 242.2109.

6.17 5-Nonyl-1,5-dihydro-2H-pyrrol-2-one (4n)

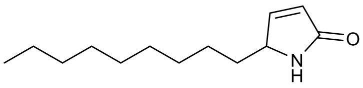

4n

From **3t** (24 mg, 0.1 mmol) using phenanthrene (17.8 mg, 0.1 mmol) as internal UV-C filter on irradiation at 254 nm for 24 h, and purification by silica gel column chromatography using MeOH/DCM (1:49), gave **4n** (12 mg, 57%) as a white solid; Mp 44–46 °C; R_f = 0.2 (MeOH/DCM 1:49); ^{1}H NMR (400 MHz, $CDCl_3$) δ: 7.04 (dt, *J* = 5.8, 1.7 Hz, 1H), 6.53 (br s, 1H), 6.07 (dt, *J* = 5.8, 1.7 Hz, 1H), 4.17 (ddq, *J* = 7.6, 6.0, 1.6 Hz, 1H), 1.66 – 1.46 (m, 2H), 1.36 – 1.20 (m, 14H), 0.86 (t, *J* = 6.9 Hz, 3H); ^{13}C NMR (100 MHz, $CDCl_3$) δ: 172.9, 149.4, 125.9, 58.8, 32.1, 30.8, 28.5, 28.4, 28.2, 25.0, 21.6, 13.1; HRMS (ESI) for $C_{13}H_{23}NO$, m/z: $[M+Na]^+$ Calculated: 232.1672, Found: 232.1664.

6.18 (*E*)-4-Hydroxy-4-phenylbut-2-enamide (3u)

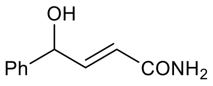

3u

CM reaction of **1a** (27 mg, 0.2 mmol) and acrylamide (**2f**, 36 mg, 0.5 mmol) in DCM (8 mL) was carried out at 350 nm using *cis*-**Ru II** (8.2 mg, 0.012 mmol) as a catalyst; half portions of which is added after 2 h. On total irradiation of 5 h and purification by silica gel column chromatography using MeOH/DCM (1:9) as the eluent, **3u** was obtained as a white solid (20 mg, 55%); Mp 84–86 °C; R_f = 0.2 (MeOH/DCM 1:9); ^{1}H NMR (400 MHz, $CDCl_3$): δ 7.42 – 7.27 (m, 5H), 6.99 (dd, *J* = 15.3, 4.5 Hz, 1H), 6.20 (dd, *J* = 15.3, 1.8 Hz, 1H), 5.45 (br s, 2H), 5.40 – 5.38 (m, 1H), 2.12 (d, *J* = 3.7 Hz, 1H); ^{13}C NMR (100 MHz, $(CD_3)_2CO$): δ 166.7, 145.4, 143.1, 128.2, 127.1, 126.2, 122.1, 72.5; HRMS (ESI) for $C_{10}H_{11}NO_2$, m/z: $[M+H]^+$ Calculated: 178.0863; Found: 178.0850; $[M+Na]^+$ Calculated: 200.0682; Found: 200.0668.

6.19 4-Oxo-4-phenylbutanamide (5p)[13a]

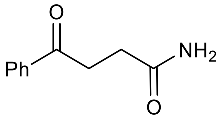

5p

0.01 M solution of **3u** (17 mg, 0.1 mmol) in 10 mL ^{t}BuOH/DCM (1:4) upon irradiation for 5 h at 254 nm and purification of crude product by silica gel column chromatography using MeOH/DCM (1:19) as the eluent gave **5p** (14 mg, 79%) as a white solid; Mp 116–118 °C; R_f = 0.22 (MeOH/DCM 1:19); ^{1}H NMR (400 MHz, $CDCl_3$) δ: 7.99 (d, *J* = 7.5 Hz, 2H), 7.58 (t, *J* = 7.4 Hz, 1H), 7.47 (t, *J* = 7.6 Hz, 2H), 5.73 (br s, 1H), 5.37 (br s, 1H), 3.39 (t, *J* = 6.5 Hz, 2H), 2.68 (t, *J* = 6.5 Hz, 2H); GC-MS (EI) for $C_{10}H_{11}NO_2$, M^+ Calculated: 177.2, Found: 177.1.

6.20 (*E*)-4-Hydroxy-*N*-isopropyl-4-phenylbut-2-enamide (3v)

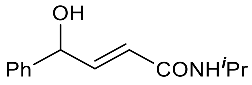

3v

CM reaction of **1a** (27 mg, 0.2 mmol) and *N*-isopropyl acrylamide (**2g**, 57 mg, 0.5 mmol) was carried out in the same manner as described above for **3u** using *cis*-**Ru II** (8.2 mg, 0.012 mmol) as a catalyst in DCM (8 mL). After 5 h total irradiation at 350 nm followed by purification with silica gel column chromatography using MeOH/DCM (1:9) as the eluent gave **3v** (25 mg, 57%) as a white solid; Mp 128–130 °C; R_f = 0.23 (MeOH/DCM 1:9); ^{1}H NMR (400 MHz, $CDCl_3$): δ 7.42 – 7.28 (m, 5H), 6.94 (dd, *J* = 15.2, 4.7 Hz, 1H), 6.06 (dd, *J* = 15.2, 1.5 Hz, 1H), 5.39 (br s, 1H), 5.37 (m, 1H), 4.29 – 4.01 (m, 1H), 2.22 (s, 1H), 1.17 (d, *J* = 6.6 Hz, 6H); ^{13}C NMR (100 MHz, $CDCl_3$): δ 164.5, 144.1, 141.3, 128.8, 128.3, 126.6, 123.0, 73.6, 41.5, 22.8; HRMS (ESI) for $C_{13}H_{17}NO_2$ m/z: $[M+H]^+$ Calculated: 220.1332; Found: 220.1317; $[M+Na]^+$ Calculated: 242.1152; Found: 242.1136.

6.21 *N*-Isopropyl-4-oxo-4-phenylbutanamide (5q)[13b]

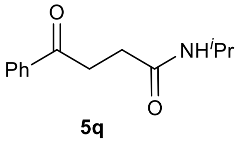

5q

0.01M solution of **3v** (22 mg, 0.1 mmol) in 10 mL ^{t}BuOH/DCM (1:4) on irradiation for 5 h at 254 nm and purification of crude product by silica gel column chromatography using

MeOH/DCM (1:19) gave **5q** (14 mg, 64%) as a white solid; Mp 119–121 °C; R_f = 0.25 (MeOH/DCM 1:19); ^{1}H NMR (400 MHz, $CDCl_3$) δ: 7.99 (d, *J* = 7.4 Hz, 2H), 7.57 (t, *J* = 7.3 Hz, 1H), 7.46 (t, *J* = 7.6 Hz, 2H), 5.55 (s, 1H), 4.06 (dq, *J* = 13.3, 6.6 Hz, 1H), 3.36 (t, *J* = 6.6 Hz, 2H), 2.57 (t, *J* = 6.6 Hz, 2H), 1.15 (d, *J* = 6.5 Hz, 6H); GC-MS (EI) for $C_{13}H_{17}NO_2$, M^+ Calculated: 219.1, Found: 219.1.

7. Synthesis of (-)-Isocladospolide-B (4o) and *tert*-Butyl (5*S*,11*R*)-5,11-dihydroxy-4-oxododecanoate (5r)

7.1. (2*R*,8*S*,9*S*)-Undec-10-ene-2,8,9-triol (1o)

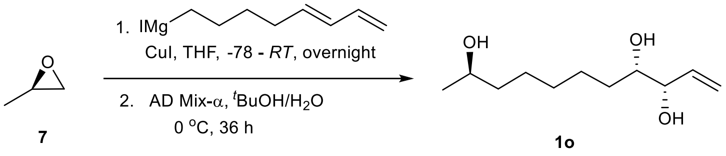

A 50 mL three neck round bottom flask equipped with magnetic stir bar a reflux condenser and addition funnel was charged with activated magnesium turnings (233 mg, 9.6 mmol). It was applied to high vacuum under heating with hot air gun and flushed with argon. The cycle was repeated twice and after cooling to room temperature, anhydrous diethyl ether (2 mL) was added to it. The addition funnel was charged with a solution of (*E*)-8-iodoocta-1,3-diene[14] (1.89 g, 8 mmol) in anhydrous diethyl ether (14 mL). 2 mL of this solution was added to the above suspension with vigorous stirring. Once the reaction was initiated, rest of the solution of (*E*)-8-iodoocta-1,3-diene was added dropwise and reaction mass was further stirred for 2 h. In a 100 mL Schlenk flask, CuI (57 mg, 0.3 mmol) was suspended in anhydrous THF (3 mL) under argon atmosphere and *R*-(+)-propylene oxide (**7**, 0.21 mL, 3 mmol) was added to it. The suspension was cooled to -78 °C, and above solution of Grignard reagent was dropwise added to it. The reaction mass was allowed to warm-up slowly, and the resulting purple reaction mass was stirred overnight at room temperature. It was then cooled to 0 °C and quenched with saturated aqueous NH_4Cl solution. Organic layer was separated and the aqueous phase was extracted with diethyl ether (2 × 20 mL). Combined organic extract was washed with brine and dried over anhydrous $MgSO_4$. It was filtered and concentrated to get the crude product, which was filtered through a short column of silica gel, washing it with hexane to EtOAc/hexane (1:9) to get the product as colorless oil. This oil was dissolved in a mixture of ^{t}BuOH (40 mL) and water (40 mL) and the solution was cooled to 0 °C. Methane sulfonamide (380 mg, 4 mmol) and AD-mix-α (4.7 g,

containing 0.07 mol% osmium) was added to it. The orange suspension was stirred vigorously at 0 °C for 36 h. Sodium metabisulfite (6 g) was added slowly to it and ^{t}BuOH was carefully removed under reduced pressure. The residue was extracted with ethyl acetate (3 × 20 mL); the combined organic extract was washed with brine (20 mL) and dried over anhydrous magnesium sulfate. It was concentrated to get crude product which was purified by silica gel column chromatography using EtOAc as the eluent to afford triol **1o** as colorless oil (320 mg, 53%). $[\alpha]^{23}_{D}$= -24.75 (*c* 6.87, $CHCl_3$); R_f = 0.2 (EtOAc); ^{1}H NMR (400 MHz, $CDCl_3$) δ 5.97 – 5.71 (m, 1H), 5.35 (dt, *J* = 17.2, 1.4 Hz, 1H), 5.25 (dt, *J* = 10.5, 1.2 Hz, 1H), 3.93 (t, *J* = 6.2 Hz, 1H), 3.83 – 3.74 (m, 1H), 3.53 – 3.43 (m, 1H), 2.30 (br s, 1H), 1.68 (br s, 1H), 1.56 – 1.29 (m, 11H), 1.18 (dd, *J* = 6.2, 1.3 Hz, 3H); ^{13}C NMR (100 MHz, $CDCl_3$) δ: 137.6, 117.4, 76.2, 74.3, 68.1, 39.2, 32.7, 29.5, 25.6, 25.5, 23.5; HRMS (ESI) for $C_{11}H_{22}O_3$ m/z: $[M+Na]^+$ Calculated: 225.1461; Found: 225.1455.

7.2. *tert*-Butyl (4*S*,5*S*,11*R*)-4,5,11-trihydroxydodec-2-enoate (3w)

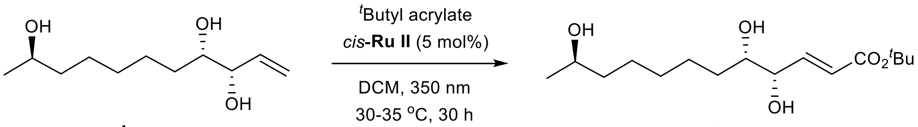

1o 3w

Following the general procedure for CM reaction, the solution of **1o** (81 mg, 0.4 mmol), *tert*-butyl acrylate (**2b**, 513 mg, 4 mmol) and *cis*-**Ru II** (8 mg, 0.012 mmol) in anhydrous dichloromethane (16 mL) was irradiated with 350 nm light at 30-35 °C, in the Rayonet with magnetic stirring. The progress of reaction was monitored by ^{1}H NMR. After 10 h, argon was flushed through the reaction mass and *cis*-**Ru II** (5.3 mg, 0.008 mmol) was added to it. After further irradiation for 20 h at 350 nm; volatiles were removed on the rotavapor and the crude product obtained was purified by silica gel column chromatography using EtOAc/hexane (2:1) as the eluent to get **3w** as an oil (102 mg, 84%) having *E* and *Z* isomers (97:3). $[\alpha]^{23}_{D}$= -21.28 (*c* 8.93, $CHCl_3$); R_f = 0.2 (EtOAc/hexane 2:1); ^{1}H NMR (400 MHz, $CDCl_3$): δ 6.81 (dd, *J* = 15.6, 5.2 Hz, 1H), 6.04 (dd, *J* = 15.6, 1.6 Hz, 1H), 4.08 (dd, *J* = 10.4, 5.2 Hz, 1H), 3.82 – 3.74 (m, 1H), 3.53 (dt, *J* = 9.2, 4.6 Hz, 1H), 2.81 (br s, 1H), 2.54 (s, 1H), 1.77 (br s, 1H), 1.56 – 1.49 (m, 2H), 1.48 (s, 9H), 1.46 – 1.29 (m, 8H), 1.17 (d, *J* = 6.2 Hz, 3H); ^{13}C NMR (100 MHz, $CDCl_3$): δ 165.7, 145.6, 124.3, 80.8, 74.1, 73.9, 68.1, 39.1, 32.9, 29.4, 28.1, 25.5, 25.5, 23.5; HRMS (ESI)

for $C_{16}H_{30}O_5$ m/z: $[M+H]^+$ Calculated: 303.2166, Found: 303.2162; $[M+Na]^+$ Calculated: 325.1985, Found: 325.1977.

7.3. (-)-Iso-cladospolide B (4o)[15]

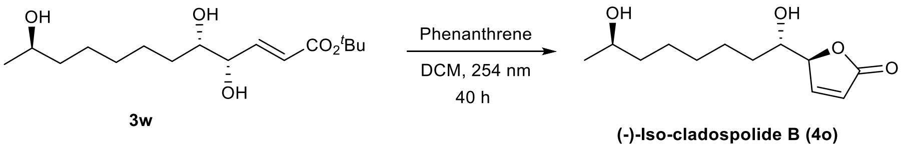

Using the general procedure for lactonization, the solution of **3w** (0.1 mmol, 30 mg) and phenanthrene (5.34 mg, 0.03 mmol) in DCM (10 mL) was irradiated with 254 nm light under slow stirring, monitoring the reaction with TLC. After completion of the reaction (40 h), the solvent was removed on the rotavapor and the crude product was purified by silica gel column chromatography using EtOAC/hexane (4:1) as the eluent, to get **(-)-Iso-cladospolide B** (**4o**) as off-white solid (14 mg, 61%). $[\alpha]_D^{23}$ = -64.0 (*c* 5.0, MeOH), Lit.[15] $[\alpha]_D^{25}$ = -73.28 (*c* 1.0, MeOH); R_f = 0.4 (EtOAc); ^{1}H NMR (400 MHz, $CDCl_3$): δ 7.45 (dd, *J* = 5.8, 1.6 Hz, 1H), 6.19 (dd, *J* = 5.8, 2.1 Hz, 1H), 5.00 – 4.96 (m, 1H), 3.83 – 3.71 (m, 2H), 2.08 (br s, 1H), 1.64 – 1.51 (m, 3H), 1.48 – 1.32 (m, 8H), 1.19 (d, *J* = 6.2 Hz, 3H); ^{13}C NMR (100 MHz, $CDCl_3$): δ 172.8, 153.6, 122.8, 86.1, 71.8, 68.1, 39.1, 33.1, 29.3, 25.6, 25.4, 23.6; HRMS (ESI) for $C_{12}H_{20}O_4$ m/z: $[M+H]^+$ Calculated: 229.1434, Found: 229.1432; $[M+Na]^+$ Calculated: 251.1254, Found: 251.1251.

7.4. *tert*-Butyl (5*S*,11*R*)-5,11-dihydroxy-4-oxododecanoate (5r)

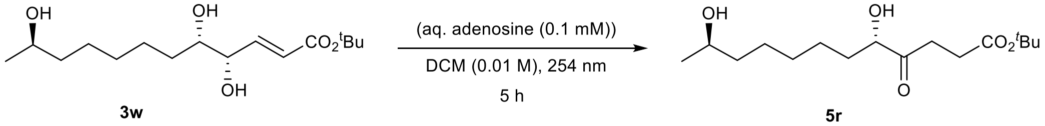

According the procedure for photoisomerization, the solution of **3w** (0.1 mmol, 30 mg) in DCM (10 mL) was irradiated with 0.01 mM aqueous adenosine solution as external UV-C filter. After completion of the reaction (5 h), solvent was evaporated and the crude product was purified by silica gel column chromatography, eluting it with ethyl actetate/hexane (1:1), to get **5r** as colorless oil (16 mg, 53%). $[\alpha]_D^{23}$= 68.62 (*c* 1.60, $CHCl_3$); R_f = 0.2 (EtOAc/hexane 1:1); ^{1}H NMR (400 MHz, $CDCl_3$): δ 4.25 – 4.20 (m, 1H), 3.79 (d, *J* = 4.3 Hz, 1H), 3.41 (d, *J* = 4.7 Hz, 1H), 2.79 – 2.63 (m, 2H), 2.62 – 2.55 (m, 2H), 1.67 – 1.45 (m, 4H), 1.43 (s, 9H), 1.42 –1.26 (m,

6H), 1.18 (d, J = 6.2 Hz, 4H); ^{13}C NMR (100 MHz, $CDCl_3$): δ 210.9, 171.6, 81.0, 76.5, 68.0, 39.1, 33.6, 32.6, 29.3, 29.1, 28.0, 25.5, 24.7, 23.5; HRMS (ESI) for $C_{16}H_{30}O_5$ m/z: $[M+Na]^+$ Calculated: 325.1985, Found: 325.1984.

8. Synthesis of (±)-1o (racemic)

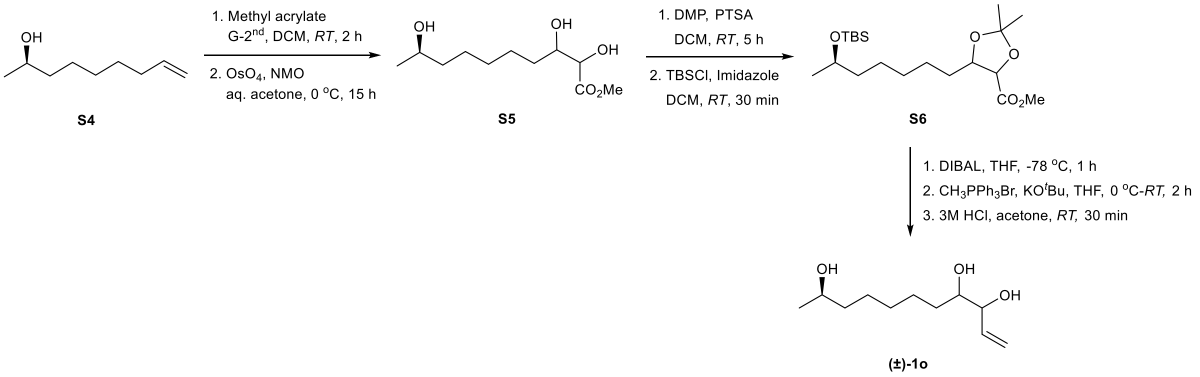

To the solution of **S4**[16a] (853 mg, 6 mmol) and methyl acrylate (5.44 mL, 60 mmol) in anhydrous DCM (240 mL); Grubb's IInd generation catalyst (0.18 mmol, 150 mg) was added under argon atmosphere and the solution was stirred at room temperature. After completion of the reaction (2 h), solvent was evaporated and the crude product was purified by column chromatography using EtOAc/hexane (1:3) as the eluent to get CM-product[16b] as viscous oil. This was dissolved in acetone (29 mL) and water (7.2 mL). It was cooled to 0 °C and NMO (1.1 g, 12 mmol) and 4% aqueous OsO_4 (1.5 mL) and stirred at same temperature for 15 h. The reaction mass was poured into saturated aqueous sodium sulfite (30 mL), acetone was evaporated on rotavapor and the product was extracted with ethyl acetate (3 × 30 mL). Combined organic extract was washed with brine (30 mL) and dried over $MgSO_4$ and concentrated to get crude product which was purified by silica gel column chromatography using EtOAc/hexane (1:1) as the eluent to get triol **S5** as viscous oil (940 mg, 67%). R_f = 0.2 (EtOAc/hexane 1:1); ^{1}H NMR (400 MHz, $CDCl_3$): δ 4.10 (d, J = 2.1 Hz, 1H), 3.91 – 3.86 (m, 1H), 3.83 (s, 3H), 3.82 – 3.71 (m, 1H), 1.65 – 1.58 (m, 2H), 1.55 – 1.28 (m, 9H), 1.19 (d, J = 6.2 Hz, 3H); ^{13}C NMR (100 MHz, $CDCl_3$): δ 174.1, 73.0, 72.4, 68.1, 52.9, 39.2, 33.6, 29.4, 25.6, 23.5. HRMS (ESI) for $C_{11}H_{22}O_5$ m/z: $[M+Na]^+$ Calculated: 257.1359, Found: 257.1364.

To the solution of **S5** (473 mg, 2 mmol) in anhydrous DCM (4 mL) dimethoxy propane (0.25 mL, 2.1 mmol) and PTSA (17 mg, 0.1 mmol) was added at room temperature. After stirring for 4 h; the reaction mass was washed with water (10 mL) and brine (10 mL). Organic phase was dried over anhydrous $MgSO_4$ and concentrated on rotavapor. It was dissolved in

anhydrous DCM (4 mL) and TBSCl (301 mg, 2 mmol) and Imidazole (150 mg, 2.2 mmol) was added to it. After stirring for 30 min; solvent was evaporated and the crude product was purified by column chromatography using EtOAc/hexane (1:19) as the eluent to afford **S6** (545 mg, 70%) as a colorless oil. R_f = 0.2 (EtOAc/hexane 1:19); ^{1}H NMR (400 MHz, $CDCl_3$): δ 4.15 – 4.06 (m, 2H), 3.78 (s, 3H), 3.78 – 3.74 (m, 1H), 1.82 – 1.58 (m, 2H), 1.53 – 1.47 (m, 1H), 1.45 (d, J = 9.7 Hz, 6H), 1.41 – 1.23 (m, 6H), 1.11 (d, J = 6.1 Hz, 3H), 0.91 (s, 1H), 0.88 (s, 9H), 0.04 (d, J = 1.6 Hz, 6H); ^{13}C NMR (100 MHz, $CDCl_3$): δ 171.4, 110.8, 79.2, 79.0, 68.6, 52.3, 39.6, 33.4, 29.6, 27.2, 25.9, 25.7, 23.8, 18.2, -4.4, -4.7; HRMS (ESI) for $C_{20}H_{40}O_5Si$ m/z: $[M+Na]^+$ Calculated: 411.2537, Found: 411.2554.

The solution of **S6** (390 mg, 1 mmol) in anhydrous THF (3 mL) was cooled to -78 °C and treated with DIBAL (1.5 mL, 1.0 M in toluene). After stirring at the same temperature for 1 h, the reaction was quenched with aqueous sodium potassium tartarate solution. The residue was filtered through the buckner funnel and filtrate was extracted with diethyl ether (3 × 15 mL). It was dried over anhydrous $MgSO_4$ and concentrated to get crude aldehyde. A 25 mL Schlenk flask was charged with methyl triphenyl phosphonium bromide (430 mg, 1.2 mmol) and potassium ^{t}butoxide (170 mg, 1.5 mmol) under nitrogen atmosphere. The flask was cooled to 0 °C and anhydrous THF (5 mL) was added to it and the yellow suspension was vigorously stirred for 30 min. The solution of above crude aldehyde in THF (3 mL) was added dropwise and after stirring for 20 min at 0 °C, the reaction mass was brought to room temperature. After completion of the reaction (2 h) as indicated by TLC, it was quenched with saturated aqueous NH_4Cl and extracted with diethyl ether (3 × 15 mL). The combined organic extract was dried over anhydrous $MgSO_4$ and concentrated to get crude product. It was dissolved in acetone (5 mL) and treated with 3 M HCl (3 mL). After stirring for 1 h, acetone was evaporated and the reaction mass was extracted with ethyl acetate (3 × 15 mL). The organic extract was washed with brine, dried over anhydrous $MgSO_4$ and concentrated to get crude product. This on purification by silica gel column chromatography using ethyl acetate as the eluent gave the required **(±)-1o** as a colorless oil (120 mg, 60%). R_f = 0.2 (EtOAc); ^{1}H NMR (400 MHz, $CDCl_3$): δ 5.95 – 5.80 (m, 1H), 5.36 (dt, J = 17.2, 1.4 Hz, 1H), 5.28 – 5.19 (m, 1H), 3.93 (tt, J = 6.2, 1.1 Hz, 1H), 3.83 – 3.74 (m, 1H), 3.48 (ddd, J = 9.3, 6.0, 3.2 Hz, 1H), 2.18 (t, J = 14.4 Hz, 1H), 1.56 – 1.30 (m, 12H), 1.18 (d, J = 6.2 Hz, 3H).

9. NMR spectra

^{1}H NMR (400 MHz, $CDCl_3$) of **1a-D**

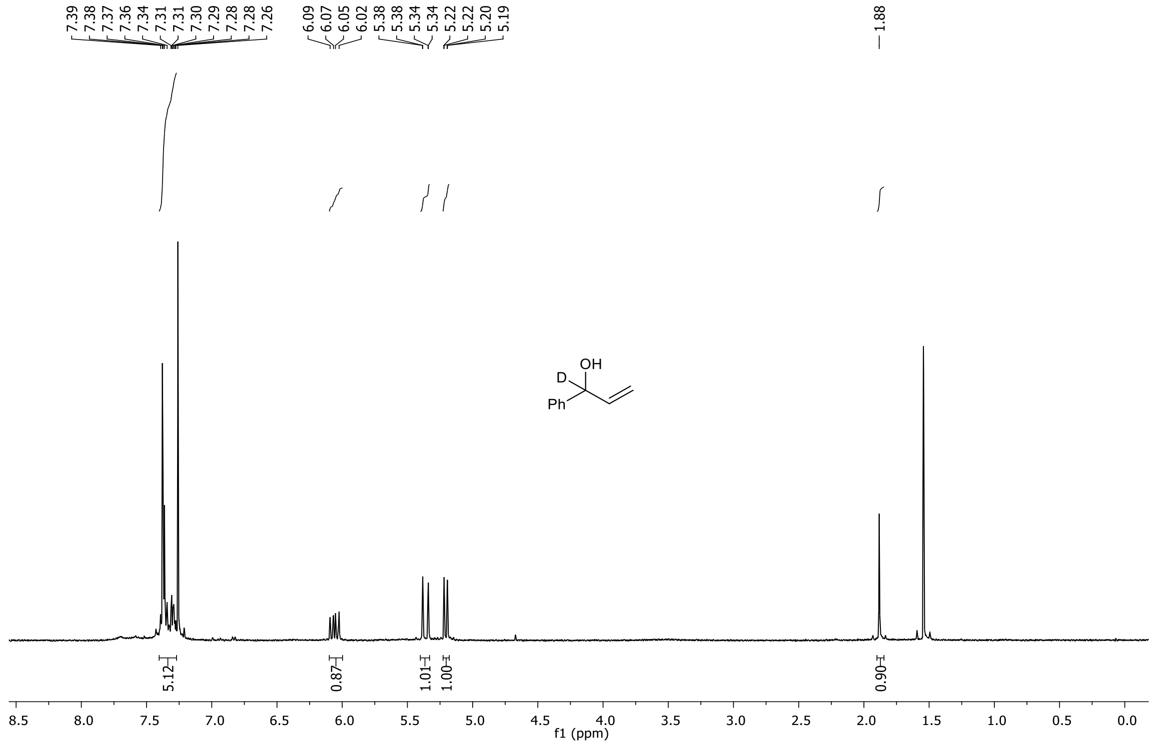

^{1}H NMR (400 MHz, $CDCl_3$) of **S1**

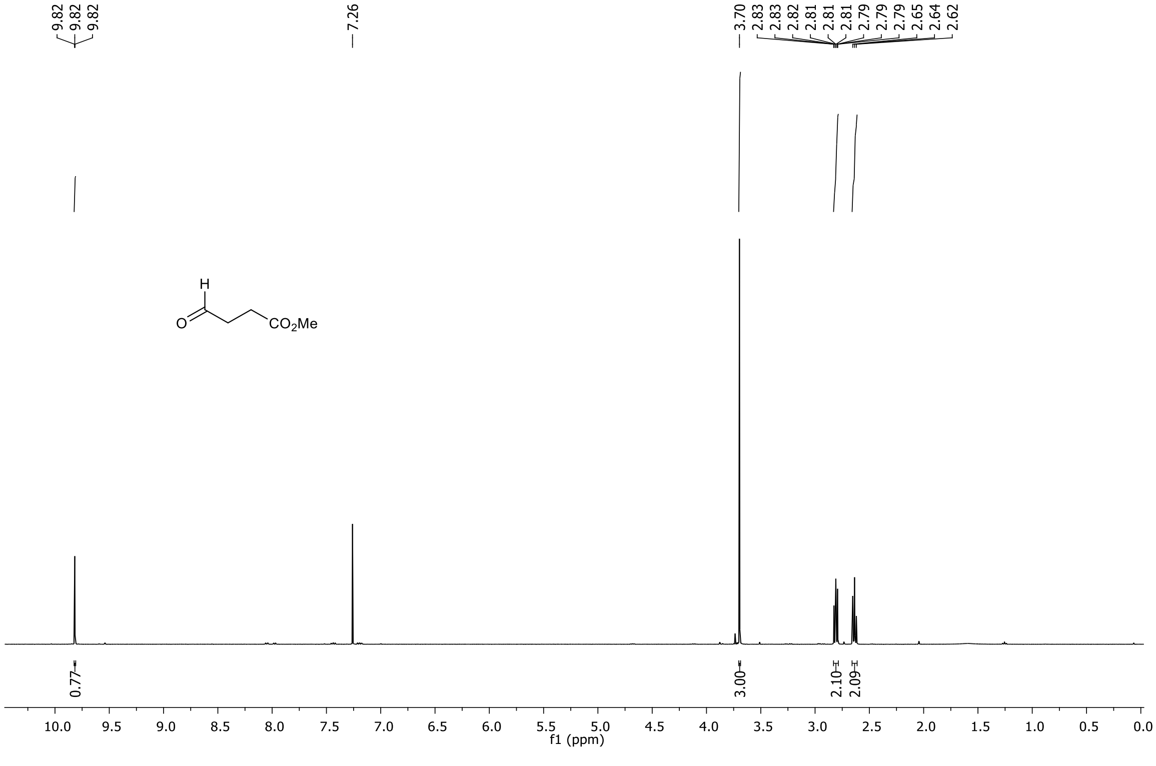

^{1}H NMR (400 MHz, $CDCl_3$) of **1e**

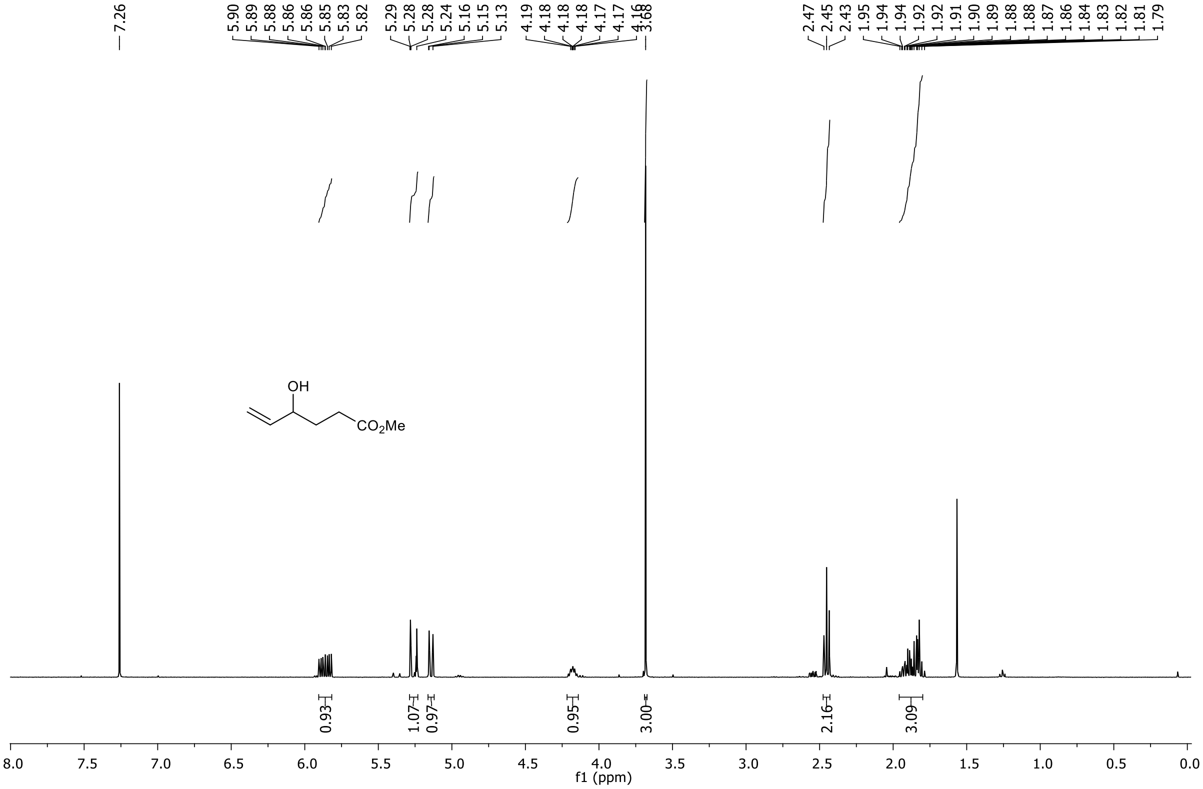

^{1}H NMR (400 MHz, $CDCl_3$) of **S2**

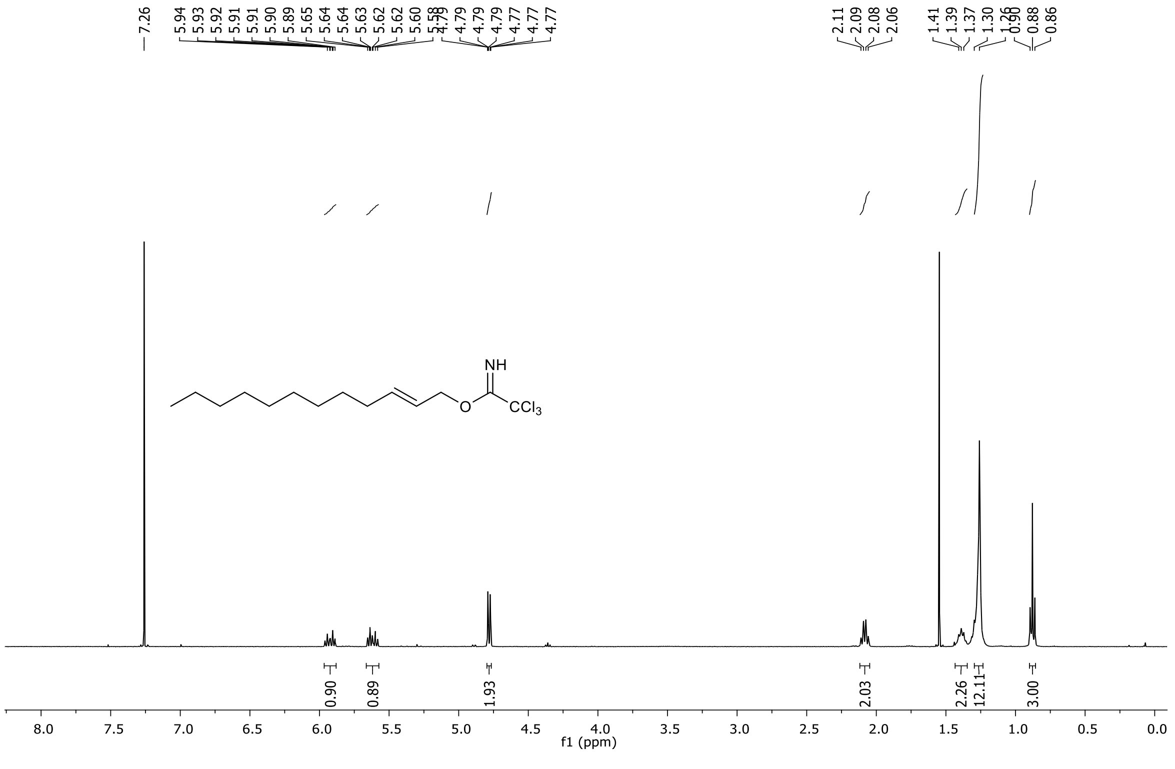

^{13}C NMR (100 MHz, $CDCl_3$) of **S2**

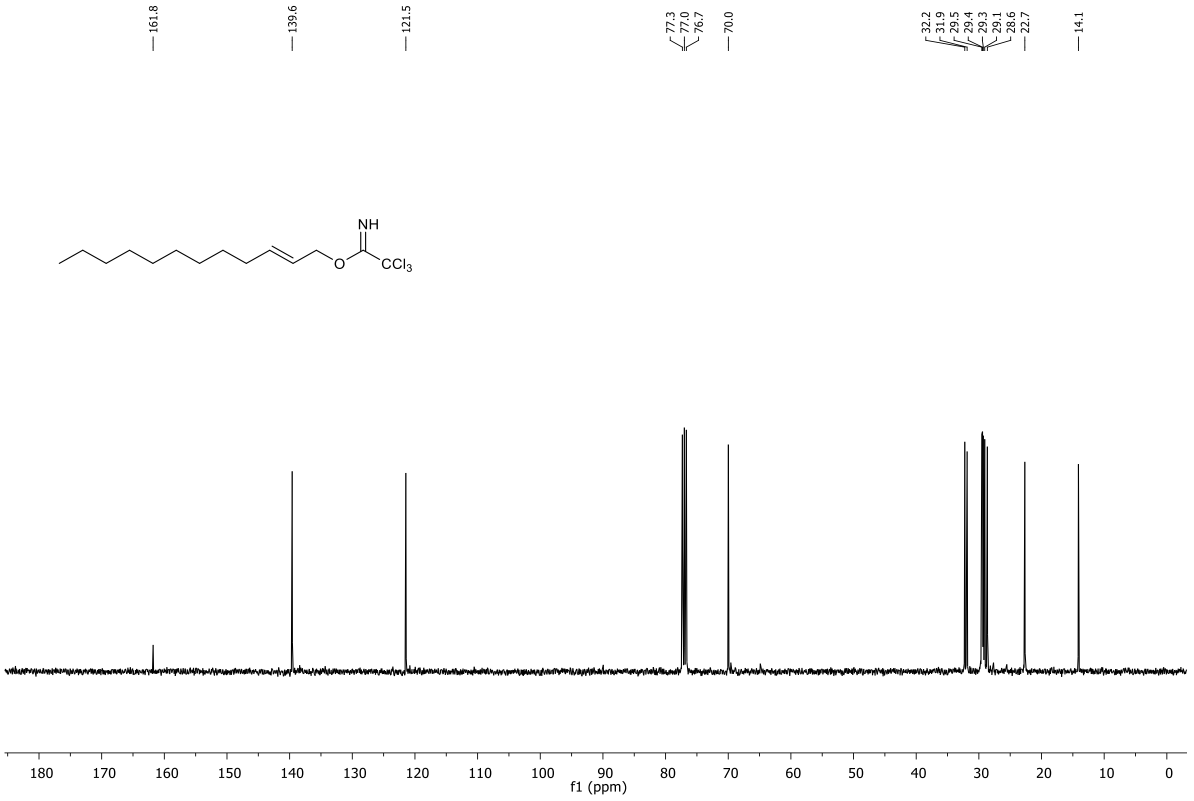

^{1}H NMR (400 MHz, $CDCl_3$) of **2,2,2-trichloro-N-(dodec-1-en-3-yl)acetamide**

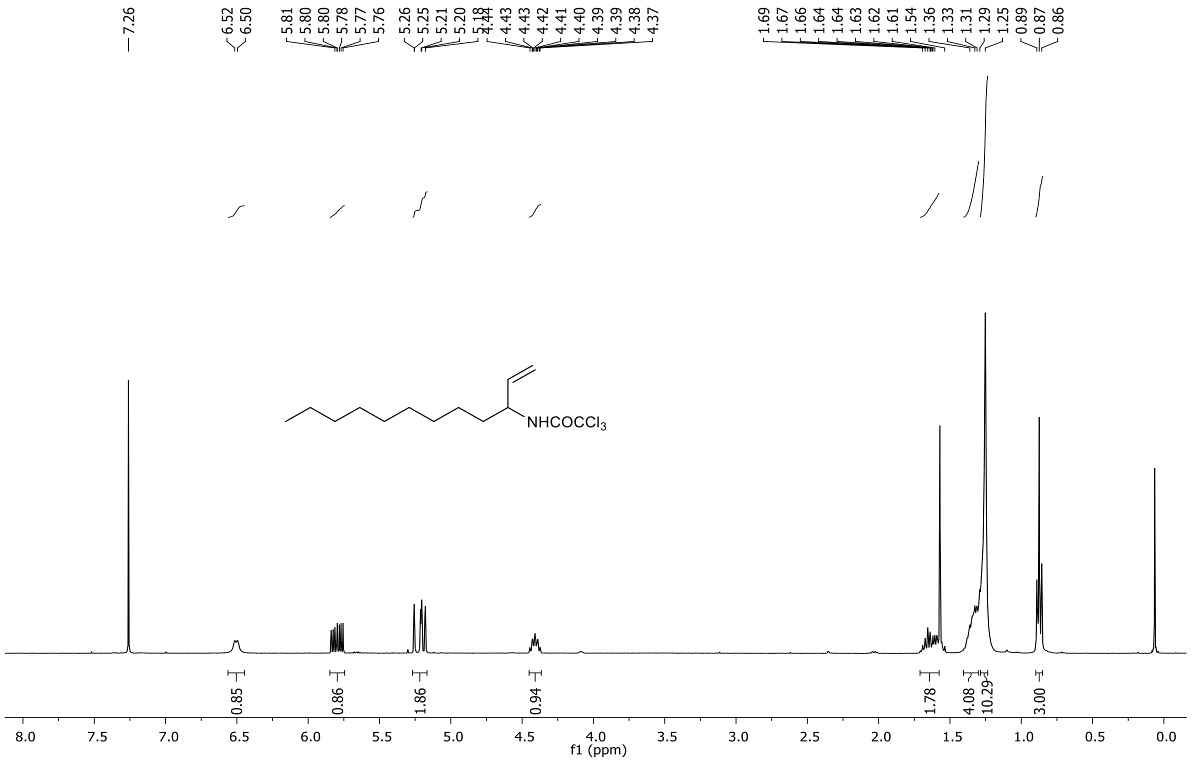

^{13}C NMR (100 MHz, $CDCl_3$) of **2,2,2-trichloro-N-(dodec-1-en-3-yl)acetamide**

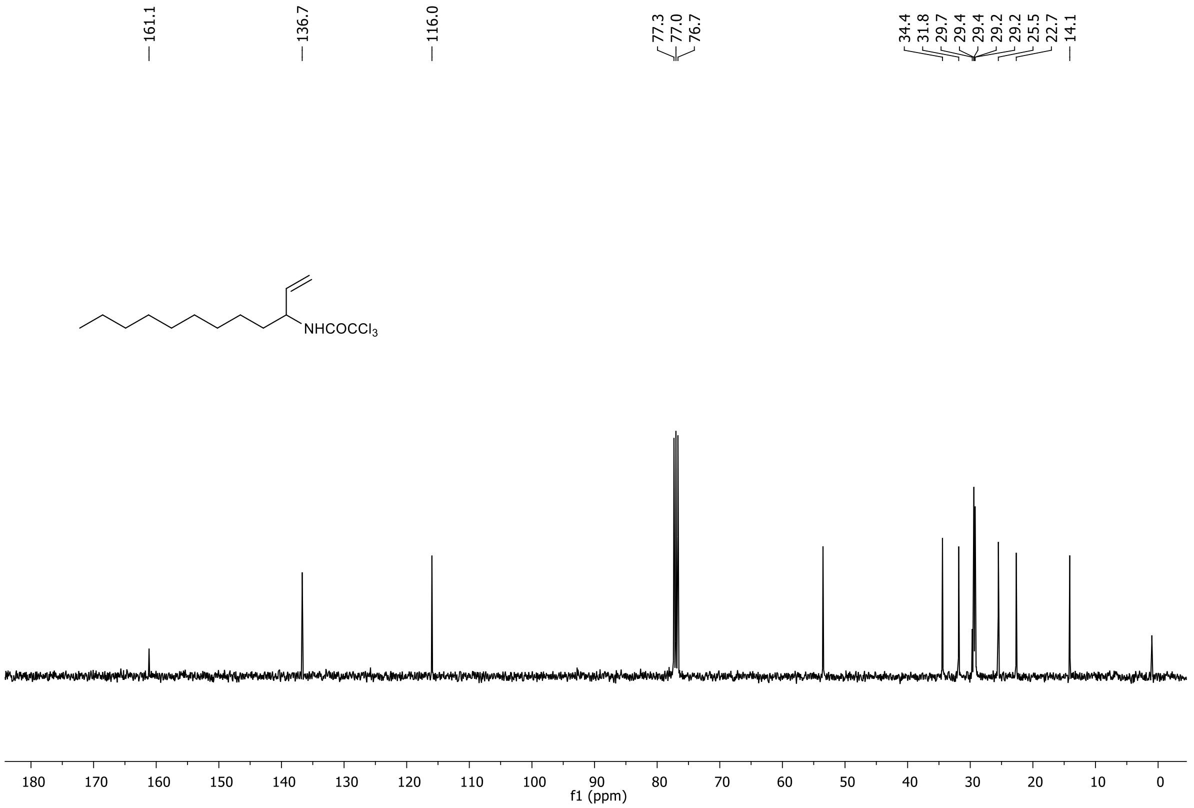

^{1}H NMR (500 MHz, $CDCl_3$) of **1n**

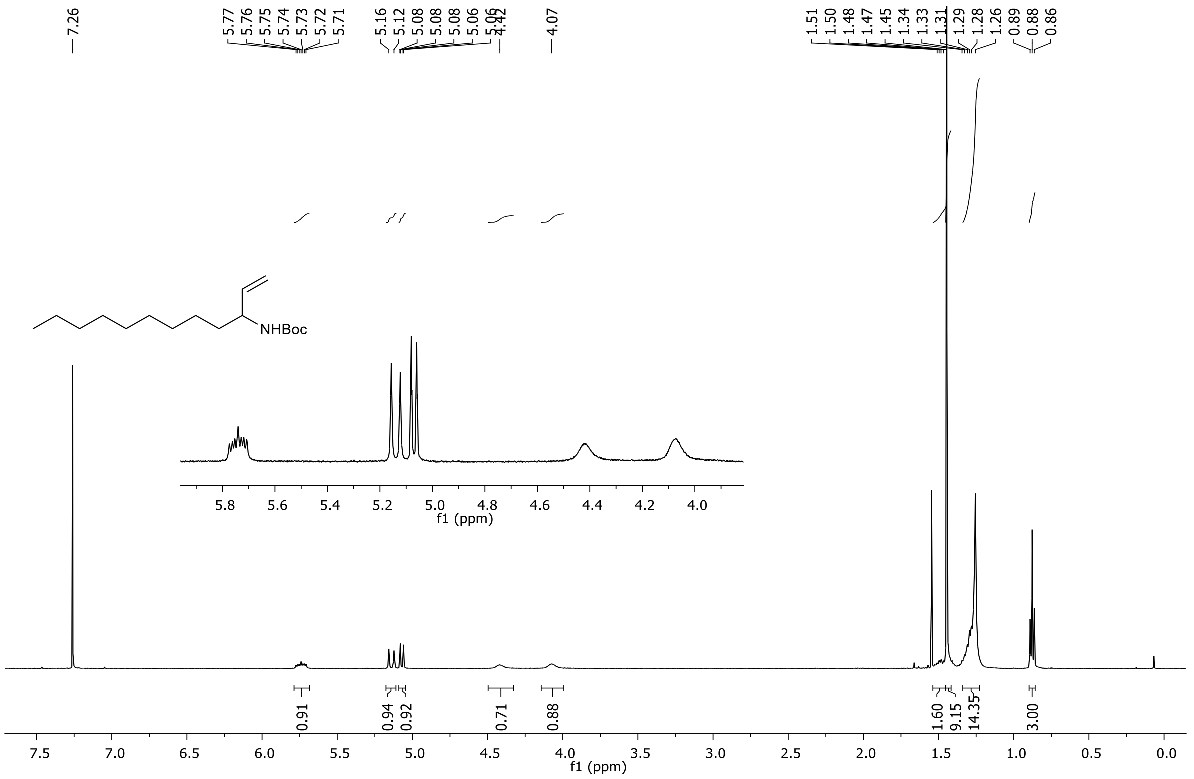

^{13}C NMR (125 MHz, $CDCl_3$) of **1n**

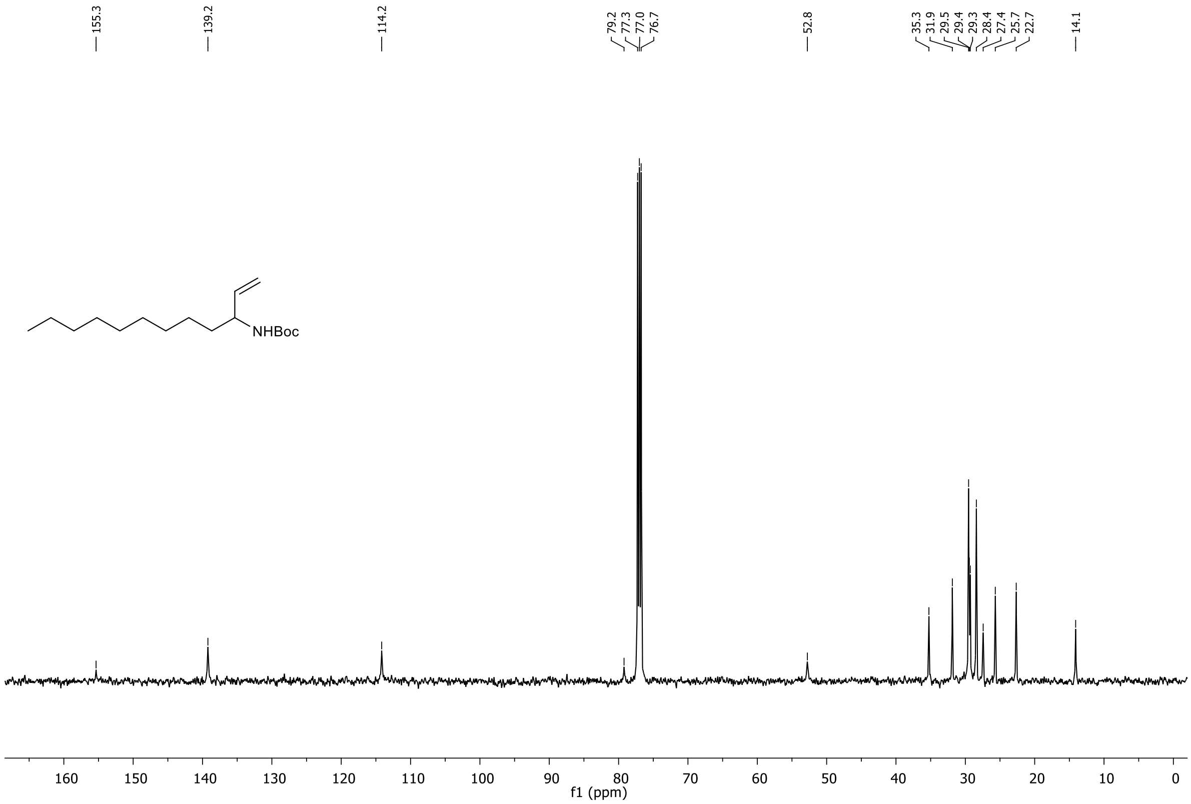

^{1}H NMR (400 MHz, $CDCl_3$) of **3b-D**

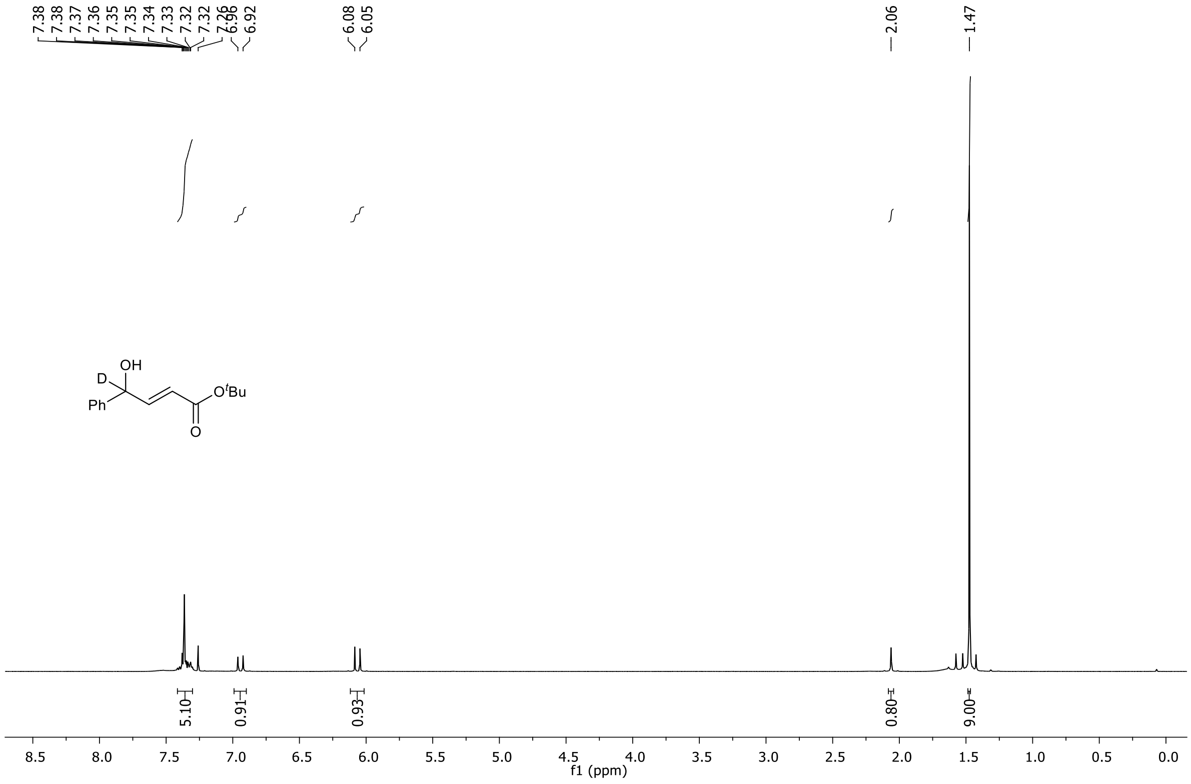

^{13}C NMR (100 MHz, $CDCl_3$) of **3b-D**

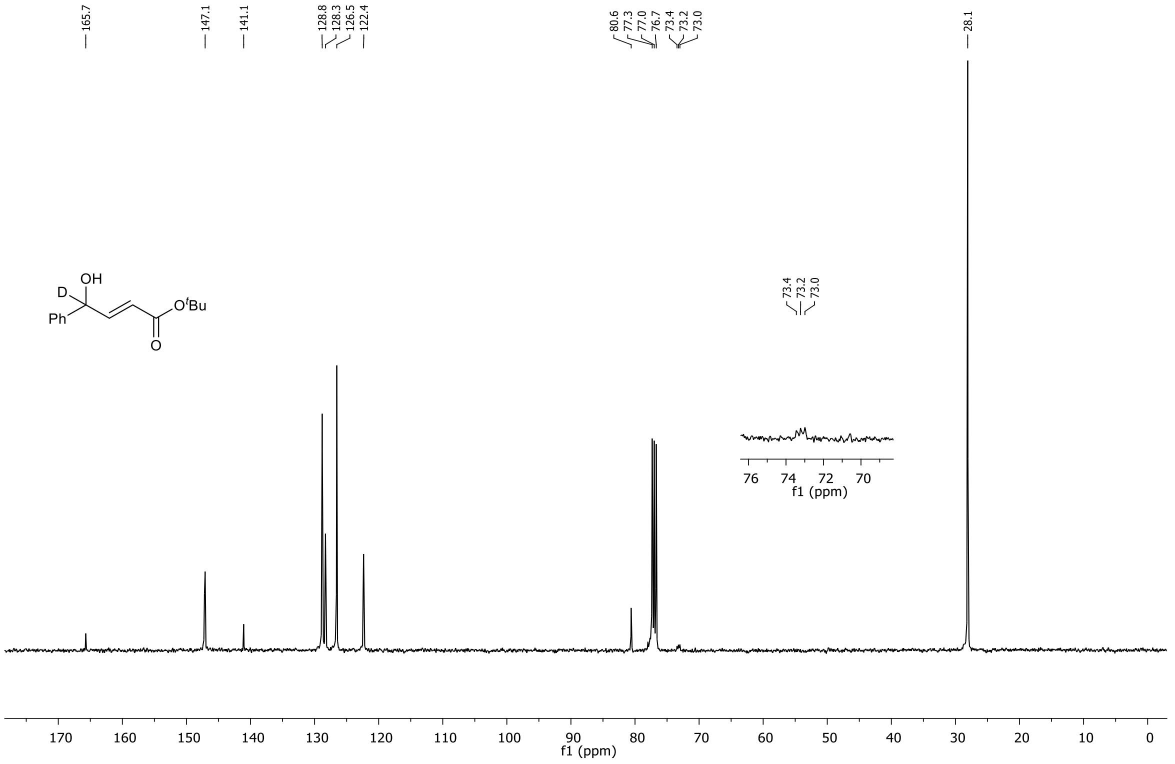

^{1}H NMR (400 MHz, $CDCl_3$) of **3c**

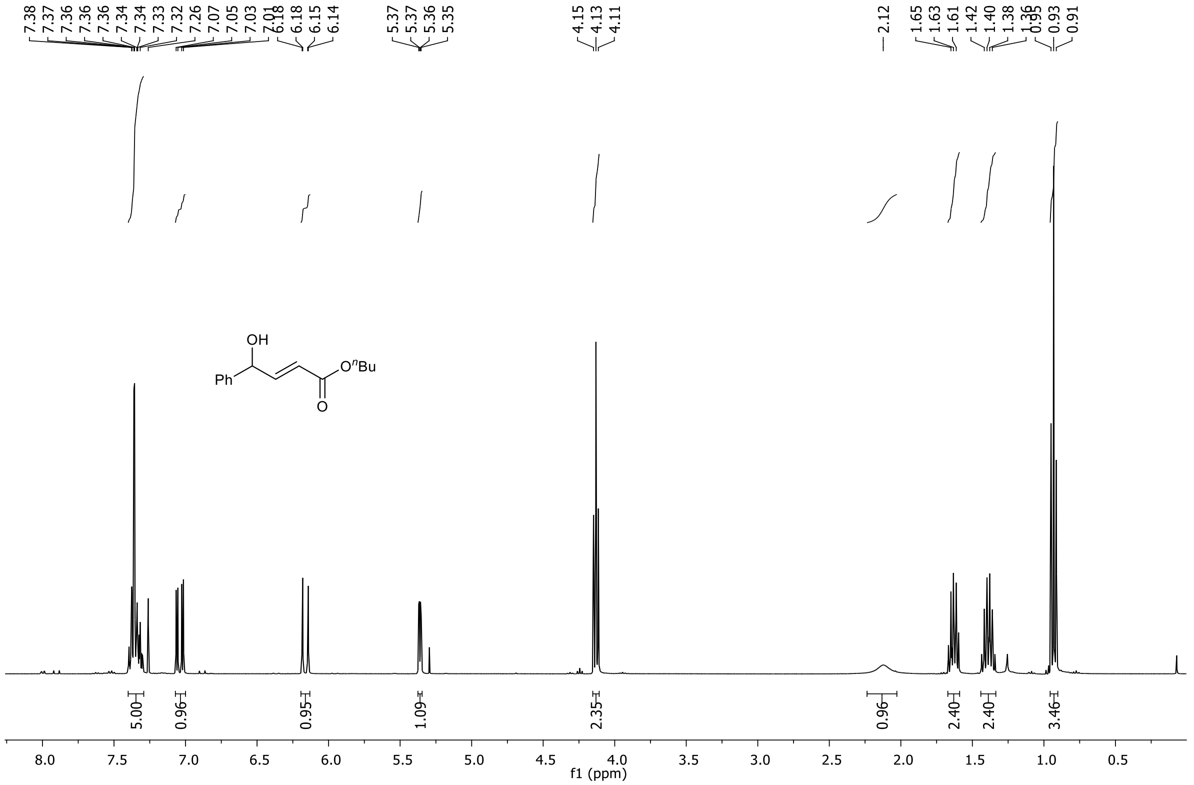

^{13}C NMR (100 MHz, $CDCl_3$) of **3c**

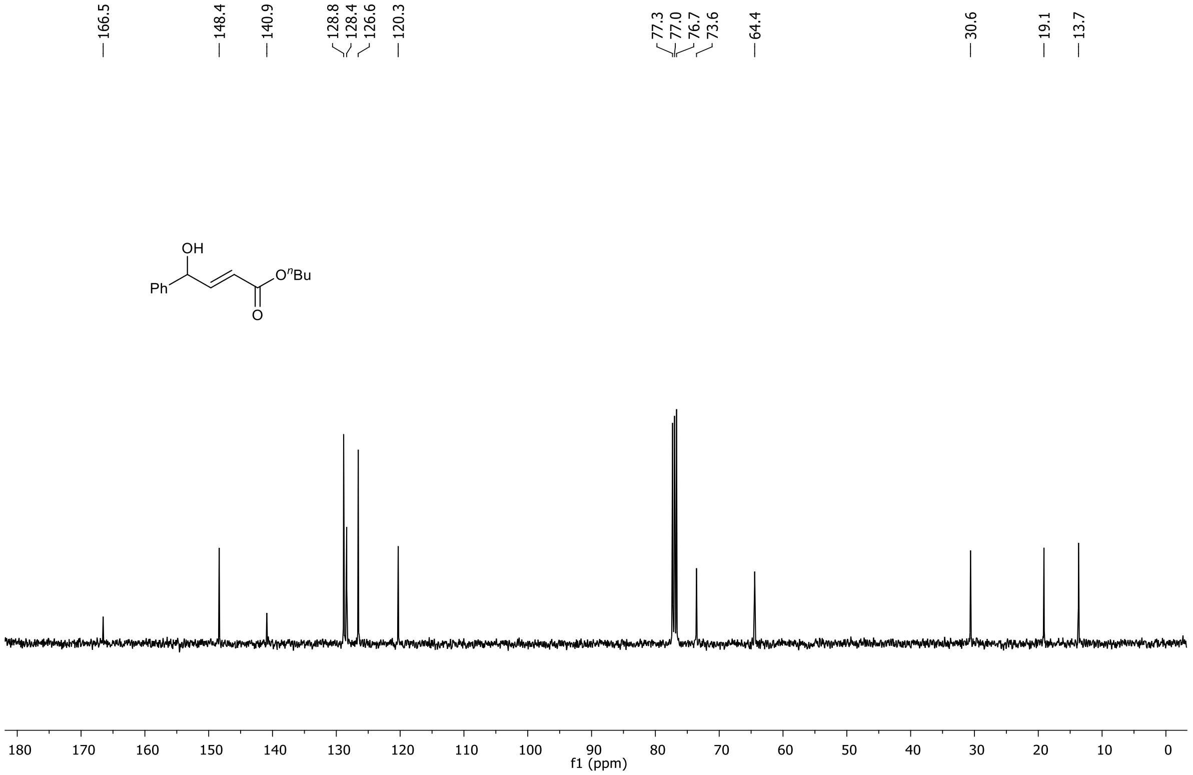

^{1}H NMR (400 MHz, $CDCl_3$) of **3d**

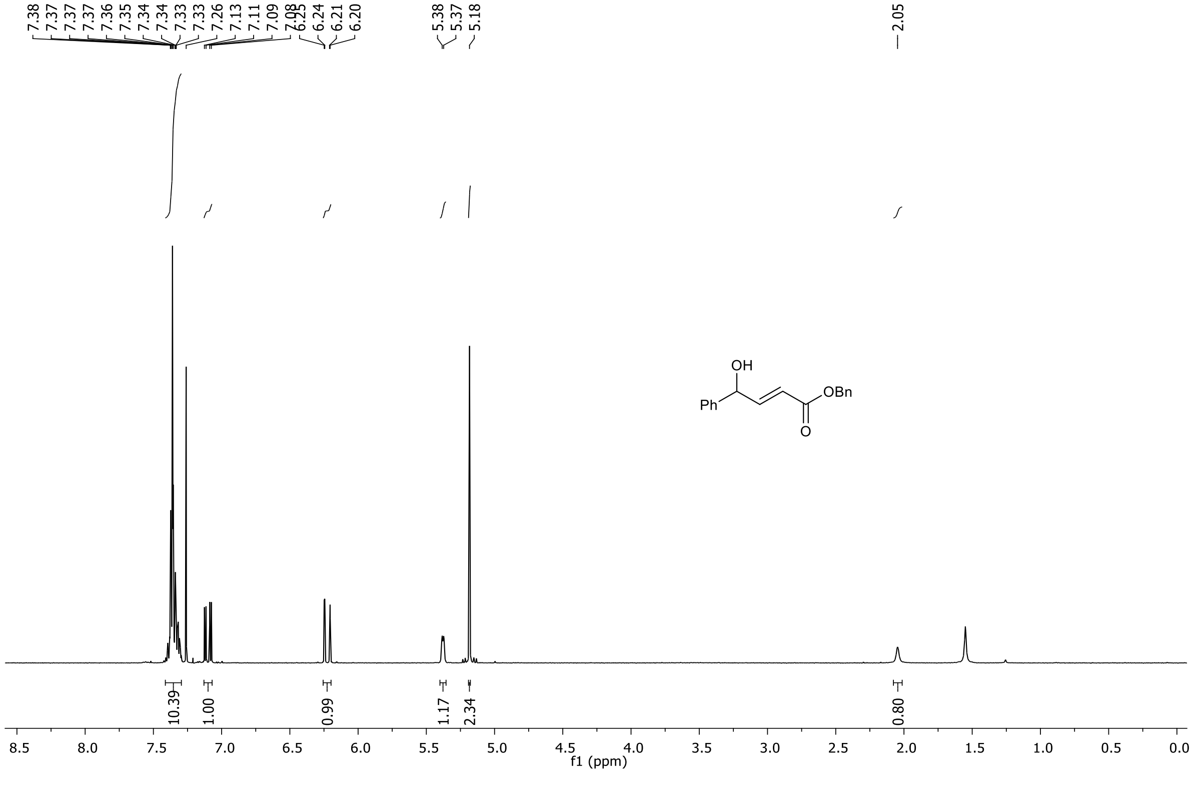

^{13}C NMR (100 MHz, $CDCl_3$) of **3d**

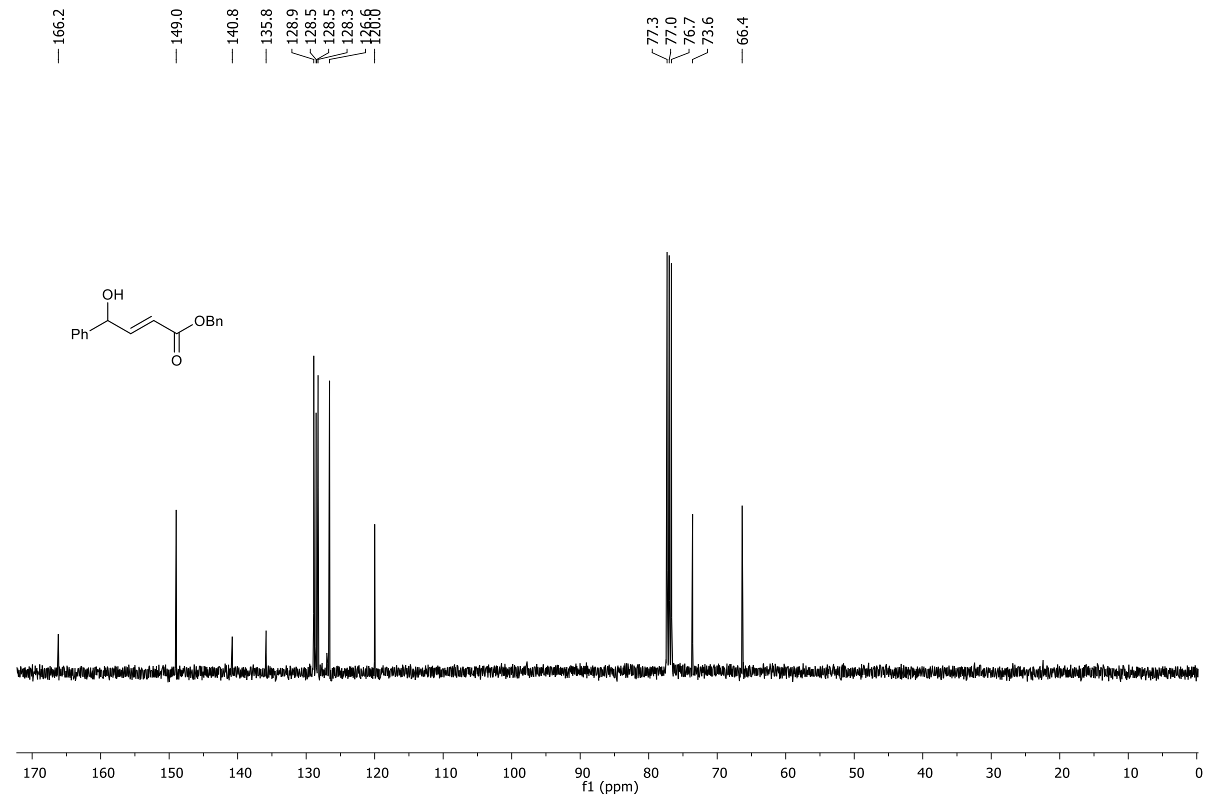

^{1}H NMR (400 MHz, $CDCl_3$) of **3e**

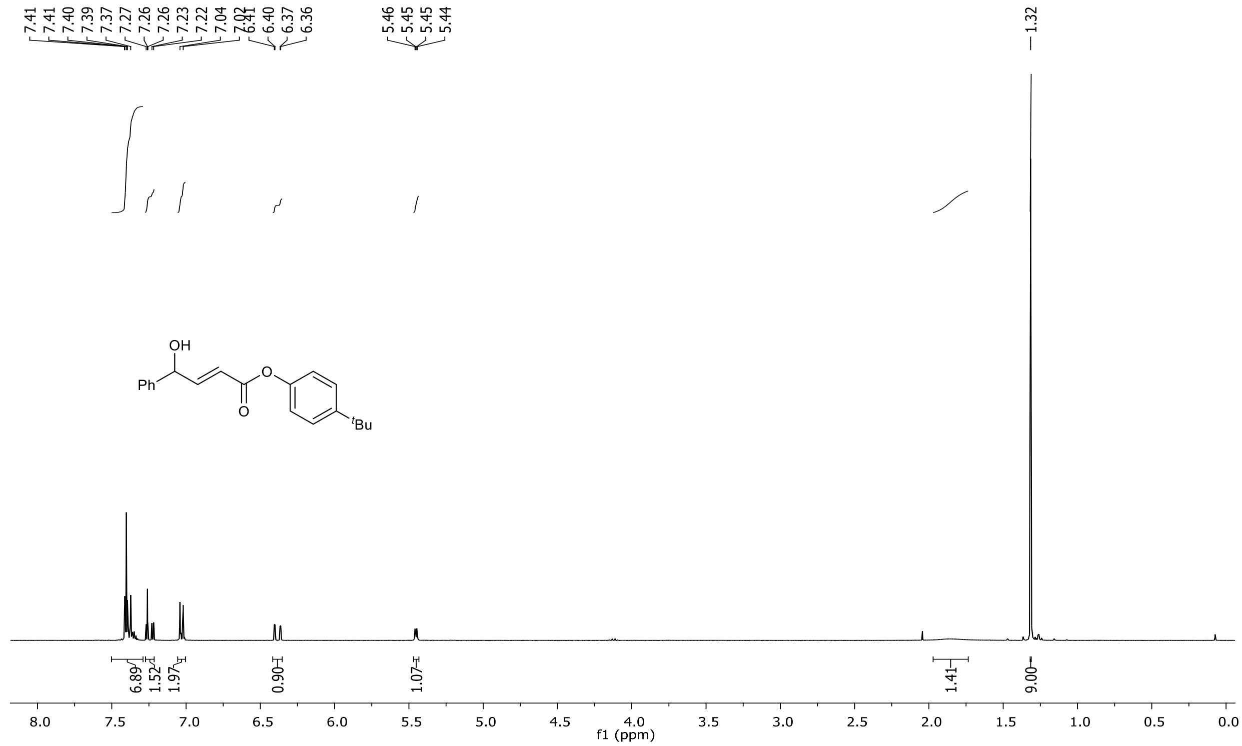

^{13}C NMR (100 MHz, $CDCl_3$) of **3e**

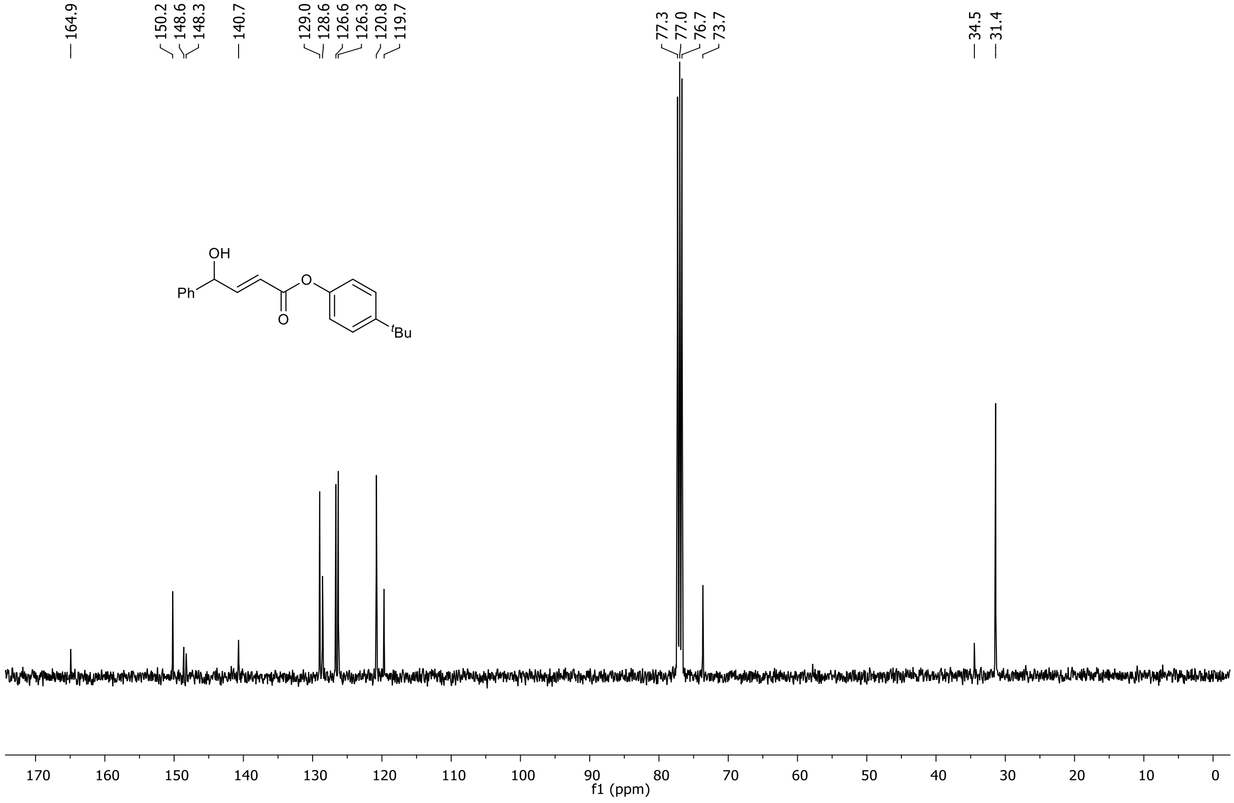

^{1}H NMR (400 MHz, $CDCl_3$) of **3f**

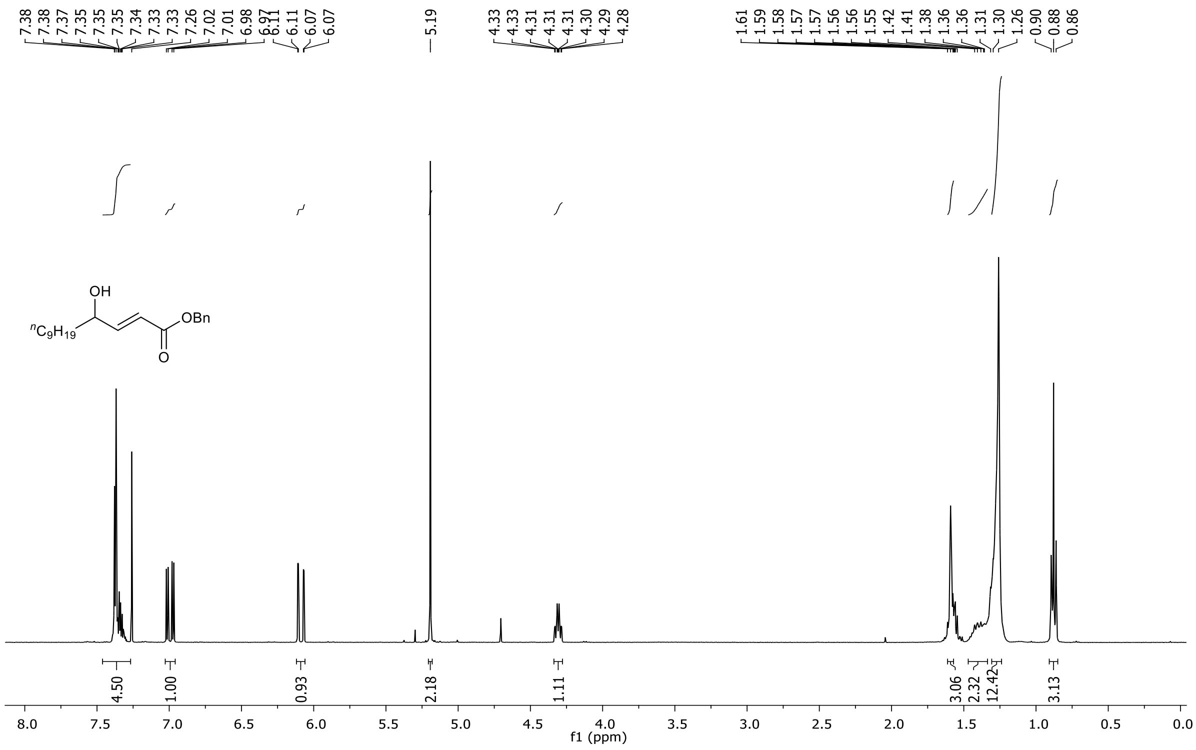

^{13}C NMR (100 MHz, $CDCl_3$) of **3f**

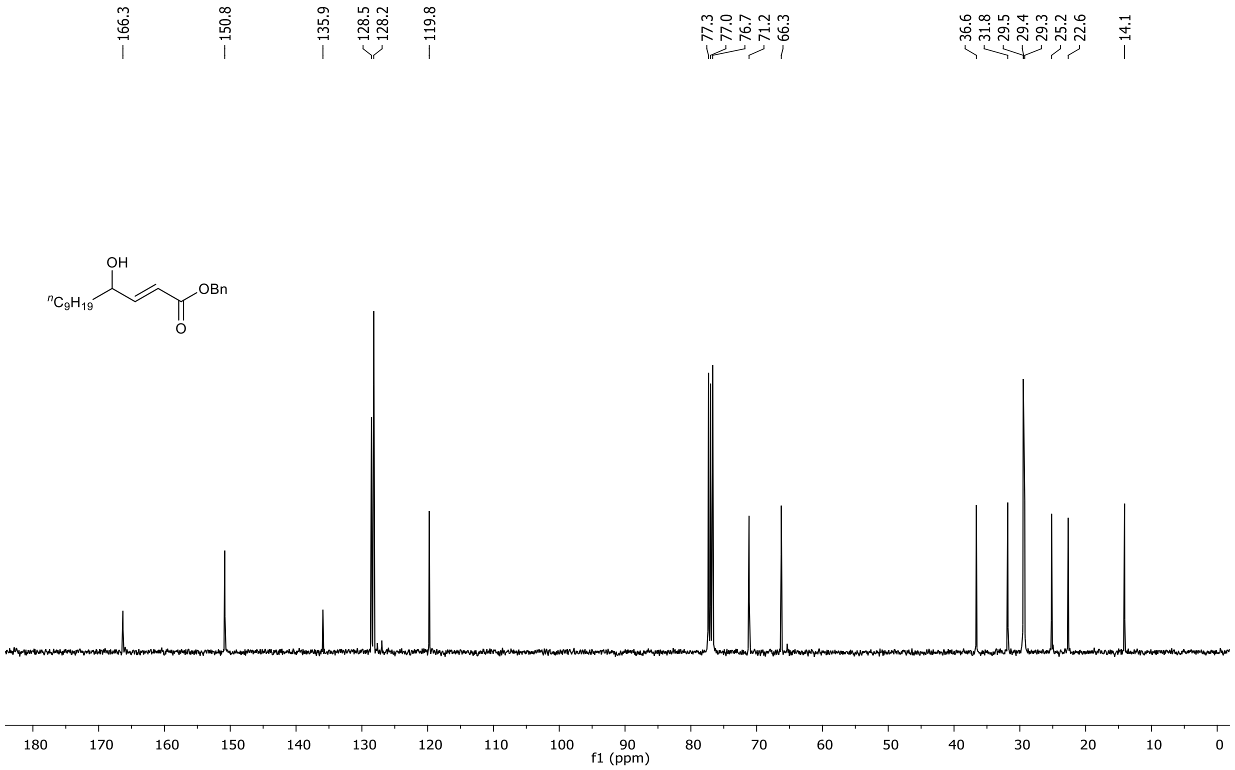

^{1}H NMR (400 MHz, $CDCl_3$) of **3g**

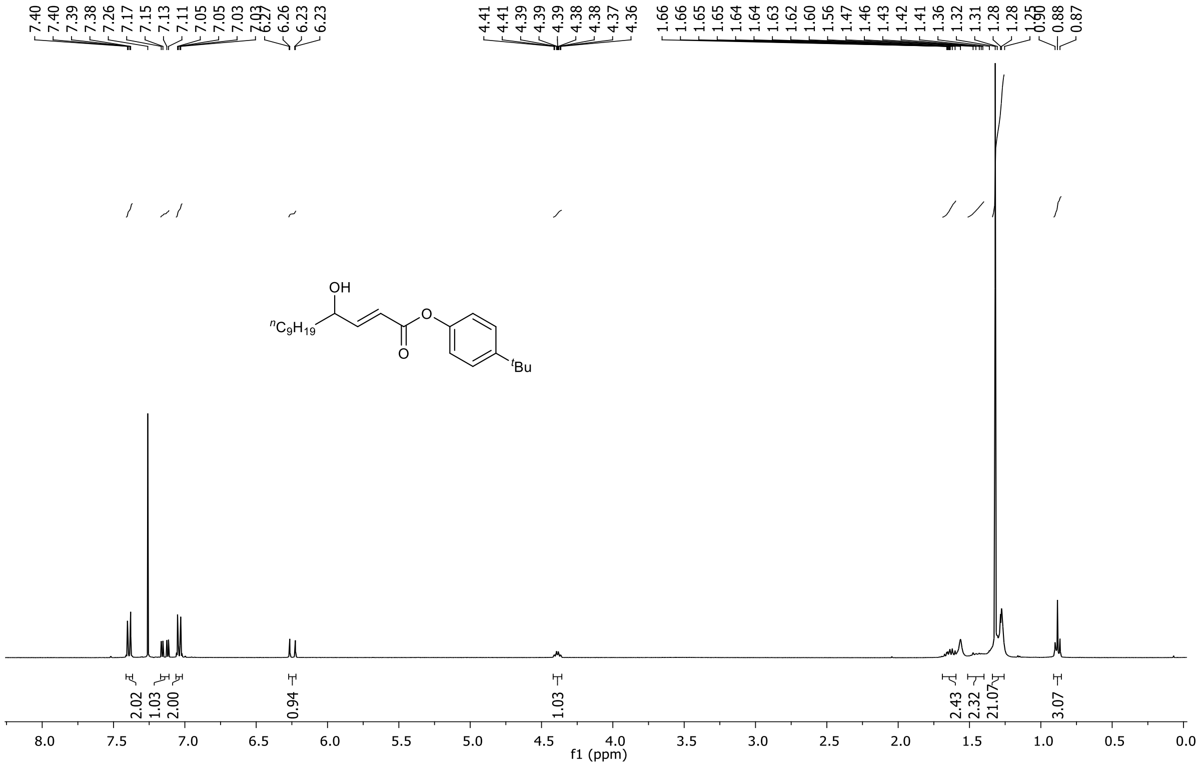

^{13}C NMR (100 MHz, $CDCl_3$) of **3g**

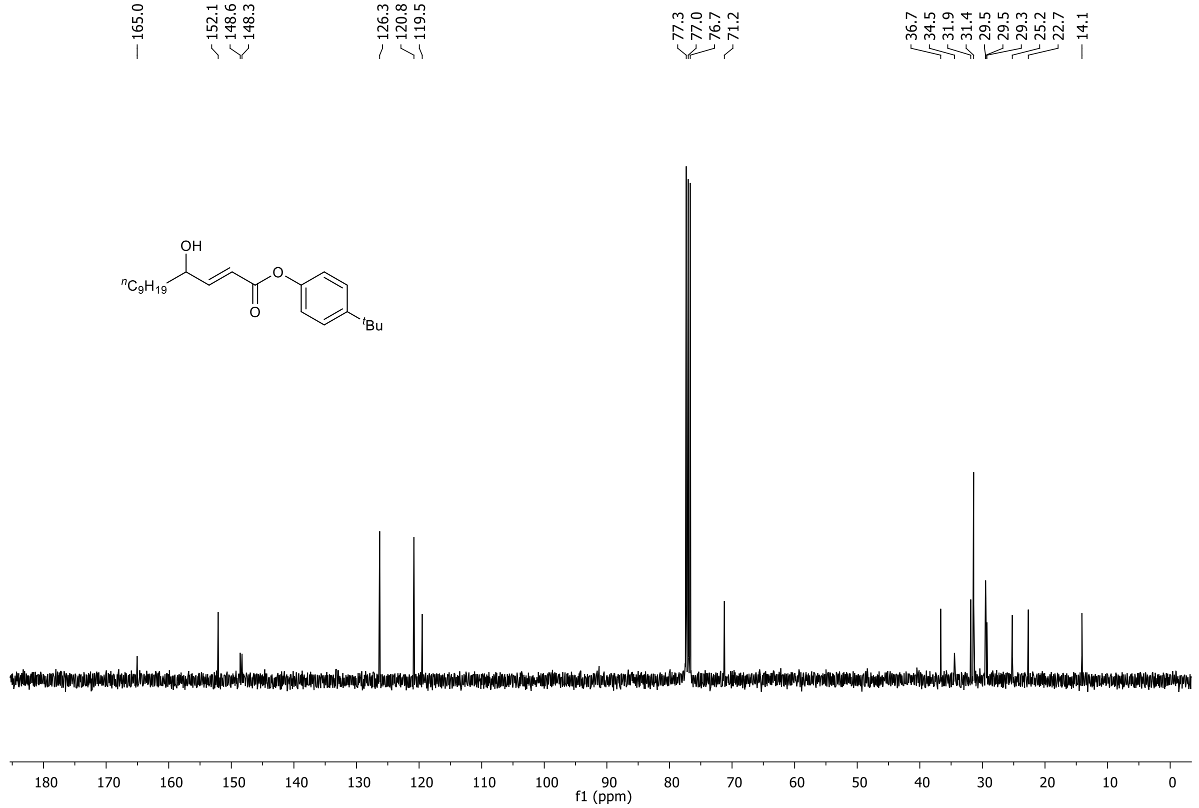

^{1}H NMR (400 MHz, $CDCl_3$) of **3h**

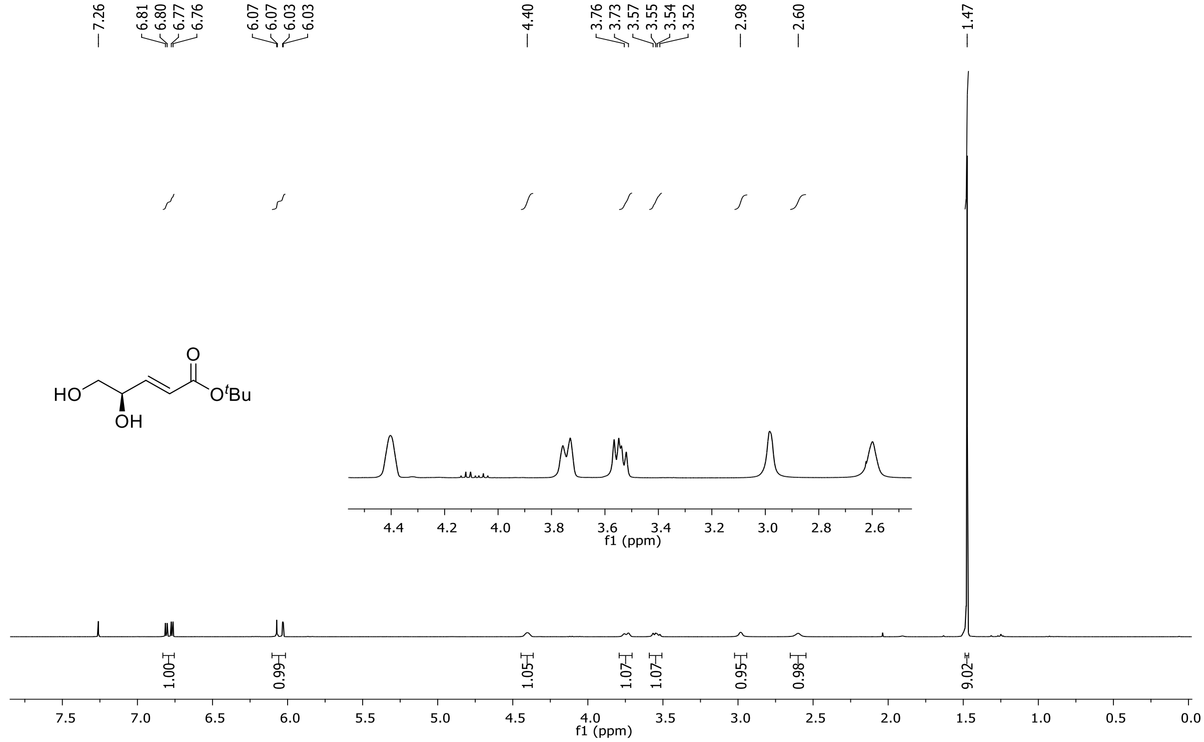

^{13}C NMR (100 MHz, $CDCl_3$) of **3h**

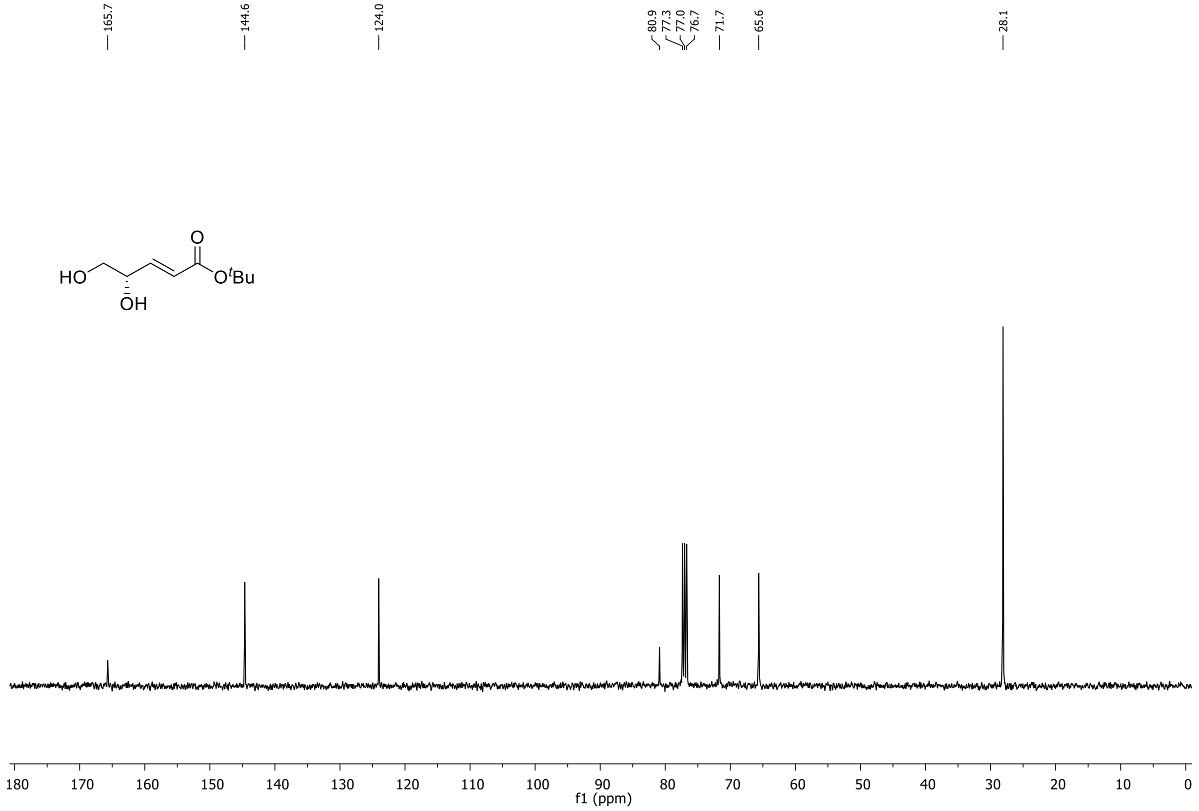

^{1}H NMR (400 MHz, $CDCl_3$) of **3i**

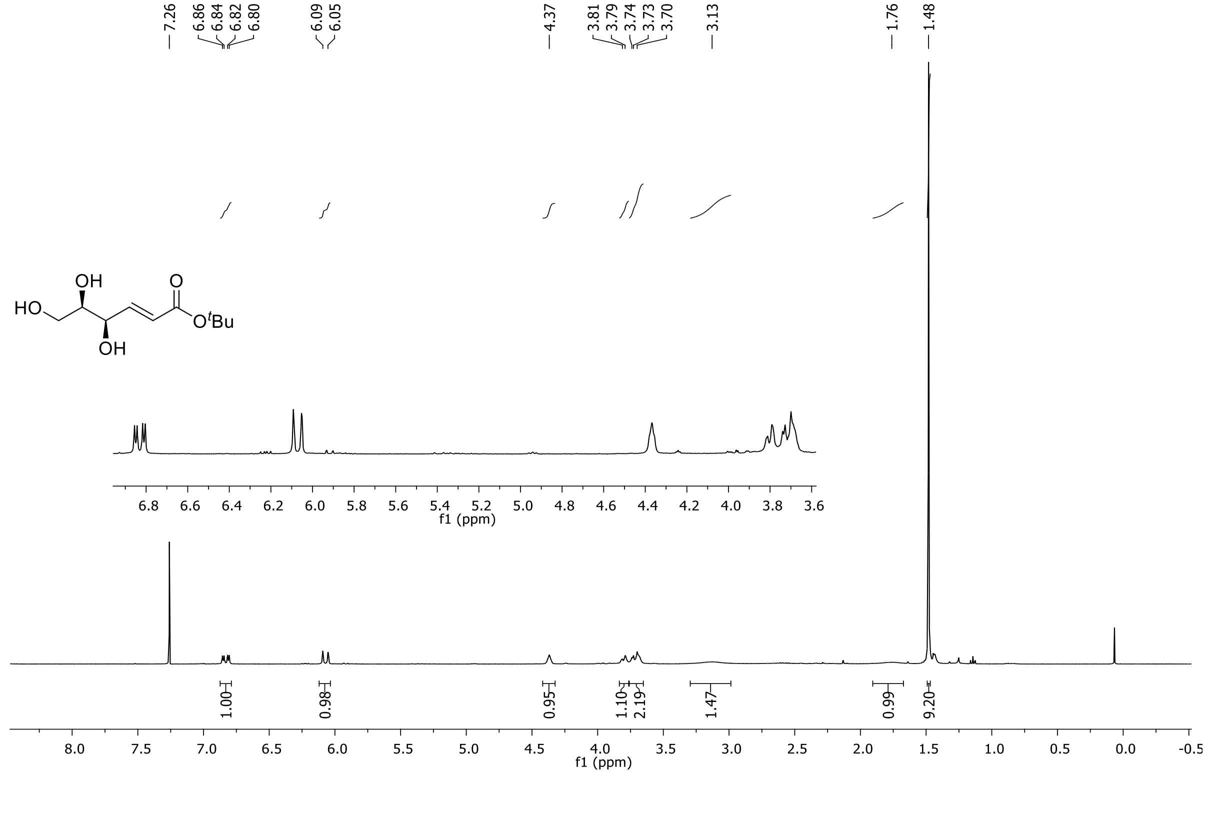

^{13}C NMR (100 MHz, $CDCl_3$) of **3i**

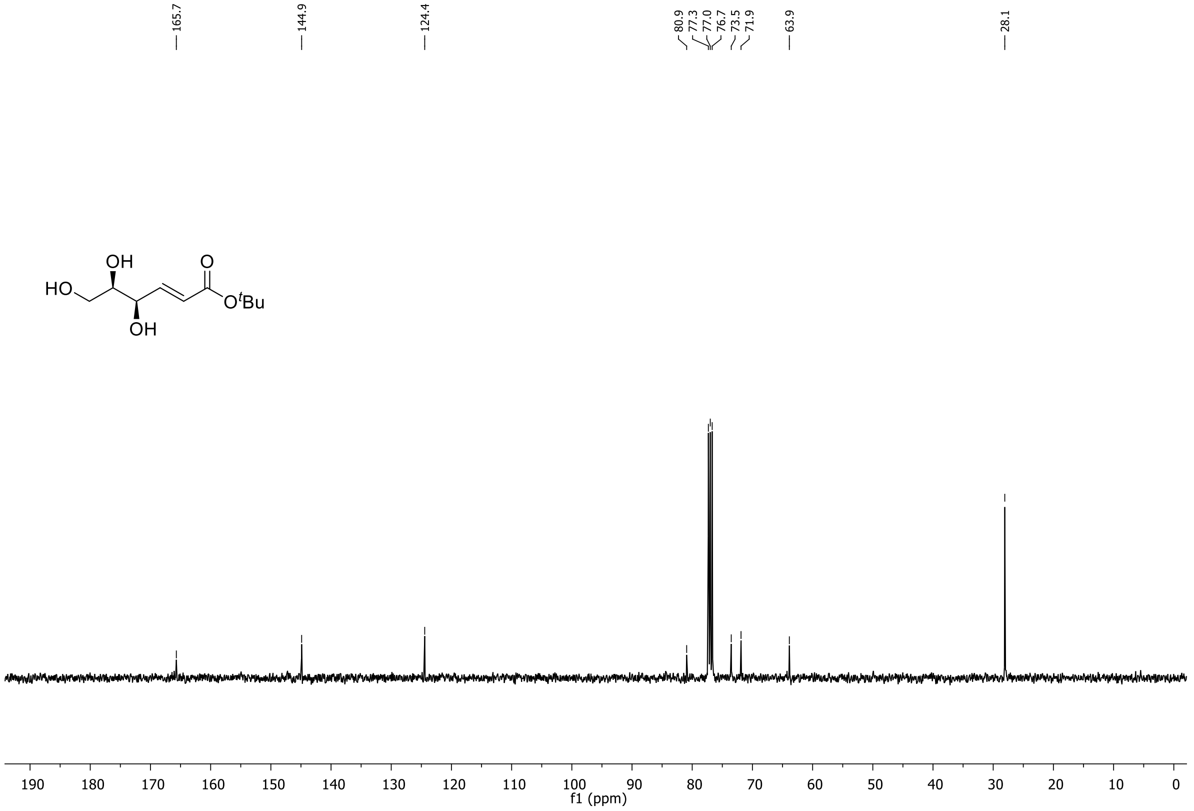

^{1}H NMR (400 MHz, $CDCl_3$) of **3k**

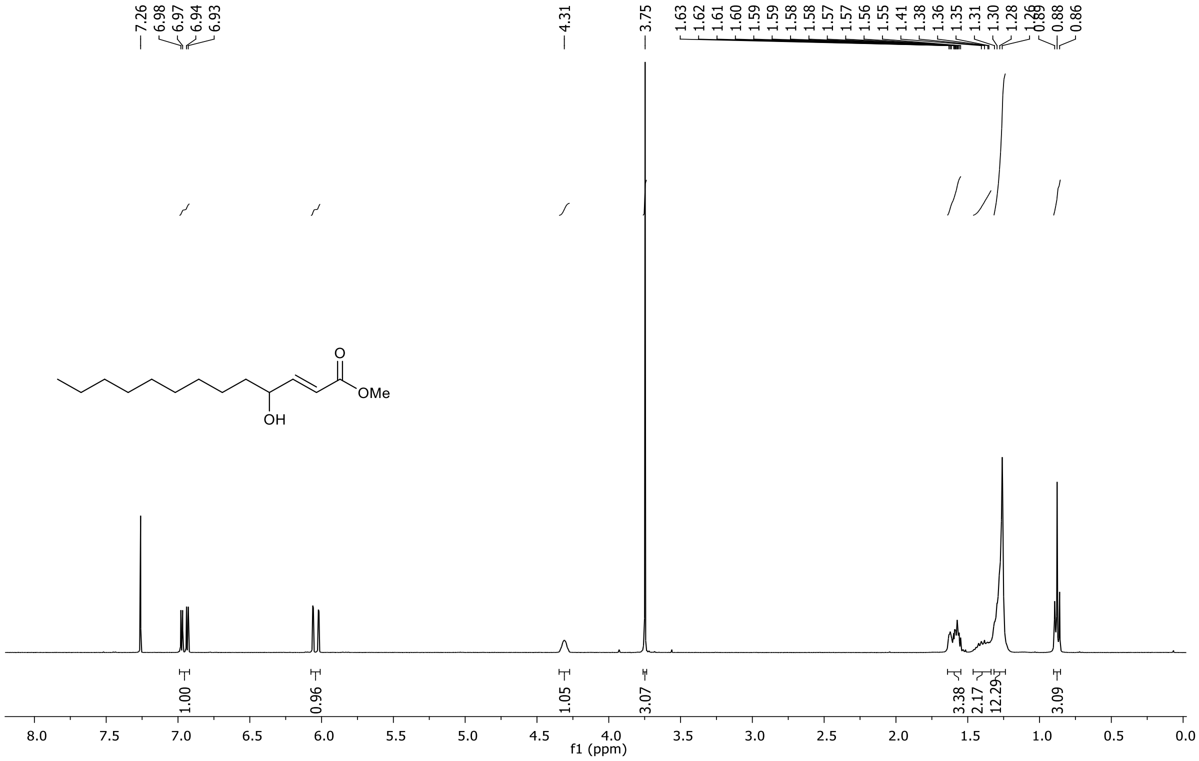

^{13}C NMR (100 MHz, $CDCl_3$) of **3k**

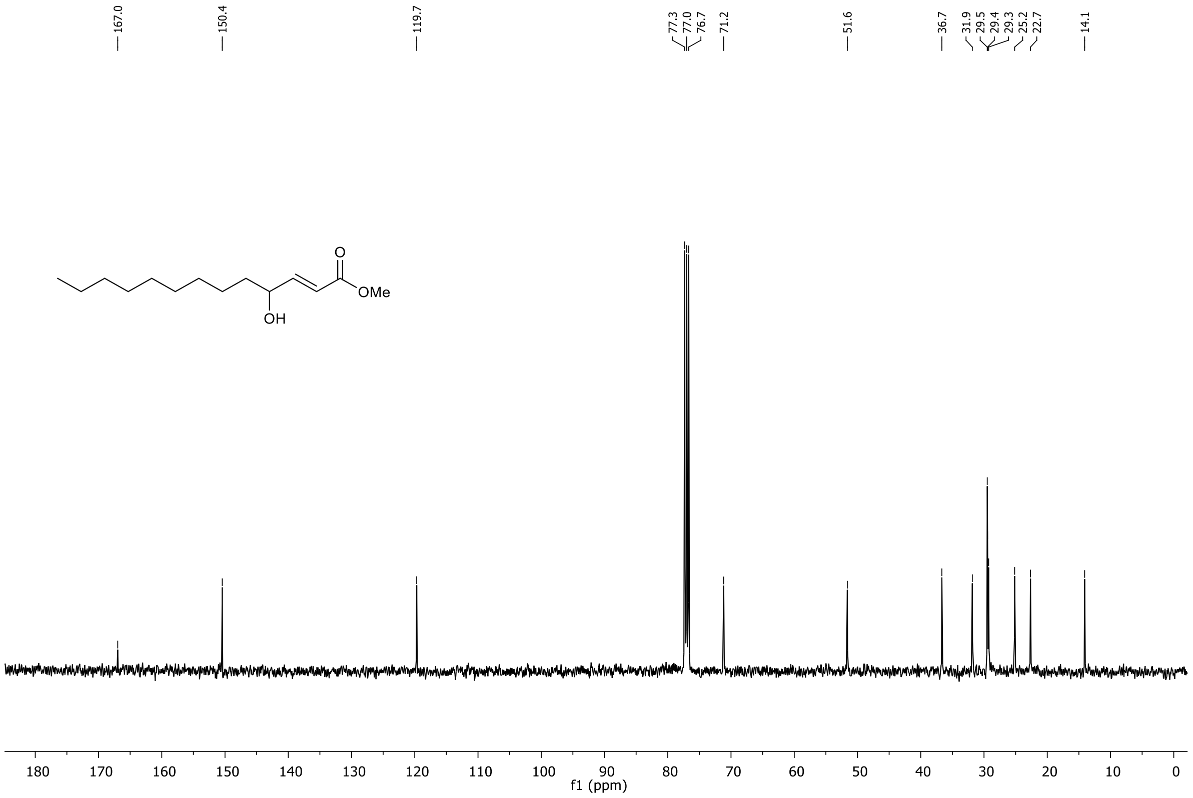

^{1}H NMR (400 MHz, $CDCl_3$) of **3m**

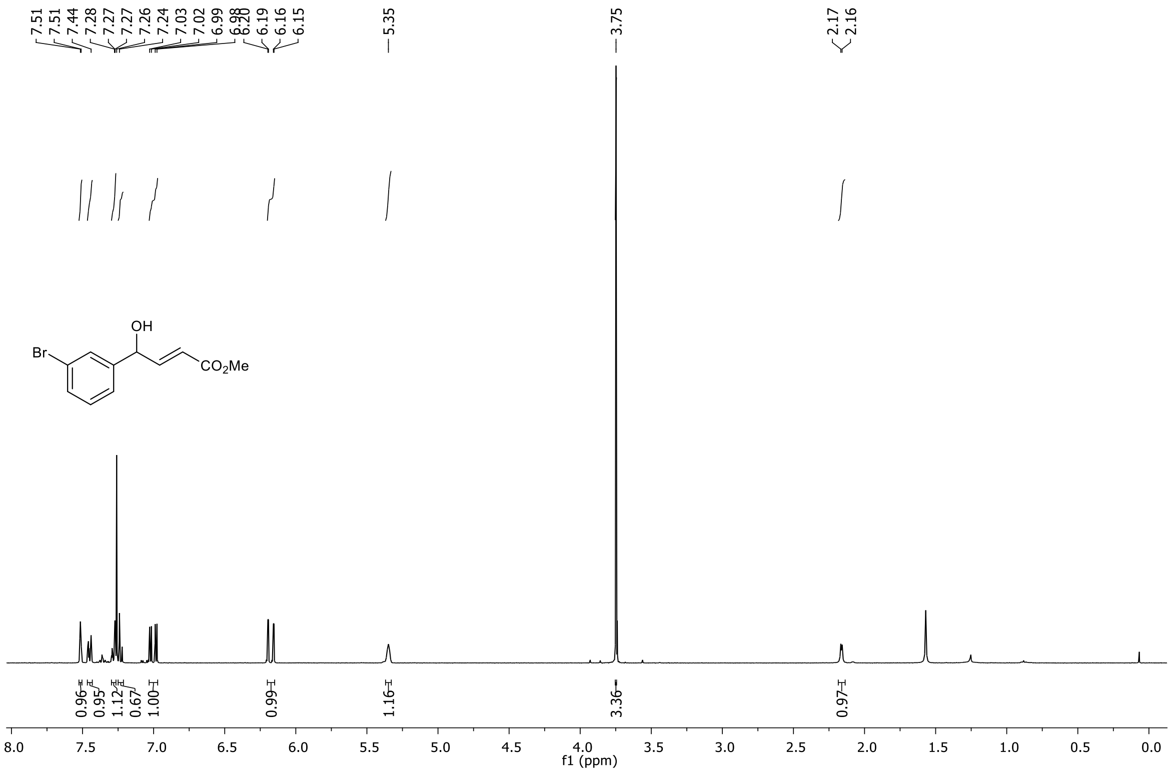

^{13}C NMR (100 MHz, $CDCl_3$) of **3m**

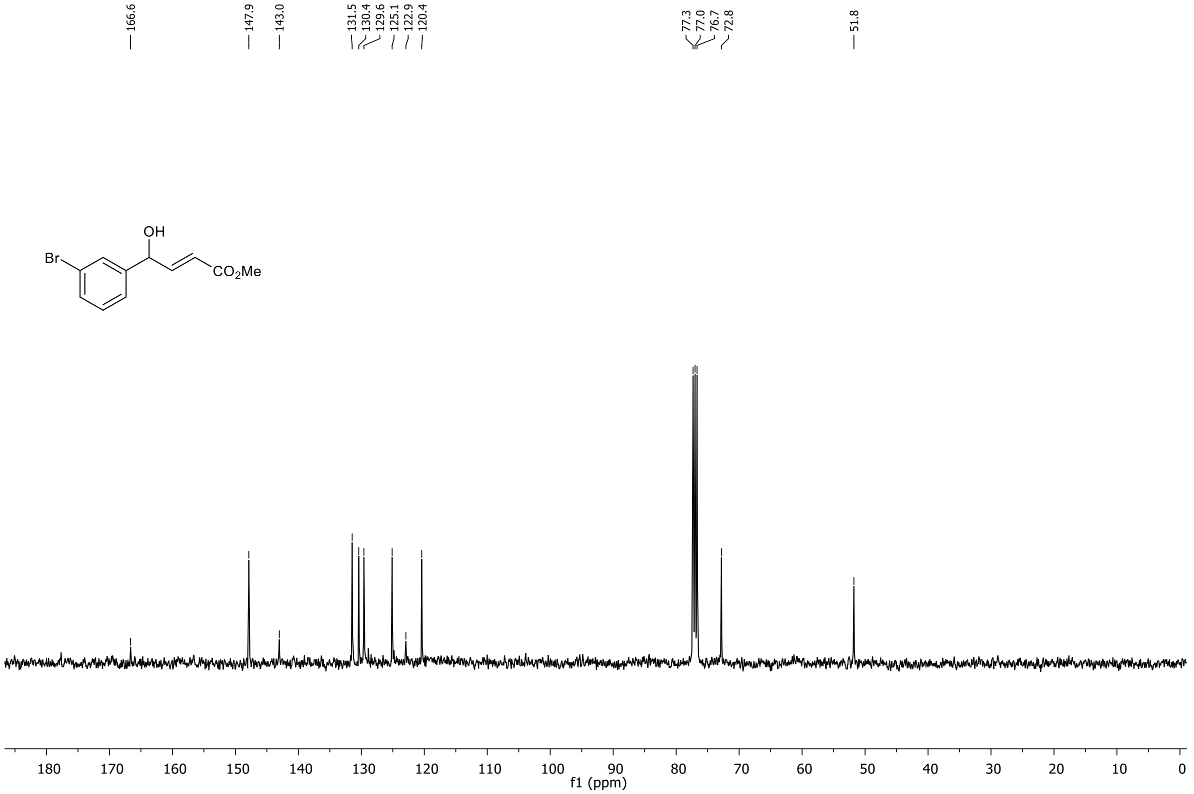

^{1}H NMR (400 MHz, $CDCl_3$) of **3n**

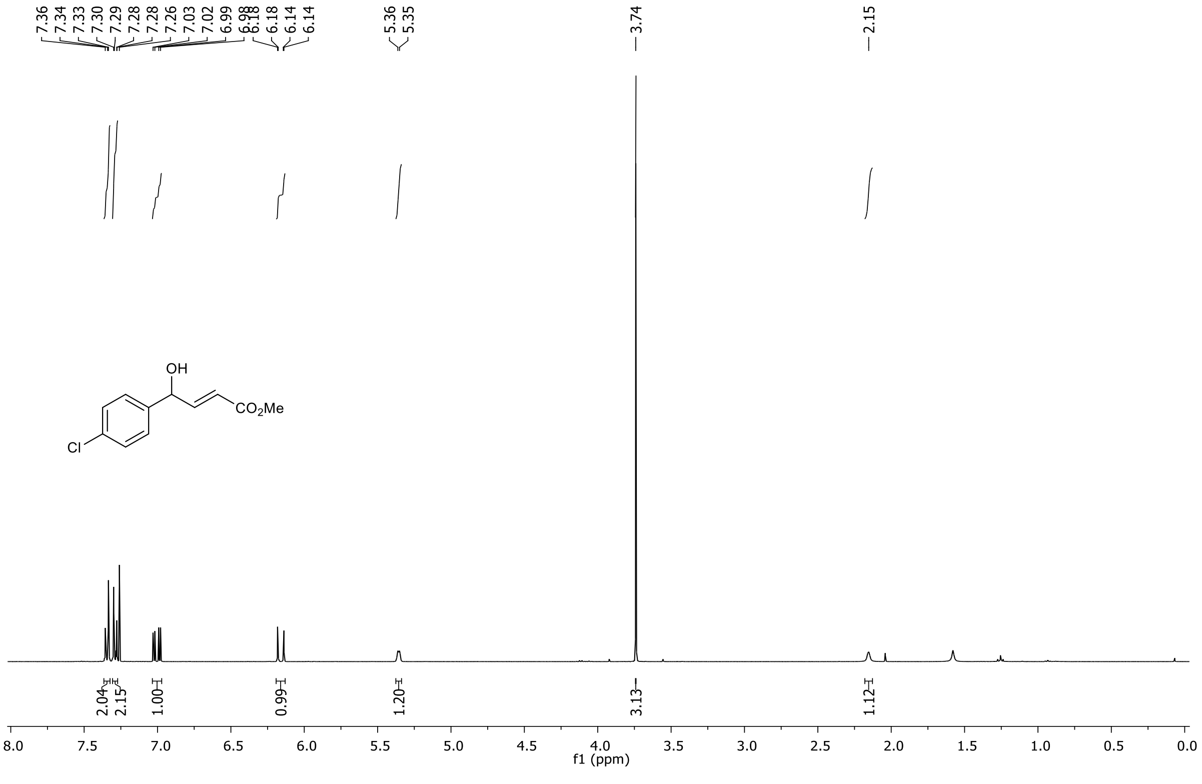

^{13}C NMR (100 MHz, $CDCl_3$) of **3n**

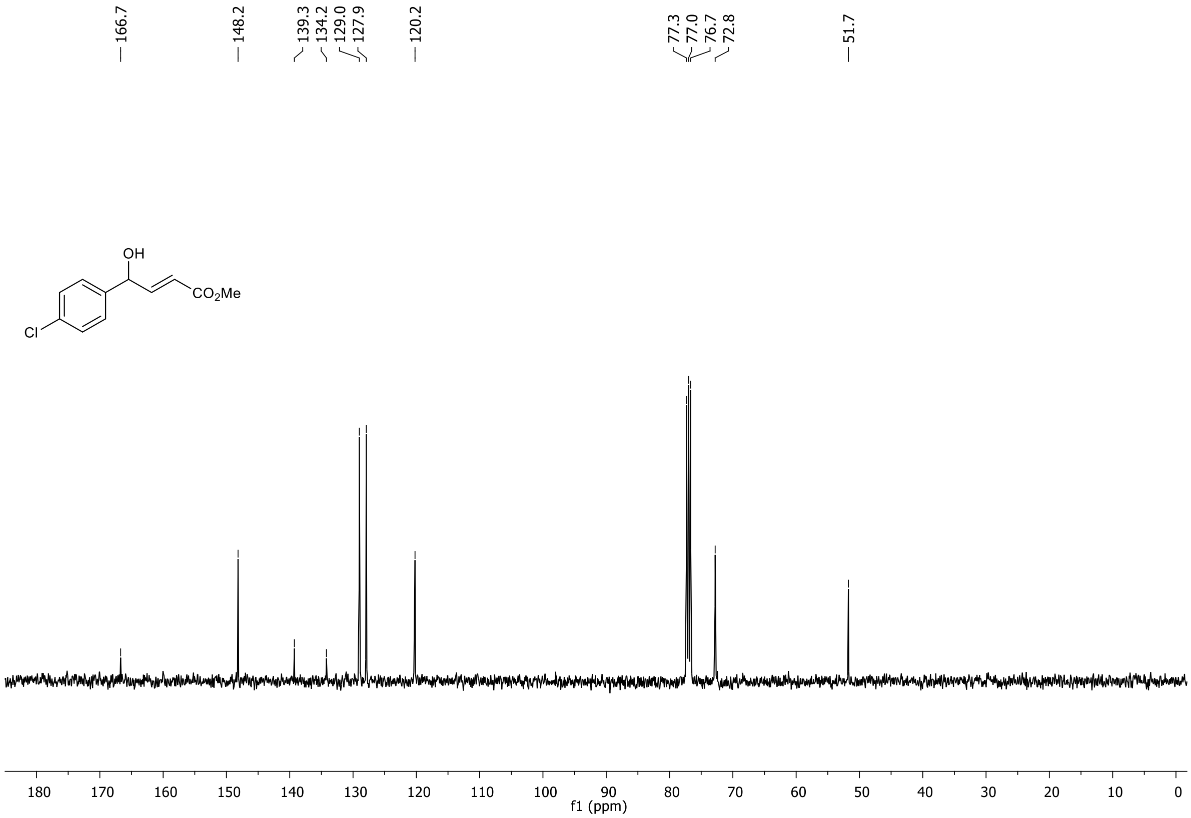

^{1}H NMR (500 MHz, $CDCl_3$) of **3p**

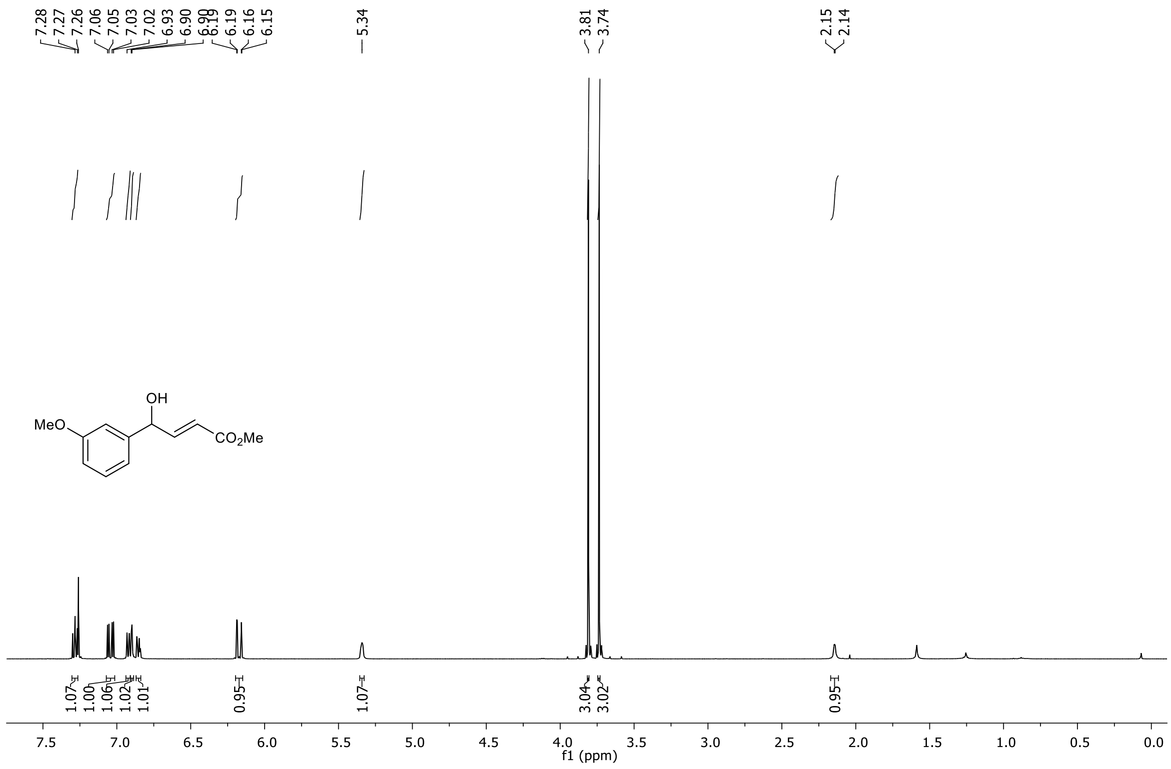

^{13}C NMR (125 MHz, $CDCl_3$) of **3p**

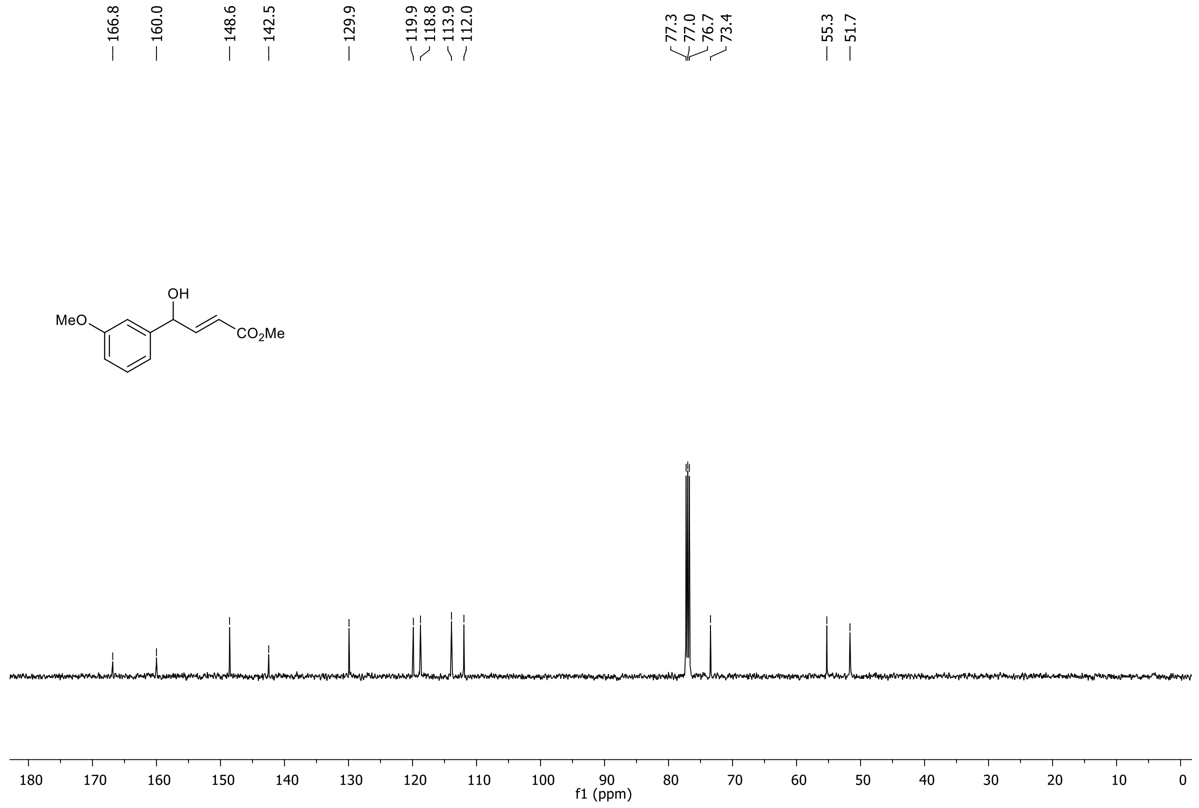

^{1}H NMR (400 MHz, CD_2Cl_2) of **3q**

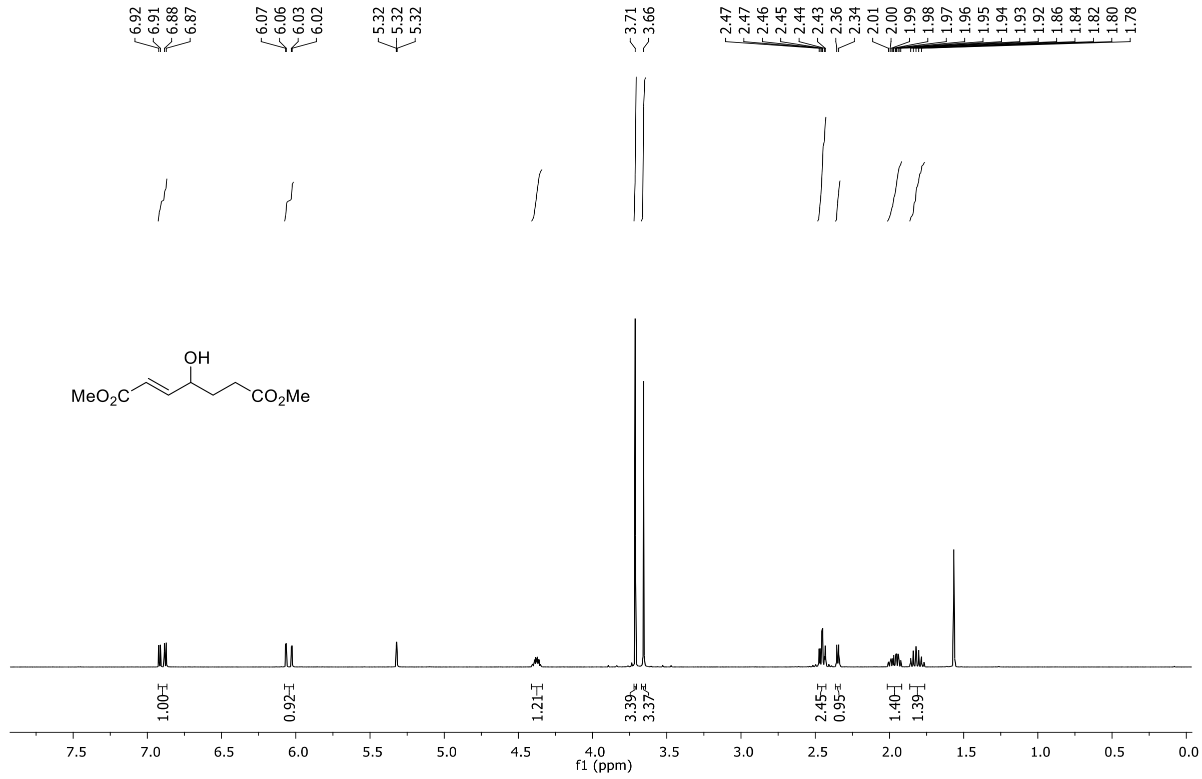

^{13}C NMR (100 MHz, CD_2Cl_2) of **3q**

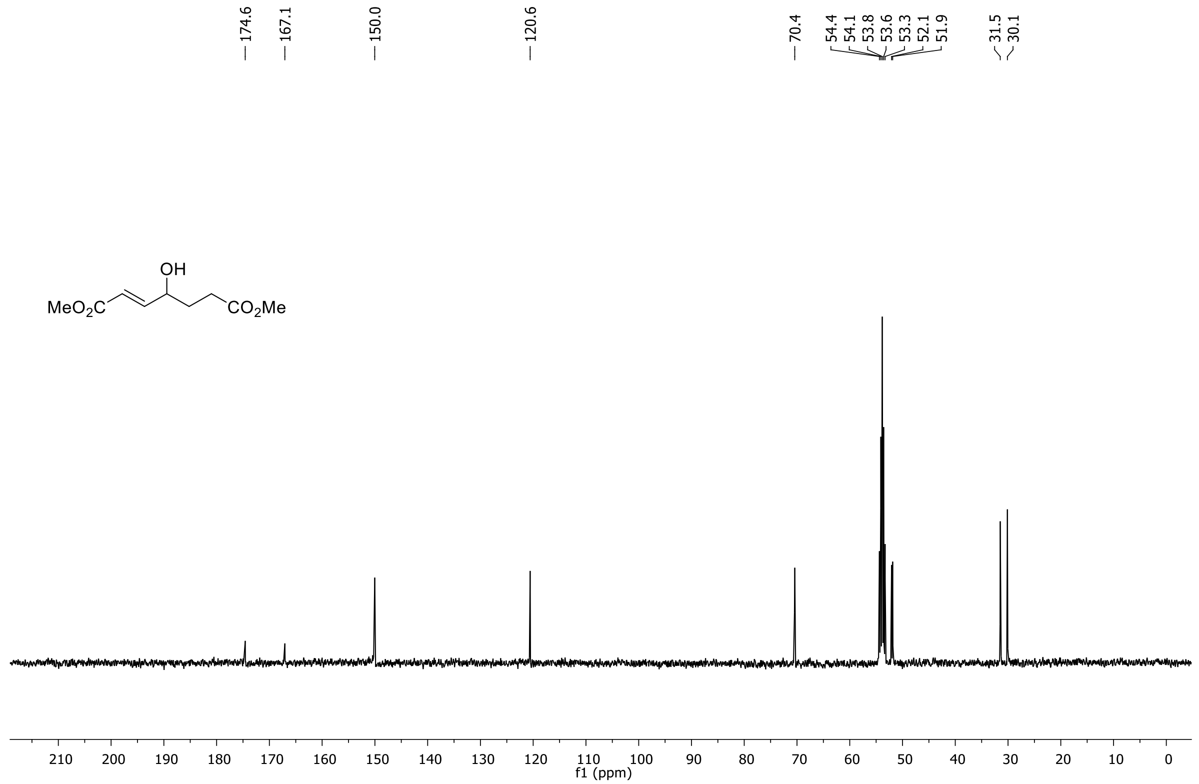

^{1}H NMR (400 MHz, $CDCl_3$) of **3s**

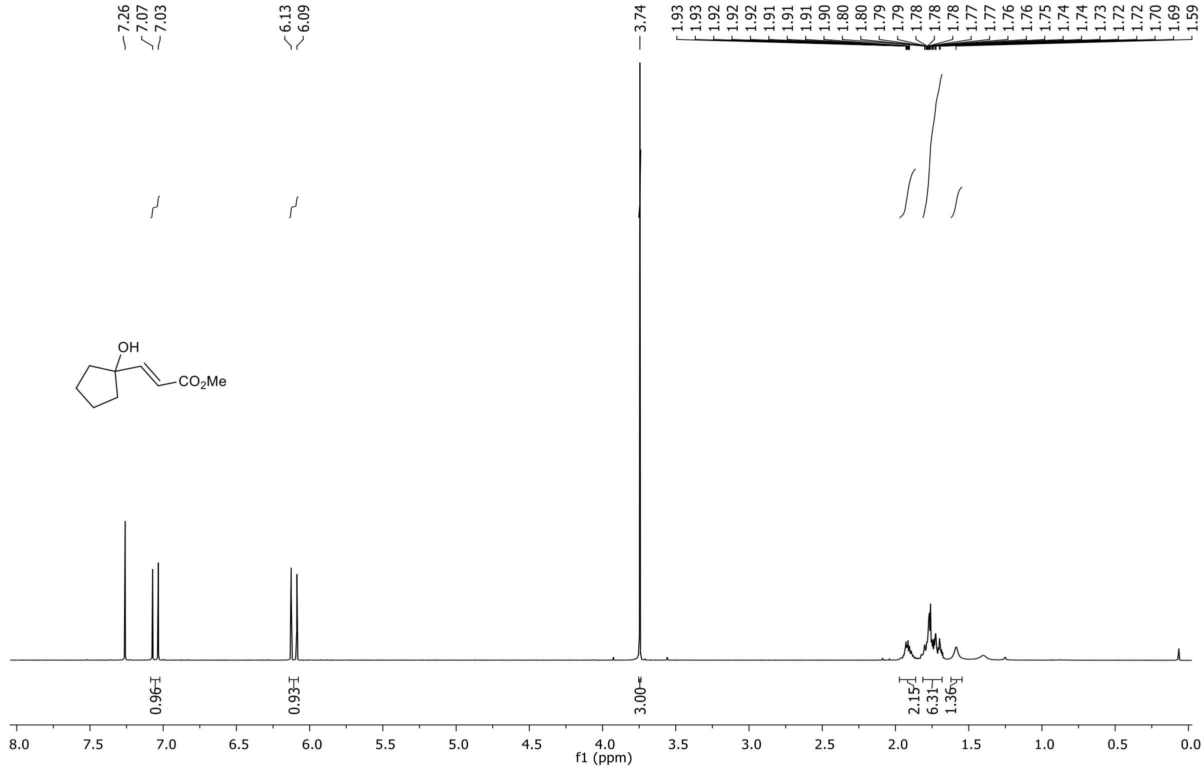

^{13}C NMR (100 MHz, $CDCl_3$) of **3s**

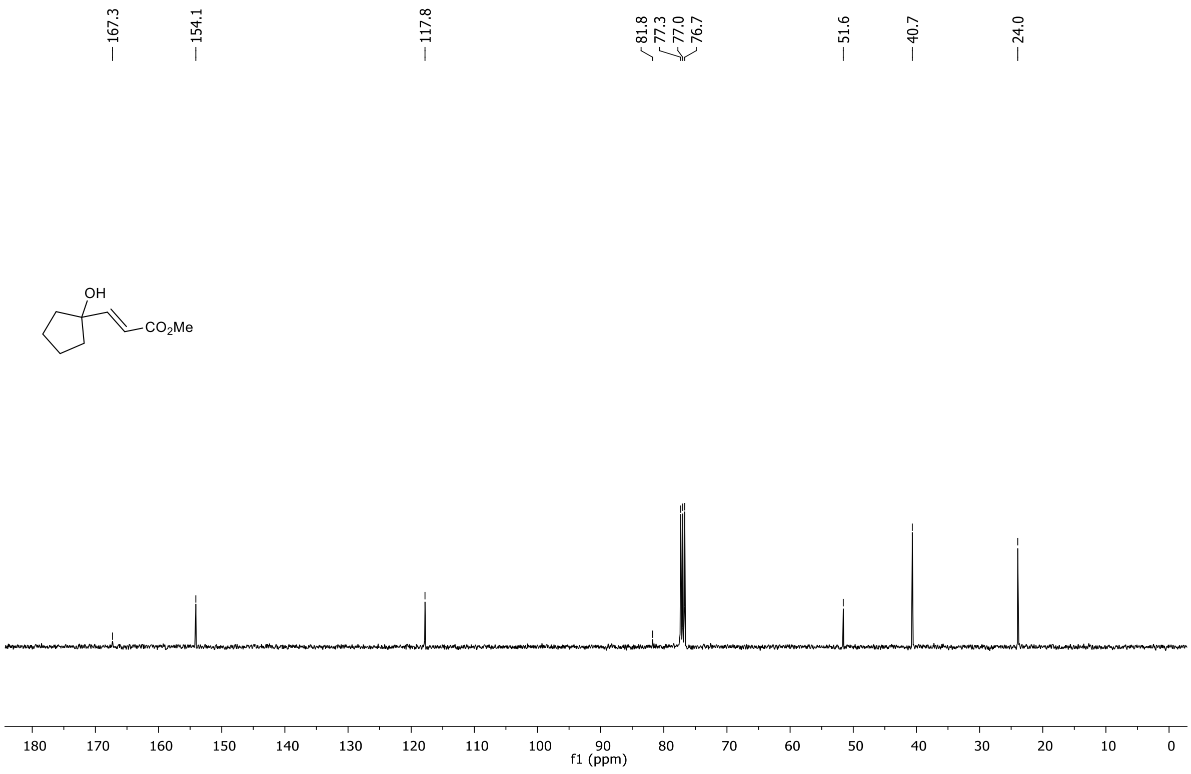

^{1}H NMR (400 MHz, $CDCl_3$) of **S3**

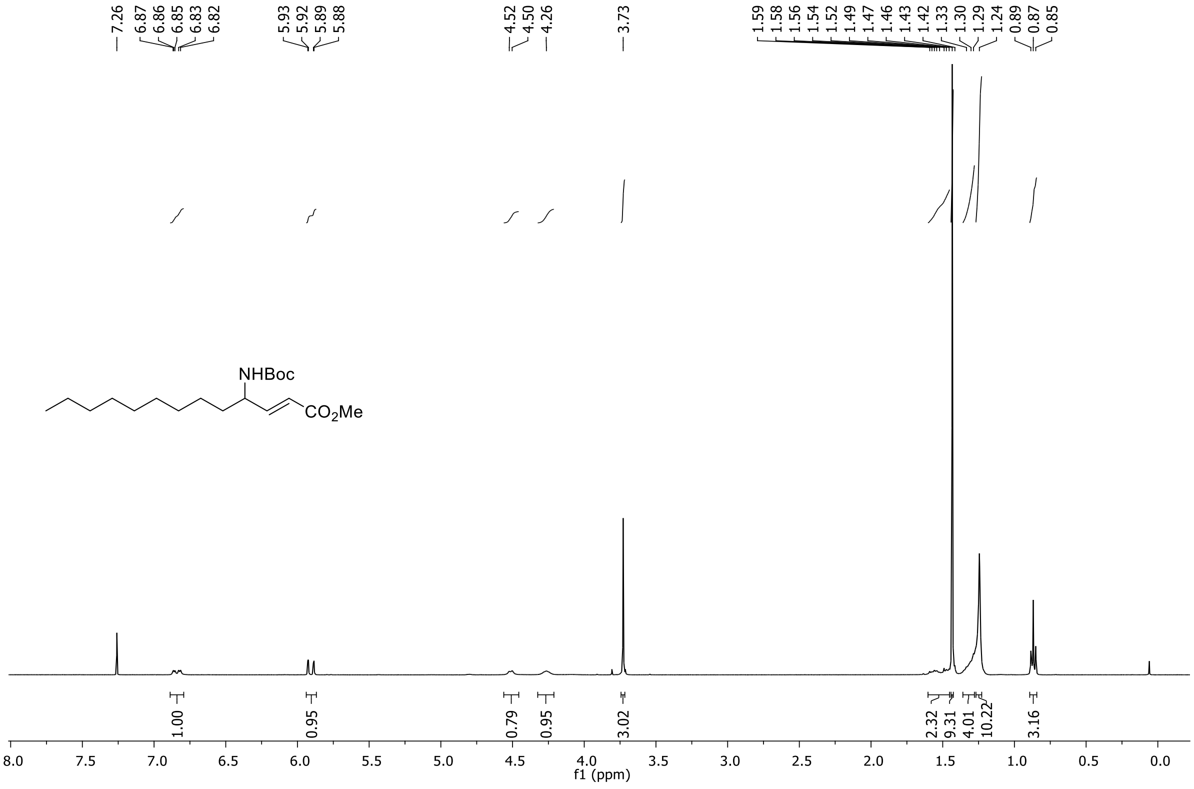

^{13}C NMR (100 MHz, $CDCl_3$) of **S3**

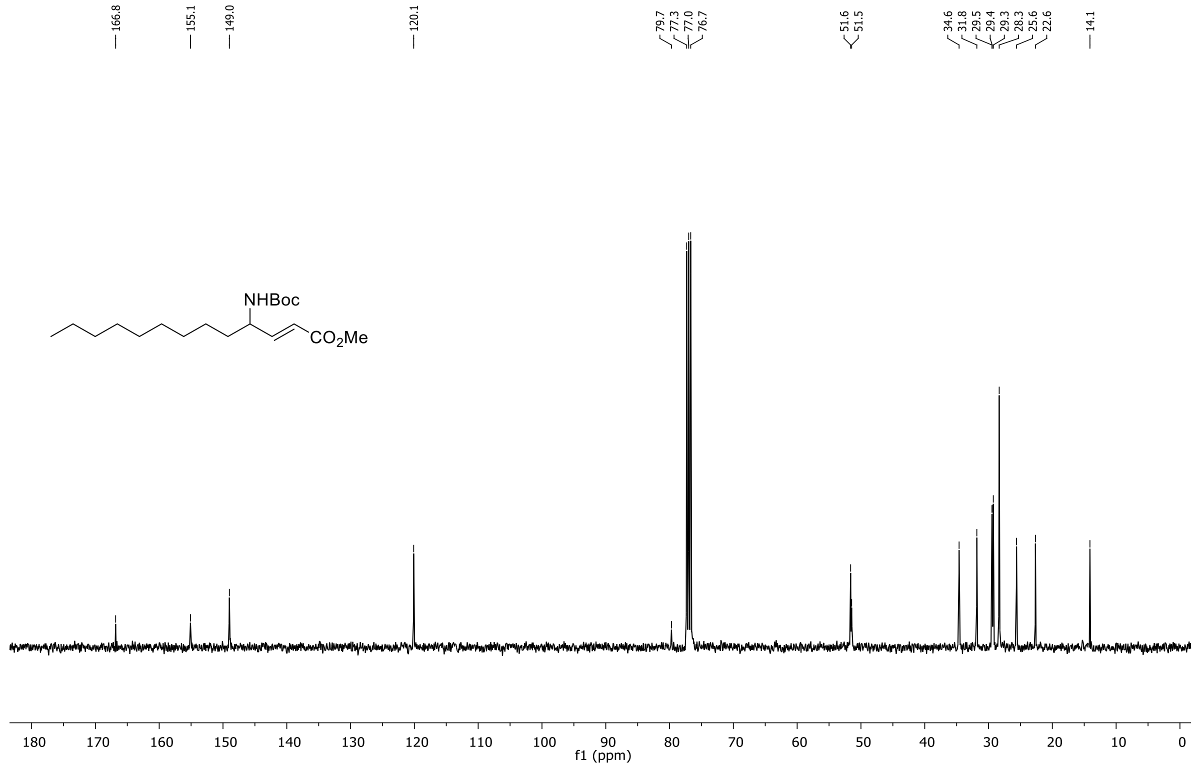

^{1}H NMR (500 MHz, $CDCl_3$) of **3t**

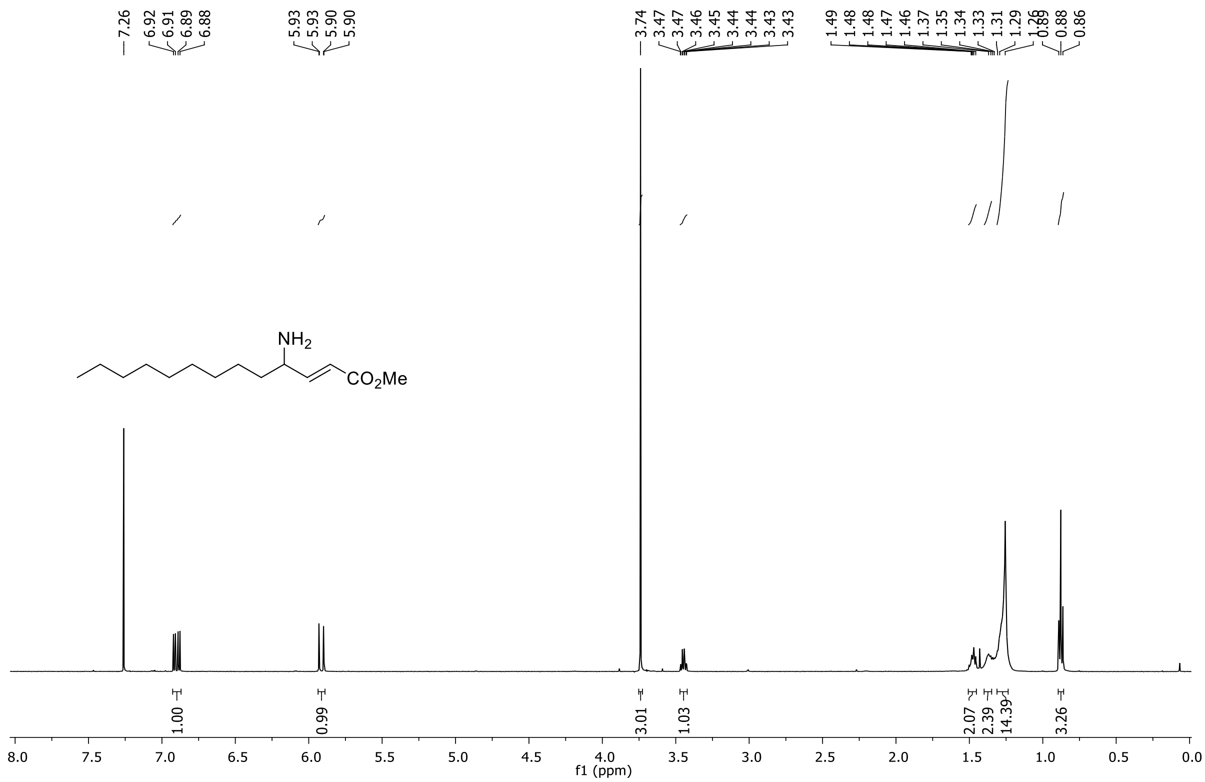

^{13}C NMR (125 MHz, $CDCl_3$) of **3t**

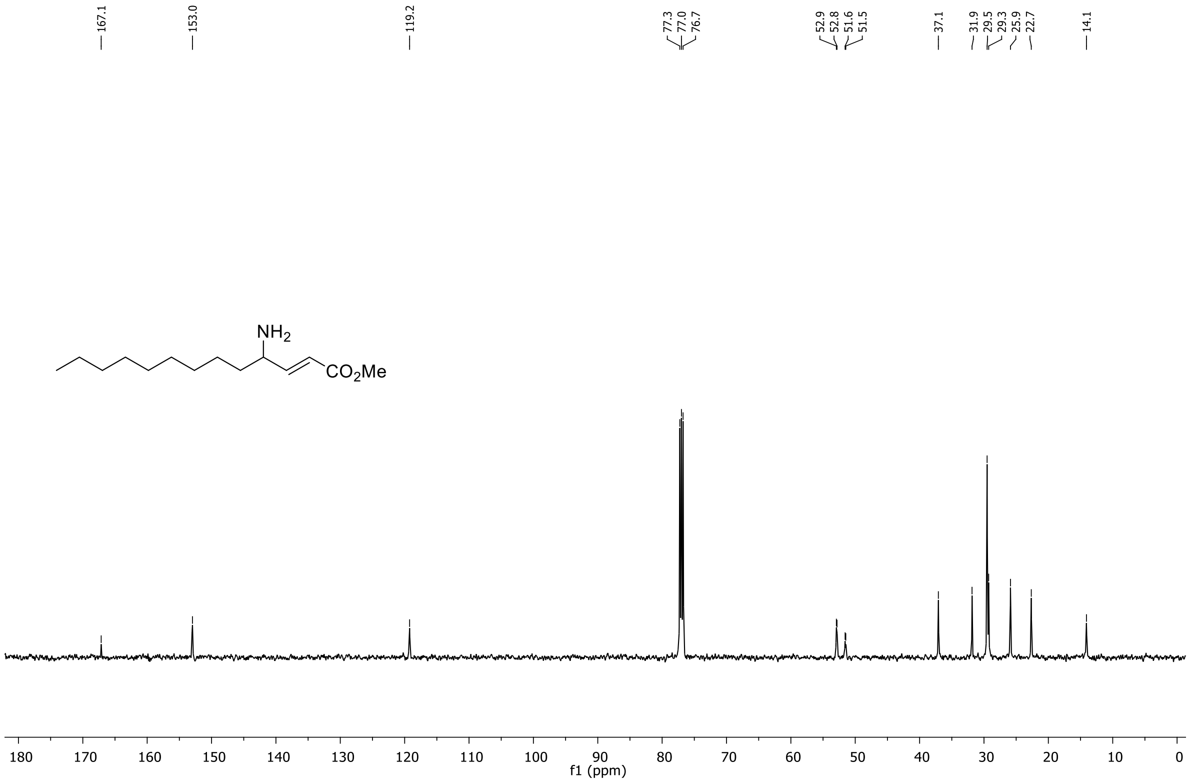

^{1}H NMR (400 MHz, $CDCl_3$) of **3u**

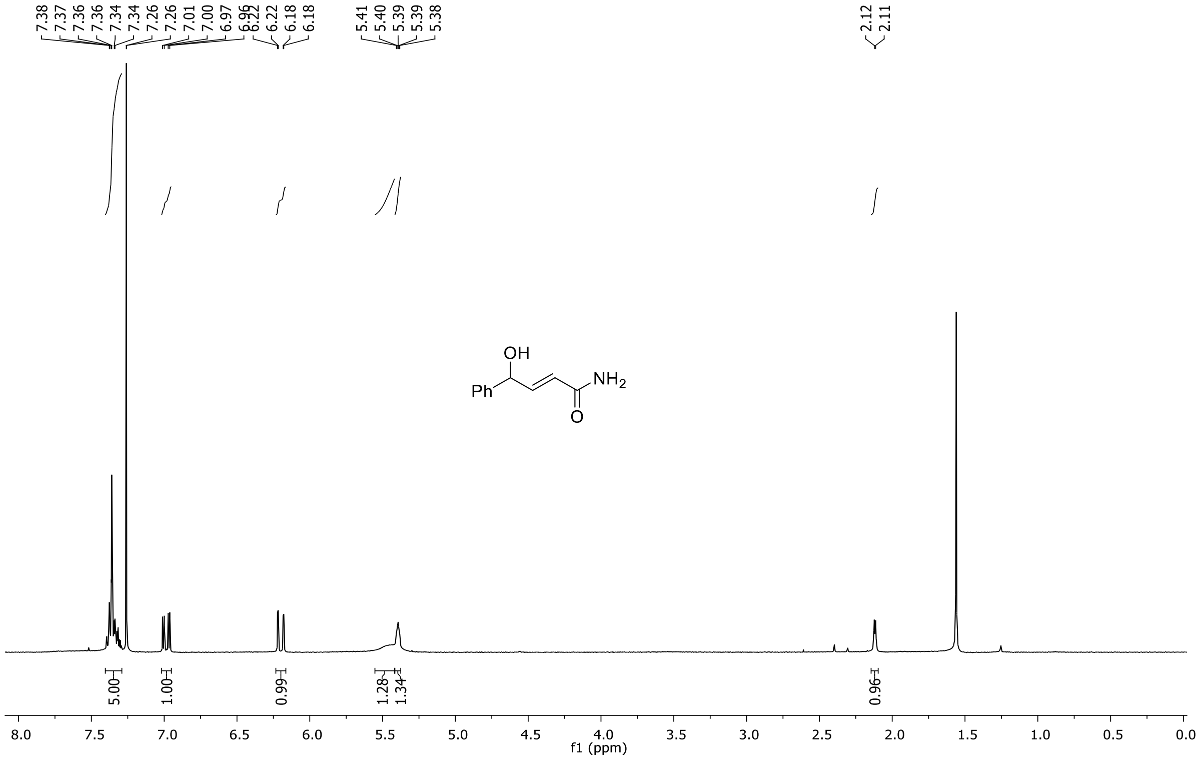

^{13}C NMR (100 MHz, Acetone-d6) of **3u**

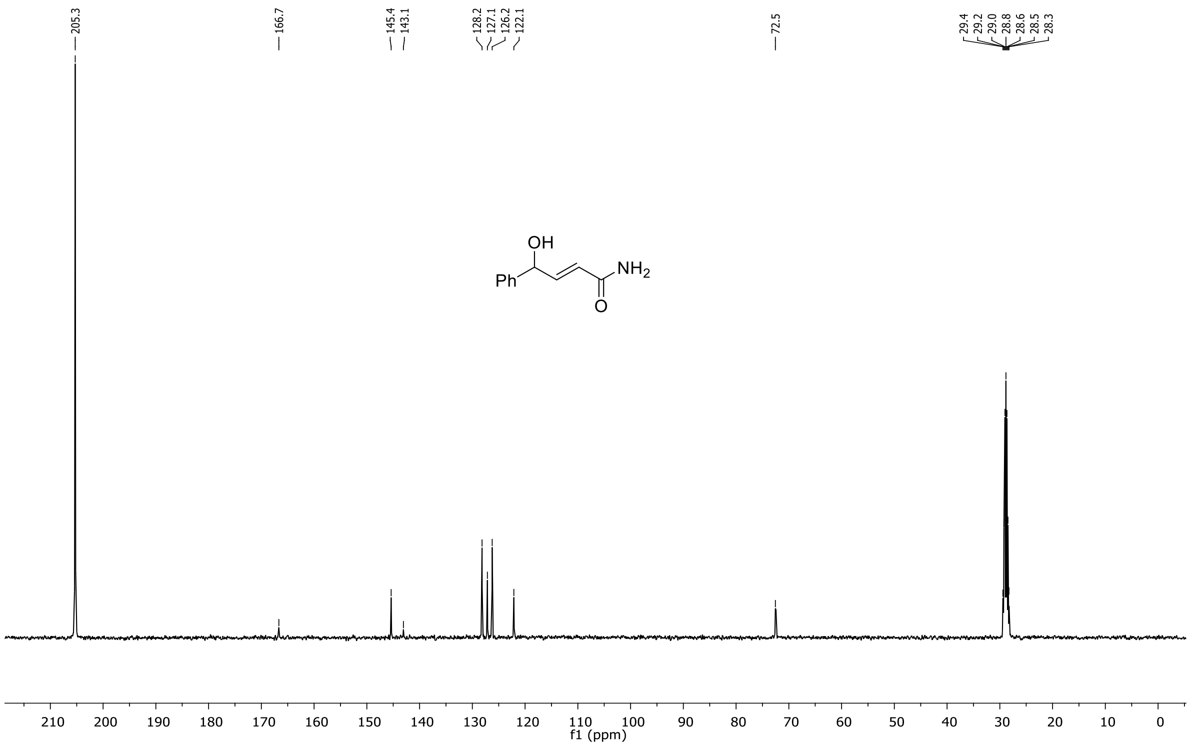

^{1}H NMR (400 MHz, $CDCl_3$) of **3v**

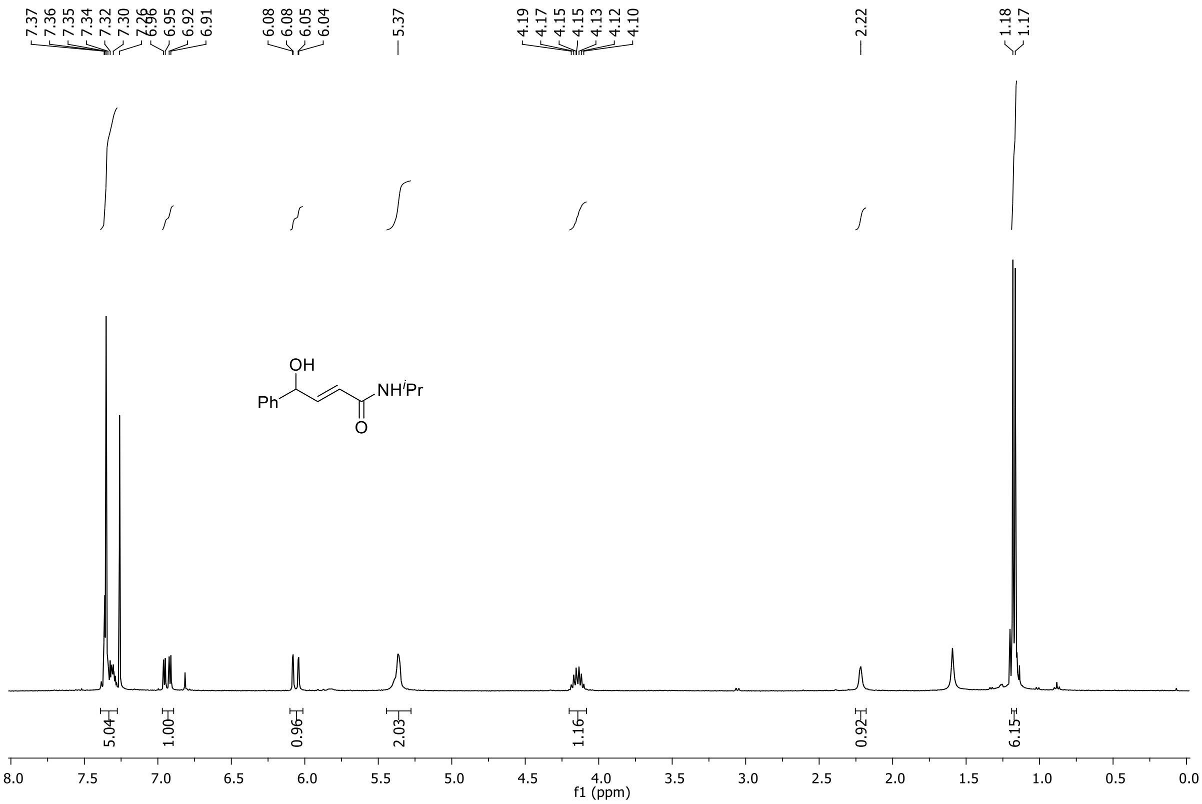

^{13}C NMR (100 MHz, $CDCl_3$) of **3v**

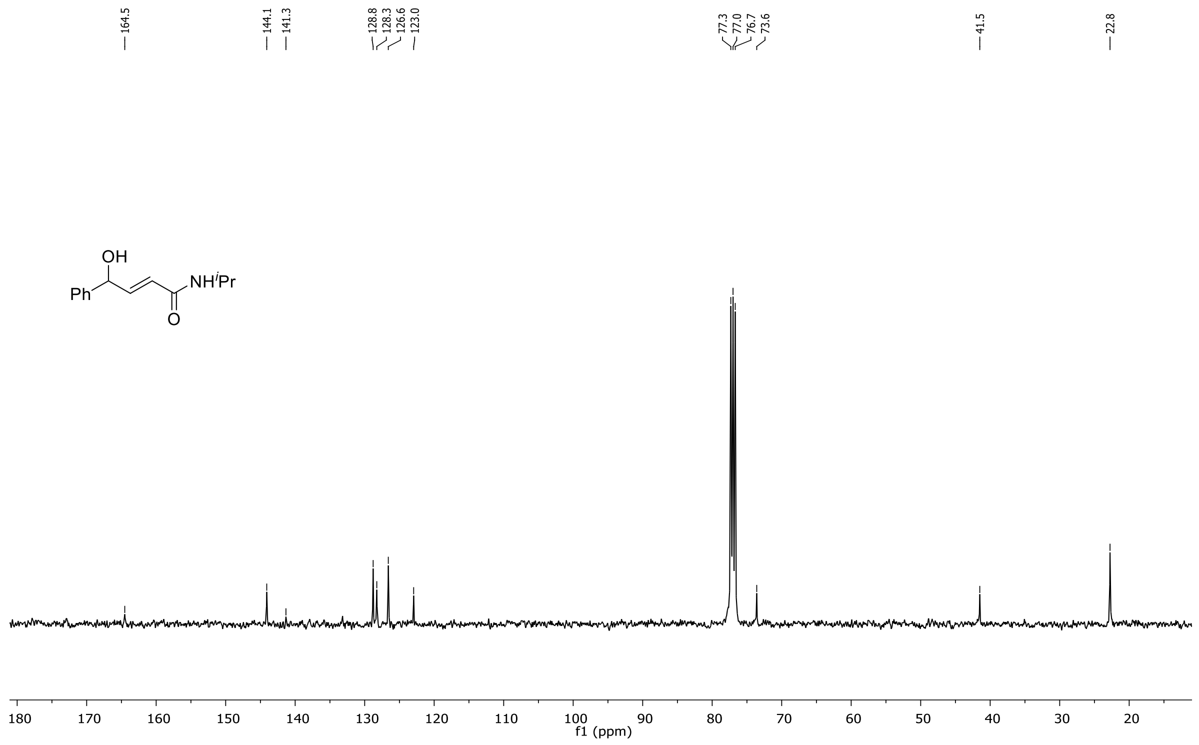

^1H NMR (400 MHz, $CDCl_3$) of **4a**

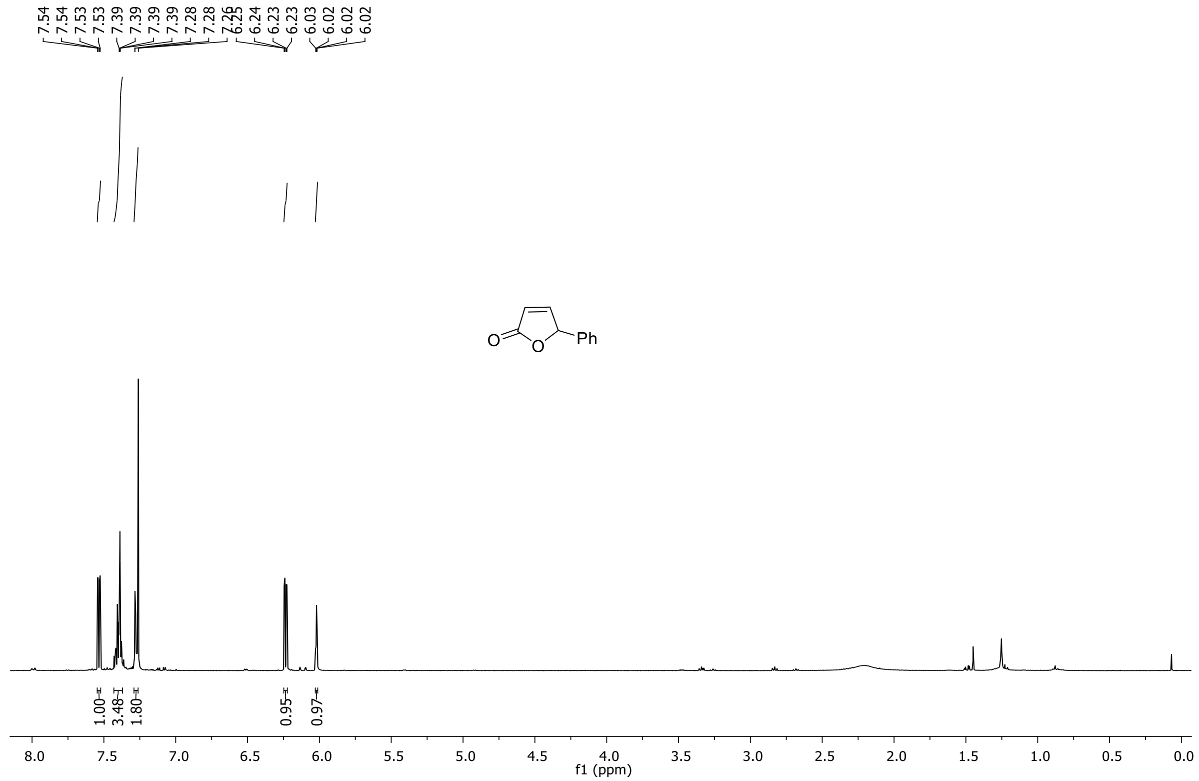

^{1}H NMR (400 MHz, $CDCl_3$) **4b**

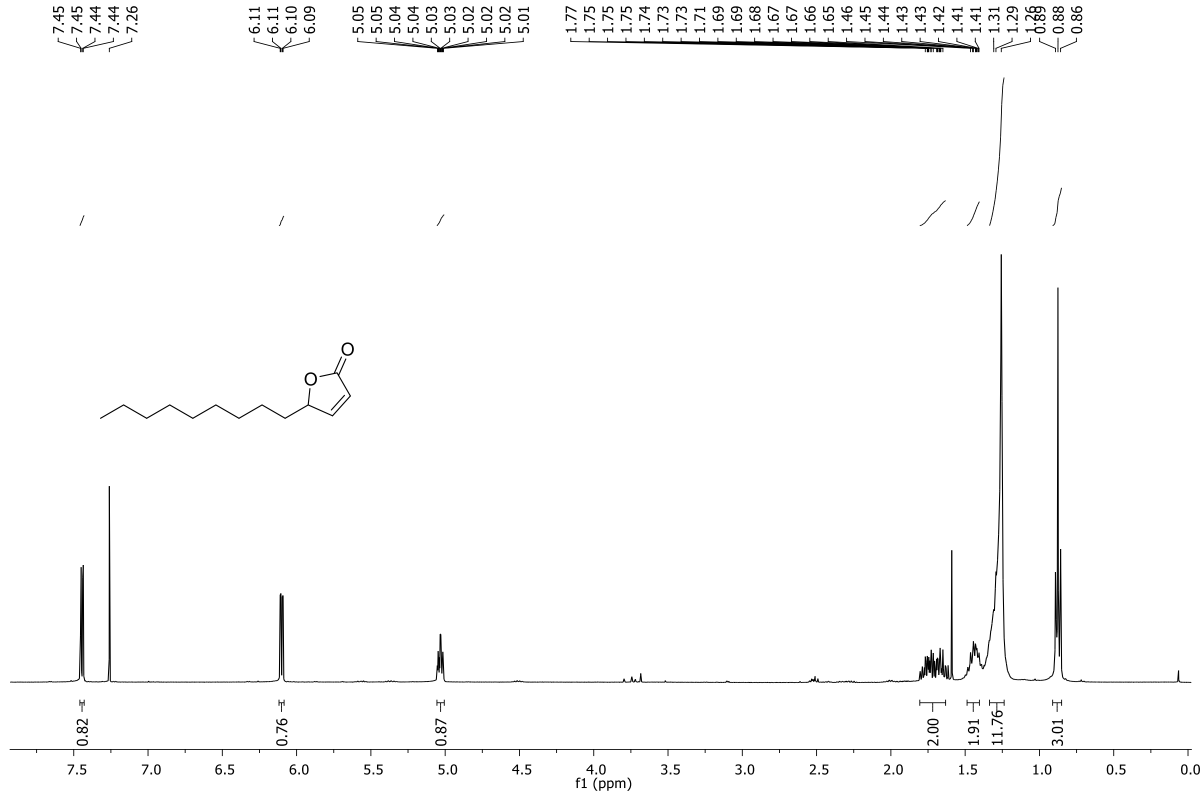

^{1}H NMR (400 MHz, $CDCl_3$) of **(*S*)-4c**

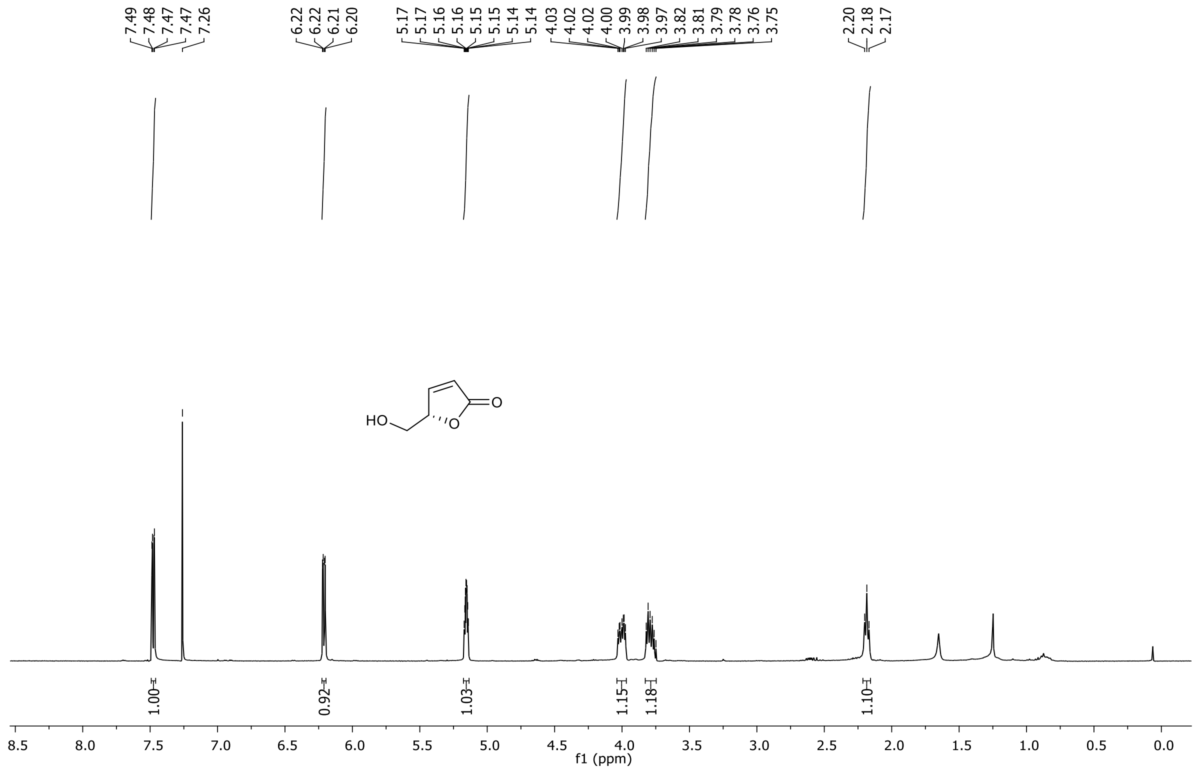

^{1}H NMR (400 MHz, Acetone-d6) of **4d**

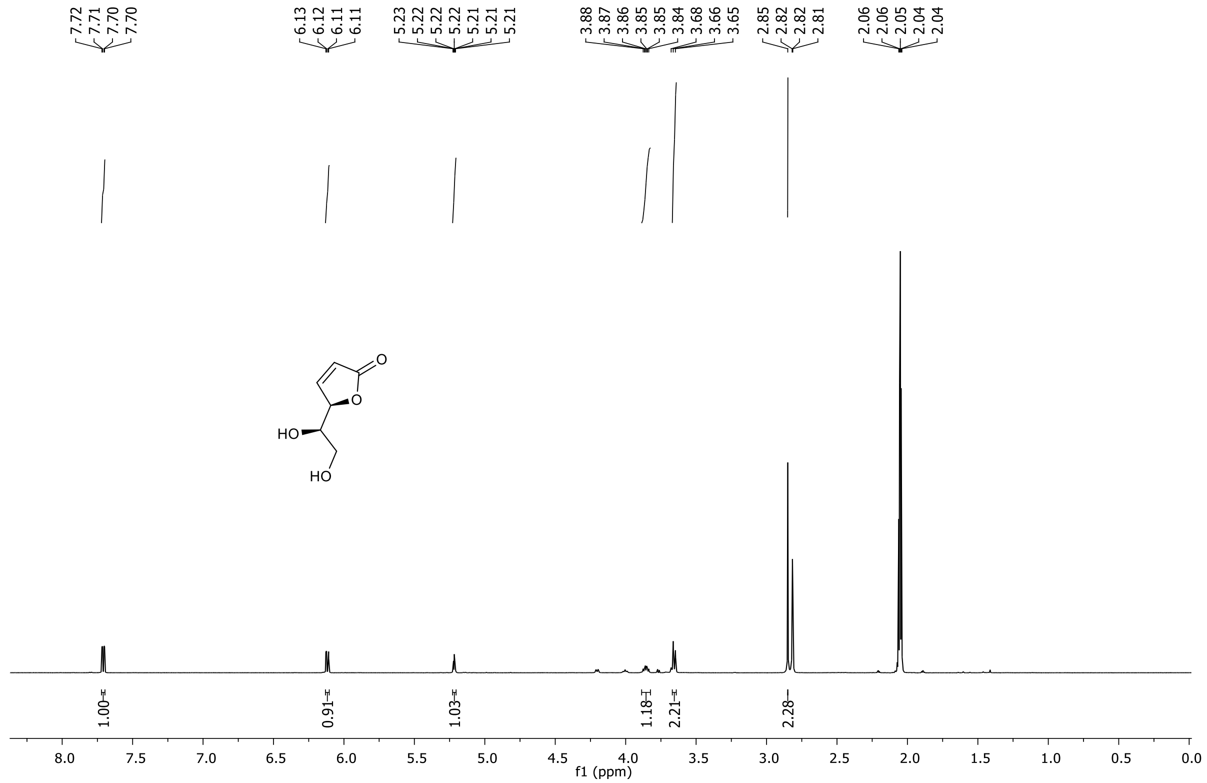

^{1}H NMR (400 MHz, $CDCl_3$) of **4e**

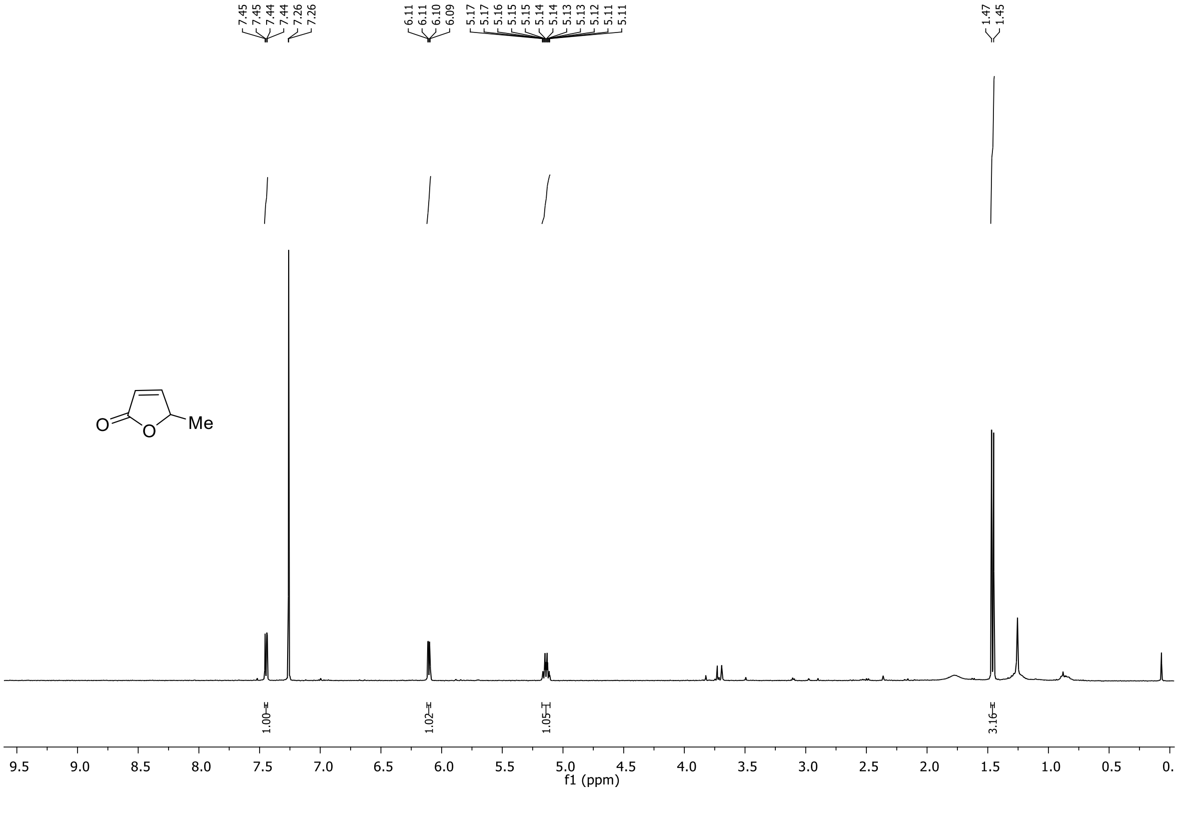

^{1}H NMR (400 MHz, $CDCl_3$) of **4f**

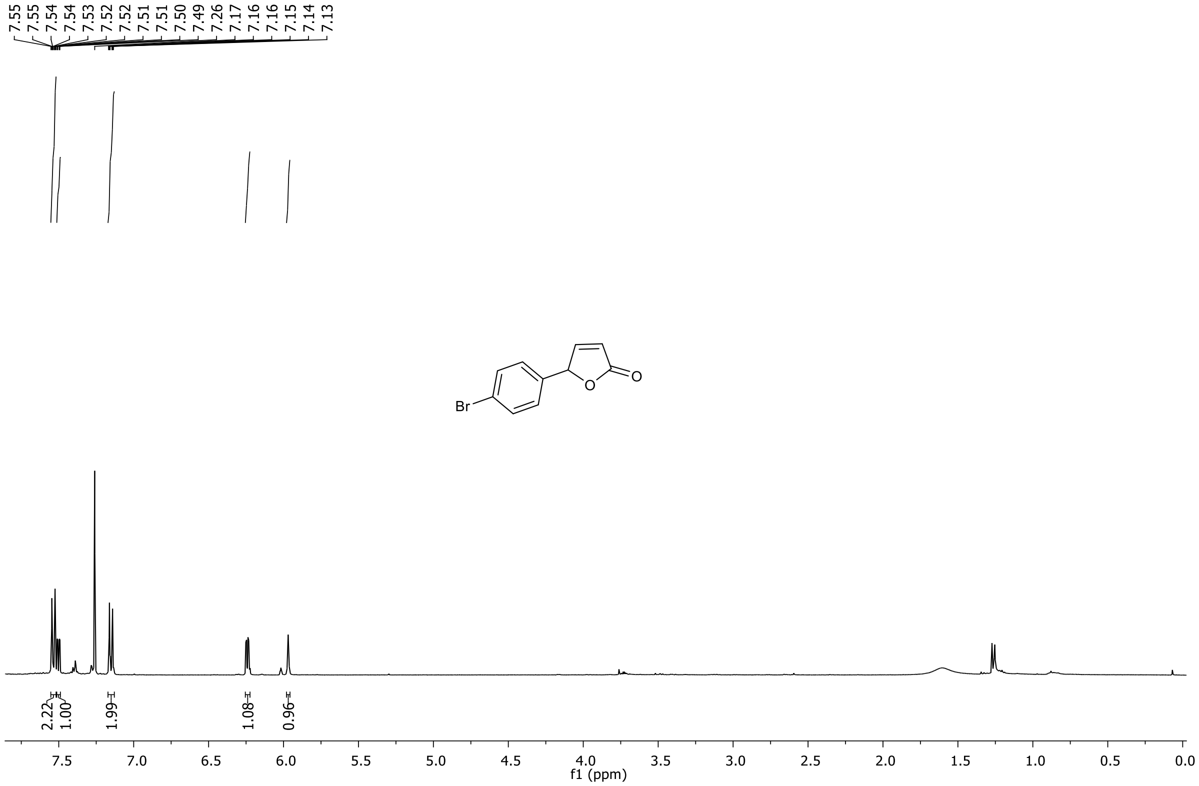

^{1}H NMR (400 MHz, $CDCl_3$) of **4g**

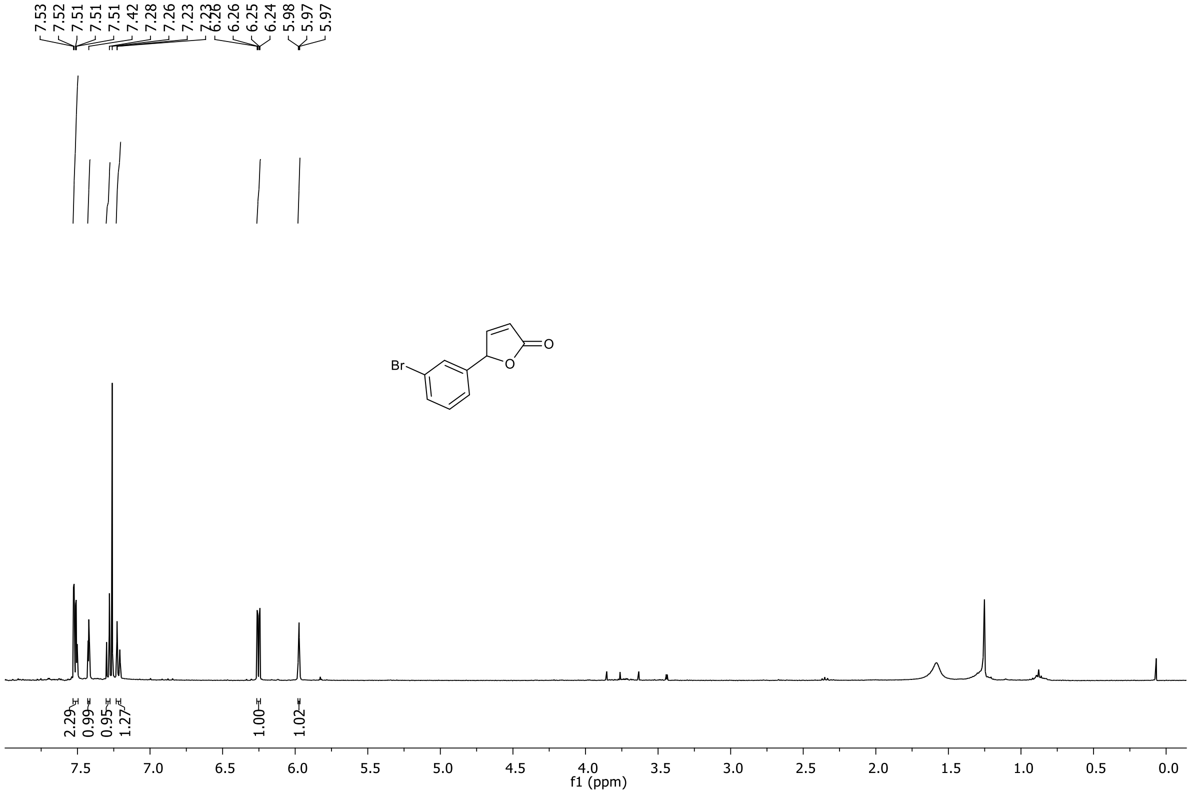

^{13}C NMR (100 MHz, $CDCl_3$) of **4g**

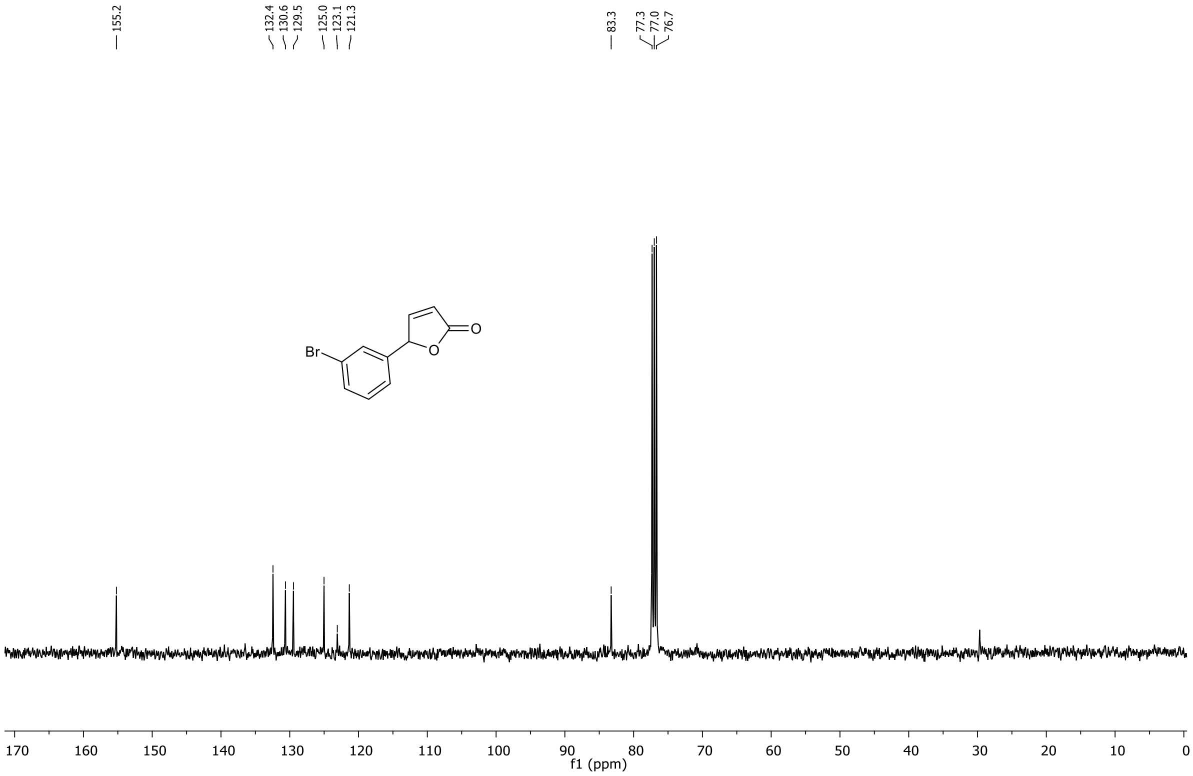

^{1}H NMR (400 MHz, $CDCl_3$) of **4h**

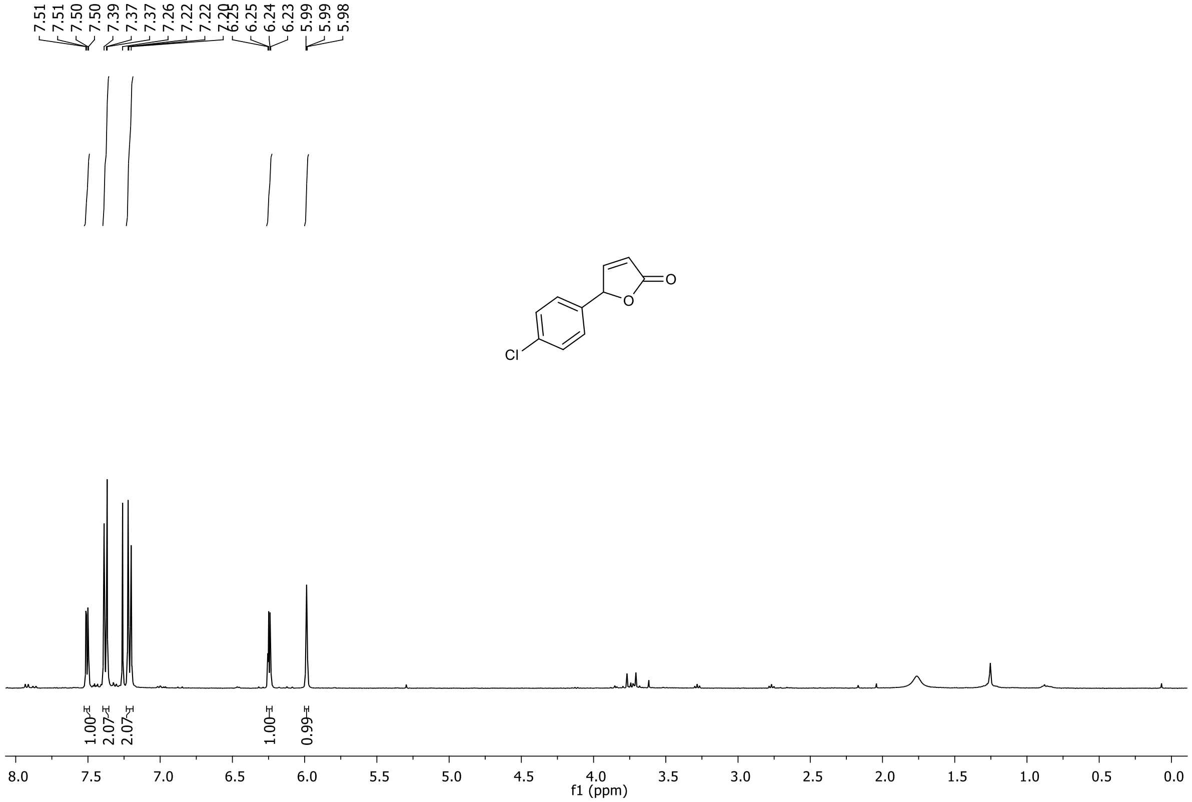

^{13}C NMR (100 MHz, $CDCl_3$) of **4h**

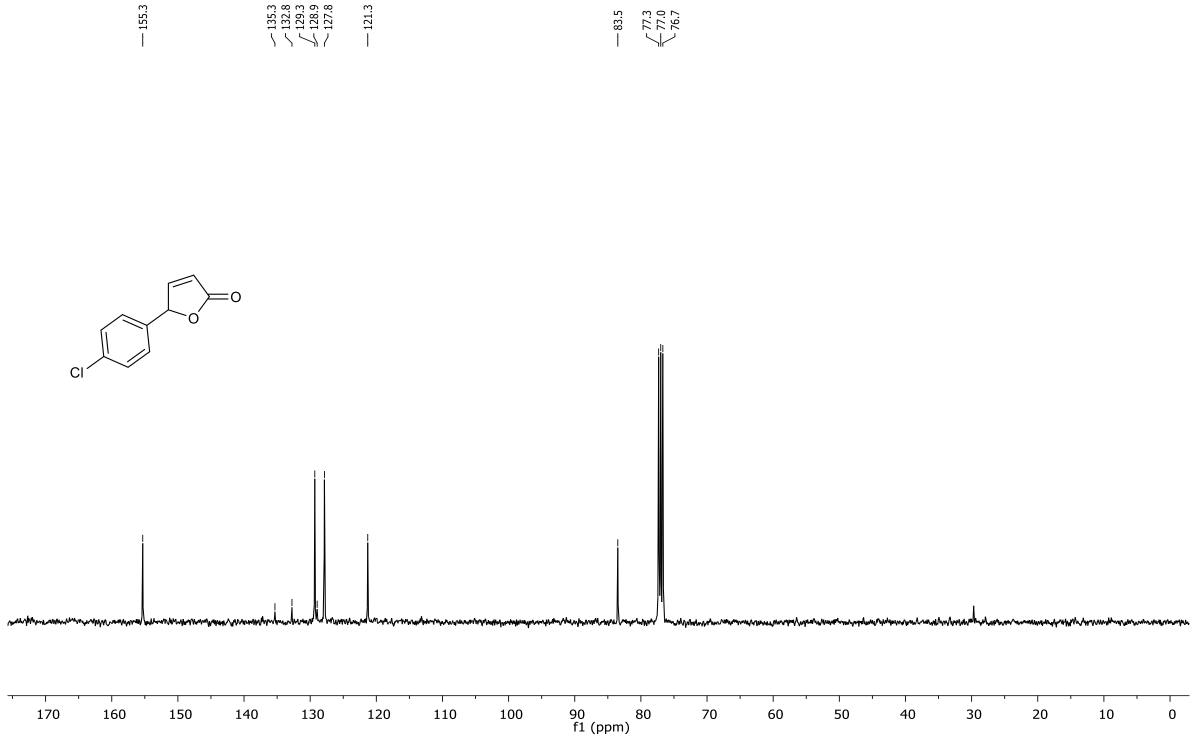

^{1}H NMR (400 MHz, $CDCl_3$) of **4j**

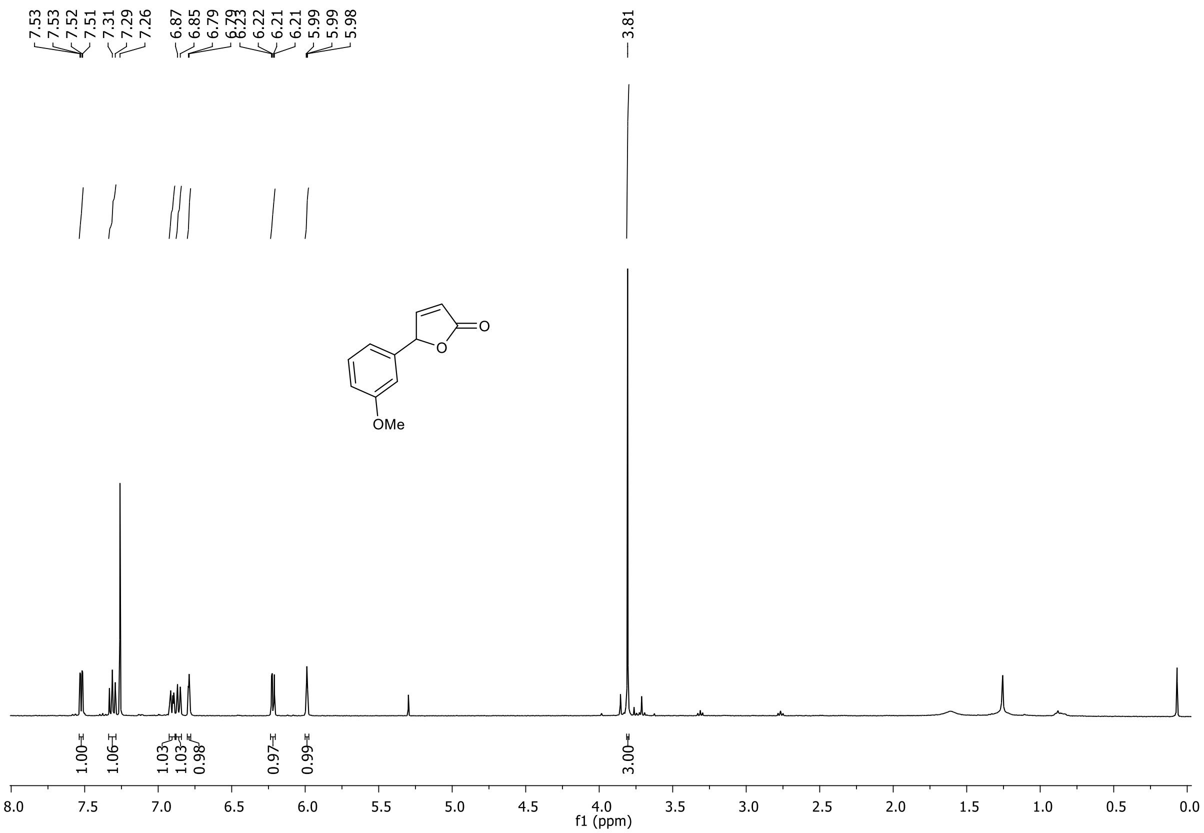

^{1}H NMR (400 MHz, $CDCl_3$) of **4k**

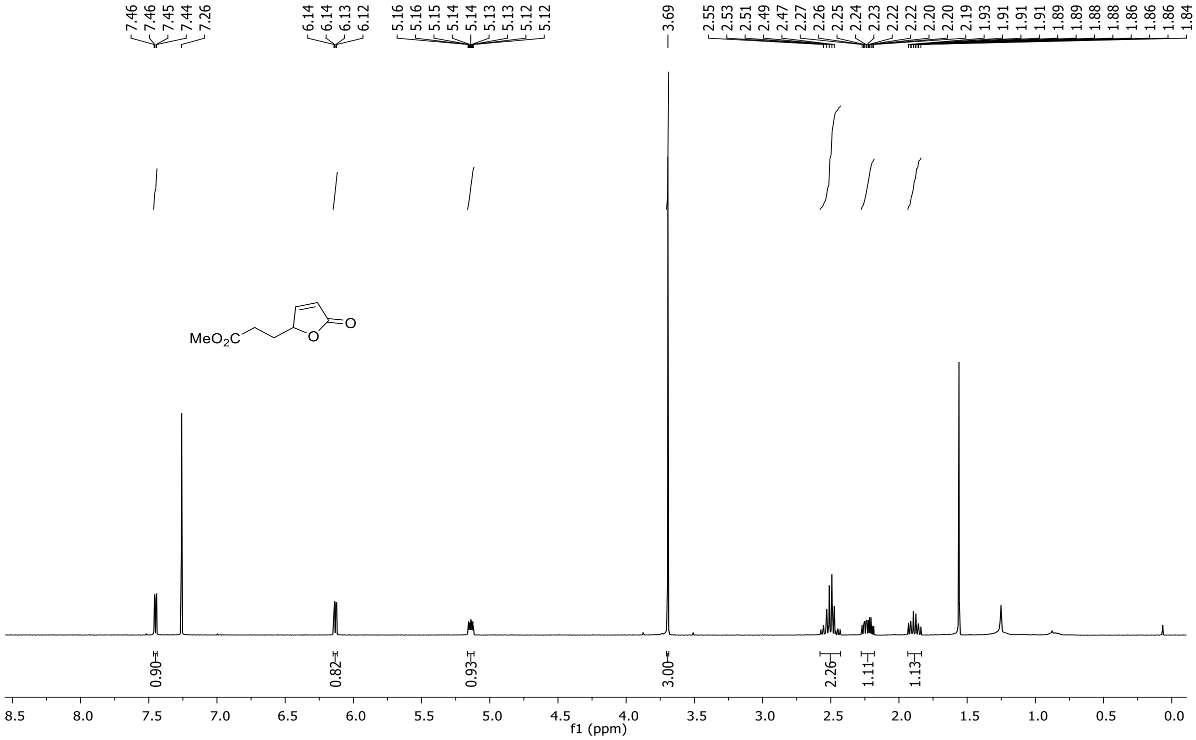

^{13}C NMR (100 MHz, $CDCl_3$) of **4k**

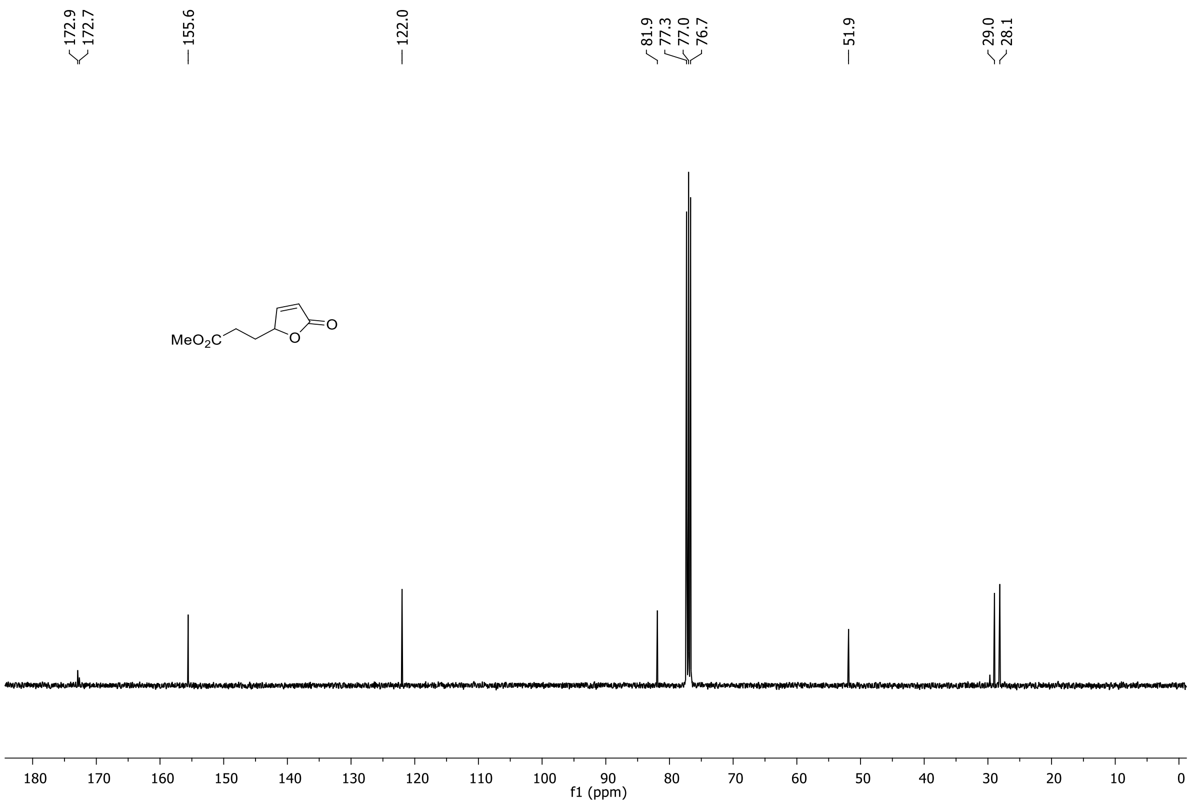

^{1}H NMR (400 MHz, $CDCl_3$) of **8**

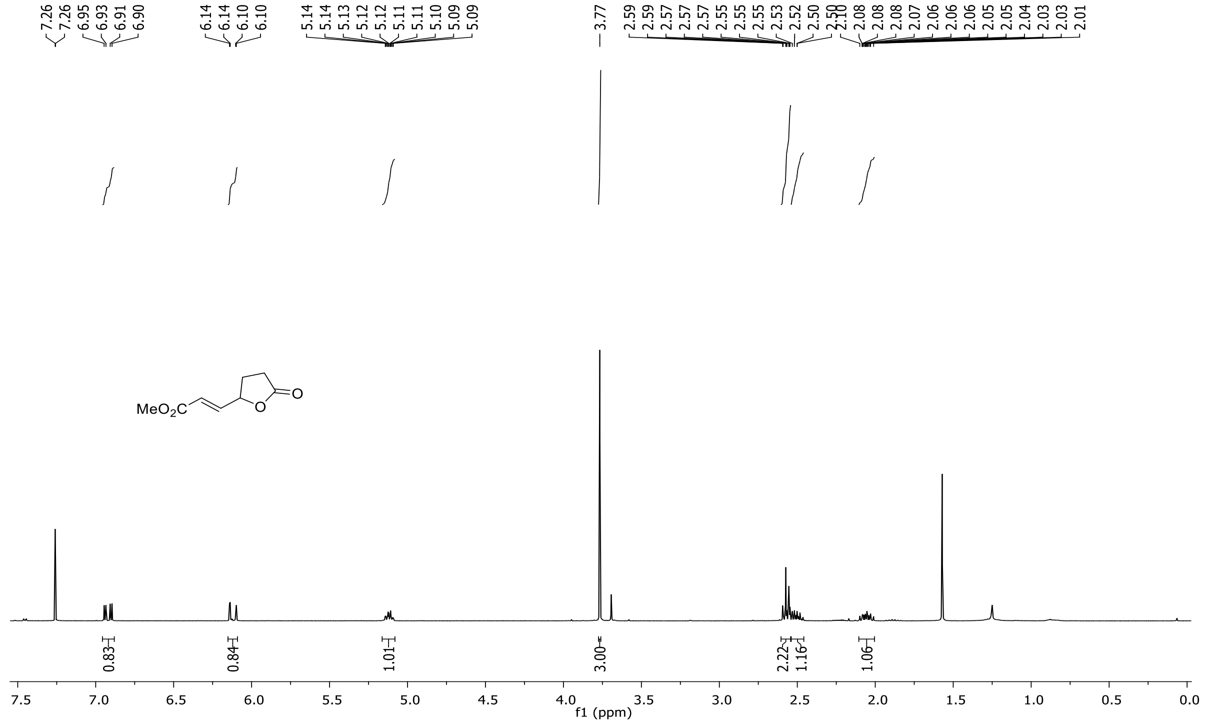

^{13}C NMR (100 MHz, $CDCl_3$) of **8**

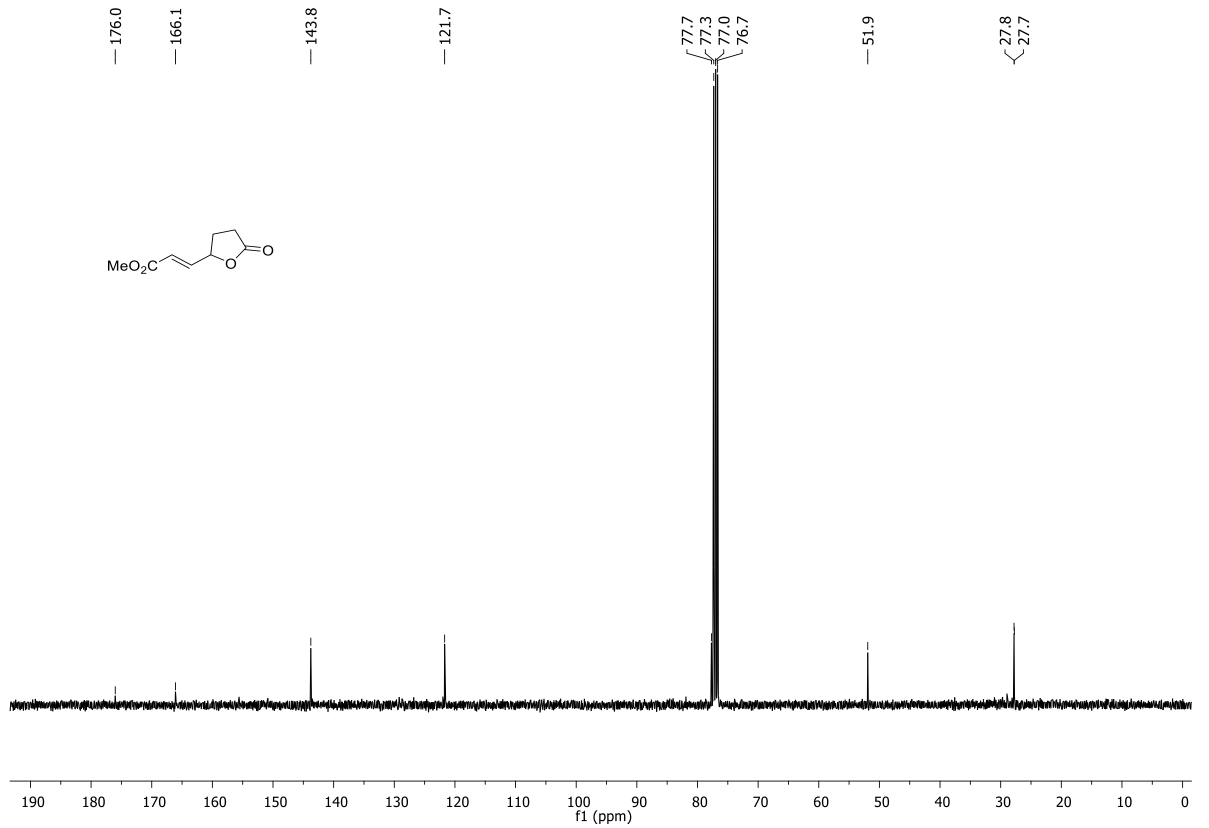

^{1}H NMR (400 MHz, $CDCl_3$) of **4l**

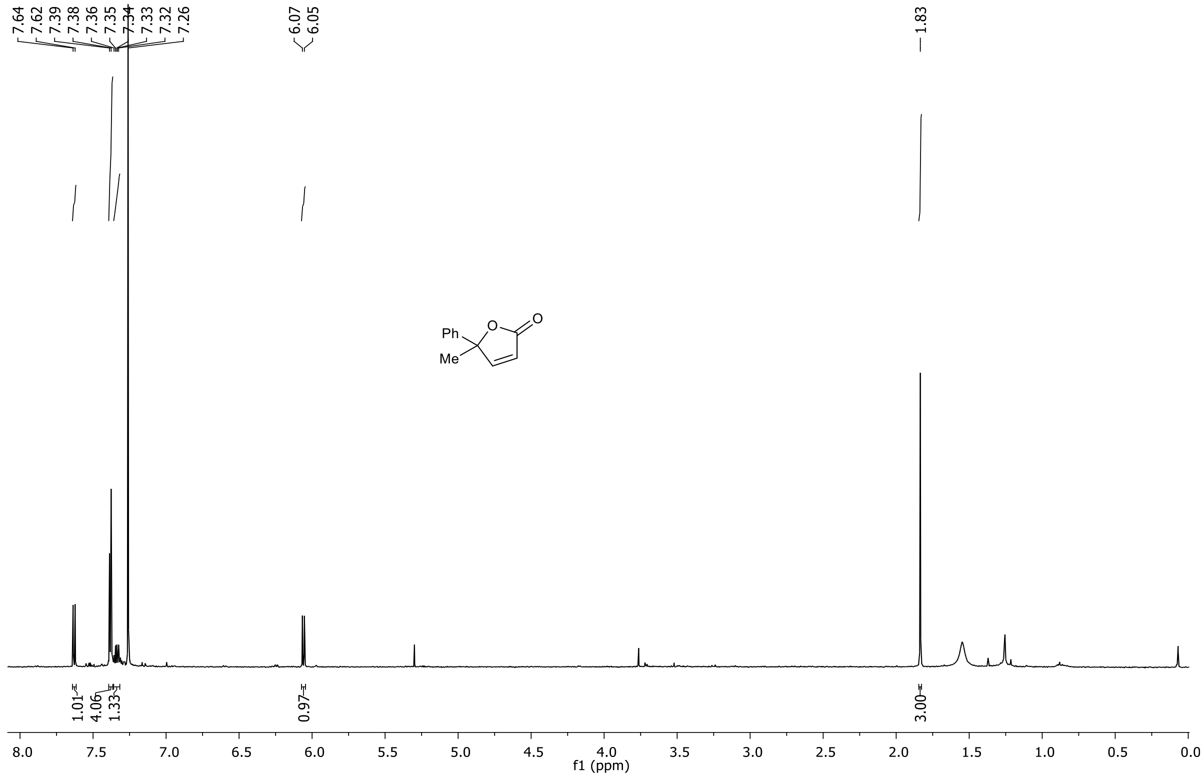

^{1}H NMR (400 MHz, $CDCl_3$) of **4m**

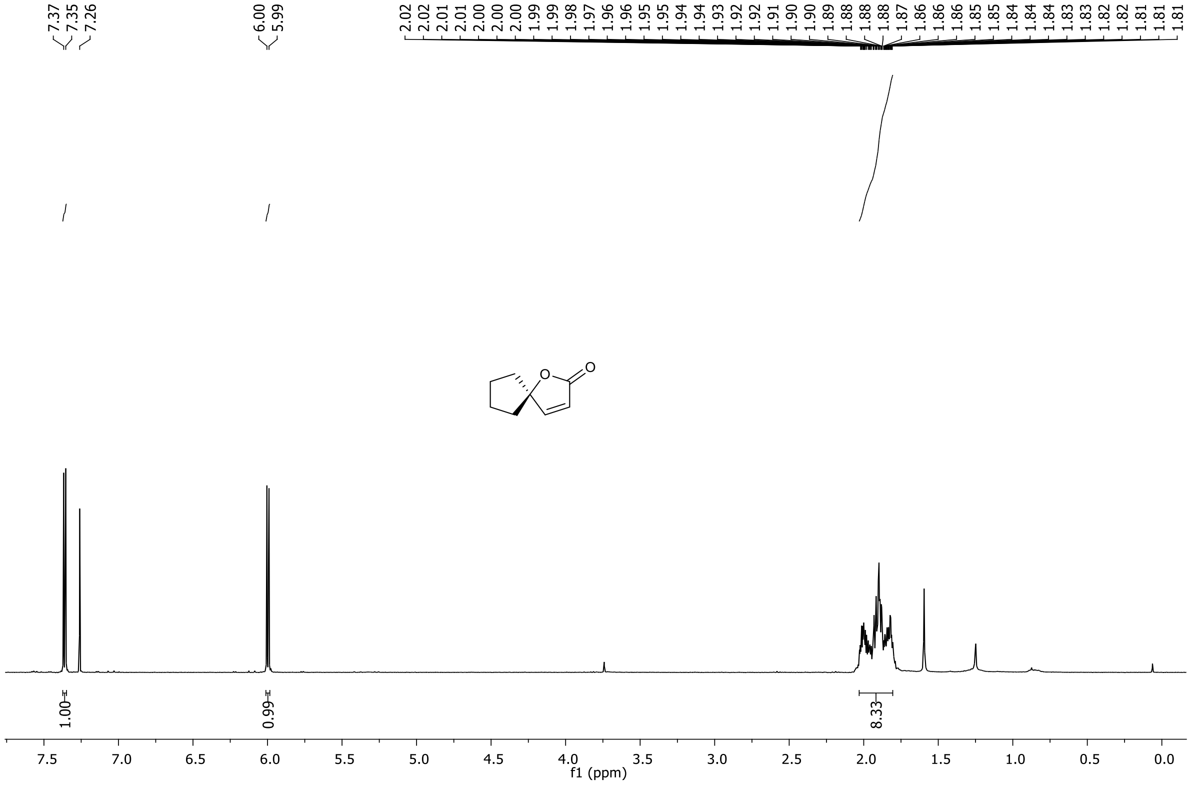

^{1}H NMR (400 MHz, $CDCl_3$) of **4n**

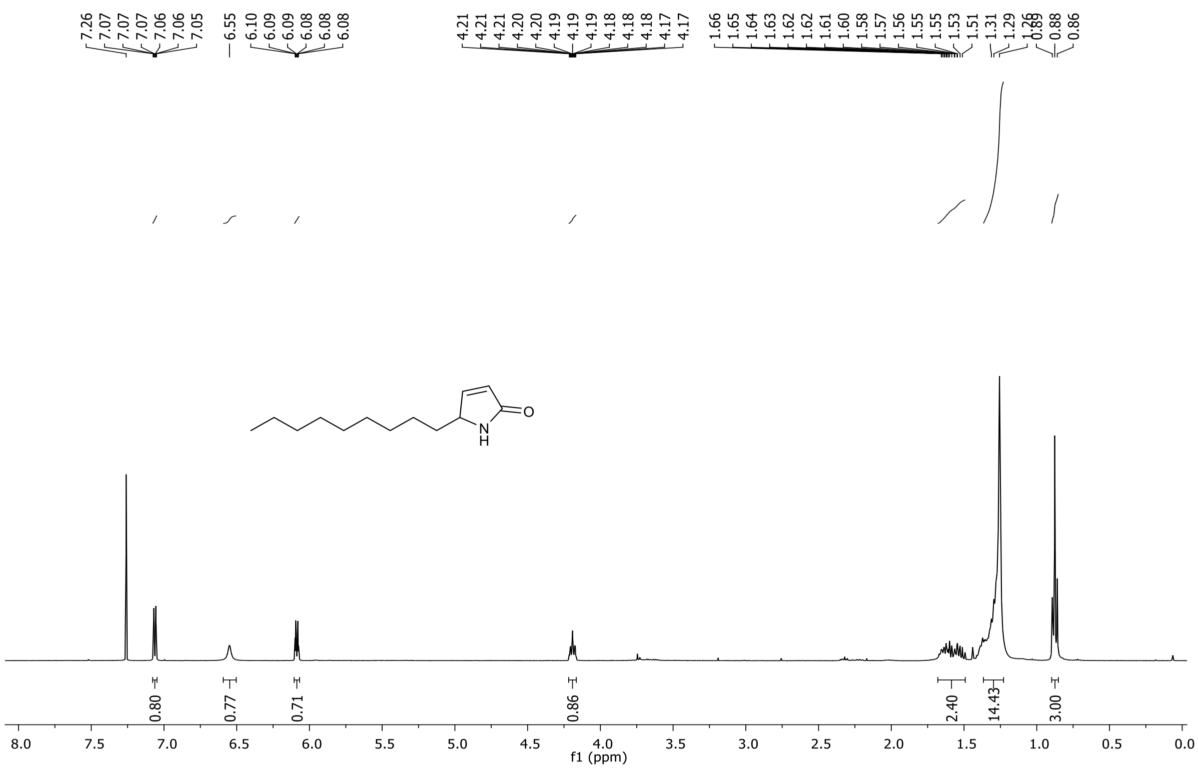

^{13}C NMR (100 MHz, $CDCl_3$) of **4n**

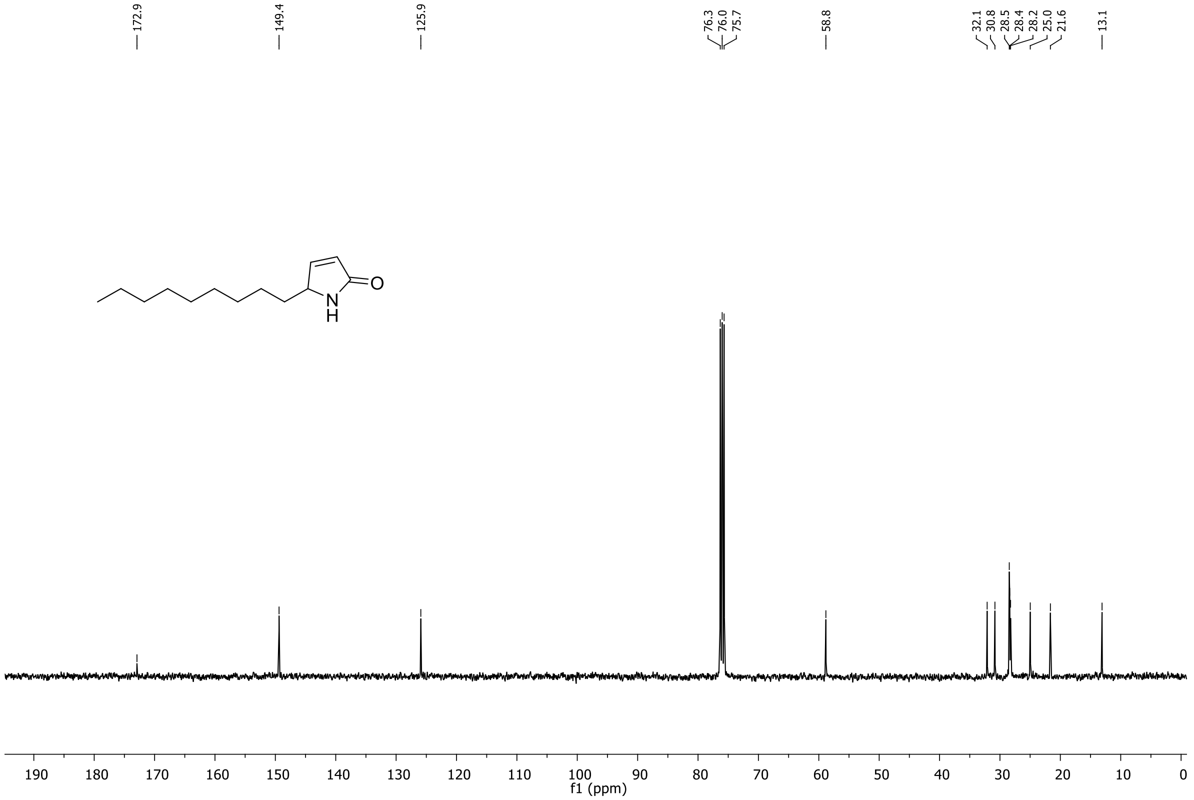

^{1}H NMR (400 MHz, CDCl$_3$) of **5a**

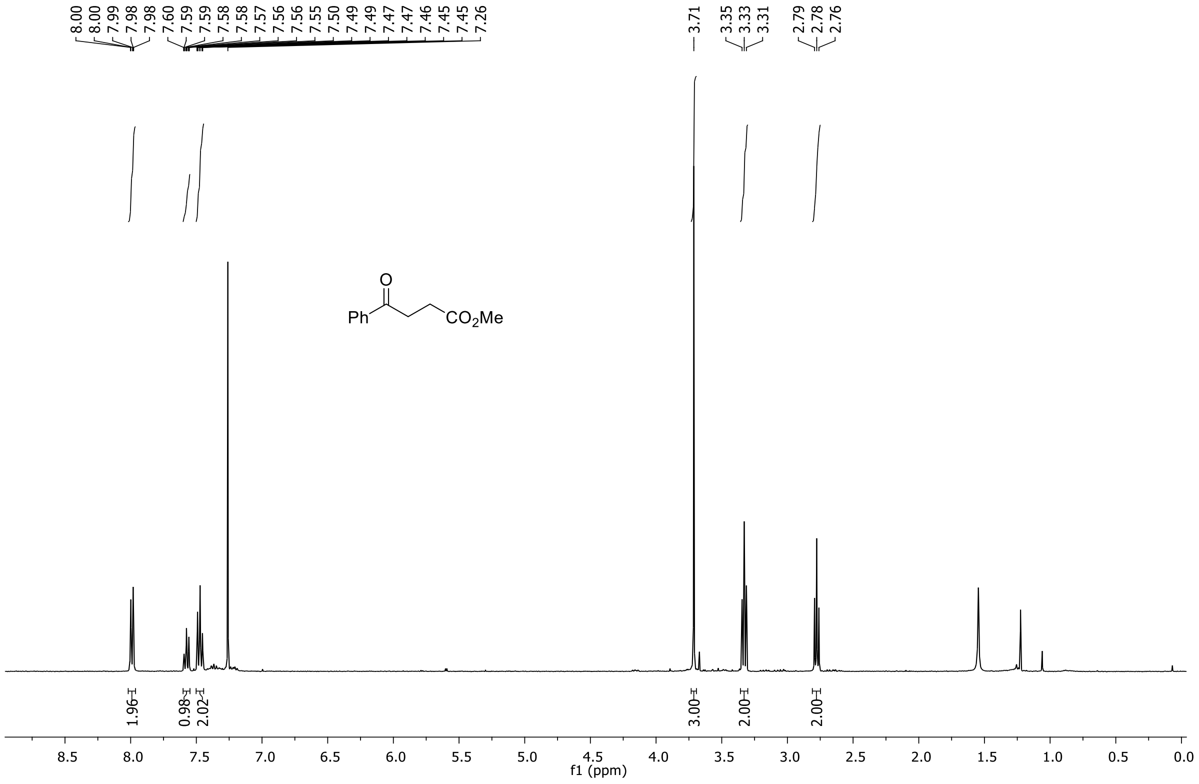

^{1}H NMR (400 MHz, CDCl$_3$) of **5b**

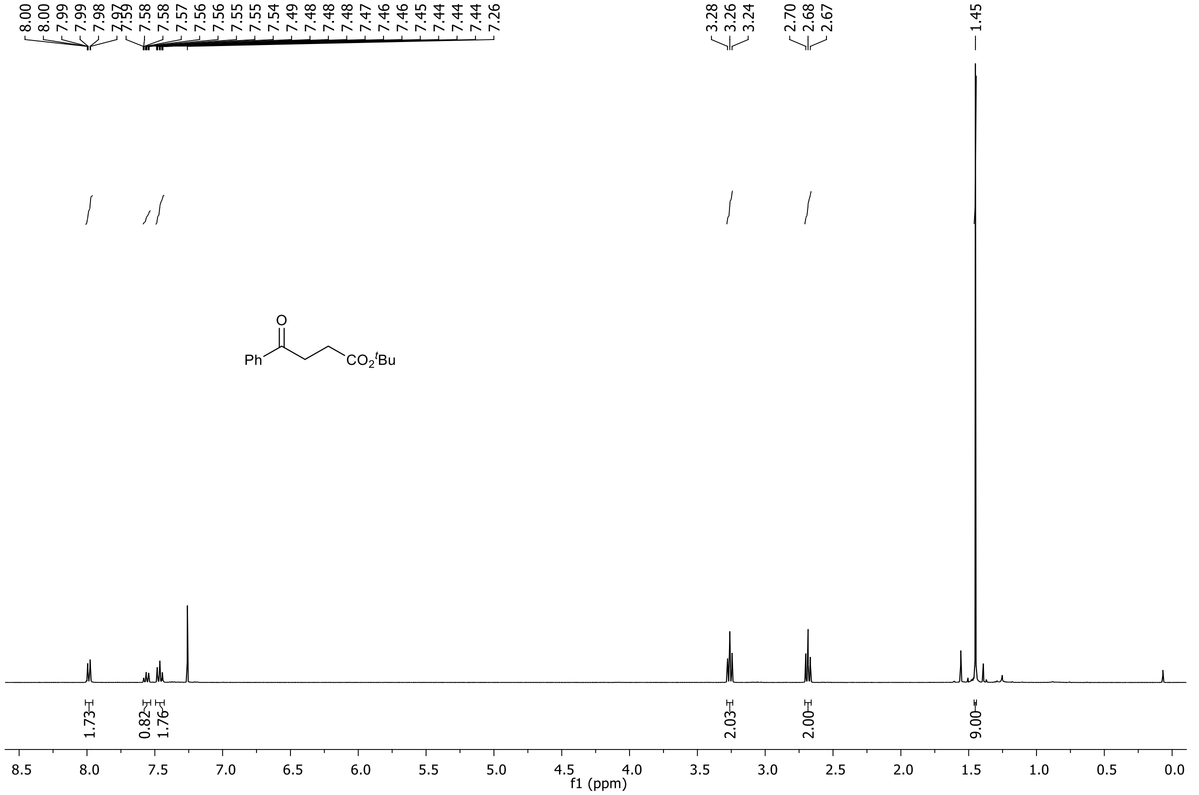

^{1}H NMR (400 MHz, $CDCl_3$) of **5c**

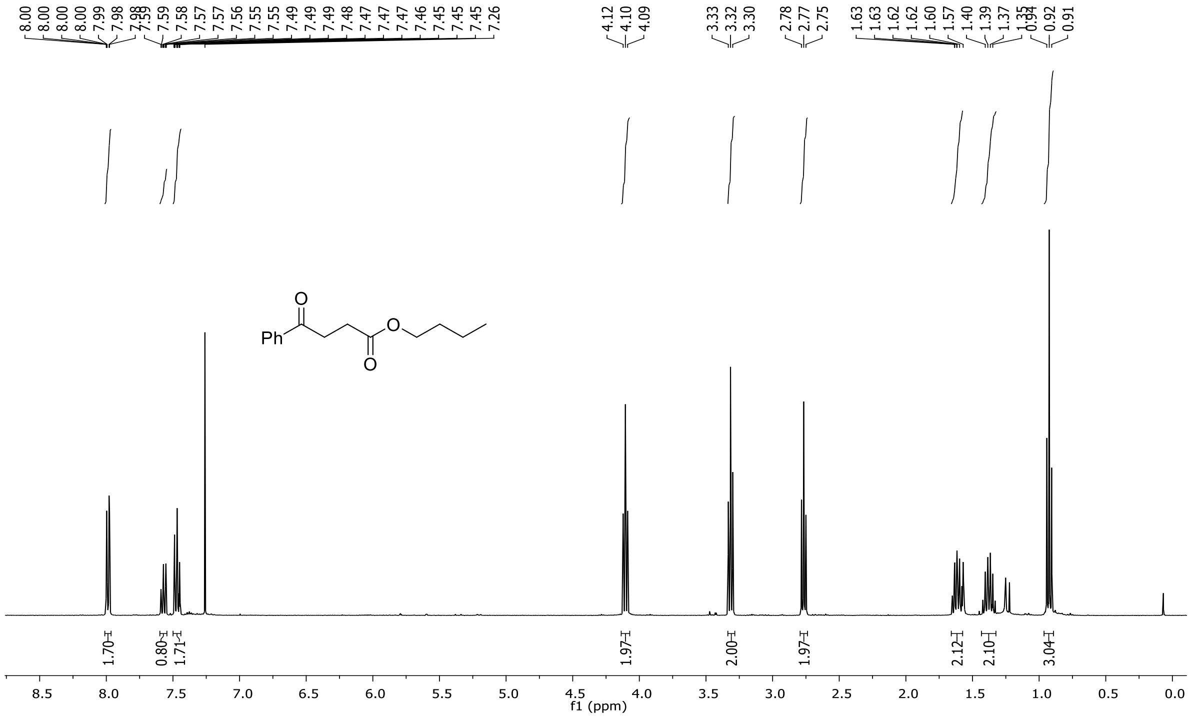

^{1}H NMR (400 MHz, $CDCl_3$) of **5d**

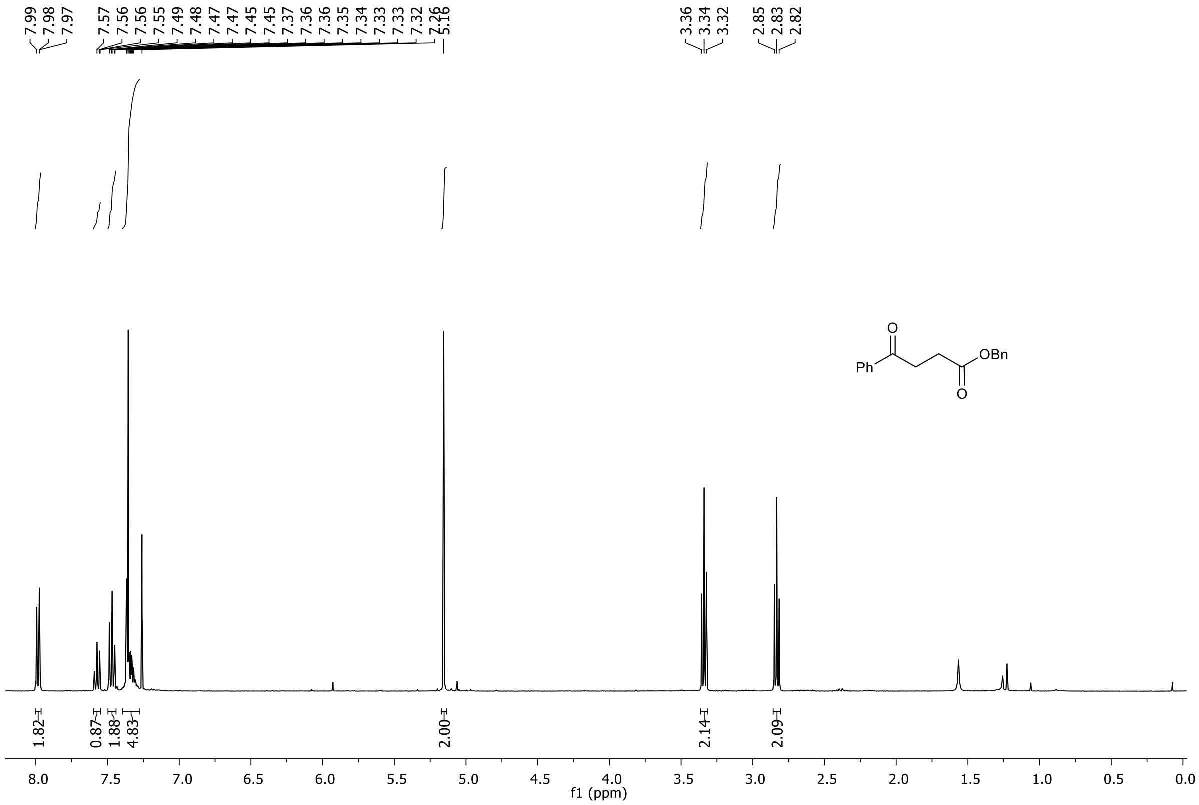

^{1}H NMR (400 MHz, $CDCl_3$) of **5h**

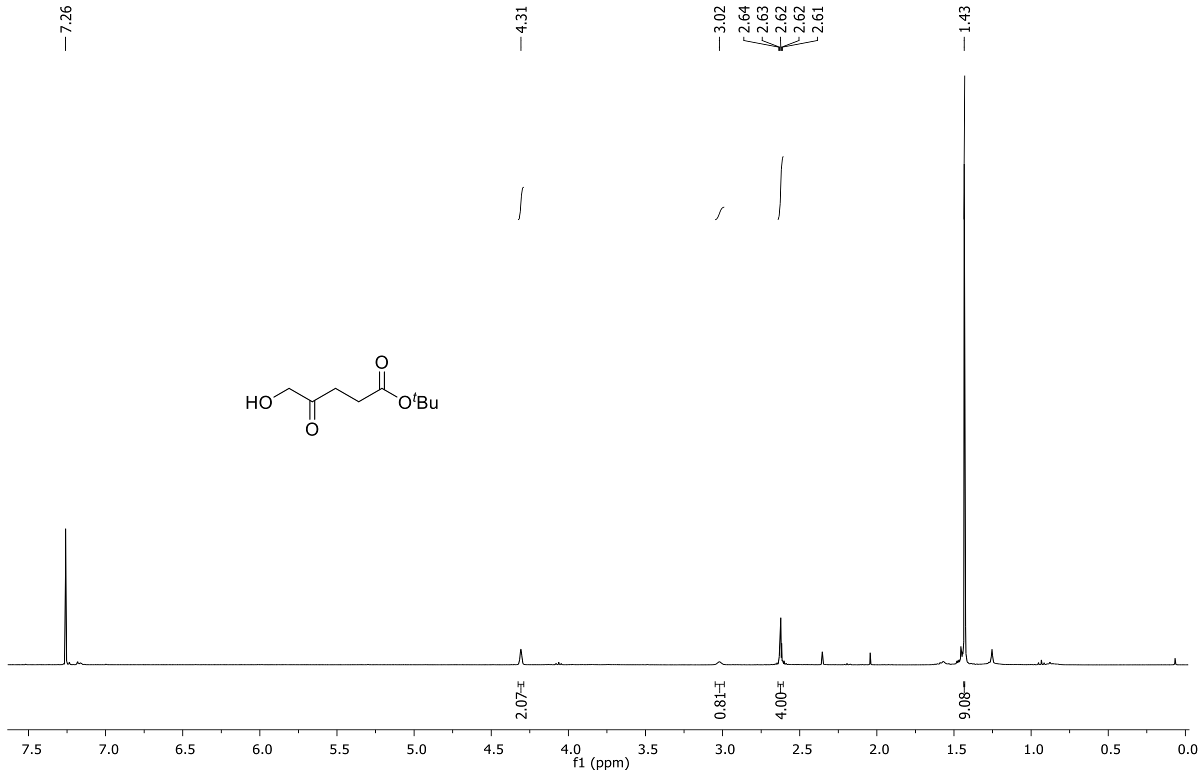

^{1}H NMR (400 MHz, $CDCl_3$) of **5i**

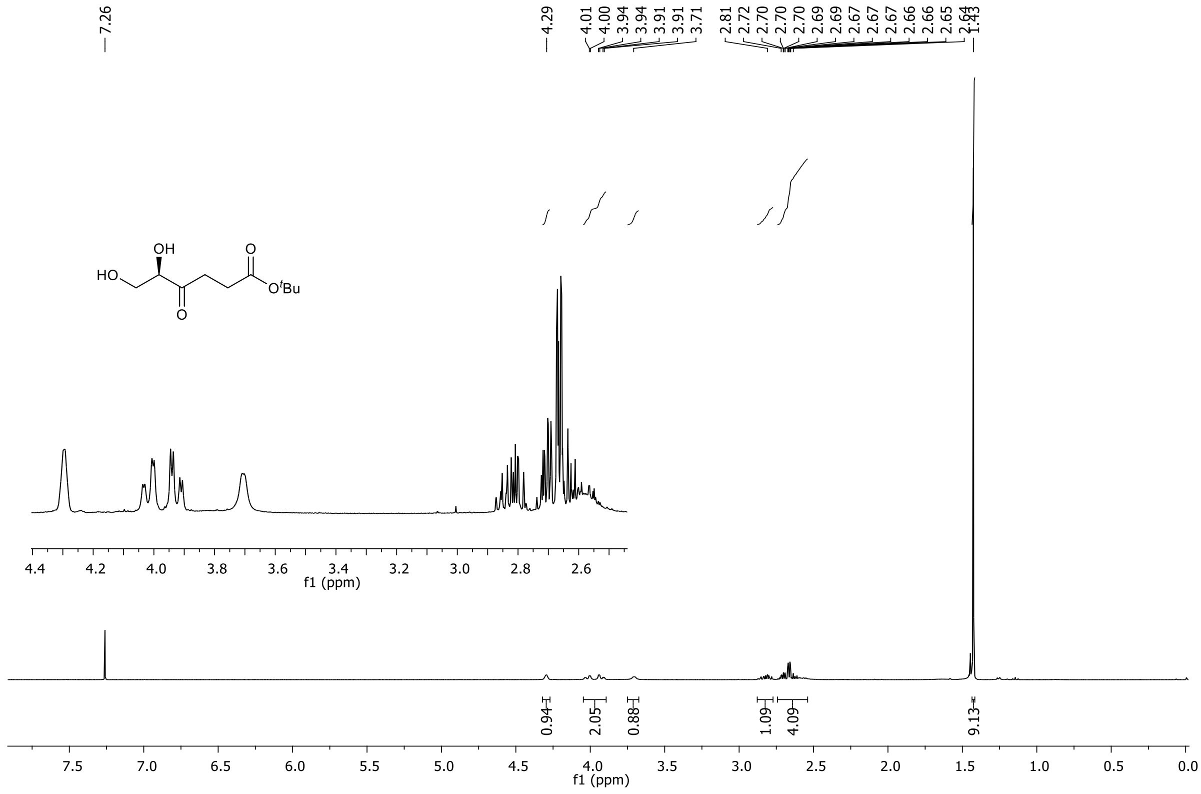

^{13}C NMR (100 MHz, $CDCl_3$) of **5i**

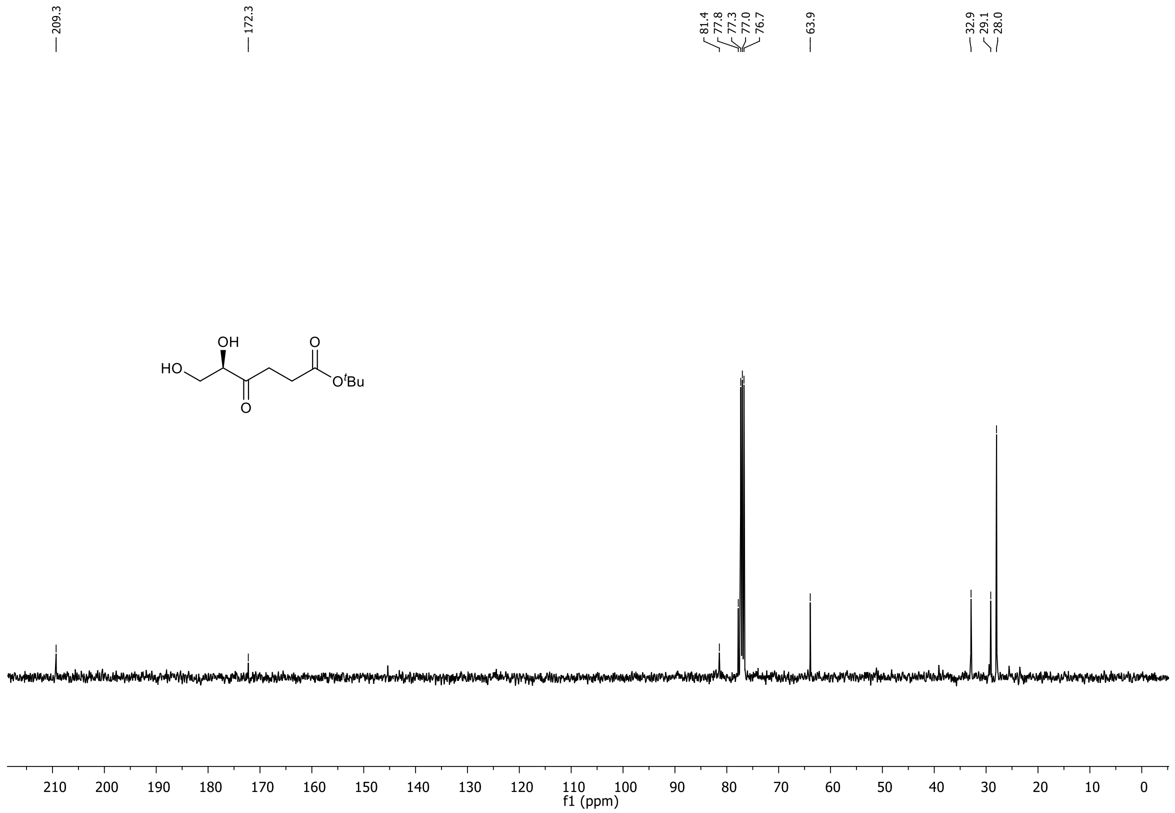

^{1}H NMR (400 MHz, $CDCl_3$) of **5j**

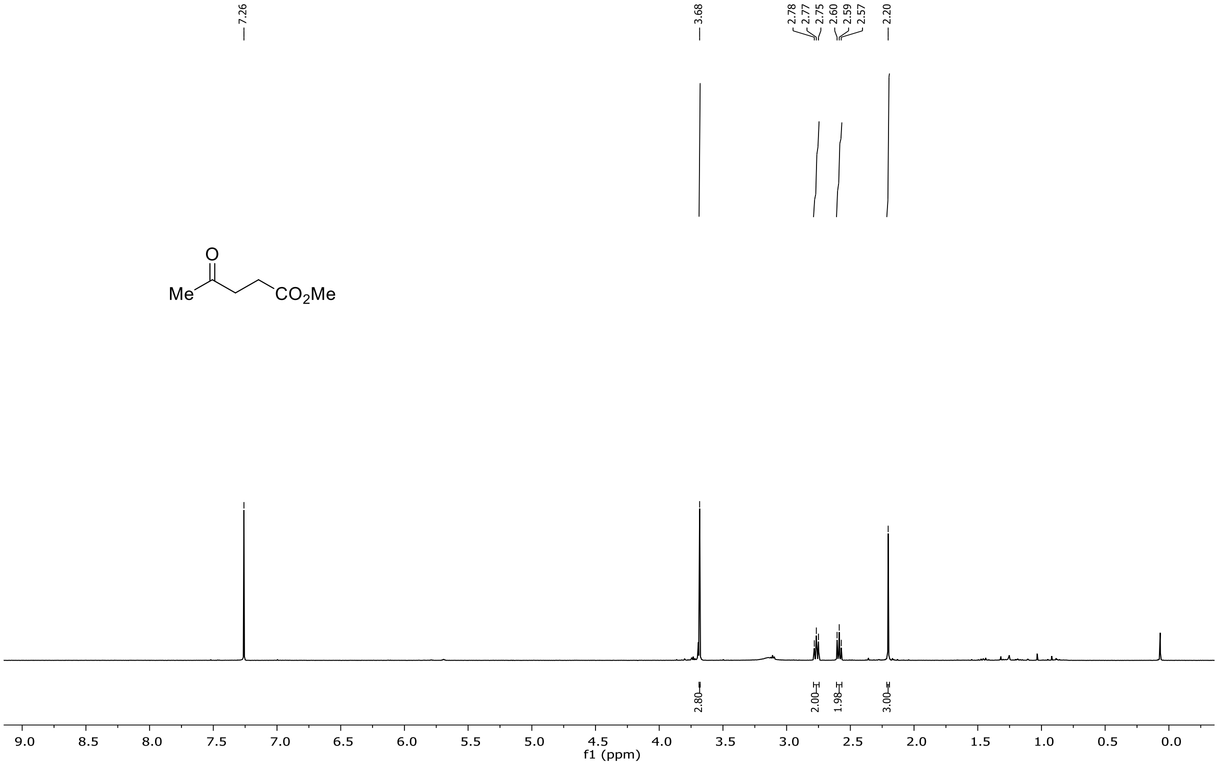

^{1}H NMR (400 MHz, $CDCl_3$) **5k**

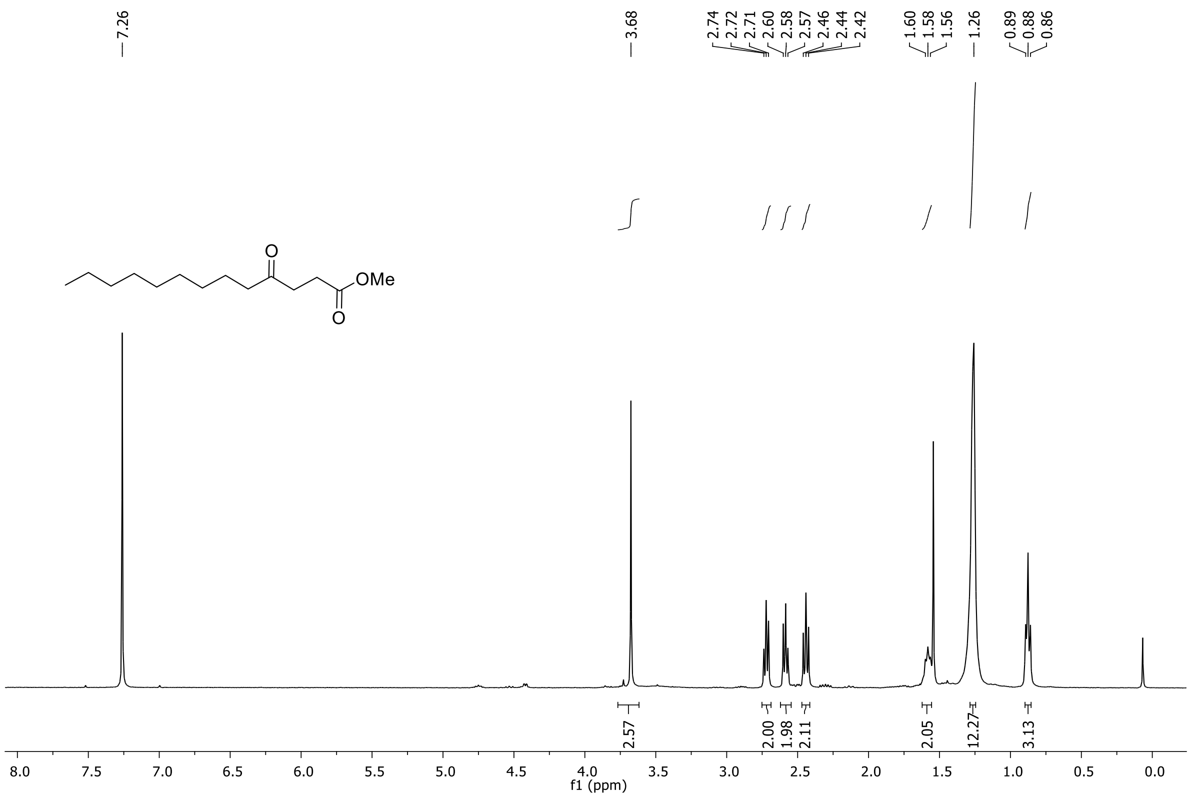

^{13}C NMR (100 MHz, $CDCl_3$) **5k**

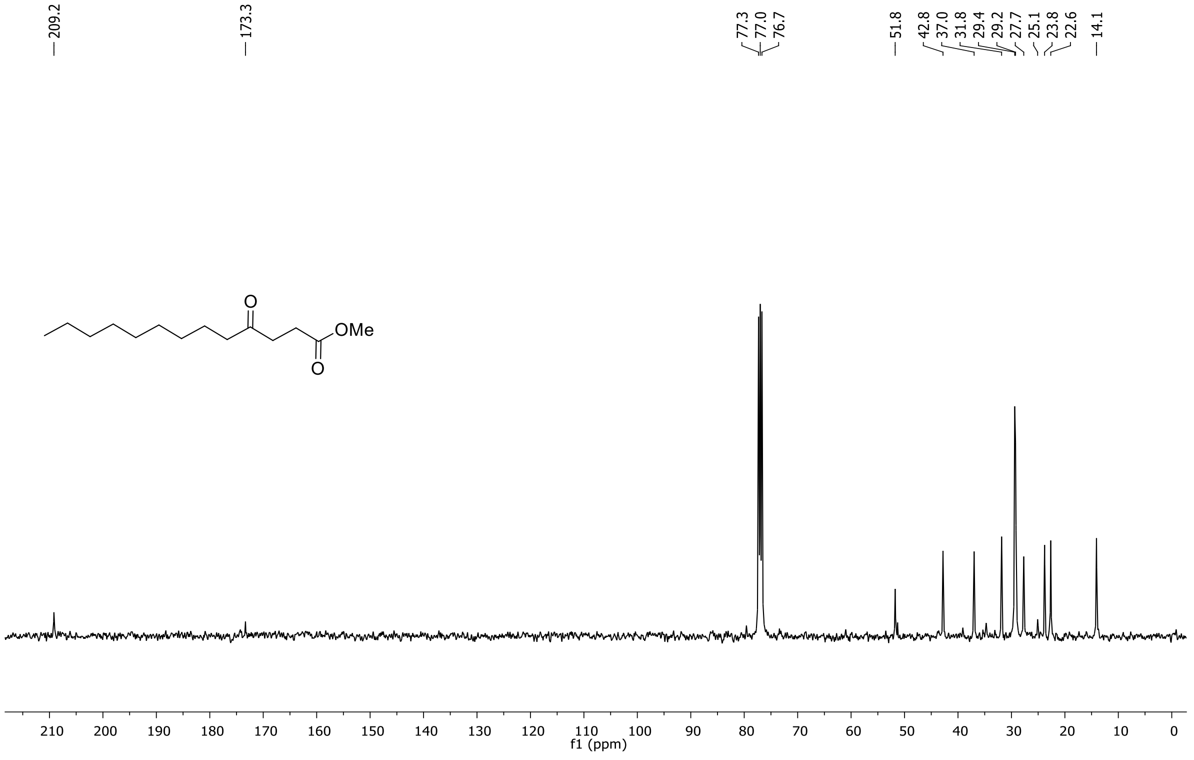

^{1}H NMR (400 MHz, $CDCl_3$) of **5l**

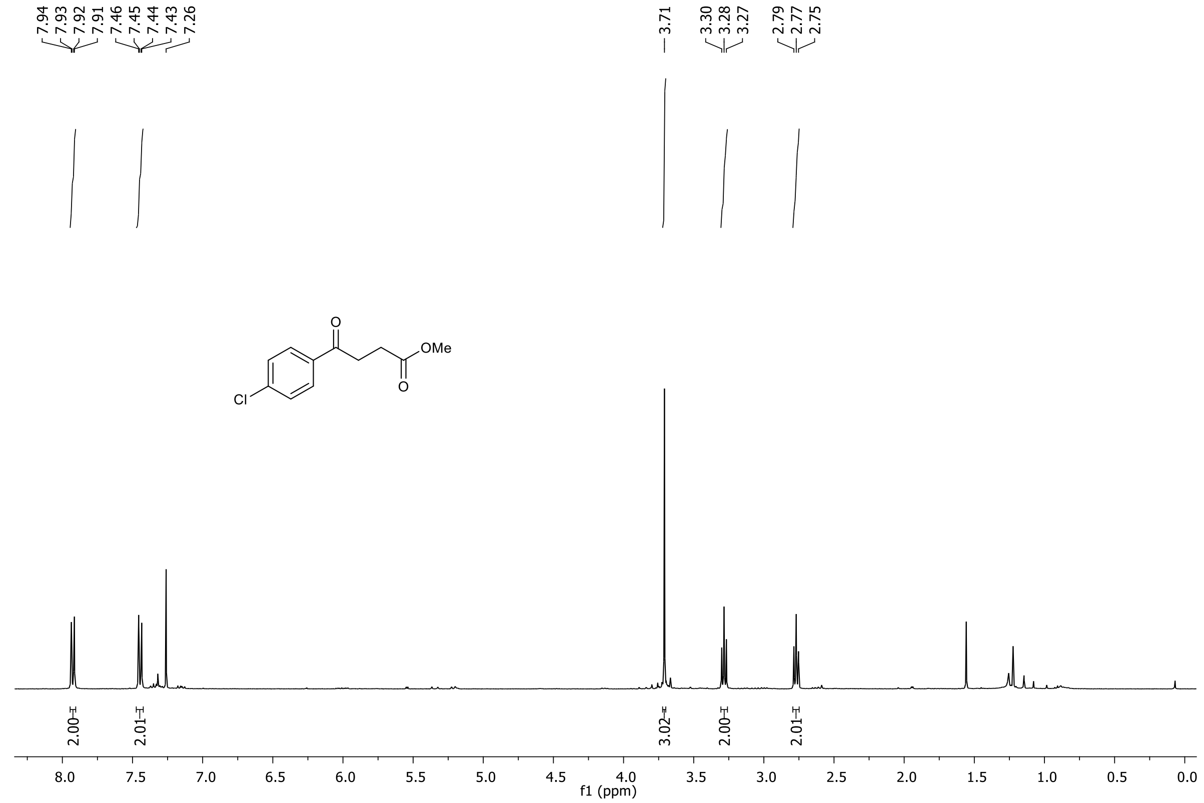

^{1}H NMR (400 MHz, $CDCl_3$) of **5m**

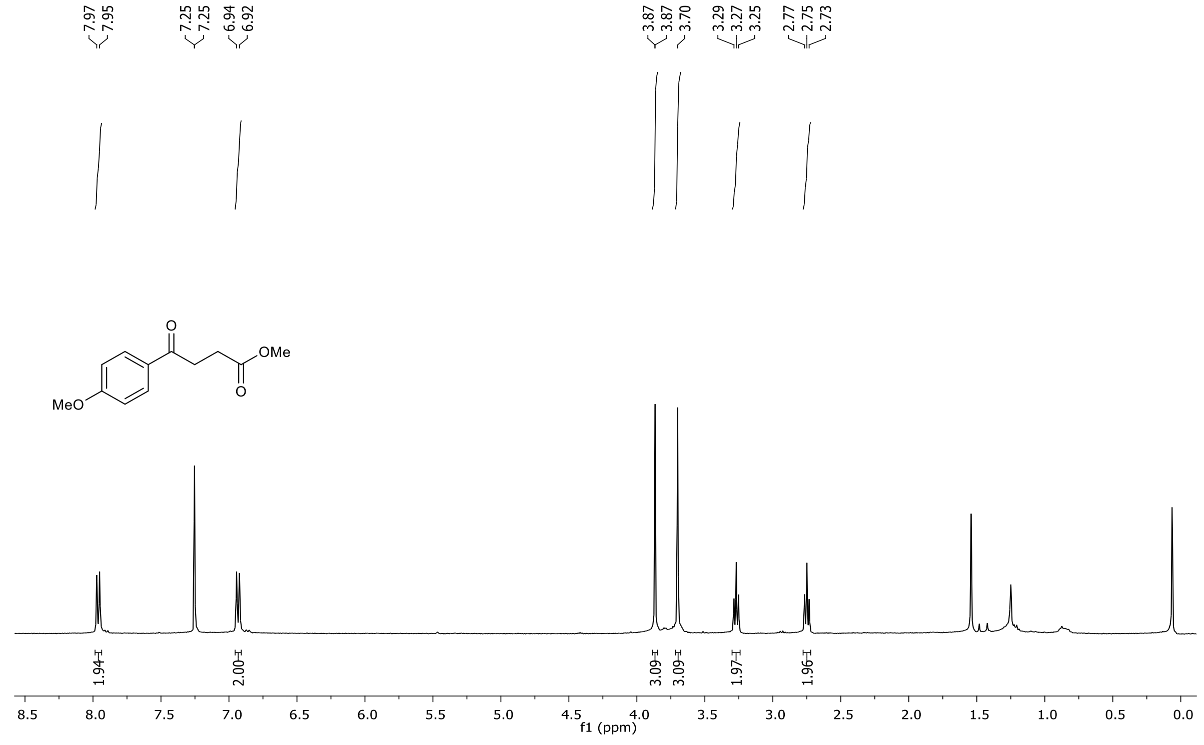

^{1}H NMR (400 MHz, CDCl$_3$) of **5n**

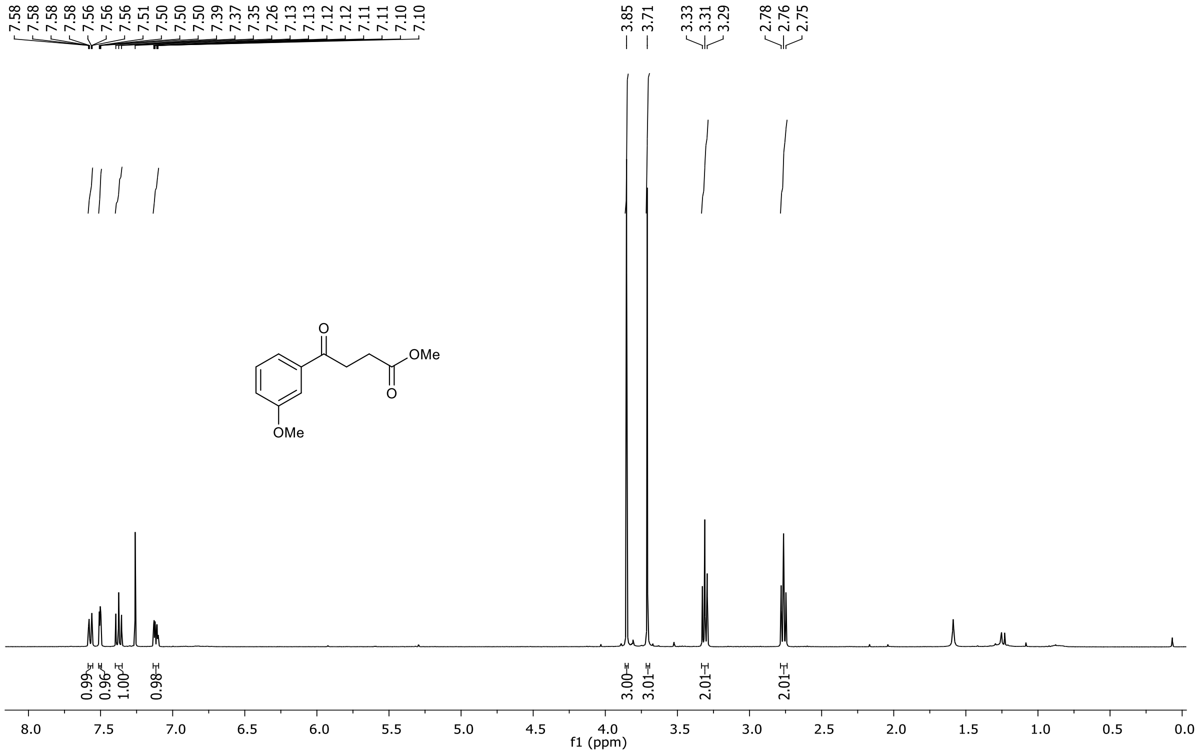

^{1}H NMR (400 MHz, CDCl$_3$) of **5o**

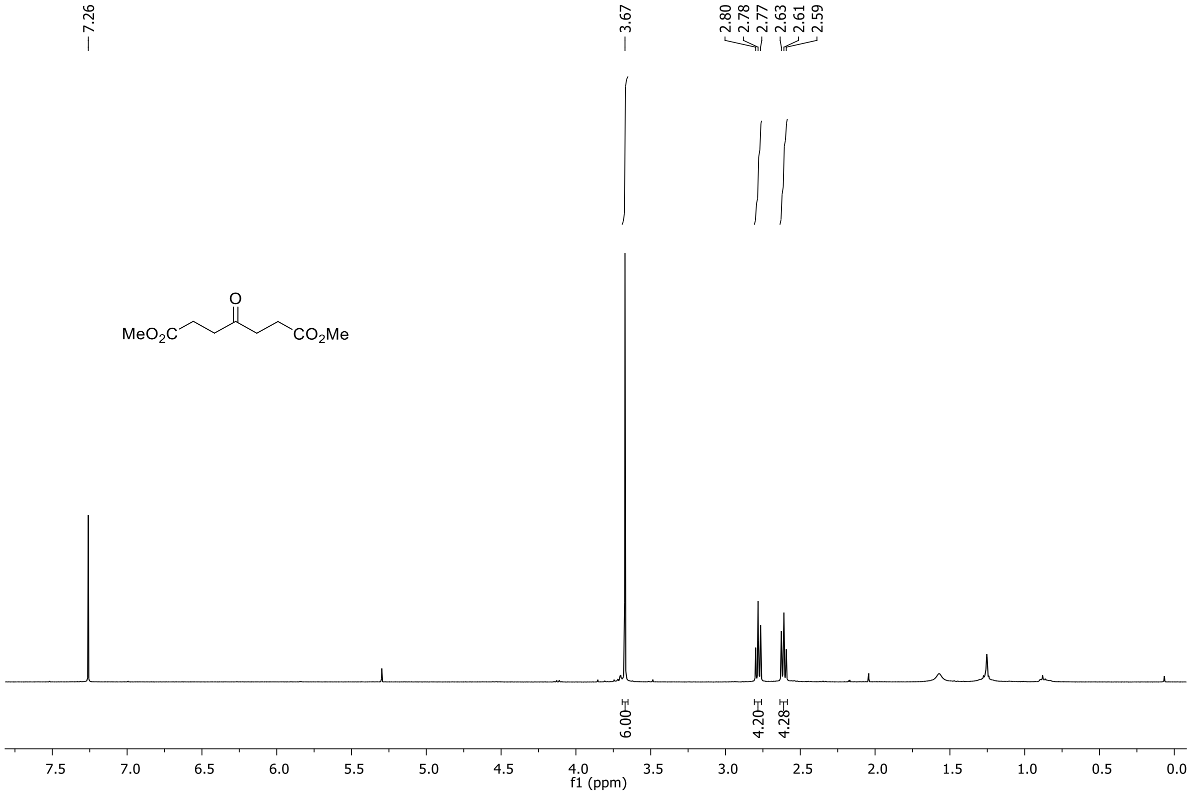

^1H NMR (400 MHz, $CDCl_3$) of **5p**

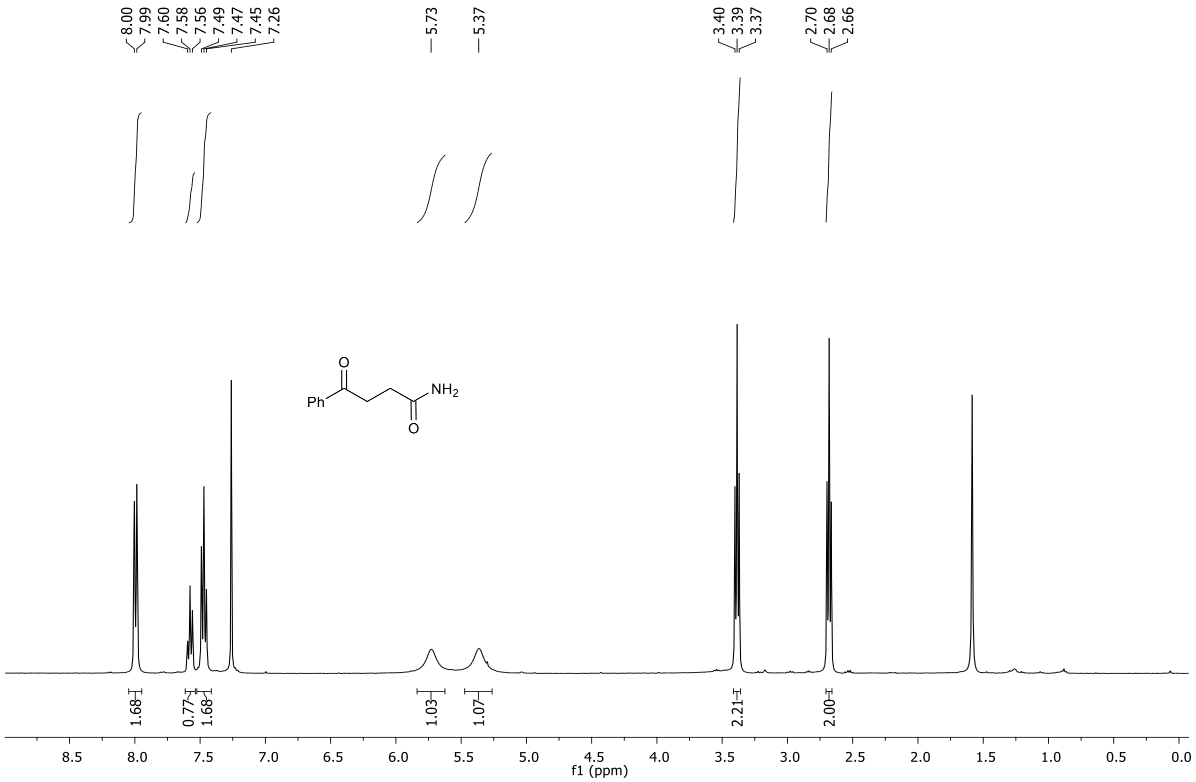

^1H NMR (400 MHz, $CDCl_3$) of **5q**

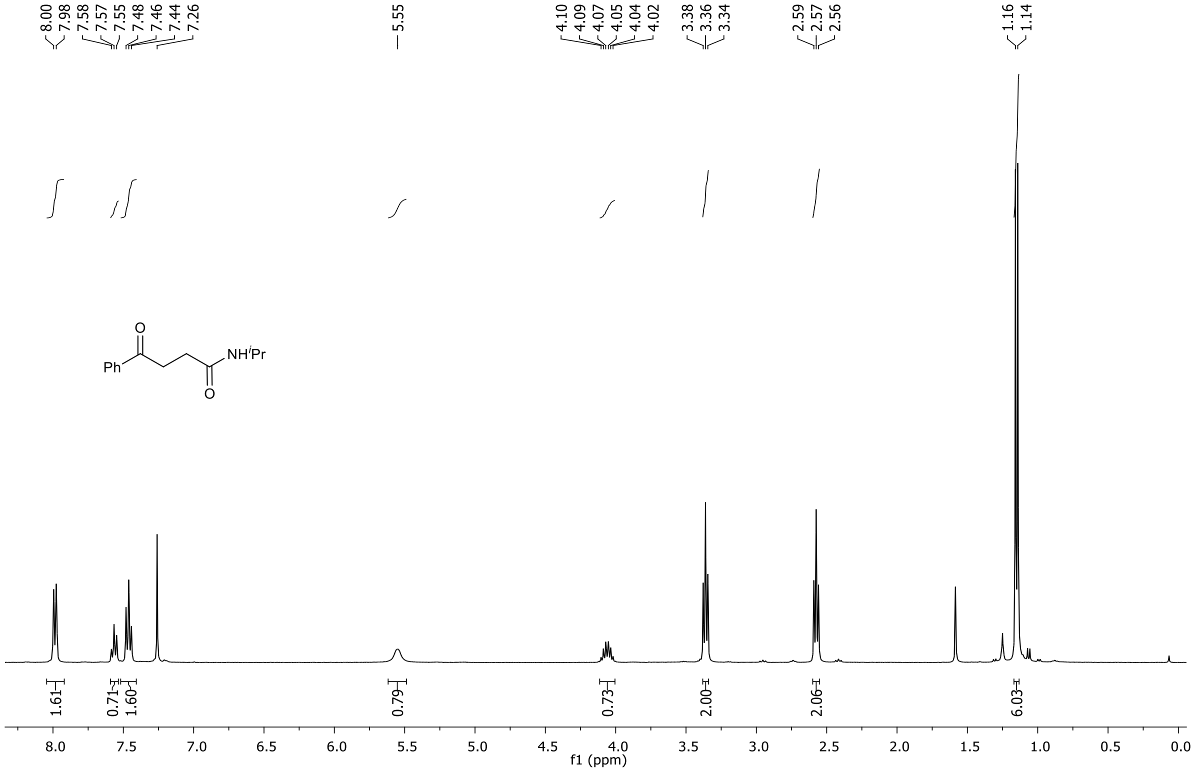

^{1}H NMR (400 MHz, $CDCl_3$) of **(-)-1o**

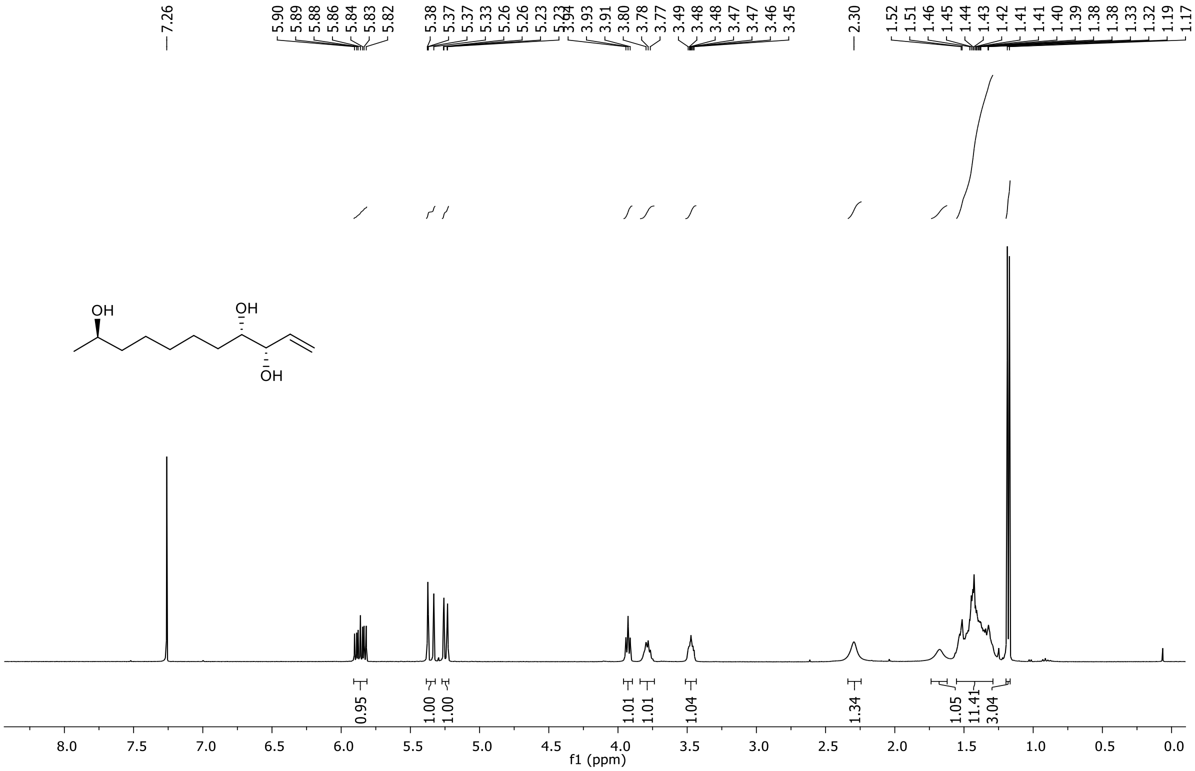

^{13}C NMR (100 MHz, $CDCl_3$) of **(-)-1o**

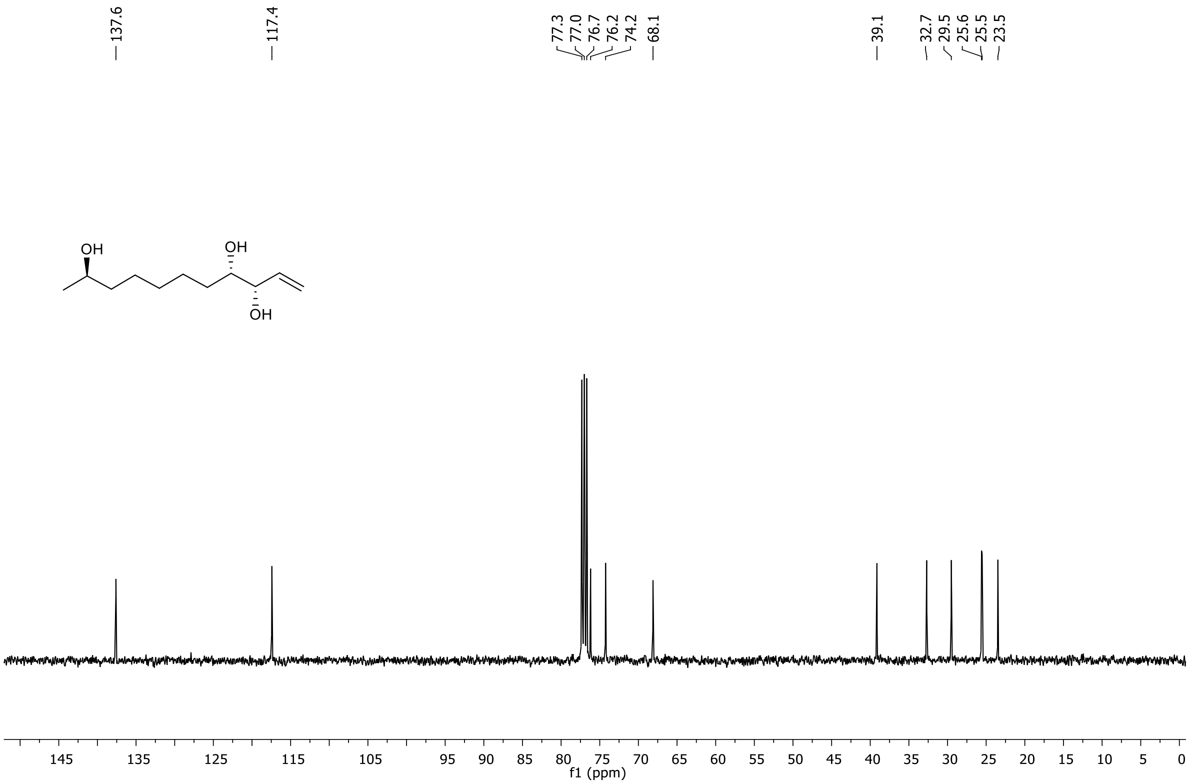

^{1}H NMR (400 MHz, $CDCl_3$) of **3w**

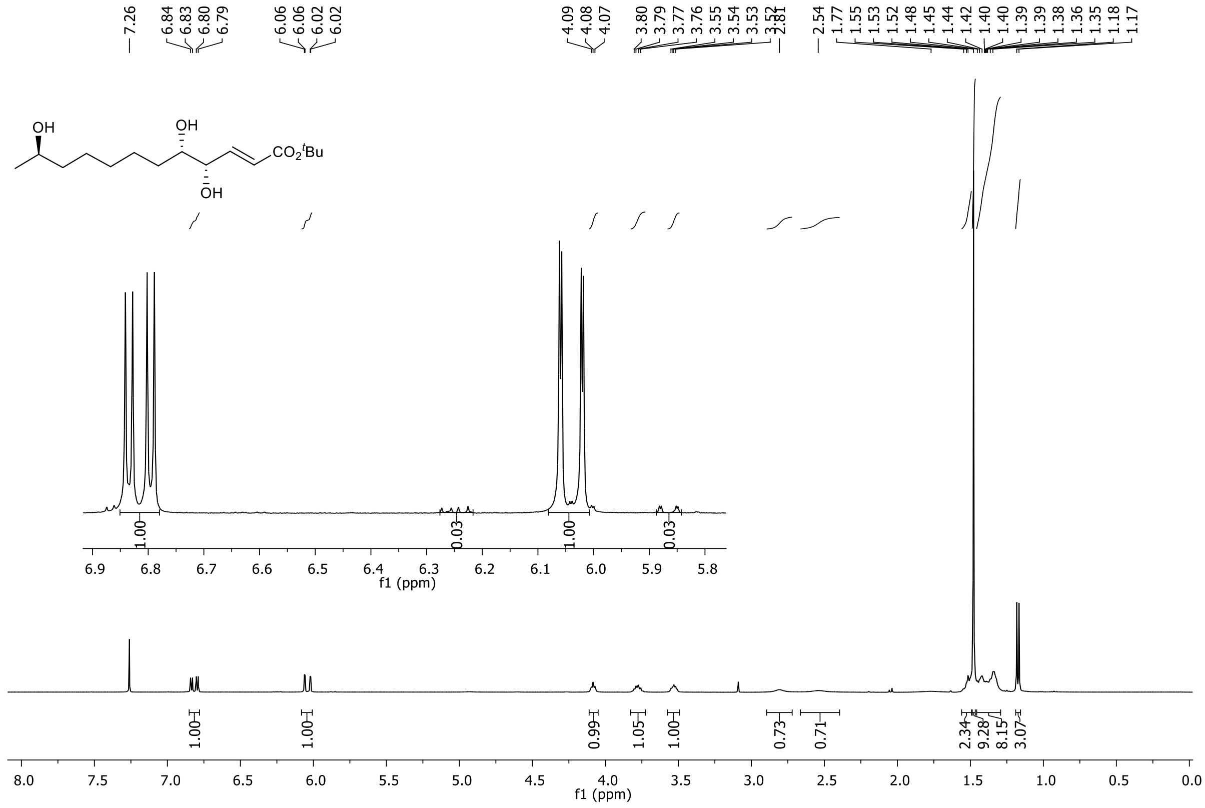

^{13}C NMR (100 MHz, $CDCl_3$) of **3w**

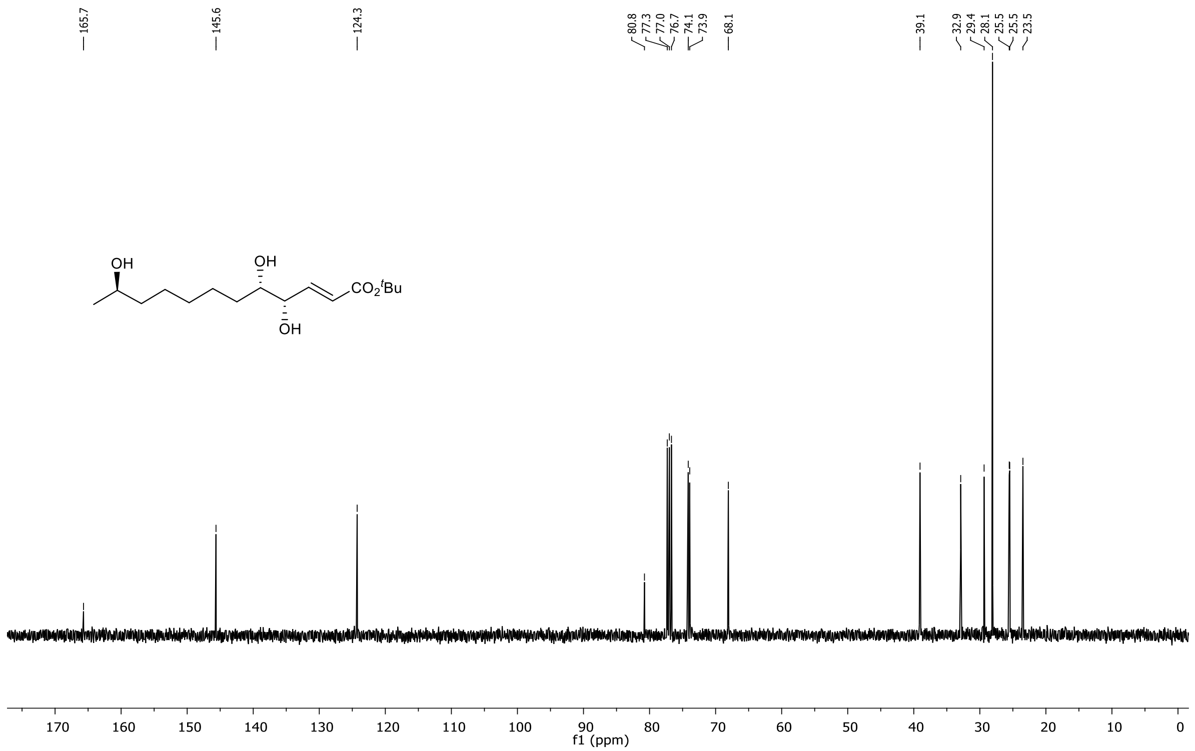

^{1}H NMR (400 MHz, $CDCl_3$) of **Iso-cladospolide B (4o)**

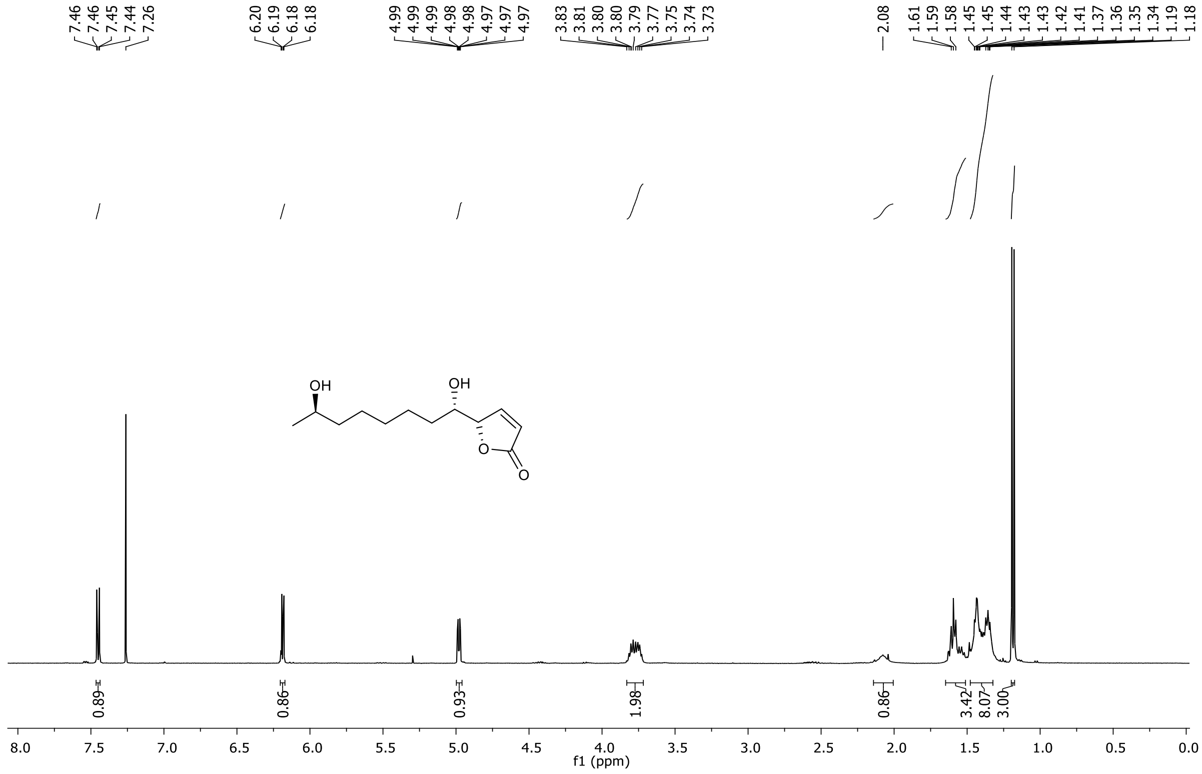

^{13}C NMR (100 MHz, $CDCl_3$) of **Iso-cladospolide B (4o)**

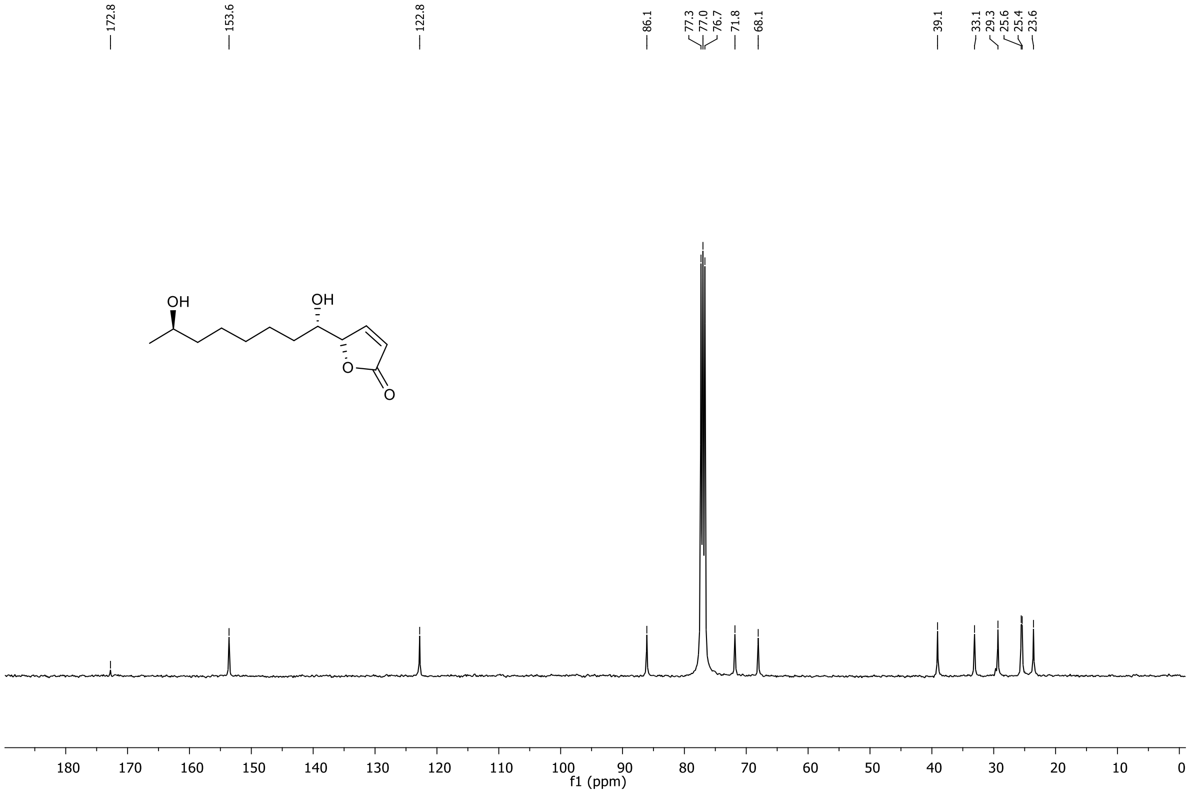

^{1}H NMR (400 MHz, $CDCl_3$) of **5r**

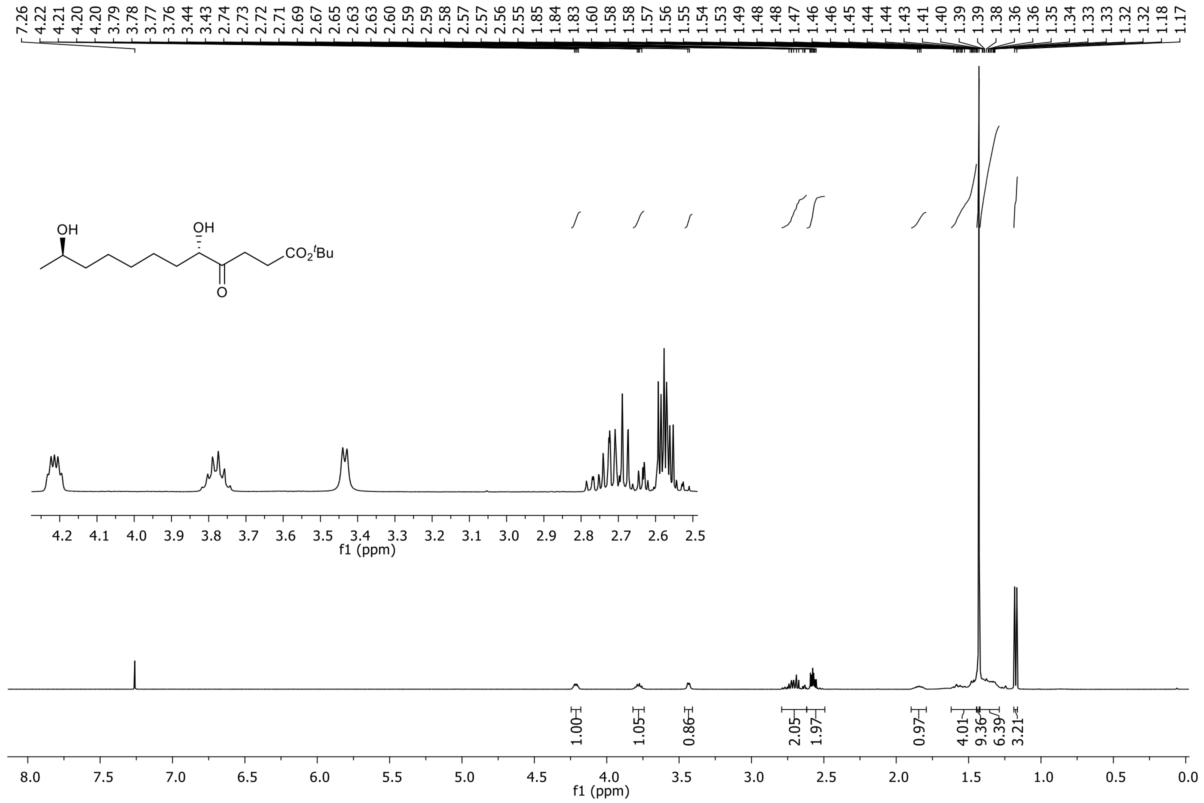

^{13}C NMR (100 MHz, $CDCl_3$) of **5r**

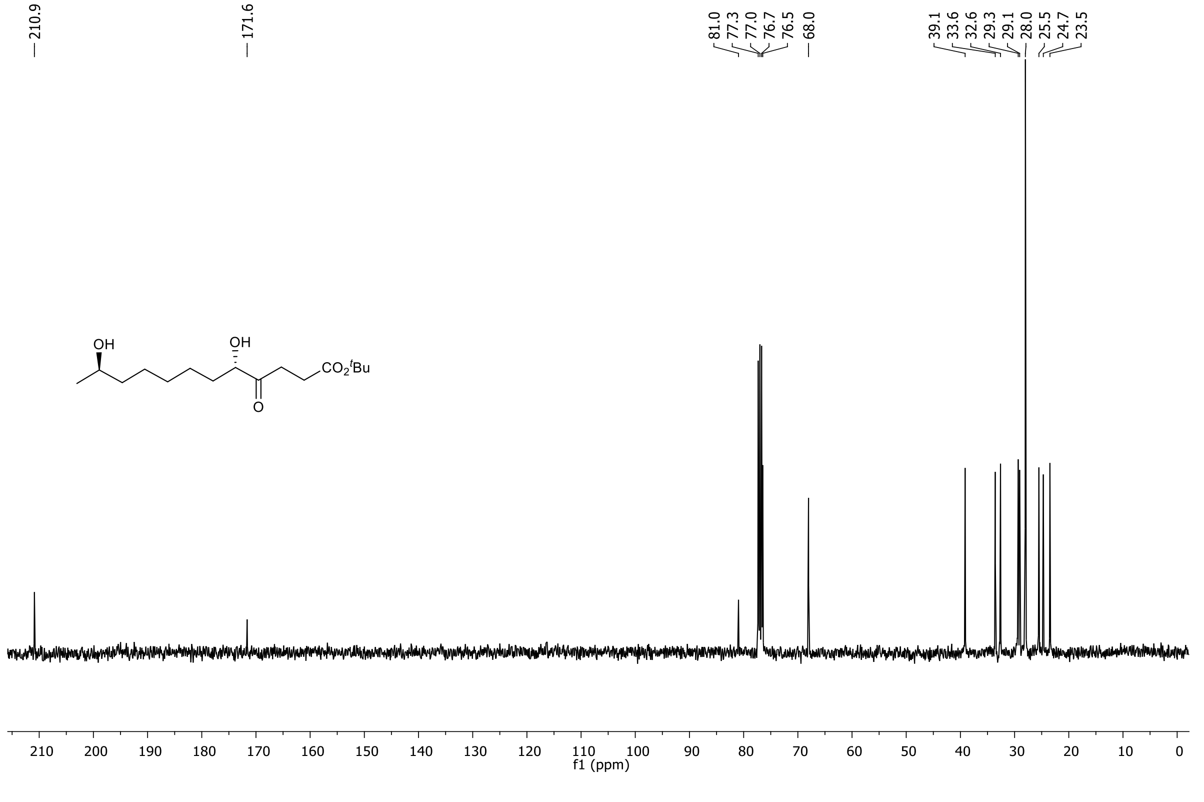

^{1}H NMR (400 MHz, $CDCl_3$) of **S5**

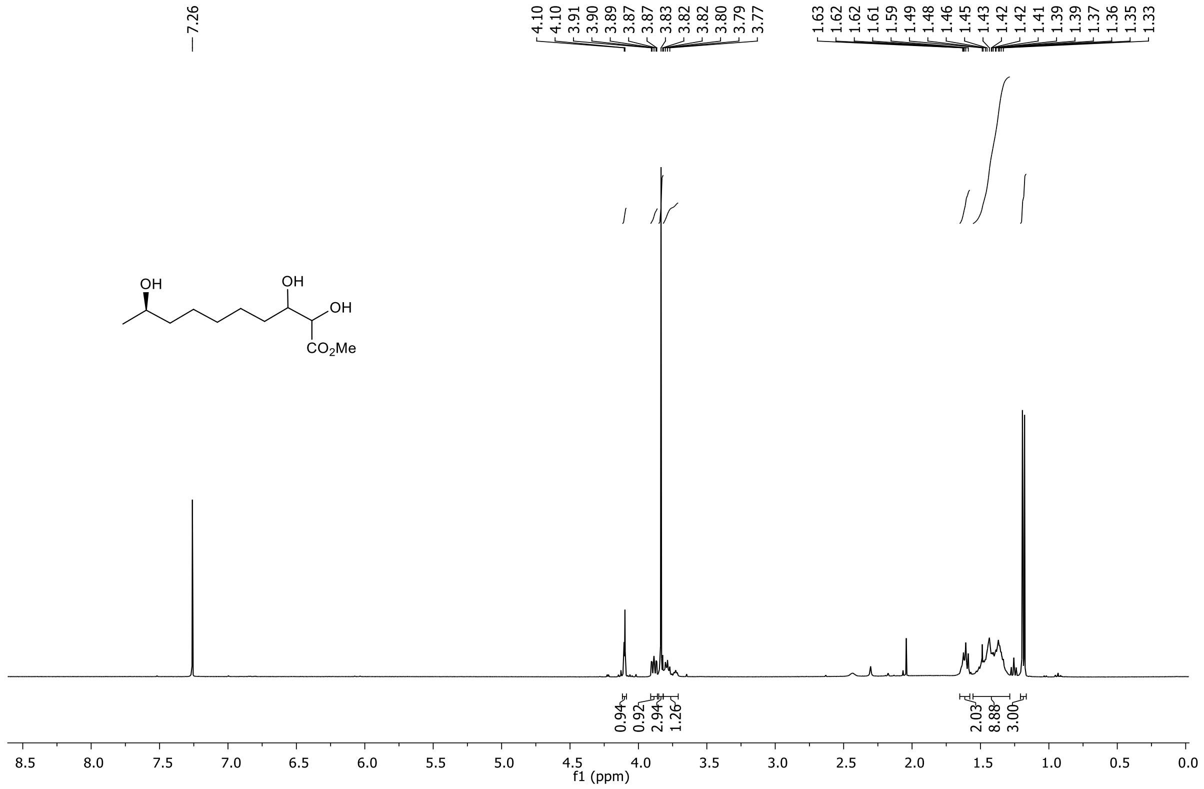

^{13}C NMR (100 MHz, $CDCl_3$) of **S5**

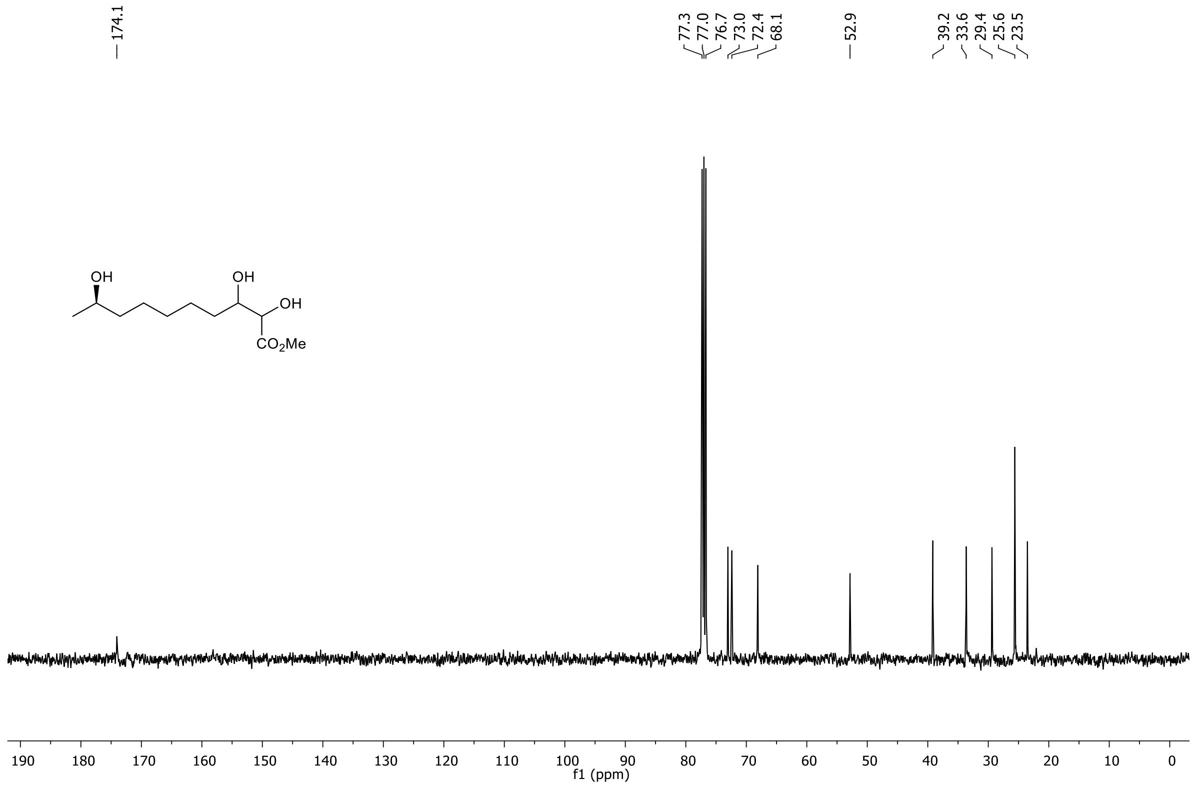

^{1}H NMR (400 MHz, $CDCl_3$) of **S6**

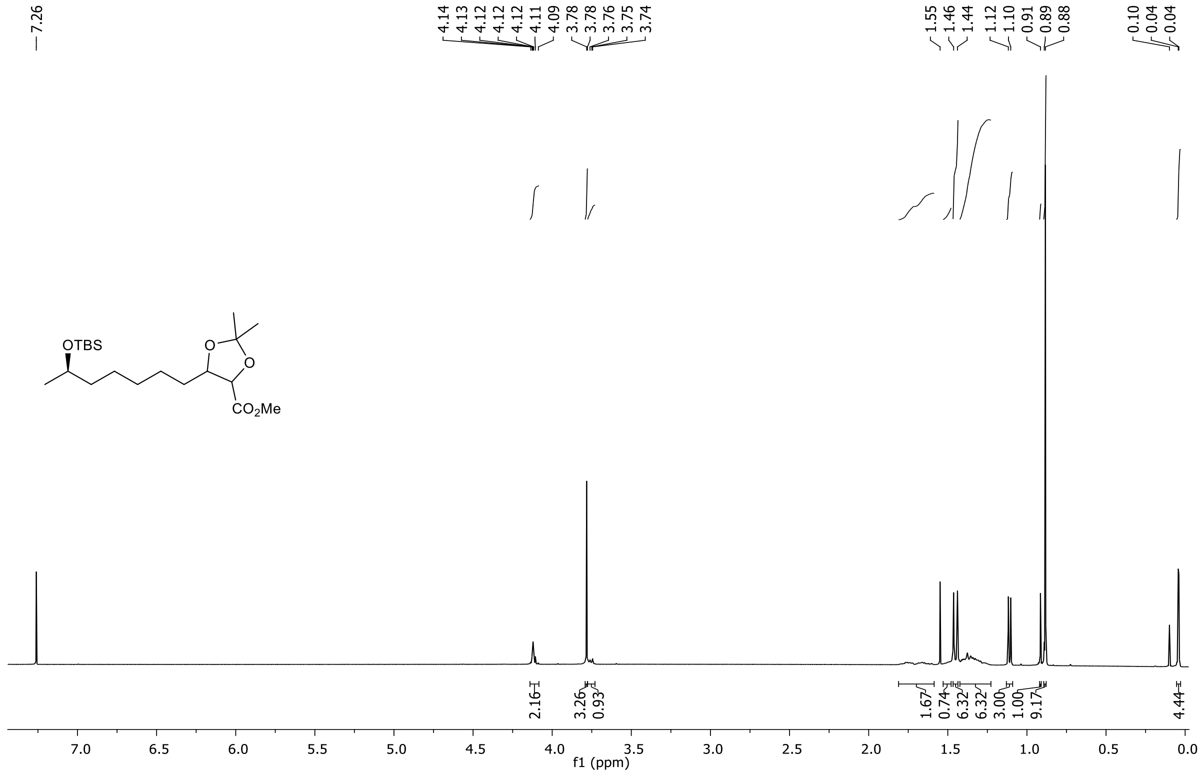

^{13}C NMR (100 MHz, $CDCl_3$) of **S6**

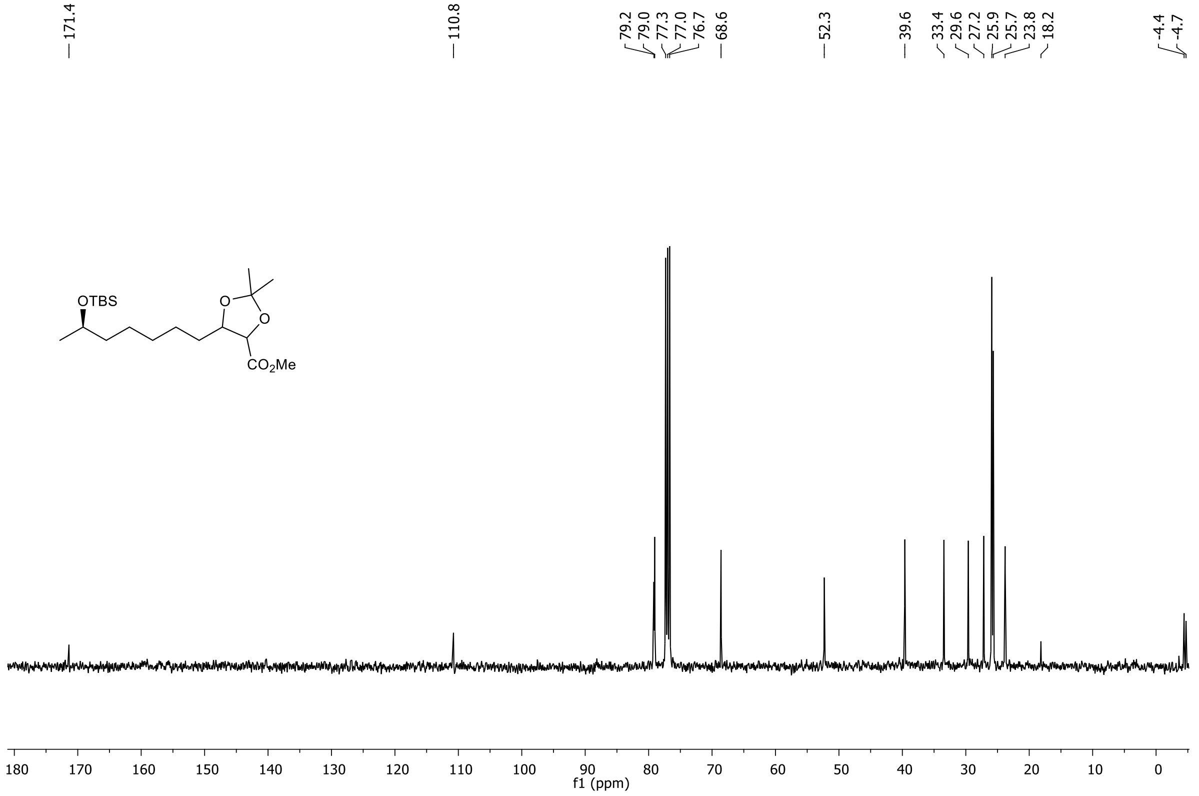

^{1}H NMR (400 MHz, $CDCl_3$) of **spectra of (±)-1o**

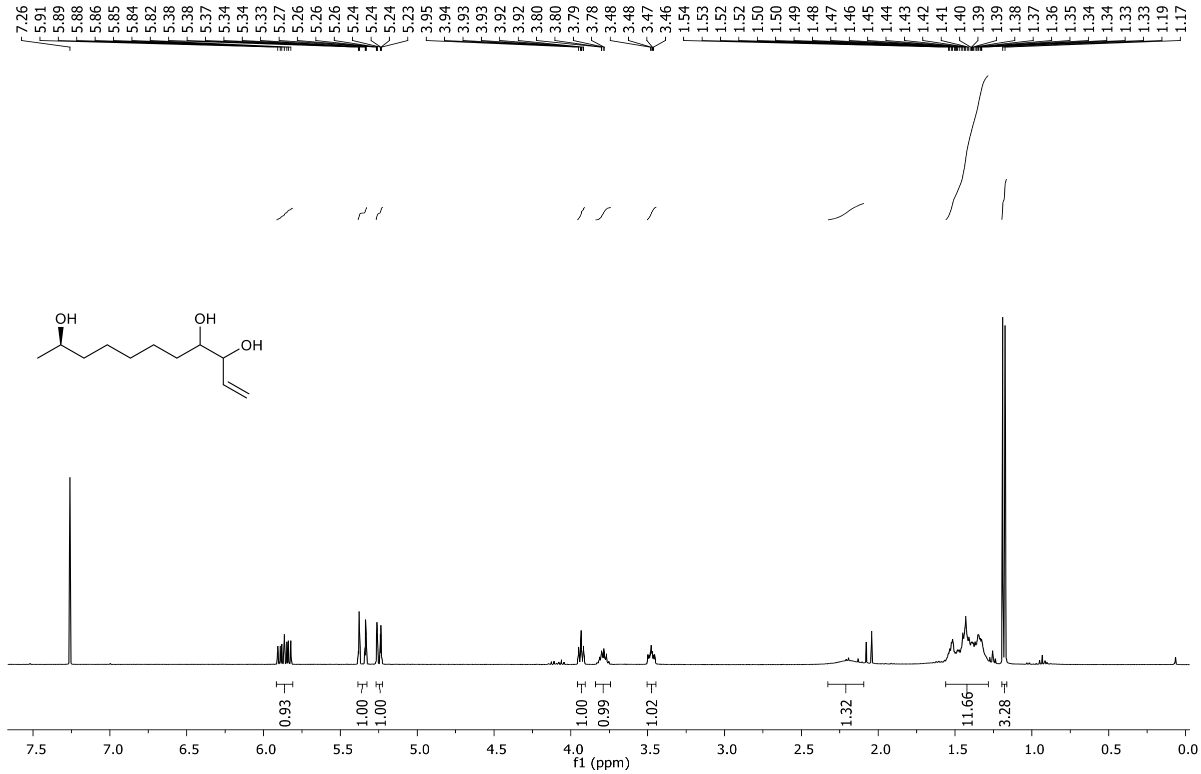

10. Chiral GC spectra

a. Chiral GC spectra of (±)-1b

Data File : RLS-653-SM-RAC.D Method : C:\msdchem\1\methods\Revchiral

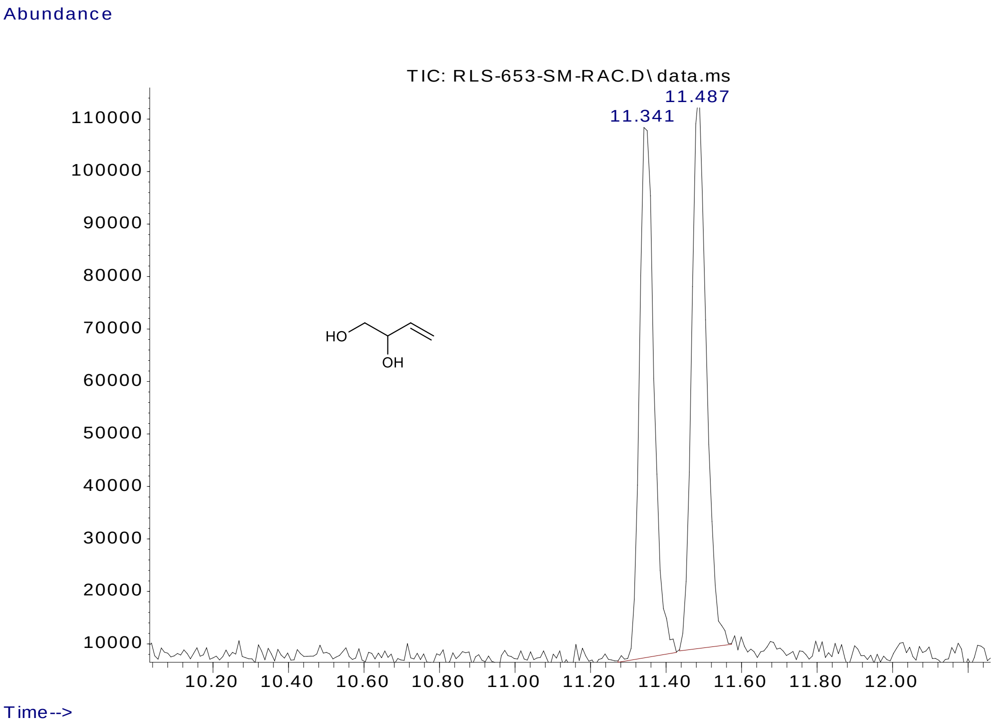

Peak #	R.T. min	first scan	max scan	last scan	PK TY	peak height	corr. area	corr. % max.	% of total
1	11.341	955	963	973	rM	101074	276339	95.48	48.844
2	11.487	974	980	990	rM	106827	289416	100.00	51.156

b. Chiral GC spectra of (*S*)-1b (Commercial Sample)

Data File : RLS-653-SM-CHIRAL.D Method : C:\msdchem\1\methods\Revchiral

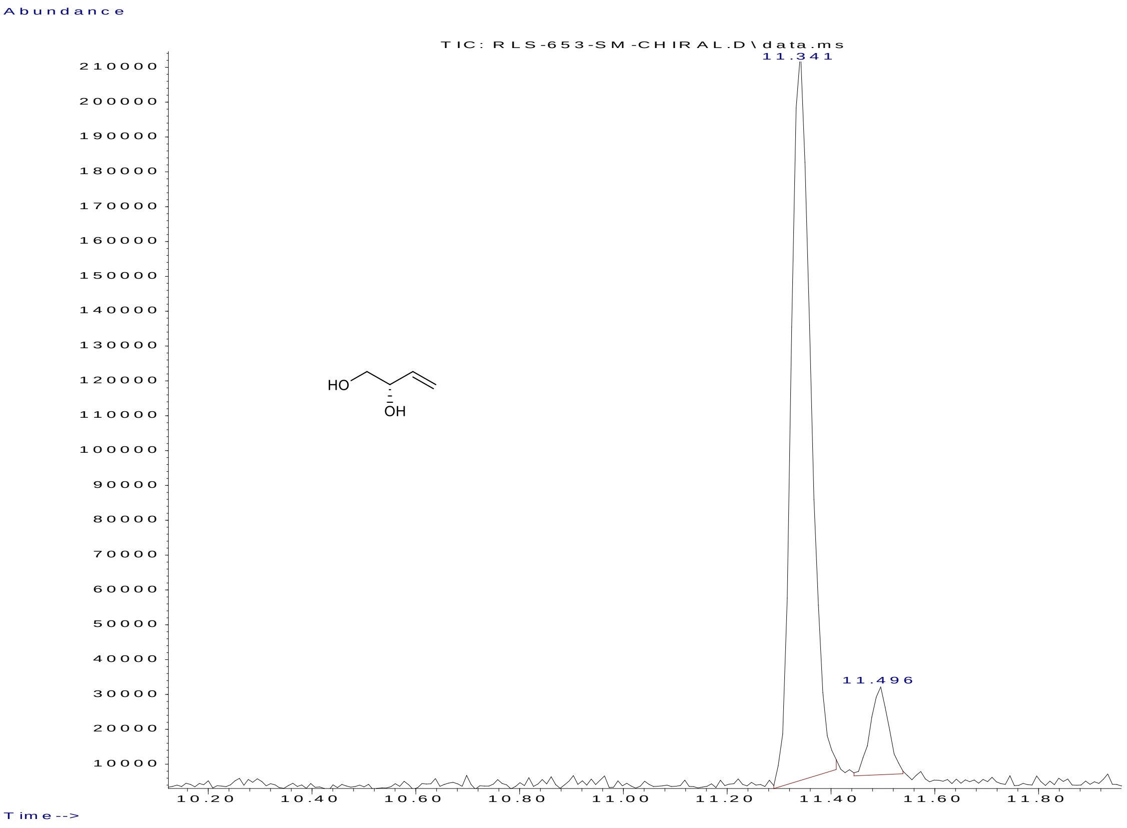

peak #	R.T. min	first scan	max scan	last scan	PK TY	peak height	corr. area	corr. % max.	% of total
1	11.341	957	963	972	rM	209355	561681	100.00%	89.413%
2	11.496	974	981	991	rM	25621	66505	11.84%	10.587%

c. Chiral GC spectra of (±)-4b

Data File : RLS-653-RAC.D Method : C:\msdchem\1\methods\TEMP.M

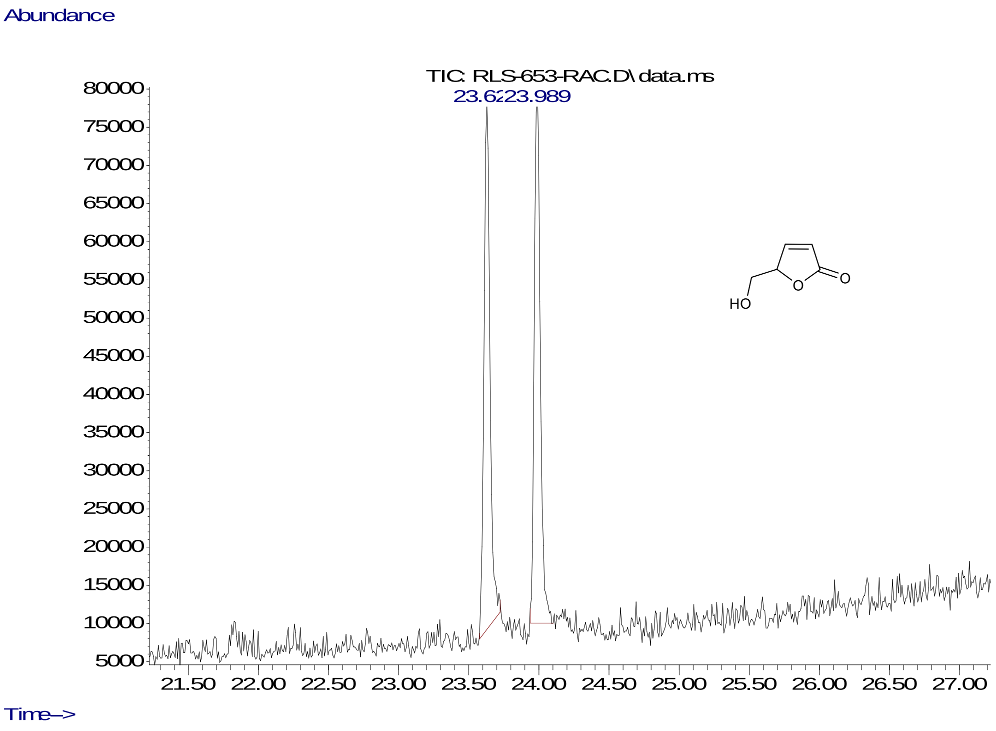

Peak #	R.T. min	first scan	max scan	last scan	PK TY	peak height	corr. area	corr. % max.	% of total
1	23.629	2389	2395	2406	rM	68851	207400	99.11	49.778
2	23.989	2431	2437	2450	rVB	70189	209252	100.00	50.222

d. Chiral GC spectra of (*S*)-4b

Data File : RLS-653-CHIRAL.D Method : C:\msdchem\1\methods\TEMP.M

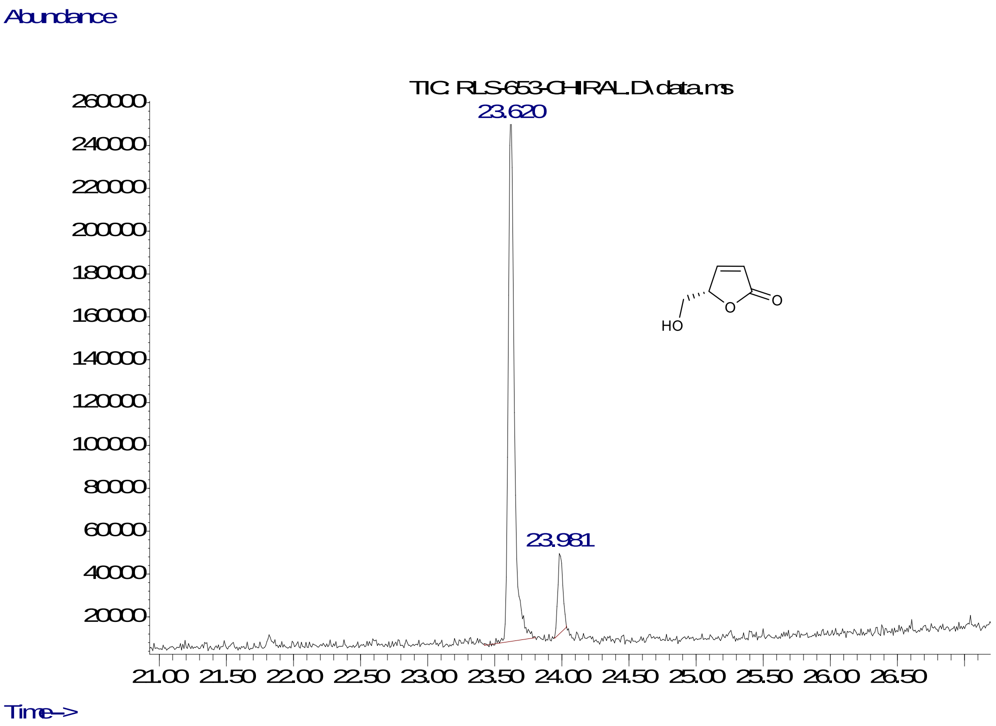

Peak #	R.T. min	first scan	max scan	last scan	PK TY	peak height	corr. area	corr. % max.	% of total
1	23.620	2371	2394	2415	rM	252082	776727	100.00	88.811
2	23.981	2432	2436	2442	rM	37554	97858	12.60	11.189

e. Chiral GC spectra of (±)-1o

Data File : RLS-814 RAC-1.D Method : C:\msdchem\1\methods\Revchiral

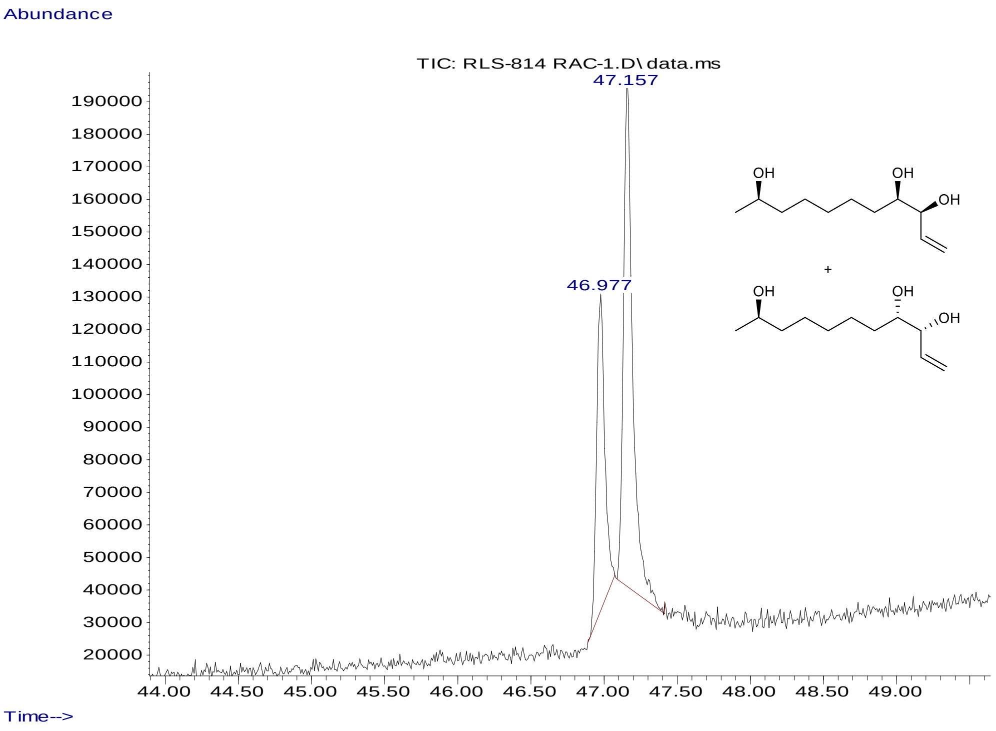

Peak #	R.T. min	first scan	max scan	last scan	PK TY	peak height	corr. area	corr. % max.	% of total
1	46.977	5106	5116	5127	rM	297214	381449	56.13	35.950
2	47.157	5129	5137	5167	rM	157940	679611	100.00	64.050

f. Chiral GC spectra of (-)-1o

Data File : RLS-782 CHIRAL.D   Method   : C:\msdchem\1\methods\Revchiral

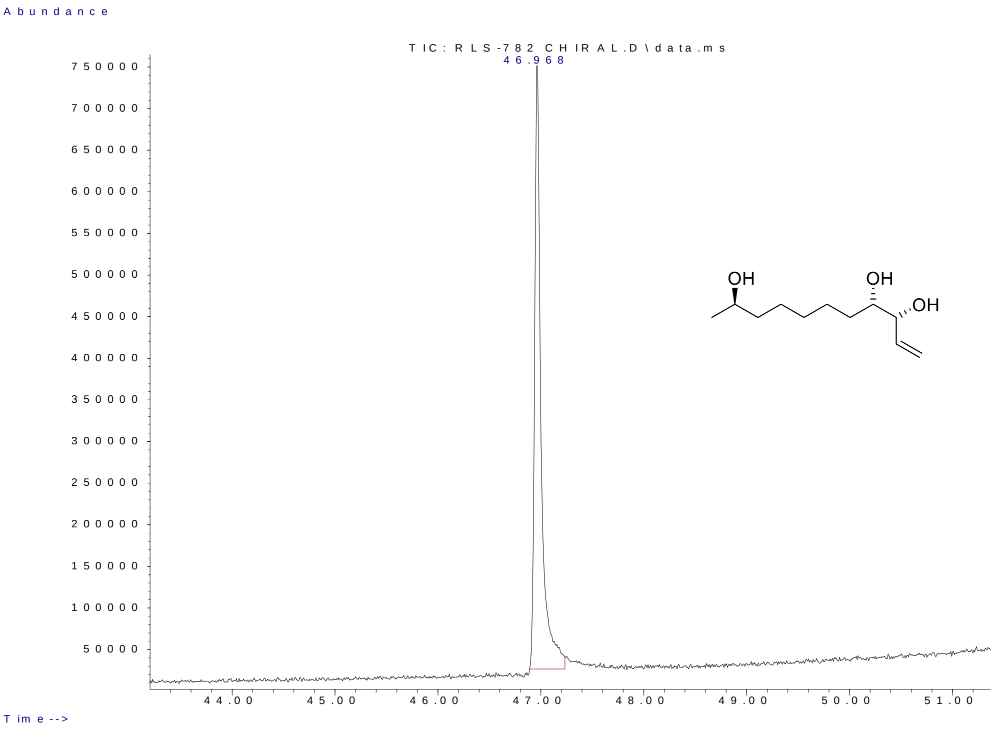

Peak #	R.T. min	first scan	max scan	last scan	PK TY	peak height	corr. area	corr. % max.	% of total
1	46.968	5106	5115	5146	rBV	738326	3212953	100.00	100.000

11. Computational Methods

All the molecules in the scheme below as well as phenanthrene were optimized in the ground state using the density functional B3LYP[17] with dispersion correction using Becke-Johnson damping D3(BJ).[18] The 6-311++G(d,p) basis set was used. The minimized ground state geometries of the molecules were used to calculate the vertical excitation energies at linear-response RI-CC2 level of theory[19] with frozen core orbitals and cc-pVTZ auxiliary basis set. In addition, the vertical excitation energies were calculated at the CAM-B3LYP/cc-pVTZ level in both gas and solution (solvent: Dichloromethane) phase. The Polarizable Continuum Model (PCM) using the integral equation formalism variant (IEFPCM)[20] and polarizable conductor model (CPCM)[21] were used for the solution phase calculations. The excitation energies, wavelengths and oscillator strengths of all the molecules in both gas and solution phase are listed in Table S5. The geometry optimization and CAM-B3LYP calculations were carried out using Gaussian 09 suite of program,[22] and the RI-CC2 calculations were performed with Turbomole 7 program package.[23]

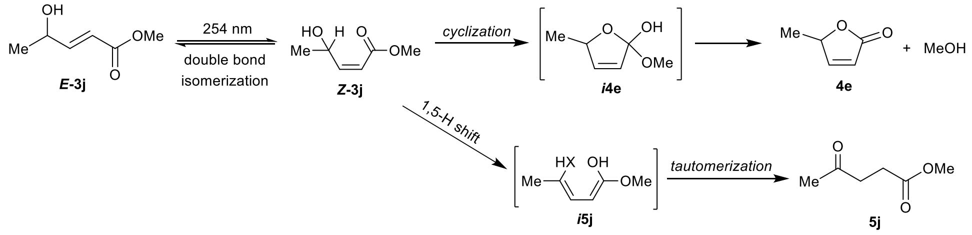

Table S 5: Excited state numbers, excitation energies (eV), wavelengths (nm) and oscillator strengths of all the molecules of photoisomerization reaction of *E*-3j in the gas and solution phase using cc-pVTZ basis set

Molecules	Ex. St. No.	CC2 (Gas Phase)			CAM-B3LYP (Gas Phase)			CAM-B3LYP/IEFPCM (CH_2Cl_2 Solvent)			CAM-B3LYP/CPCM (CH_2Cl_2 Solvent)		
		Ex. En.	Wavelength	Osc. Str.	Ex. En.	Wavelength	Osc. Str.	Ex. En.	Wavelength	Osc. Str.	Ex. En.	Wavelength	Osc. Str.
E-3j	S_1	5.17	240	0.0002	5.1679	240	0.0002	5.3105	233	0.0003	5.3182	233	0.0004
	S_2	6.55	189	0.3273	6.1932	200	0.427	6.0414	205	0.5331	6.0226	206	0.5447
	S_3	6.87	181	0.0819	6.6109	188	0.0836	6.5445	189	0.0748	6.5428	190	0.0703
	S_4	6.97	178	0.3037	7.0975	175	0.1164	7.1309	174	0.1124	7.1265	174	0.1156
	S_5	7.61	163	0.0207	7.5467	164	0.0142	7.6738	162	0.0038	7.6729	162	0.0035
	S_6	7.97	156	0.0038	7.6985	161	0.0027	7.8005	159	0.0133	7.816	159	0.0139
	S_7	8.34	149	0.0002	8.0681	154	0.0002	8.0665	154	0.0003	8.0667	154	0.0003
	S_8	8.50	146	0.0227	8.141	152	0.0148	8.3595	148	0.0215	8.3673	148	0.0237
	S_9	8.57	145	0.0458	8.5699	145	0.011	8.5595	145	0.0068	8.556	145	0.0068
	S_{10}	8.70	143	0.0746	8.5807	144	0.0042	8.6616	143	0.0005	8.6654	143	0.0006
Z-3j	S_1	5.00	248	0.003	4.9701	249	0.0021	5.0786	244	0.0011	5.0844	244	0.0011
	S_2	5.51	225	0.0147	5.4206	229	0.0099	5.5951	222	0.033	5.6034	221	0.0388
	S_3	6.46	192	0.4076	6.0623	205	0.3519	5.9902	207	0.4144	5.9712	208	0.4171
	S_4	6.88	180	0.0548	6.9605	178	0.0237	6.9633	178	0.0494	6.9591	178	0.0534
	S_5	7.56	164	0.0614	7.2642	171	0.083	7.3046	170	0.0584	7.3058	170	0.0571
	S_6	7.91	157	0.0019	7.7149	161	0.0006	7.7149	161	0.0012	7.7157	161	0.0013
	S_7	8.17	152	0.0106	8.1415	152	0.0118	8.1737	152	0.0072	8.1743	152	0.0077
	S_8	8.42	147	0.0081	8.2105	151	0.0077	8.3494	149	0.0175	8.3515	148	0.019
	S_9	8.59	144	0.0206	8.2883	150	0.0104	8.399	148	0.0118	8.4038	148	0.0129
	S_{10}	8.66	143	0.0258	8.3581	148	0.0226	8.4919	146	0.0342	8.4985	146	0.036
Dienol (*i*5j)	S_1	5.09	243	0.0584	4.9107	252	0.0417	4.992	248	0.5701	4.9791	249	0.5767
	S_2	5.23	237	0.5148	5.0668	245	0.4753	5.3959	230	0.0162	5.4196	229	0.0148
	S_3	6.40	194	0.0032	6.1861	200	0.002	6.408	193	0.0015	6.4248	193	0.0017
	S_4	6.68	186	0.0073	6.4829	191	0.0074	6.6551	186	0.012	6.6663	186	0.013
	S_5	6.98	178	0.0063	6.7423	184	0.0069	7.1465	173	0.0038	7.1668	173	0.0037
	S_6	7.26	171	0.0191	7.2346	171	0.0094	7.2356	171	0.0095	7.2342	171	0.0112
	S_7	7.58	163	0.0035	7.3331	169	0.0057	7.5409	164	0.0271	7.5512	164	0.0518
	S_8	7.70	161	0.0033	7.5291	165	0.0021	7.6578	162	0.0166	7.6355	162	0.3305
	S_9	7.74	160	0.0049	7.6227	163	0.0212	7.661	162	0.3145	7.6692	162	0.0009
	S_{10}	7.92	156	0.2118	7.6937	161	0.173	8.0559	154	0.0077	8.0781	153	0.0074
*i*4e	S_1	6.69	185	0.0759	6.8157	182	0.1351	6.8981	180	0.2356	6.8956	180	0.2526
	S_2	7.11	174	0.0171	7.1842	173	0.0036	7.3187	169	0.0041	7.3255	169	0.0044

	S_3	7.88	157	0.0016	7.5617	164	0.2229	7.5635	164	0.2115	7.5548	164	0.2083
	S_4	7.98	155	0.2880	7.9093	157	0.0085	8.0904	153	0.0091	8.1003	153	0.0098
	S_5	8.08	154	0.0155	7.9961	155	0.0073	8.1638	152	0.0071	8.1711	152	0.0074
	S_6	8.26	150	0.0121	8.1678	152	0.0138	8.3047	149	0.0042	8.3093	149	0.0046
	S_7	8.28	150	0.0118	8.2316	151	0.0034	8.3743	148	0.0186	8.3873	148	0.02
	S_8	8.35	148	0.0109	8.3699	148	0.0035	8.5573	145	0.0028	8.5688	145	0.003
	S_9	8.61	144	0.0576	8.6531	143	0.0325	8.8389	140	0.0382	8.8508	140	0.0412
	S_{10}	8.71	142	0.0267	8.7598	142	0.0073	8.9576	138	0.0034	8.9642	138	0.0029
5j	S_1	4.67	265	0.0001	4.5637	272	0.0001	4.6589	266	0.0001	4.6653	266	0.0001
	S_2	6.14	202	0.0006	6.0008	207	0.0006	6.0792	204	0.0008	6.0847	204	0.0009
	S_3	7.13	174	0.0321	7.4757	166	0.0299	7.7488	160	0.0509	7.7631	160	0.0537
	S_4	8.13	153	0.0493	7.9197	157	0.0102	7.9467	156	0.0059	7.9502	156	0.0051
	S_5	8.18	152	0.0163	8.0385	154	0.0171	8.1367	152	0.0419	8.1419	152	0.053
	S_6	8.25	150	0.1480	8.2541	150	0.0600	8.2037	151	0.0922	8.1967	151	0.0878
	S_7	8.35	149	0.0038	8.4373	147	0.0646	8.6229	144	0.0374	8.6266	144	0.0403
	S_8	8.45	147	0.0135	8.5184	146	0.0335	8.6856	143	0.0993	8.6923	143	0.1065
	S_9	8.91	139	0.0098	8.8358	140	0.0019	8.7851	141	0.0009	8.7837	141	0.001
	S_{10}	9.03	137	0.0199	8.8575	140	0.002	8.8479	140	0.0009	8.8485	140	0.0009
4e	S_1	5.04	246	0.0001	5.0733	244	0	5.3839	230	0	5.3967	230	0
	S_2	6.29	197	0.1359	6.476	191	0.2657	6.4459	192	0.4048	6.4253	193	0.4189
	S_3	7.17	173	0.2130	6.864	181	0.0612	6.8744	180	0.0133	6.8742	180	0.0149
	S_4	7.64	162	0.0023	7.59	163	0.0028	7.5631	164	0.0054	7.5622	164	0.0058
	S_5	8.05	154	0.0017	8.0349	154	0.0006	8.1808	152	0.0007	8.1871	151	0.0007
	S_6	8.44	147	0.0455	8.2484	150	0.0018	8.4199	147	0.0031	8.4295	147	0.0033
	S_7	8.48	146	0.0008	8.4364	147	0.0052	8.739	142	0.0091	8.7467	142	0.0102
	S_8	8.69	143	0.0097	8.8615	140	0.0355	9.1835	135	0.0648	9.1784	135	0.0813
	S_9	9.08	136	0.0246	9.0777	137	0.0193	9.2149	135	0.0464	9.2087	135	0.0392
	S_{10}	9.13	136	0.0207	9.2185	135	0.0015	9.3244	133	0.0045	9.3279	133	0.0036
Phenanthrene	S_1	4.08	304	0.0008	4.2461	292	0.0011	4.2489	292	0.0023	4.248	292	0.0027
	S_2	4.73	262	0.0759	4.5145	275	0.0627	4.4912	276	0.1272	4.4854	276	0.1399
	S_3	5.13	242	0.0817	5.2319	237	0.1727	5.1484	241	1.2318	5.1272	242	1.2659
	S_4	5.17	240	0.1662	5.2851	235	0.7181	5.1751	240	0.262	5.1573	240	0.2852
	S_5	5.40	229	1.0475	5.4302	228	0.3418	5.4032	229	0.0775	5.4008	230	0.0651
	S_6	5.59	222	0.0249	5.8186	213	0.0812	5.7835	214	0.1367	5.7739	215	0.1501
	S_7	6.20	200	0.1883	6.0722	204	0.0838	6.0381	205	0.0895	6.0299	206	0.0879
	S_8	6.33	196	0.0494	6.4166	193	0.4282	6.3308	196	0.4417	6.311	196	0.4435
	S_9	6.43	193	0.4264	6.6234	187	0.0231	6.6273	187	0.0101	6.6276	187	0.0086
	S_{10}	6.53	190	0.0009	6.813	182	0	6.8227	182	0	6.8233	182	0
	S_{11}	6.85	181	0	6.9018	180	0.0093	6.8916	180	0.0116	6.8871	180	0.0118
	S_{12}	7.02	177	0	6.9528	178	0.0008	6.9998	177	0.0002	6.9941	177	0.0002

S_{13}	7.03	176	0.0384	7.0524	176	0.0003	7.0817	175	0.4479	7.0478	176	0.4533
S_{14}	7.10	175	0.0020	7.155	173	0.002	7.117	174	0	7.1112	174	0
S_{15}	7.12	174	0	7.169	173	0	7.1819	173	0.0012	7.2043	172	0.0013
S_{16}	7.17	173	0.1798	7.2081	172	0.003	7.2372	171	0.1914	7.2297	171	0.1905
S_{17}	7.19	172	0	7.2084	172	0.3806	7.2434	171	0	7.2393	171	0
S_{18}	7.20	172	0.0009	7.2714	171	0.2032	7.3809	168	0.0045	7.3814	168	0.0036
S_{19}	7.23	172	0.0884	7.2826	170	0	7.3926	168	0	7.4097	167	0.0014
S_{20}	7.27	170	0.3386	7.4287	167	0	7.4349	167	0	7.4572	166	0
S_{21}	7.30	170	0	7.4323	167	0.0018	7.5067	165	0.0052	7.5064	165	0.0052
S_{22}	7.32	169	0.0009	7.4993	165	0.0057	7.6662	162	0	7.6904	161	0
S_{23}	7.46	166	0	7.5573	164	0.0131	7.7512	160	0.0175	7.746	160	0.0191
S_{24}	7.60	163	0	7.5912	163	0	7.785	159	0.0171	7.7976	159	0
S_{25}	7.61	163	0.0095	7.7619	160	0.0118	7.8022	159	0	7.8064	159	0.0191
S_{26}	7.65	162	0.0138	7.8679	158	0	7.8379	158	0	7.8597	158	0
S_{27}	7.91	157	0	7.9273	156	0	7.9285	156	0.2546	7.9053	157	0.2908
S_{28}	8.02	155	0	7.9986	155	0.1305	7.973	156	0.0002	7.9671	156	0.0003
S_{29}	8.06	154	0.0171	8.0257	154	0.0009	8.0013	155	0.0031	7.9934	155	0.0033
S_{30}	8.07	154	0.0012	8.0527	154	0.0075	8.0905	153	0.0294	8.0844	153	0.0291
S_{31}	8.12	153	0.3240	8.0755	154	0.0002	8.1739	152	0	8.1939	151	0
S_{32}	8.12	153	0.0019	8.1072	153	0.0293	8.2047	151	0	8.2026	151	0
S_{33}	8.17	152	0.0038	8.2582	150	0	8.2534	150	0.3046	8.2425	150	0.2939
S_{34}	8.29	150	0	8.2966	149	0.3171	8.299	149	0.008	8.3217	149	0.0091
S_{35}	8.30	149	0.1987	8.2978	149	0.0001	8.4688	146	0.0068	8.467	146	0.0053
S_{36}	8.36	148	0.0039	8.3495	148	0.0031	8.5368	145	0	8.5556	145	0.0071
S_{37}	8.38	148	0.0011	8.4683	146	0.0198	8.5627	145	0.0071	8.5612	145	0
S_{38}	8.39	148	0	8.5902	144	0.0161	8.5995	144	0.0037	8.6249	144	0.0045
S_{39}	8.52	146	0	8.6354	144	0	8.7076	142	0.0105	8.688	143	0.0112
S_{40}	8.58	144	0	8.6486	143	0	8.7197	142	0	8.7146	142	0

12. References

(1) S. H. Kang and H-W. Choi, *Chem. Commun.* 1996, 1521–1522.

(2) (a) T. A. McTeague and T. F. Jamison, *Angew. Chem. Int. Ed.* 2016, **55**, 15072–15075; (b) H. Lin, Y. Liu and Z-L. Wu, *Chem. Commun*. 2011, **47**, 2610–2612; (c) J. Rintjema, R. Epping, G. Fiorani, E. Martín, E. C. Escudero-Adán and A. W. Kleij, *Angew. Chem. Int. Ed.* 2016, **55**, 3972–3976; (d) N. Miralles, R. Kálamán, K. J. Szabó and E. Fernndez, *Angew. Chem. Int. Ed*. 2016, **55**, 4303–4307; (e) Q. Xiao, Q. He, J. Li and J. Wang, *Org. Lett*. 2015, **17**, 6090−6093.

(3) C. T. Meta and K. Koide, *Org. Lett*. 2004, **6**, 1785–1787.

(4) C. M. Yoing, D. G. Stark, T. H. West, J. E. Taylor and A. D. Smith, *Angew. Chem. Int. Ed.* 2016, **55**, 14394–14399.

(5) D. Cuperly, J. Petrignet, C. Crévisy and R. Grée, *Chem. Eur. J.* 2006, **12**, 3261–3274.

(6) S. M. Gromek, J. A. deMayo, A. T. Maxwell, A. M. West, C. M. Pavlik, Z. Zhao, J. Li, A. J. Wiemer, A. Zweifach and M. J. Balunas, *Bioorg. Med. Chem.* 2016, **24**, 5183–5196.

(7) I. Kholod, O. Vallat, A-M. Buciumas, A. Neels and R. Neier, *Eur. J. Org. Chem.* 2014, 7865–7877.

(8) J. Song and R. I. Hollingsworth, *Tetrahedron: Asymmetry* 2001, **12**, 387–391.

(9) A. G. M. Barrett, J. Head, M. L. Smith, N. S. Stock, A. J. P. White and D. J. Williams, *J. Org. Chem.* 1999, **64**, 6005–6018.

(10) (a) B. Schmidt and S. Hauke, *Org. Biomol. Chem.* 2013, **11**, 4194–4206; (b) J. H. Lee and S. Lee, *Chem. Sci.* 2013, **4**, 2922–2927; (c) G-Q. Tian, J. Yang and K. Rosa-Perez, *Org. Lett.* 2010, **12**, 5072–5074; (d) C. Gaul, K. Schärer and D. Seebach, *J. Org. Chem.* 2001, **66**, 3059–3073.

(11) (a) D. M. Browne, O. Niyomura and T. Wirth, *Org. Lett*. 2007, **9**, 3169–3171; (b) J-E. Kang, E-S. Lee, S-I. Park and S. Shin, *Tetrahedron Lett.* 2005, **46**, 7431–7433; (c) J. Ma, S. Wang and H. G. R. Tian, *Synth. Commun.* 2006, **36**, 1229–1233; (d) Y. Kawamata, T. Hashimoto and K. Maruoka, *J. Am. Chem. Soc.* 2016, **138**, 5206–5209; (e) S. Phae-nok, C. Kuhakarn, M. Pohmakotr, V. Reutrakul and D. Soorukram, *Org. Biomol. Chem.* 2015, **13**, 11087–11095; (f) M. M. Islam, S. Bhunia, R. A. Molla, A. Bhaumik and S. M. Islam, *ChemistrySelect* 2016, **1**, 6079–6085; (g) G-Z. Wang, R. Shang, W-M. Cheng and Y. Fu, *Org. Lett.* 2015, **17**, 4830−4833; (h) L. Lai, A-N. Li, J. Zhou, Y. Guo, L. Lin, W. Chena and R. Wang, *Org. Biomol. Chem.* 2017, **15**, 2185–2190; (i) H. Liu, X. He, D. Phillips, X. Zhu, K. Yang, T. Lau, B. Wu, Y. Xie, T. N. Nguyen and X. Wang, WO 2008/076754 A2, 26 Jun.

2008; (j) A. Bashir-Hashemi' and J. R. Hardee, *J. Org. Chem.* 1994, **59**, 2132–2134; (k) T. Mitamura, A. Nomoto, M. Sonoda and A. Ogawa, *Tetrahedron*, 2008, **64**, 9983–9988; (l) K. Yamada, S. Yoshida, H. Fujita, M. Kitamura and M. Kunishima, *Eur. J. Org. Chem.* 2015, 7997–8002.

(12) (a) A. L. Flourat, A. A. M. Peru, A. R. S. Teixeira, F. Brunissen and F. Allais, *Green Chem.* 2015, **17**, 404–412; (b) J. Sedelmeier, S. V. Ley, I. R. Baxendale and M. Baumann, *Org. Lett.* 2010, **12**, 3618–3621; (c) J. A. J. M. Vekemans, J. Boerekamp, E. F. Godefroi and G. J. F. Chittenden, *Recl. Trau. Chim. Pays-Bas* 1985, **104**, 266–272.

(13) (a) M. Zhao, W. Li, X. Li, K. Ren, X. Tao, X. Xie, T. Ayad, V. Ratovelomanana-Vidal and Z. Zhang, *J. Org. Chem.* 2014, **79**, 6164−6171; (b) T. J. Commons, WO 99/25682, 27 May 1999.

(14) A. G. Ross, X. Li and S. Danishefsky, J. *J. Am. Chem. Soc.* 2012, **134**, 16080−16084.

(15) M. González, Z. Gándara, A. Martínez, G. Gómez and Y. Fall, *Tetrahedron Lett.* 2013, **54**, 3647–3650.

(16) (a) P. Saidhareddy and A. K. Shaw, *RSC Adv.* 2015, **5**, 29114–29120; (b) J-X. Gu, Z-Y. Li and G-Q. Lin, *Tetrahedron Asymmetry.* 1992, **3**, 1523.1524.

(17) (a) S. H. Vosko, L. Wilk and M. Nusair, *Can. J. Phys.* 1980, **58**, 1200–1211; (b) C. Lee, W. Yang, and R. G. Parr, *Phys. Rev. B* 1988, **37**, 785–789; (c) A. D. Becke, *J. Chem. Phys.* 1993, **98**, 5648–5652; (d) P. J. Stephens, F. J. Devlin, C. F. Chabalowski and M. J. Frisch, *J. Phys. Chem.* 1994, **98**, 11623–11627.

(18) S. Grimme, S. Ehrlich and L. Goerigk, *J. Comp. Chem*. 2011, **32**, 1456–1465.

(19) (a) O. Christiansen, H. Koch and P. Jørgensen. *Chem. Phys. Lett.* 1995, **243**, 409–418; (b) C. Hättig and F. Weigend, *J. Chem. Phys.* 2000, **113**, 5154–5161; (c) C. Hättig and A. Köhn, *J. Chem. Phys.* 2002, **117**, 6939–6951.

(20) J. Tomasi, B. Mennucci and R. Cammi, *Chem. Rev.* 2005, **105**, 2999–3093.

(21) (a) V. Barone and M. Cossi, *J. Phys. Chem. A* 1998, **102**, 1995–2001. (b) M. Cossi, N. Rega, G. Scalmani and V. Barone, *J. Comp. Chem.* 2003, **24**, 669–681.

(22) M. J. Frisch, G. W. Trucks, H. B. Schlegel, G. E. Scuseria, M. A. Robb, J. R. Cheeseman, G. Scalmani, V. Barone, B. Mennucci, G. A. Petersson, H. Nakatsuji, M. Caricato, X. Li, H. P. Hratchian, A. F. Izmaylov, J. Bloino, G. Zheng, J. L. Sonnenberg, M. Hada, M. Ehara, K. Toyota, R. Fukuda, J. Hasegawa, M. Ishida, T. Nakajima, Y. Honda, O. Kitao, H. Nakai, T. Vreven, J. A. Montgomery, J. E. Peralta, F. Ogliaro, M. Bearpark, J. J. Heyd, E. Brothers, K. N. Kudin, V. N. Staroverov, R. Kobayashi, N. Normand, K. Raghavachari, A. Rendell, J. C. Burant, S. S. Iyengar, J. Tomasi, M. Cossi, N. Rega, J. M.

Millam, M. Klene, J. E. Knox, J. B. Cross, V. Bakken, C. Adamo, J. Jaramillo, R. Gomperts, R. E. Stratmann, O. Yazyev, A. J. Austin, R. Cammi, C. Pomelli, J. W. Ochterski, R. L. Martin, K. Morokuma, V. G. Zakrzewski, G. A. Voth, P. Salvador, J. J. Dannenberg, S. Dapprich, A. D. Daniels, Ö. Farkas, J. B. Foresman, J. V. Ortiz, J. Cioslowski and D. J. Fox, *Gaussian 09*, Revision D.01; Gaussian, Inc., Wallingford CT, 2013.

(23) (a) TURBOMOLE V7.0 2015, a development of University of Karlsruhe and Forschungszentrum Karlsruhe GmbH, 1989–2007, TURBOMOLE GmbH, since 2007. Available at: http://www.turbomole.com. Accessed on 9 April 2017; (b) F. Furche, R. Ahlrichs, C. Hättig, W. Klopper, M. Sierka and F. Weigend, *WIREs Comput Mol. Sci.* 2014, **4**, 91–100.
